# PLDα1-knockdown soybean seeds display higher unsaturated glycerolipid contents and seed vigor in high temperature and humidity environments

**DOI:** 10.1186/s13068-018-1340-4

**Published:** 2019-01-04

**Authors:** Gaoyang Zhang, Sung-Chul Bahn, Geliang Wang, Yanrui Zhang, Beibei Chen, Yuliang Zhang, Xuemin Wang, Jian Zhao

**Affiliations:** 1 State Key Laboratory of Tea Plant Biology and Utilization, College of Tea and Food Science and Technology, Anhui Agricultural University, Hefei, 230036 China; 2 University of Missouri at St Louis, Donald Danforth Plant Science Center, St. Louis, MO 63132 USA; 3 National Key Laboratory of Crop Genetic Improvement, Huazhong Agricultural University, Wuhan, 430075 China; 4 Key Laboratory of Biology and Genetic Resources of Tropical Crops. Ministry of Agriculture, Institute of Tropical Bioscience and Biotechnology, Chinese Academy of Tropical Agricultural Sciences, Haikou, Hainan 571101 China

**Keywords:** Phospholipase D, Glycerolipid, Oxidative stress, Acyl editing, Unsaturation, High temperature and humidity, Oil content, Seed vigor

## Abstract

**Background:**

Soybean oil constitutes an important source of vegetable oil and biofuel. However, high temperature and humidity adversely impacts soybean seed development, yield, and quality during plant development and after harvest. Genetic improvement of soybean tolerance to stress environments is highly desirable.

**Results:**

Transgenic soybean lines with knockdown of phospholipase Dα1 (*PLDα1KD*) were generated to study *PLDα1′*s effects on lipid metabolism and seed vigor under high temperature and humidity conditions. Under such stress, as compared with normal growth conditions, *PLDα1KD* lines showed an attenuated stress-induced deterioration during soybean seed development, which was associated with elevated expression of reactive oxygen species-scavenging genes when compared with wild-type control. The developing seeds of *PLDα1KD* had higher levels of unsaturation in triacylglycerol (TAG) and major membrane phospholipids, but lower levels of phosphatidic acid and lysophospholipids compared with control cultivar. Lipid metabolite and gene expression profiling indicates that the increased unsaturation on phosphatidylcholine (PC) and enhanced conversion between PC and diacylglycerol (DAG) by PC:DAG acyltransferase underlie a basis for increased TAG unsaturation in *PLDα1KD* seeds. Meanwhile, the turnover of PC and phosphatidylethanolamine (PE) into lysoPC and lysoPE was suppressed in *PLDα1KD* seeds under high temperature and humidity conditions. *PLDα1KD* developing seeds suffered lighter oxidative stresses than did wild-type developing seeds in the stressful environments. *PLDα1KD* seeds contain higher oil contents and maintained higher germination rates than the wild-type seeds.

**Conclusions:**

The study provides insights into the roles of PLDα1 in developing soybean seeds under high temperature and humidity stress. *PLDα1KD* decreases pre-harvest deterioration and enhances acyl editing in phospholipids and TAGs. The results indicate a way towards improving production of quality soybean seeds as foods and biofuels under increasing environmental stress.

**Electronic supplementary material:**

The online version of this article (10.1186/s13068-018-1340-4) contains supplementary material, which is available to authorized users.

## The significances statement

1. Developing soybean seeds suffer from high temperature and humidity stress conditions that often occur in Southern China and cause oxidative stress and seed pre-harvest deterioration. Such soybean seed deterioration can be alleviated by seed-specific knockdown of *phospholipase Dα1* (*PLDα1KD*). Thus, *PLDα1KD* soybeans have improved nutrition quality and seed vigor.
2. *PLDα1KD* developing seeds have higher levels of unsaturation in triacylglycerol and phospholipids due to enhanced expression of desaturase genes and enhanced acyl editing and PC–DAG conversion activities in *PLDα1KD* developing seeds compared with the wild-type seeds.
3. The finding that *PLDα1KD* seeds had improved seed vigor, nutrition quality, and tolerance to high temperature and humidity provides a molecular tool for genetic improvement of soybean for adaptation to wider growth conditions.

## Background

With global temperature increasing steadily in recent decades, high temperature conditions, accompanied either by drought or by humidity in different areas, caused damages and losses on crops and yield [1–4]. However, how high temperature and humidity affect crop growth, seed development, as well as yield is not well understood [2, 4]. The high temperature and humidity conditions adversely affect membrane lipids and storage triacylglycerol (TAG) in wheat [3, 5] and oilseed crops, such as soybean (*Glycine max*), particularly during seed development [6]. High temperatures impact developing soybean seeds’ sensitivity and vulnerability to stresses by causing seeds with poor germination, increased incidence of pathogen infection, and decreased economic value [6]. Previous studies indicated that phospholipase Dα (PLDα1) is involved in seed naturally and artificially aging, by affecting both phospholipids and TAG of mature Arabidopsis and soybean seeds [7–9]. However, how PLDα1 affects lipid metabolism and storage nutrition of developing soybean seeds grown under high temperature and humidity conditions was unknown [8, 10].

Assembly of phospholipids and TAG occurs primarily in the endoplasmic reticulum (ER) and shares a common biosynthetic precursor phosphatidic acid (PA) [11]. Membrane phospholipid synthesis is active in young and green tissues [12]. However, in developing oilseeds, phospholipid metabolism is overwhelmingly directed to TAG accumulation [13, 14], through two major pathways: the diacylglycerol acyltransferase (DGAT)-mediated Kennedy pathway that uses acyl-CoA and the diacylglycerol (DAG) to generate TAG, and phospholipid:diacylglycerol acyltransferase (PDAT)-mediated pathway that uses phosphatidylcholine (PC) and DAG to produce TAG [14]. Overexpression of an A-type phospholipase, *pPLAIIIδ*, enhanced TAG production in Arabidopsis and Camelina seeds [12, 15]. A-type phospholipase, PLA catalyzing the hydrolysis of PC to generate a free fatty acid and lysoPC (LPC), can also affect TAG production [12, 15]. Acyl-CoA:LPC acyltransferase (LPCAT) can modify PC saturation by introducing an acyl-CoA into a new PC [16, 17]. De novo PC biosynthesis from choline by the actions of choline/ethanolamine kinase (CEK), choline-phosphate cytidylyltransferase (CCT), and DAG cholinephosphotransferase (DAG:CPT) is also important for phospholipid and TAG metabolism [18]. The interconversion between PC and DAG by PC:DAG cholinephosphotransferase (PDCT, also ROD1) can significantly affect TAG biosynthesis and unsaturation through above mentioned pathways [16, 19, 20]. PDCT transfers phosphocholine from PC to DAG actively during oil seed development, and edits TAG composition using PC that is also extensively modified by fatty acid desaturases (FADs) on their acyl chains [19, 21, 22]. In the ER, the partitioning of PA, PC, and DAG precursors for TAG or phospholipid biosynthesis may be controlled by a unexplored complex network. For instance, it has been estimated that more than 70% PC-derived DAG is used to synthesize TAG in flax seeds [23]. Thus, PC plays multiple roles in TAG and phospholipid biosynthesis by recycling or incorporation of the newly synthesized fatty acids in TAG acyl editing [13].

PLD may contribute to diurnal cycling of PA, and PC acyl editing and significantly affect PC pools and TAG production [24–28]. A study demonstrated that PC-derived DAG is the major source for TAG synthesis in *Camelina* seeds overexpressing *PLDζ1* and *PLDζ2* [28]. Recently, a genome-wide association study suggested a *PLDα* gene as a key locus affecting oil biosynthesis in soybean [29, 30]. Studies indicated that PLD*α*1 is involved in the mature seed aging and deterioration stored under high temperature and humidity [7, 9]. Two *PLDα*-*RNAi* knockdown (*PLDα1KD*) soybean lines had had altered unsaturation fatty acids in both phospholipids and TAG when grown in Kansas in the United States [9]. However, it is still not understood why *PLDα1KD* seeds have such changes in fatty acyl chains saturation.

Although the anti-deterioration effects of *PLDα1* mutation on naturally or artificially aging Arabidopsis and soybean seeds have been reported [7–9], the underlying molecular mechanism by which *PLDα1* mutation affects lipids metabolism and seed quality has not been explored [8, 9]. Especially, how PLDα1 mutation affects soybean developing seeds under heat and humidity stresses is unknown. High temperature and humidity stresses occur frequently in southern China amid increasing global temperature in recent years and cause soybean pre-harvest deterioration and significant losses [6]. High temperature and humidity are major problems and limiting factors in soybean production area, and they not only caused soybean yield loss, but also affected adversely on seed storage and reduced nutrition [6]. In the Mid-Yangzi River region of China, soybean seed development and ripening occur during the season of high precipitations (~ more than 150 mm), high temperature (~ 34–38 °C), and high humidity (~ 75–80%) usually from the late June to the early September (Fig. 3a). The soybean seeds grown in these regions usually have pre-harvest deterioration, rapidly losing seed vigor, reducing nutrition quality severely, and are more vulnerable to pathogens during storage [6, 31]. A better understanding of the mechanism by which high temperature and humidity impact developing soybean seeds will help design effective genetic strategies to improve soybean tolerance to these environmental stress conditions. Here, we generated *PLDα1KD* soybean lines and investigated the *PLDα1KD* soybean performance in such stressful environments. The data indicate a critical role of PLDα1 in lipid metabolism and stress response in developing soybean seeds under high temperature and humidity stress conditions, as compared in normal environments.

## Results

### Generation of transgenic soybean plants with seed-specific knockdown of *PLDα1*

**Fig. 1:**
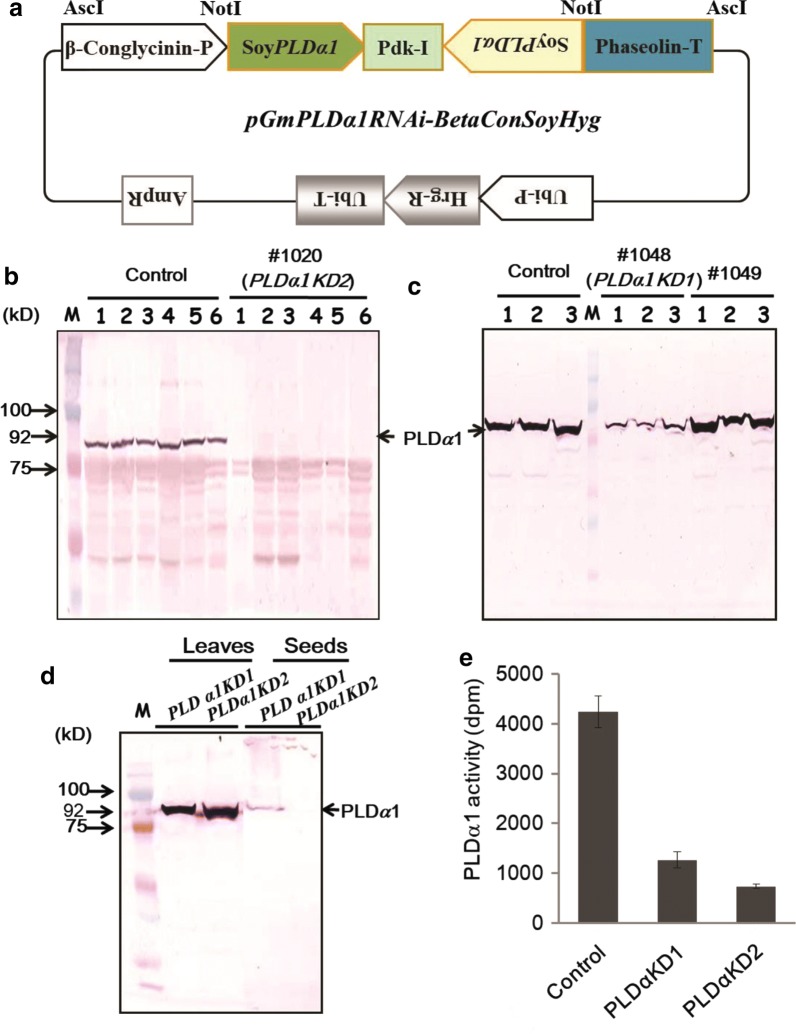
Generation of soybean *PLDα1* knockdown lines. Soybean *PLDα1KD* transgenic plants were generated by stable transformation of soybean cultivar Jack with a soybean *PLDα1* RNA interference (RNAi) construct. Transgenic soybean plants were used for analysis. **a** Construction of soybean *PLDα1KD*. 1151bp one in forward and the other in reverse and separated by a DNA fragment of *Pdk*-*I* gene, is driven by a seed specific promoter for soybean *β*-*conglycinin* gene encoding a major seed storage protein in seed, and terminated by a *Phaseo* gene terminator. **b** Immunoblotting screening PLDα1 in seeds of transgenic soybean plants 1020 (*PLDα1KD1*). 1–6 indicate individual plants from transgenic line or wild-type Jack (control). **c** Immunoblotting detection PLDα1 in seeds of another transgenic soybean line 1048 (*PLDα1KD2*). 1–3 indicate the individual plants from each transgenic line or wild-type Jack (control). **d** Immunoblotting of *PLDα1* using proteins extracted from leaves and seeds from two *PLDα1KD* lines1. **e** PLDα1 activity in two *PLDα1KD* lines 1 and wild-type Jack line1 (control) developing seeds

Soybean *PLDα1KD* transgenic plants were generated by stable transformation of soybean cultivar Jack with a soybean *GmPLDα1* RNA interference (RNAi) construct under the control of the seed-specific promoter β-conglycinin (Fig. 1a, Additional file 1: Figure S1). T0 and T2 transgenic plants were screened with qRT-PCR and immunoblotting with an antibody against Arabidopsis PLDα1. This antibody specifically recognized the ~ 92 kDa GmPLDα1 at both leaf and seed tissues of soybean (Fig. 1). The transgenic and background cultivar Jack seeds displayed different levels of PLDα1 accumulation (Fig. 1). Immunoblotting screening for regenerated transgenic soybean lines showed that in the line #1020 (*PLDα1KD2*), PLDα1 proteins in the developing and mature seeds were almost completely diminished by expression of *PLDα1RNAi* (Fig. 1b), whereas in another regenerated transgenic soybean line #1048 (*PLDα1KD1*) displayed about 25% of that in wild-type soybean cultivar Jack (Fig. 1c). To confirm the seed specific expression of *PLDα1RNAi* and seed-specific suppression of soybean PLDα1, proteins extracted from leaves and seeds of two *PLDα1KD* lines and wild-type control (Jack) were immunoblotted for PLDα1, and PLDα1 proteins remained in leaves of both transgenic lines, *PLDα1KD1* and *PLDα1KD2*, but was greatly diminished in their seeds (Fig. 1d). Assaying PLDα1 activity showed that PLD activity in *PLDα1KD1* and *PLDα1KD2* was 23% and 10% of that in wild- type, respectively (Fig. 1e).

### Effects of *PLDα1KD* on the expression of other *PLD*s

**Fig. 2:**
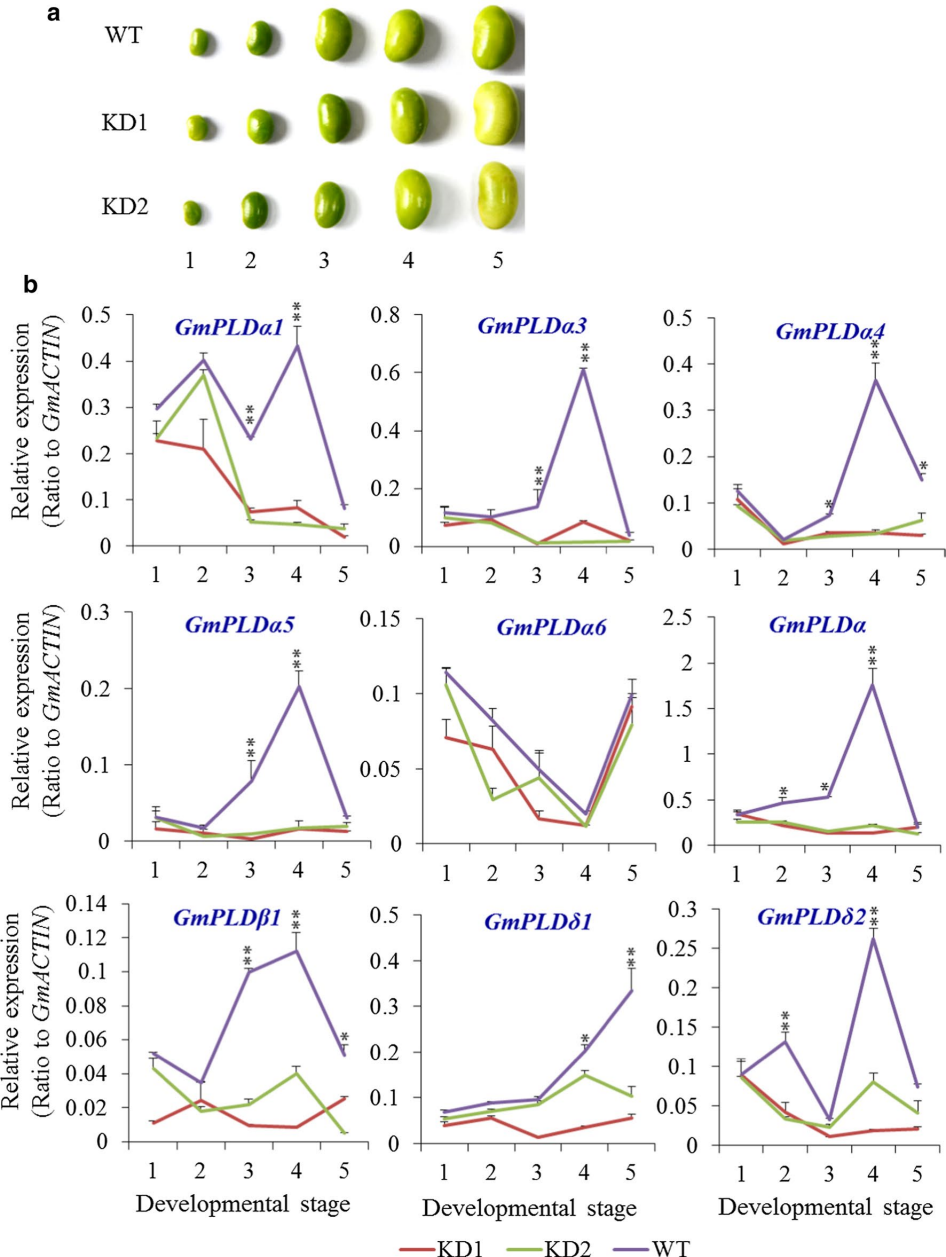
Expression profiles of *GmPLD* genes in *PLDα1KD* and wild-type developing seeds. KD1*: PLDα1KD* line 1; KD2: *PLDα1KD* line 2. 1, 2, 3, 4, 5 indicate different developing stages of seeds, corresponding to fresh weights: stage 1, 30–70 mg; stage 2, 100–150 mg; stage 3, 200–250 mg; stage 4, 300–350 mg; stage 5, 400–480 mg. Data are mean ± S.D. (*n* = 3). * and **Denote significance at *P* < 0.05 and *P* < 0.01, respectively, compared with controls on Student’s *t* test. **a** Appearances of *PLDα1KD* and wild-type Jack (WT) seeds at different developmental stages. **b** Expression profiles of major *PLD*s during seed development. Total RNA was extracted from developing seeds at different developmental stages

The soybean PLD family has 23 members (Additional file 1: Figure S2a, Additional file 2: Table S2, Data S1). To evaluate the effect of *PLDα1KD* on the expression patterns of different PLDs, developing seeds of *PLDα1KD* from T3 and T5 generations of *PLDα1KD* transgenic lines were tested (Fig. 2). Three *GmPLDα*s are highly expressed (Additional file 2: Table S3) [32]. In *PLDα1KD* mutants, the expression of various *PLDα*s, including targeted *PLDα1*s, was reduced significantly compared with that of control at different developmental stages (Fig. 2, Additional file 1: Figure S2c, d). In control Jack seeds, *GmPLDα3* is the most highly expressed, *GmPLDα1* is the second highest, and *GmPLDα4* is the third most highly expressed *PLD* gene in developing seeds (Fig. 2, Additional file 1: Figure S2b, Additional file 2: Table S4) [33]. When using a pair of primers that amplify all *GmPLDα*s, the total *GmPLDα* expression displayed a similar pattern as that for the above major *GmPLDα*s. *GmPLDβ3* transcripts were lower in *PLDα1KD* mutants than in wild-type during early stages of seed development, but higher than in Jack at late stages of seed development (Additional file 1: Figure S2c). Among *GmPLDβ*s, *GmPLDβ4* showed the highest expression level in developing seeds, and then *GmPLDβ1*. Most other *PLDβ*s were expressed at a low level in soybean developing seeds (Fig. 2, Additional file 1: Figure S2b, Additional file 2: Table S4) [33]. Among two major *GmPLDδs* expressed in developing seeds, *GmPLDδ1* was lower at the stages of 4 and 5, and *GmPLDδ2* was lower at the satges 2 and 5 than those in the wild-type (Fig. 2). The results indicate that *GmPLDα1* RNAi also interfered with the transcripts of other *PLD* genes, likely due to the high sequence similarity among *PLD* genes.

### *PLDα1KD* developing seeds had elevated levels of ROS-scavenging genes

**Fig. 3:**
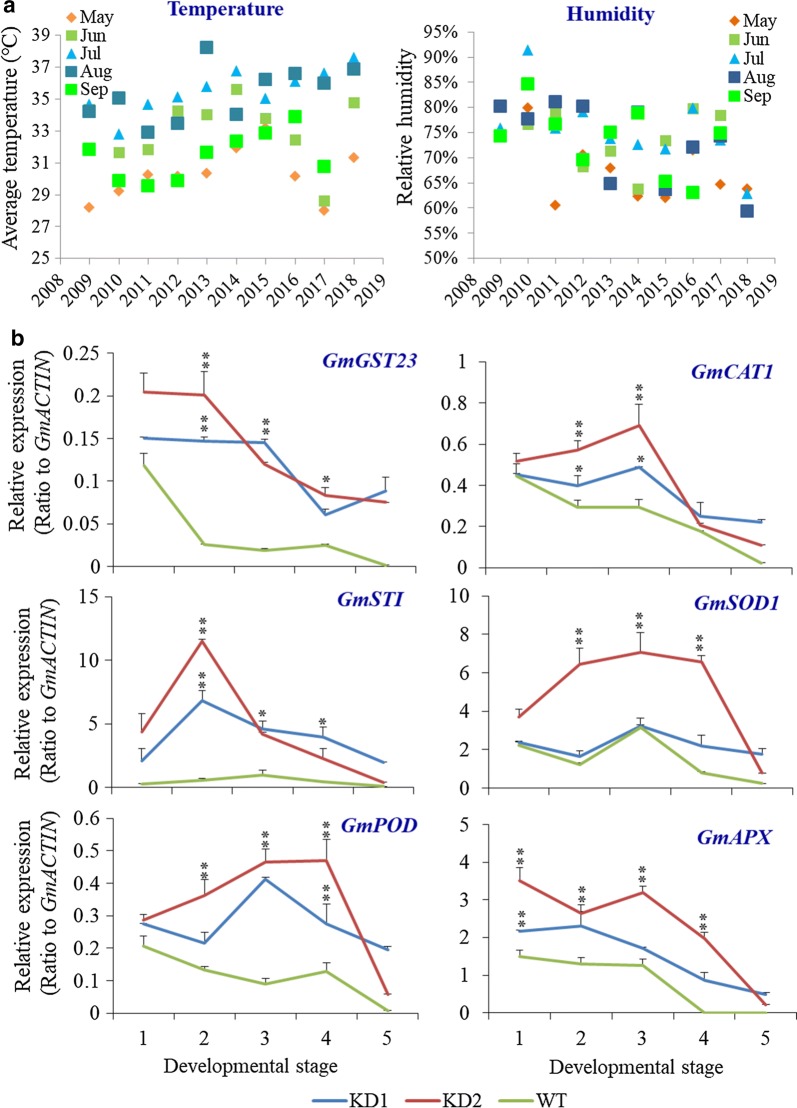
Weather conditions in Mid-Yangzi River regions and the heat stress-induced the oxidative stress and ROS-scavenging genes in developing seeds. **a** Temperature and humidity changes in Mid-Yangzi River in past 10 years. The data were downloaded from website https://tianqi.911cha.com. **b** Expression of the stress-related genes in wild-type Jack (WT) and *PLDα1 KD* seeds grown in high temperature and humility condition were examined with qRT-PCR. KD1: *PLDα1* knockdown line 1; KD2: *PLDα1* knockdown line 2. 1, 2, 3, 4, 5 indicate different developing stages of seeds, corresponding to fresh weights, described as above. The values are the mean ± SD (*n* = 3). * and **Denote significance at *P* < 0.05 and *P* < 0.01, respectively, compared with wild-type Jack (WT) based on Student’s *t* test

Most plant tissues under abiotic stress conditions, such as drought, salinity, ozone, high temperature, and flooding, usually generate more reactive oxygen species (ROS), which is often associated with the synthesis of more enzymes involved in ROS-scavenging to reduce the oxidative damage [6, 34–36]. Under high temperature and humidity, wild-type developing seeds displayed increased expression levels of ROS-scavenging related genes, indicating that seeds might be faced with elevated ROS production (Additional file 1: Figure S3). The expression of several genes, such as glutathione S-transferases (*GST23*), peroxidase (*POD*), catalase (*CAT1*), superoxide dismutase (*SOD1*), ascorbic acid peroxidase (*APX*), as well as a heat shock protein *STI* [6, 35], were highly induced over the time during high temperature and humidity stress (Additional file 1: Figure S3). The level of *GmPLDα1* transcript also increased by ninefold at 6 h after heat treatment, compared with seeds under normal temperature (Additional file 1: Figure S3). Most stress-responsive and ROS-scavenging genes, such as *GST23*, *POD*, *CAT1*, *SOD1*, *STI*, and *APX*, in *PLDα1KD1* and *PLDα1KD2* developing seeds displayed higher transcript levels than those in wild-type control under high temperature and humidity conditions (Fig. 3b).

### Increased unsaturated fatty acids of TAG and phospholipids in *PLDα1KD* seeds

**Fig. 4:**
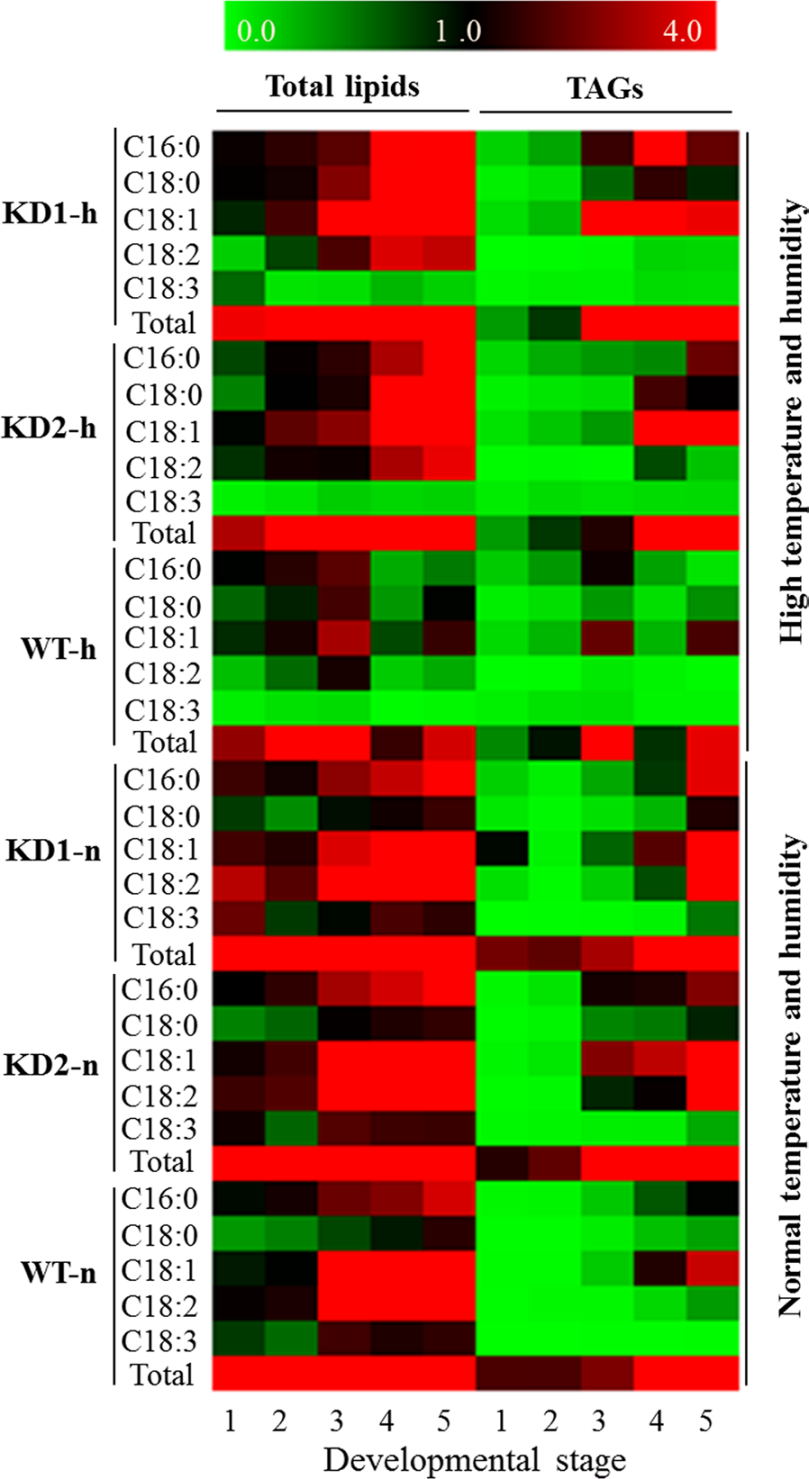
Heatmap analyses on fatty acid compositions of total lipids and TAGs from *PLDα1KD* and wild-type developing seeds. Exact quantification data see Additional file 1: Figure S4 for details. The values represented are the mean ± SD from at least three independent repeats. KD1-h, KD2-h and WT-h: *PLDα1* knockdown line 1, 2 and wild-type jack, respectively, under high temperature and humidity which used thick lines; KD1-n, KD2-n and WT-n: *PLDα1* knockdown line 1, 2 and wild-type Jack, respectively, under normal temperature and humidity. 1, 2, 3, 4, and 5 indicate different developing stages of seeds, corresponding to fresh weights as described previously. The values are from the mean ± SD (*n* = 3)

We examined changes of TAG contents and fatty acid composition of *PLDα1KD* and wild-type soybean lines at different developmental stages. Total fatty acid content steadily increased over the seed filling during maturation (Fig. 4, Additional file 1: Figure S4). In addition, clear differences between *PLDα1KD* and wild-type seeds in unsaturated fatty acids, 18:1, 18:2, and 18:3, were detected throughout the seed developmental stages. Wild-type soybean oil contains 13% palmitic acid (16:0), 4% stearic acid (18:0), 20% oleic acid (18:1), 55% linoleic acid (18:2), and 8% linolenic acid (18:3) (Fig. 4, Additional file 1: Figure S4). A higher content of unsaturated fatty acids in TAG was observed in *PLDα1KD* seeds than those in the wild-type Jack under normal conditions (Fig. 4, Additional file 1: Figure S4). The differences became bigger between *PLDα1KD2* and wild-type than those between *PLDα1KD1* and wild-type under stress conditions, suggesting that the degree of *PLDα1* suppression may be proportional to the content of unsaturated fatty acids. To distinguish whether the difference in unsaturation resulted from fatty acids in TAG or phospholipids, we assayed fatty acid composition in TAG and phospholipids separated by TLC. The total TAG content in *PLDα1KD* mutant seeds was higher than that in wild-type seeds at most developmental stages (Fig. 4, Additional file 1: Figure S4). Correspondingly, the contents of total unsaturated fatty acids (mainly 18:1 and 18:2) in stage 5-seeds of *PLDα1KD1 and 2* mutant lines were comparable. Both were about two and tenfold higher than those of wild-type seeds under normal growth conditions and the high temperature and humidity conditions, respectively (Fig. 4, Additional file 1: Figure S4). The total fatty acid content of WT under normal conditions was 1.5-fold higher than that under high temperature and humidity in stage-3 seeds. This result is consistent with a previous observation that high temperature decreased oil content of soybean seeds [6]. However, the total fatty acid content in developing *GmPLDα1KD* seeds was almost unchanged, which could mean that the knockdown of *GmPLDα1* stabilizes the seed fatty acid contents under high temperature (Fig. 4, Additional file 1: Figure S4).

### Up-regulation of *FAD*s in *PLDα1KD* developing seeds as compared with wild-type

**Fig. 5:**
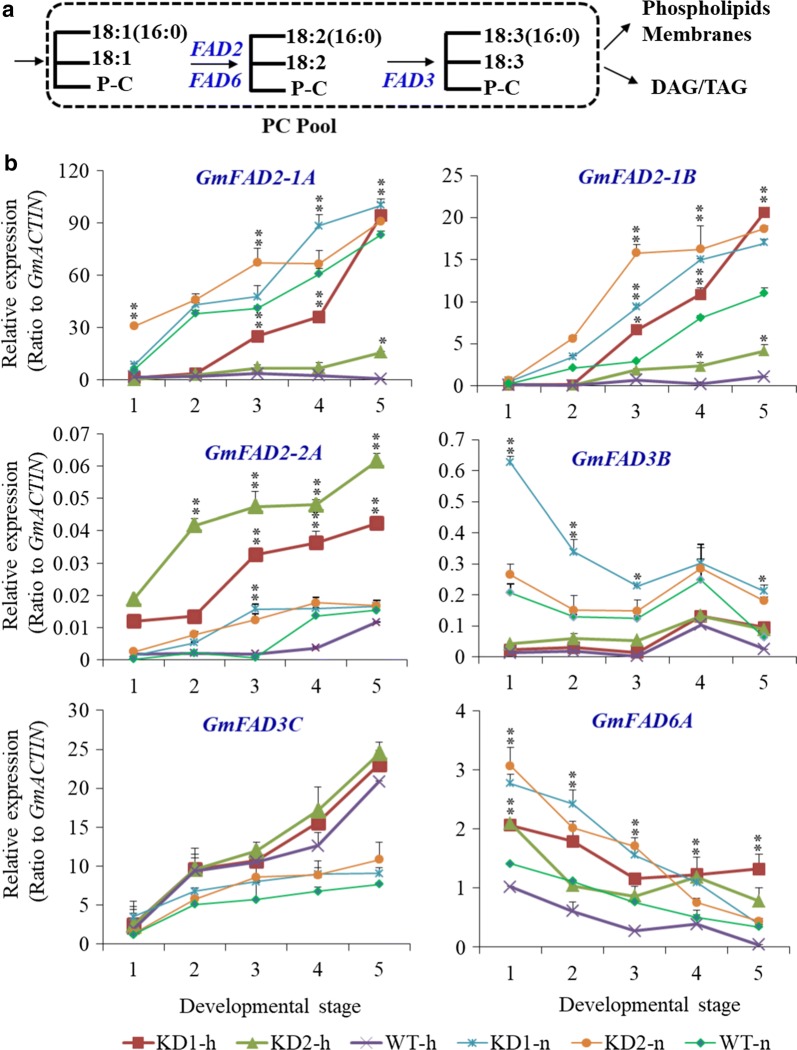
Expression patterns of *FAD* genes in *PLDα1KD* and wild-type developing seeds. **a** FAD enzymes function in phospholipid desaturation with various PC molecules as preferred substrates. **b** Expression of *FAD* genes in soybean developing seeds of different genetic backgrounds under various growth conditions. FAD2, Microsomal Δ12 desaturase; FAD3, Microsomal ω3 desaturase; FAD6, Plastidial Δ12 desaturase. The *X*-axis numbers indicated each development stage. KD1-h, KD2-h and WT-h: *PLDα1* knockdown line 1 and 2 and wild-type jack, respectively, under high temperature and humidity; KD1-n and KD2-n and WT-n: *PLDα1* knockdown line 1 and 2 and wild-type Jack, respectively, under normal temperature and humidity. 1–5 indicate different developing stages of seeds corresponding to fresh weights as described in “Materials and methods”. The values are the mean + SD (*n* = 3). * and **Denote significance at *P* < 0.05 and *P* < 0.01, respectively, compared with wild-type Jack (WT) based on Student’s *t* test

In the ER, FAD2 synthesizes linoleic acid from oleic acid and FAD3 catalyzes the conversion of linoleic acid into α-linolenic acid on PC (Fig. 5a). *FAD2*-*2A* (Glyma.19G147400), *FAD2*-*2B* (Glyma.19G147300), *FAD2*-*2C* (Glyma.15G195200), and *GmFAD2*-*2D* (Glyma.03G144500) were constitutively expressed in developing seeds and vegetative tissues of soybean [37–39] whereas *FAD2*-*1A* (Glyma.10G278000) and *FAD2*-*1B* (Glyma.20G111000) are specifically expressed in developing seeds, and play an essential role in controlling the oleic acid level in developing soybean seeds (Additional file 1: Figure S5) [32, 33]. The low-linolenic acid trait in soybean requires the combination of up to three different recessive alleles of *FAD3* genes that encode omega-3 fatty acid desaturases [40, 41]. *GmFAD3* includes *GmFAD3A* (Glyma.14G194300), *GmFAD3B* (Glyma.02G227200), and *GmFAD3C* (Glyma.18G062000).

Quantitative RT-PCR results showed that the expression of *FAD2*-*1B* was much higher in developing seeds of *PLDα1KD* than in wild-type under both normal and stress conditions (Fig. 5b). Microarray data showed that *FAD2*s were highly expressed in seeds during development [33]. *FAD2*-*1B* and *FAD2*-*1A* were mainly expressed at the late stages of developing seeds, and their transcripts were higher in *PLDα1KD* than wild-type under both conditions. The transcript levels of *GmFAD3A*, *GmFAD3B* and *GmFAD3C* were generally high in developing seeds (Fig. 5b, Additional file 1: Figure S5). Under high temperature, *FAD3B* expression levels increased to the highest level at the stage 4, and then decreased. However, under normal conditions, the *FAD3B* transcript level was higher at all developmental stages compared with that under stressed conditions. Furthermore, the *FAD3B* transcript level was much higher in *PLDα1KD1* than wild-type seeds under normal conditions (Fig. 5b). The expression levels of the major *FAD*s in seeds were higher under normal conditions than high temperature conditions, except for *GmFAD2*-*2A* and *GmFAD3C*, whose expression levels were generally low under normal conditions, and increased in response to elevated temperatures. The expression of chloroplast localized *GmFAD6A* was also higher in *PLDα1KDs* than in wild-type and up-regulated under stress conditions (Fig. 5b).

### Increased contents of PC and PE in *PLDα1KD* seeds

**Fig. 6:**
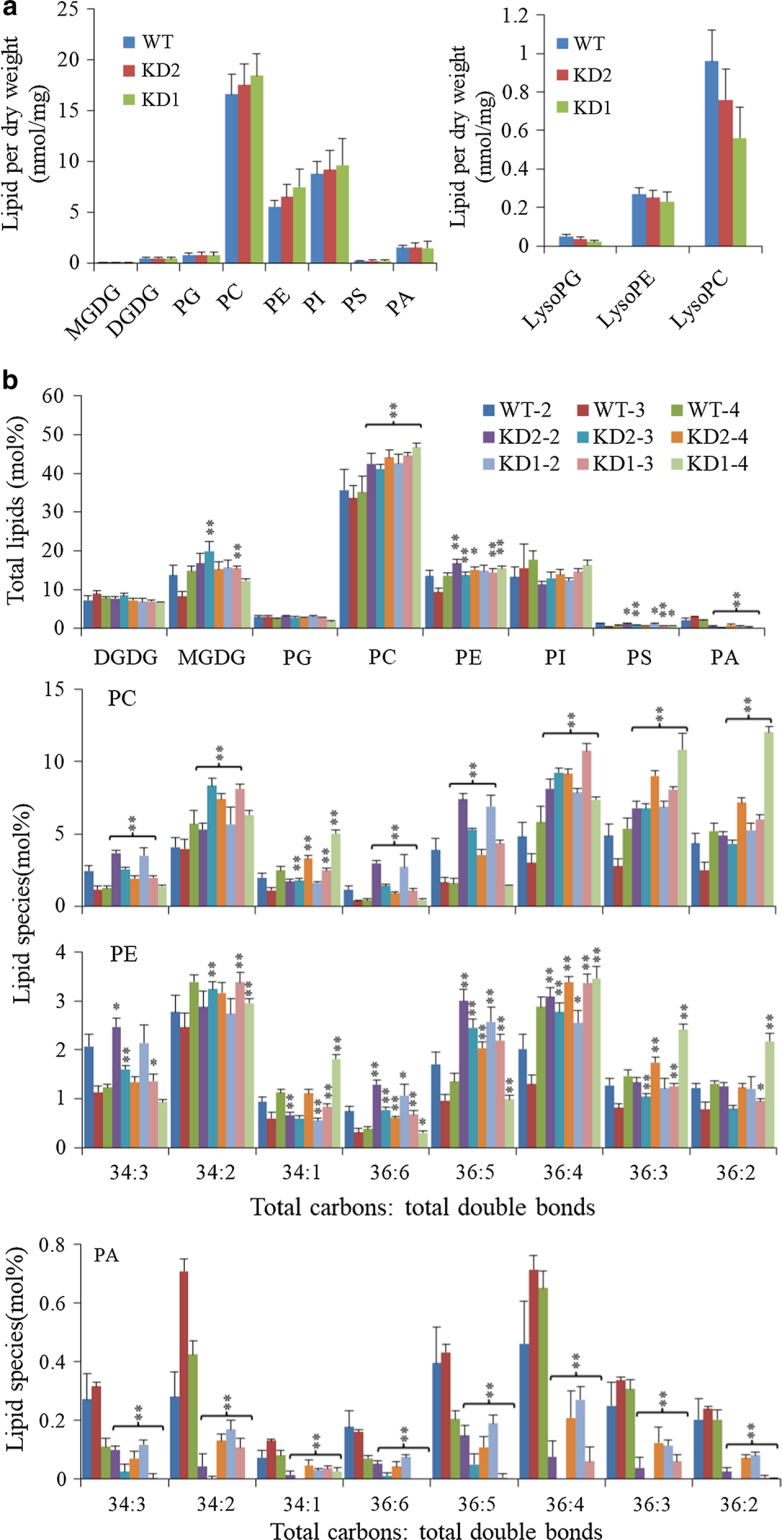
Phospholipid profiles in *PLDα1KD* and wild-type developing seeds. **a** Profiles of phospholipids in mature seeds of KD (*PLDα1KD*) and wild-type Jack (WT) by ESI-MS/MS. All seeds collected from soybean plants grown under normal conditions. **b** Total lipid contents of major lipids and the molecular species (total acyl chains: double bonds). All plants were grown under high temperature and humidity. All plants were grown under high temperature and humidity. The values are the mean ± S.D. (*n* = 4 or 5). WT-2: wild-type Jack at stage 2; KD1-2: knockdown line 1 at stage 2; KD2-2: knockdown line 2 at stage 2; The rest can be explained in the same manner. All * and **denote significance at *P* < 0.05 and *P* < 0.01, respectively, compared with wild-type Jack (WT) based on Student’s *t* test

**Fig. 7:**
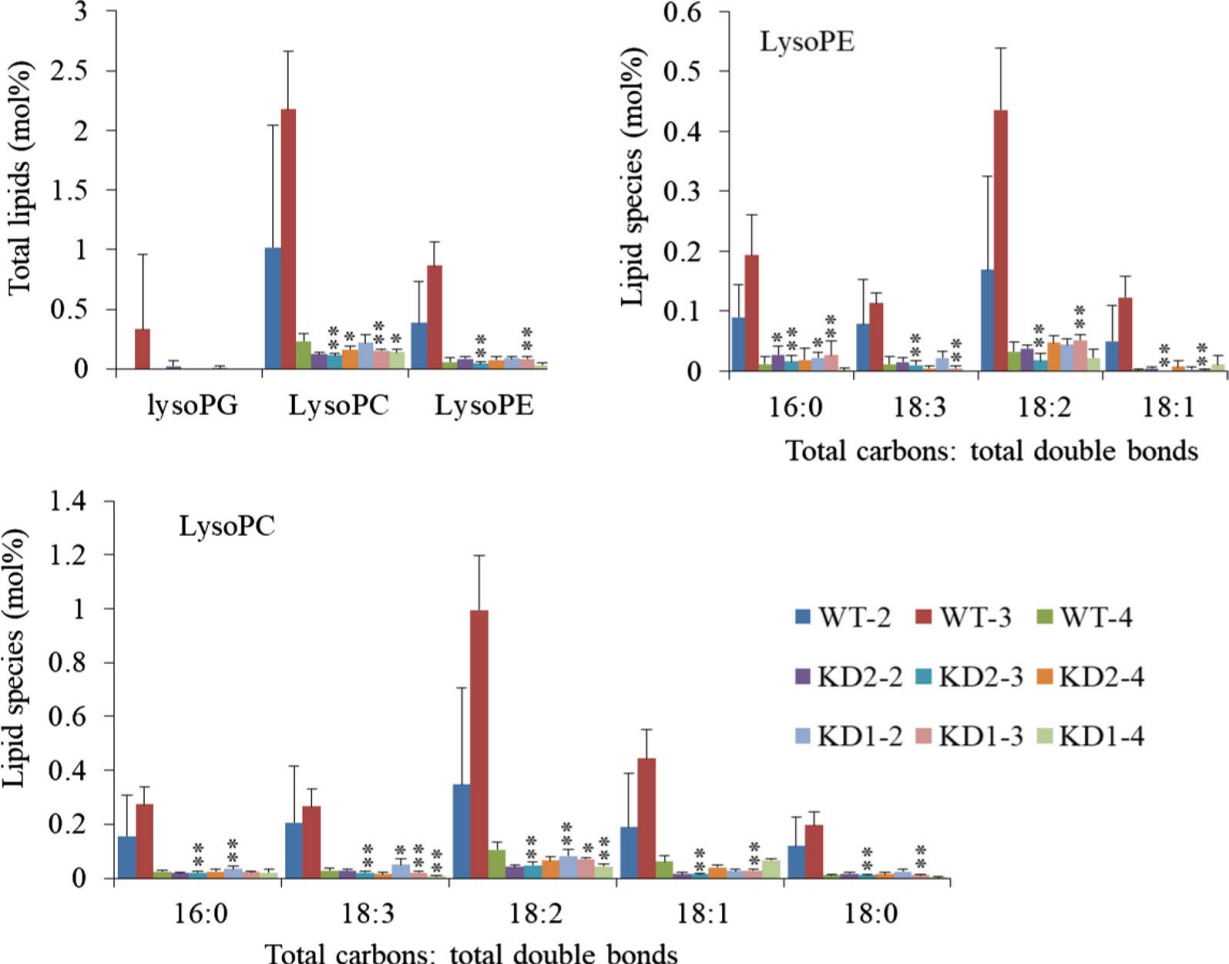
Lysophospholipid profiles in *PLDα1KD* and wild-type developing seeds. Total content of lysoPG, lysoPC, lysoPE and corresponding molecule species were measured by using ESI-MS/MS. All plants were grown under high temperature and humidity. The values are the mean ± S.D. (*n* = 4 or 5). WT-2: wild-type Jack at stage 2; KD1-2: knockdown line 1 at stage 2; KD2-2: knockdown line 2 at stage 2; The rest can be explained in the same manner. All * and **denote significance at *P* < 0.05 and *P* < 0.01, respectively, compared with wild-type Jack (WT) based on Student’s *t* test

The contents of PC and PE between wild-type and both PLDα1KDs lines were comparable under normal growth conditions (Fig. 6a), but they became different under high temperature and humidity conditions (Fig. 6b). To compare the unsaturation status of phospholipids in *PLDα1KD* seeds with wild-type at different developmental stages under stress conditions, we profiled phospholipids at developmental stages 2, 3, and 4 as *PLDα1* expression was higher at these stages. The level of most phospholipids did not change substantially over these three stages (Fig. 6b). However, seeds from *PLDα1KD1* and *2* plants had averagely 132% PC and 47% PE higher than those from wild-type in stage-3 seeds (Fig. 6b). The level of PC, particularly with unsaturated fatty acid acyl chains, was significantly higher in *PLDα1KD* seeds than wild-type. *PLDα1KD* seeds had higher levels of PCs and PEs with 36:5, 36:6, 36:4, 36:3, 36:2 34:3, 34:2 (total acyl carbons: total acyl double bonds) acyl chains (Fig. 6b). The PA level in *PLDα1KD* seeds at all three stages was much lower than that in wild-type, with reduction of more than 81% (Fig. 6b). In addition, total levels of LPC, lysophosphatidylethanolamine (LPE), and lysophosphatidylglycerol (LPG) were lower in *PLDα1KD* seeds than in wild-type seeds, with LPC and LPE being decreased by 90%, in stage-3 seeds (Fig. 7). Overall MGDG increased in both *PLDα1KD* seeds compared to wild-type seeds, whereas DGDG content kept unchanged. The difference of PC contents between *GmPLDα1KD* and wild-type seeds under high temperature and humidity was larger than those under normal conditions (Figs. 6, 7).

### Decreased PA pools in *PLDα1KD* seeds

**Fig. 8:**
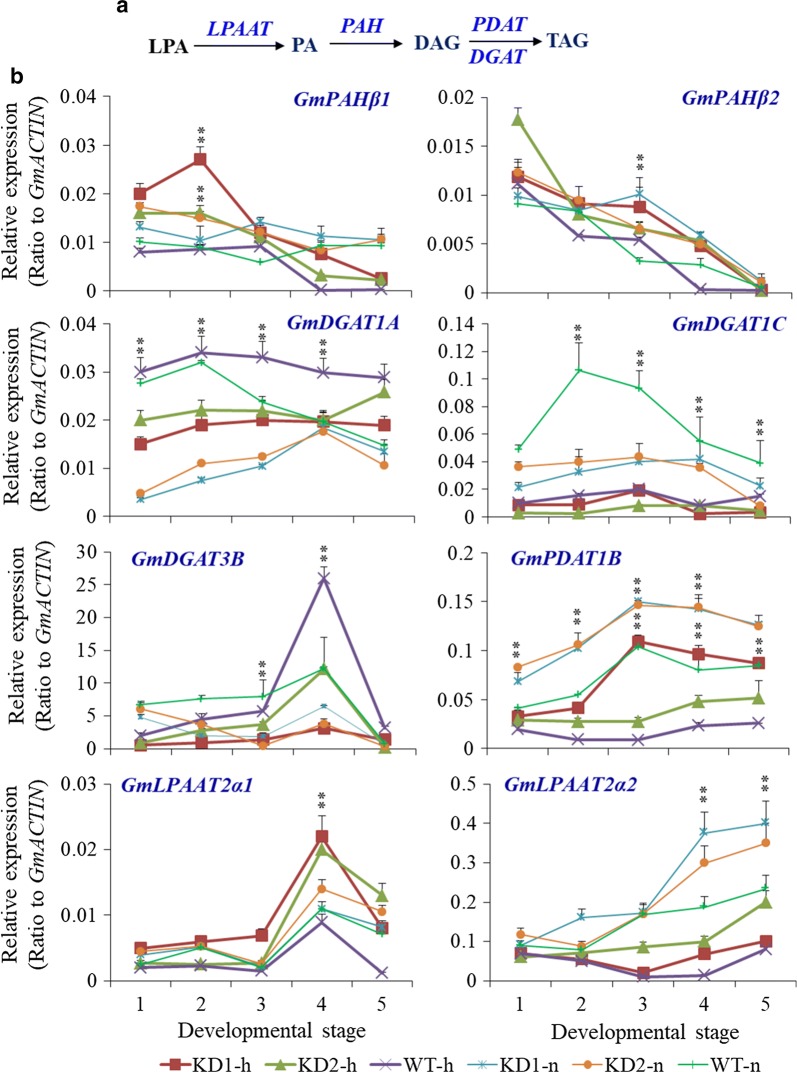
Expression profiles of TAG biosynthesis genes in *PLDα1KD* and wild-type developing seeds. **a** Kennedy pathway for TAG biosynthesis in the ER. **b** Expression of major genes involved in the Kennedy pathway in soybean developing seeds of different genetic backgrounds under various growth conditions. The *X*-axis numbers indicated each development stage. KD1-h, KD2-h and WT-h: PLDα1 knockdown line 1, 2 and wild-type Jack, respectively, under high temperature and humidity which used thick lines; KD1-n, KD2-n and WT-n: PLDα1 knockdown line 1, 2 and wild-type Jack, respectively, under normal temperature and humidity which used thin lines. 1, 2, 3, 4, and 5 indicate different developing stages of seeds, corresponding to fresh weights as described previously. The values are the mean ± SD (*n* = 3). * and **Denote significance at *P* < 0.05 and *P* < 0.01, respectively, compared with wild-type Jack (WT) based on Student’s *t* test

**Fig. 9:**
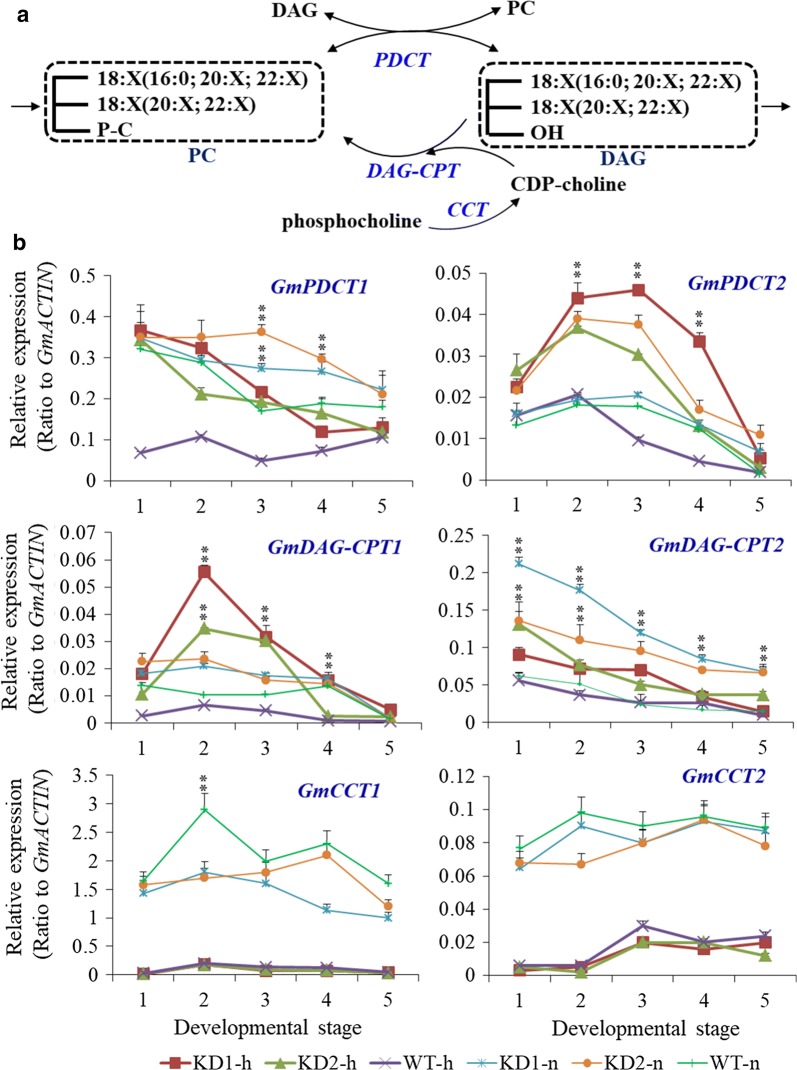
Differential expression patterns for genes involved in PC–DAG conversion in *PLDα1KD* and wild-type developing seeds. **a** Acyl editing in phospholipid and TAG through PC–DAG conversion. **b** Differential expression of genes involved in PC–DAG conversion in developing seeds of different genetic backgrounds under various growth conditions. The *X*-axis numbers indicated each development stage. KD1-h, KD2-h and WT-h: PLDα1 knockdown line 1, 2 and wild-type Jack, respectively, under high temperature and humidity which used thick lines; KD1-n, KD2-n and WT-n: PLDα1 knockdown line 1, 2 and wild-type Jack, respectively, under normal temperature and humidity which used thin lines. 1, 2, 3, 4, and 5 indicate different developing stages of seeds, corresponding to fresh weights as described previously. The values are the mean ± SD (*n* = 3). * and **Denote significance at *P* < 0.05 and *P* < 0.01, respectively, compared with wild-type Jack based on Student’s *t* test

**Fig. 10:**
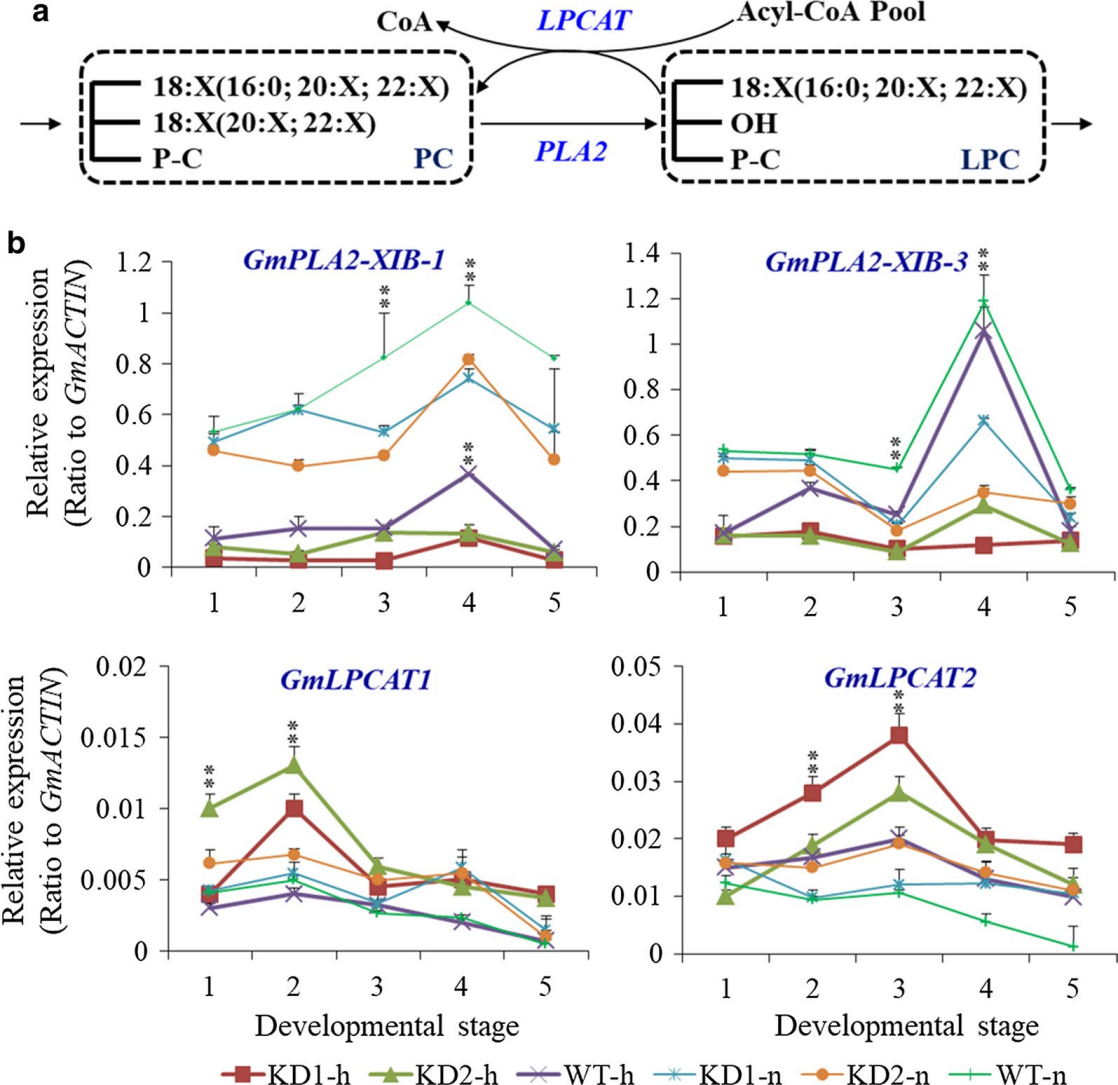
Expression of *PLAs* and *LPCATs* involved in acyl editing in *PLDα1*KD and wild-type developing seeds. **a** Acyl editing in PC fatty acyl chains through PLA–LPCAT cycle. **b** Expression of major *PLA* and *LPCAT* genes involved in developing seeds of different genetic backgrounds under various growth conditions. The *X*-axis numbers indicated each development stage. KD1-h, KD2-h and WT-h: PLDα1 knockdown line 1, 2 and wild-type Jack under high temperature and humidity which used thick lines; KD1-n, KD2-n and WT-n: PLDα1 knockdown line 1, 2 and wild-type Jack under normal temperature and humidity which used thin lines, respectively. 1, 2, 3, 4, and 5 indicate different developing stages of seeds, corresponding to fresh weights as described previously. The values are the mean ± SD (*n* = 3). * and **Denote significance at *P* < 0.05 and *P* < 0.01, respectively, compared with wild-type Jack (WT) based on Student’s *t* test

We further investigated the expression of genes involved in PA biosynthesis- and catabolism in *PLDα1KD* seeds (Fig. 8a). *LPAATs* that produce PA from lysoPA, PA hydrolases (PAHs) that dephosphorylate PA to yield DAG, and PLDs that produce PA from hydrolysis of phospholipids, all contribute to the changes of PA levels. The soybean genome contains multiple genes encoding *LPAATs*, corresponding to Arabidopsis *AtLPAAT* 1–5 that are essential enzymes for the de novo PA biosynthesis in both eukaryotic and prokaryotic pathways for glycerolipid biosynthesis [42, 43]. LPAAT function in TAG biosynthesis in soybean and Arabidopsis has been implicated [16]. In soybean developing seeds, the major *GmLPAAT* transcripts, including *GmLPAAT2α1*, *GmLPAAT2α2*, accumulated in similar patterns compared with *DGAT*, *PDAT*, or other TAG biosynthesis-related genes, which fluctuated in developing seeds (Figs. 8, 9, 10, Additional file 1: Figure S6) [33]. These *GmLPAAT* transcripts increased more than 21% in *PLDα1KD* seeds compared to wild-type at developmental stage 5 under both conditions. Both *LPAAT2α1* and *LPAAT2α2* genes were down-regulated in wild-type, and in both *PLDα1KD* lines, the transcript level of *LPAAT2α2* was down-regulated but *LPAAT2α1* remained high in response to high temperature and humidity stress (Fig. 8b).

Three *PAH* genes, homologous to *AtPAH1* and *AtPAH2*, are present in the soybean genome. Two of them, *Glyma.13G134500* (*GmPAHβ1*) and *Glyma.10G046400* (*GmPAHβ2*), were highly expressed in developing seeds, in a trend coincident with seed oil accumulation (Additional file 1: Figure S7) [33]. The transcript levels for both *GmPAHβ1* and *GmPAHβ2* in PLDα1KD were one and twofold higher, respectively, than these in wild-type seeds at stages 4 under both conditions and the expression of *GmPAHs* was not affected at all by high temperature except *GmPAHβ1* at stage 2 and *GmPAHβ2* at stage 3 (Fig. 8b). The combined effects of suppressed *PLDα1KD*, and a markedly higher *PAH* expression level contributed to the decreased PA levels, which was confirmed by mature and developing seeds (Figs. 6, 7).

### Altered transcript levels of *DGATs* and *PDAT*s for TAG biosynthesis in *PLDα1KD* seeds

To explore how *PLDα1KD* affected TAG biosynthesis and phospholipid metabolism in soybean seeds, we examined several major genes involved in the Kennedy pathway (Fig. 8b). DGAT synthesizes TAG by transferring an acyl group to DAG from newly synthesized or recycled acyl-CoA (Fig. 8a). The DGAT family in the soybean genome has 10 members. Type 1 DGATs, *Glyma.13G106100*, *Glyma.09G065300*, and *Glyma.17G053300*, were highly expressed in seeds. Type 3 DGAT *Glyma.17G041600* was also highly expressed in seeds. Compared with type 1 and type 3 DGATs, type 2 DGAT, such as *Glyma.16G115700* and *Glyma.09G195400*, were expressed at a lower level in seeds [44]. The transcript level of these genes increased steadily over seed development (Additional file 1: Figure S8) [33]. *PLDα1KD* lines have lower transcript levels for several seed-specific *DGAT*s, such as *GmDGAT1A* (Glyma.13G106100), *GmDGAT1C* (Glyma.09G065300) and *GmDGAT3B* (Glyma.17G041600), over all developmental stages under both conditions, indicating that *PLDα1KD* lines have reduced contributions through *DGAT* pathway towards TAG synthesis (Fig. 8b). Meanwhile, the expression of *GmDGAT1A* and *GmDGAT3B* was increased whereas the expression of *GmDGAT1C* was suppressed in *PLDα1KD* lines and wild-type under high temperature conditions compared with normal conditions.

PDAT transfers the *sn*-2 acyl group from phosphatidylcholine or phosphatidylethanolamine to DAG for TAG production in plants and yeast (Fig. 8a) [45, 46], and in soybean seeds, DAG from PC is primarily used for TAG biosynthesis. PDAT and DGAT were shown to have overlapping functions in TAG biosynthesis [47]. The soybean genome contains 6 putative *PDAT* genes, and among them, *Glyma.12G084000*, *Glyma.11G190400*, and *Glyma.13G108100*, as well as *Glyma.07G036400*, were highly expressed in seeds. Transcripts of these *PDATs* increased steadily during seed development except *Glyma.07G036400* (Additional file 1: Figure S9) [33]. The transcript level of *PDAT* genes in *PLDα1KD* seeds was more than higher than those in wild-type seeds from stages 3 to 5 under both conditions, suggesting that *PLDα1KD* seeds have increased PDAT-mediated, DAG-PC dependent TAG biosynthesis (Fig. 8b). Meanwhile, high temperature and humidity suppressed the expression of *GmPDAT1B* in both *PLDα1KD* and wild-type seeds.

### Enhanced PC–DAG conversion and acyl editing in *PLDα1KD* soybean seeds

To test whether the active PC–DAG–PDAT pathway contributed to more TAG biosynthesis in *PLDα1KD* than in wild-type, we compared the transcript levels of relevant genes. For PC synthesis, choline/ethanolamine kinase (CEK) produces phosphocholine that is used by CTP: phosphocholine cytidylyltransferase (CCT) to synthesize CDP- choline (Additional file 1: Figures S10, S11). DAG: cholinephosphotransferase (CPT) then transfers choline form CDP-choline to DAG to generate PC (Fig. 9a). The soybean genome has two CCT genes, *CCT1* (*Glyma.09G051200*) and *CCT2* (*Glyma.15G157500*), and their transcript levels fluctuated during seed development (Fig. 9b, Additional file 1: Figure S11) [33]. However, transcripts of *CCT*s in *PLDα1KD* lines were down-regulated by approximately 18% at stage 3 under both conditions. The expression of *GmCCTs* was suppressed in both *PLDα1KD* lines and wild-type seeds under high temperature and humidity conditions.

DAG-CPT and PDCT form an important PC–DAG exchange/conversion cycle to enforce the acyl editing of TAGs (Fig. 9a). In *PLDα1KD* developing seeds, *DAG:CPTs* (also called AAPTs), *DAG:CPT1* and 2 (*Glyma.12G081900* and *Glyma.02G128300*, respectively), were up-regulated as compared with those in developing seeds of wild-type under both conditions (Fig. 9b, Additional file 1: Figure S12). The soybean genome contains two *PDCT* genes, *GmPDCT1* (*Glyma.07G029800*) and *2* (*Glyma.08G213100*). The two genes were highly expressed in developing soybean seeds (Additional file 1: Figure S13) [33]. The transcript of *GmPDCT2* was up-regulated at early developmental stages and then decreased during late seed stages under both conditions. *GmPDCT1* and *2* were significantly up-regulated at stages 2-3 in *PLDα1KD* developing seeds under both conditions (Fig. 9b). The expression of *GmPDCT1* and *GmDAG*-*CPT2* was suppressed at stages 3-5 in *PLDα1KD* developing seeds under high temperature and humidity conditions. Other two genes *GmPDCT2* and *GmDAG*-*CPT1* displayed complicated expression patterns in wild-type and *PLDα1KD* developing seeds in both environments. These data suggest that the activity of PC and DAG interconversion is increased when *PLDα1* was suppressed in developing soybean seeds.

### Reduced transcript levels of *PLA*s but increased levels of *LPCAT* in *PLDα1KD* seeds

Since pPLA affects TAG biosynthesis [15], we examined the expression of *pPLAs* that were either specifically or highly expressed in the developing soybean seeds. The soybean genome contains a large *pPLA* gene family, and several *pPLA*s were highly expressed in developing seeds, such as *Glyma.08g028800*, *Glyma.11g036900*, and *Glyma.17G145900.* The *pPLA*s, *Glyma.18g251500* and *Glyma.09g243100*, were expressed only in seeds (Additional file 1: Figure S14) [32, 33]. The transcripts of *pPLA*s (*Glyma01G.002400* and *Glyma08G.028800*) in *PLDα1KD* soybean seeds were, on average, 44% lower than those in wild-type seeds at stage-4 under both conditions, which was consistent with higher levels of PCs in *PLDα1KD* soybean seeds and lower levels of lysophospholipids (Figs. 6, 7, 10b). Meanwhile, high temperature and humidity suppressed the expression of *GmPLA2s* in both *PLDα1KD* lines and WT seeds. The down-regulation of both *PLD* and *PLA* expression may explain the higher level of PCs in *PLDα1KD* seeds.

As LPC and LPE content decreased significantly in *PLDα1KD* than wild-type seeds by more than tenfold at stage 3, we examined the expression of genes involved in the LPC and PC cycle. LPC acyltransferases (*LPCAT*) catalyzes the synthesis of PC from LPC using a new fatty acyl-CoA (Fig. 10a). *AtLPCAT1* and 2 in Arabidopsis control the acyl editing process by acting as the main entry of unsaturated FAs into PC [16]. The *lpcat1/lpcat2* mutant showed decreased PUFA in seed TAG [16, 17]. Soybean genome contains two LPCATs, *Glyma.17G131500* (*GmLPCAT1*) and *Glyma.05G049500* (*GmLPCAT2*). Transcript levels of two genes increased to the highest levels at middle stages and then decreased at the later stage of seed development in both *PLDα1KD* lines under stress conditions (Fig. 10b, Additional file 1: Figure S15) [33]. Both *GmLPCAT* transcripts were 17% higher in *PLDα1KD* seeds than in wild-type seeds at later stages under stress conditions (Fig. 10b). Meanwhile, Both genes were up-regulated in both *PLDα1KD* lines and wild-type under stress conditions. The down-regulation of *GmPLA*s and up-regulation of *GmLPCATs* in *PLDα1KD* soybean seeds could lead to reduced contents of LPC and LPE, as compared with wild-type (Figs. 6, 7, 10).

### Higher germination rate of *PLDα1KD* seeds

**Fig. 11:**
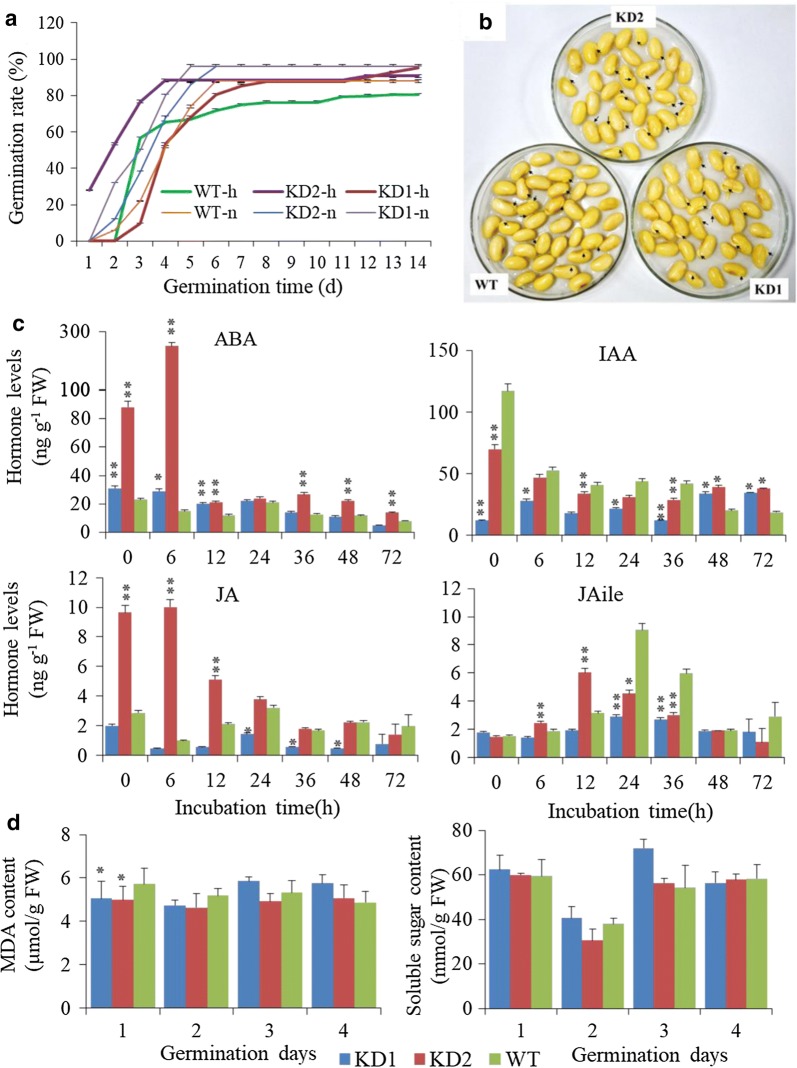
Germination rates and hormone levels of *PLDα1KD*- and wild-type-germinating seeds. **a** Germination rates of *PLDα1KD* and wild-type Jack (WT) seeds under normal conditions after maturation under high temperature (30–36 °C) and humidity (70–85%) environments. Values are mean ± SD (*n* = 30 in one replications). **b** Germination seeds of *PLDα1KD* and wild-type Jack (WT) soybean for 3 days. **c** Hormone levels in germinating seeds of *PLDα1KD* and wild-type Jack (WT). **d** The contents of malonaldehyde and soluble sugars. KD1: *PLDα1* knockdown line 1; KD2: *PLDα1* knockdown line 2; WT: wild-type Jack. All data are e mean ± SD (*n* = 3). * and **Denote significance at *P* < 0.05 and *P* < 0.01, respectively, compared with wild-type Jack (WT) based on Student’s *t* test

To address the effect of *PLDα1KD* on soybean seed vigor after harvested from high temperature and humidity conditions, we tested the seed vigor and germination rate of these knockdown lines and wild-type after stored at high temperature and humidity for three months (28 ± 3 °C in the dark and ~ 50% humidity). *PLDα1KD* seeds displayed higher germination rates, than the wild-type seeds under high temperature and humidity conditions. Under stress conditions, the germination rates of wild-type, *PLDα1KD*2 and *PLDα1KD1* seeds were 80%, 91%, and 95%, respectively, whereas they were 88%, 96%, and 96% under normal conditions (Fig. 11a, b). However, the germination rate of *PLDα1KD1* was lower than wild-type at an early stage but then caught up and became higher than wild-type at later stages. We further analyzed hormone levels in those germination seeds at different days after imbibitions. Higher ABA contents in *PLDα1KD* seeds than in wild-type seeds were detected, suggesting that *PLDα1KD* seeds had deeper seed dormancy than wild-type seeds and less nutrient consumption in *PLDα1KD* than in wild-type seeds during storage. Meanwhile, *PLDα1KD* seeds had initially a lower level of indoleacetic acid (IAA), but later a higher IAA level than that did wild-type seeds (Fig. 11c). Similarly, the seeds of two *PLDα1KD* lines showed difference in jasmonate (JA) and Ile-conjugated JA level from wild-type (Fig. 11c). *PLDα1KD* line 1 (KD1) seeds had lower total JAs and a lower germination rate, whereas *PLDα1KD* line 2 had higher total JAs and a higher germination rate than wild-type (Fig. 11c). The content of MDA was decreased in *PLDα1KD* seeds germinating for 1 day and had no significant difference within 2–4 days compared with wild-type seeds. There was also no significant difference in the content of soluble sugar in both *PLDα1KD* and wild-type germinating seeds (Fig. 11d).

## Discussion

**Fig. 12:**
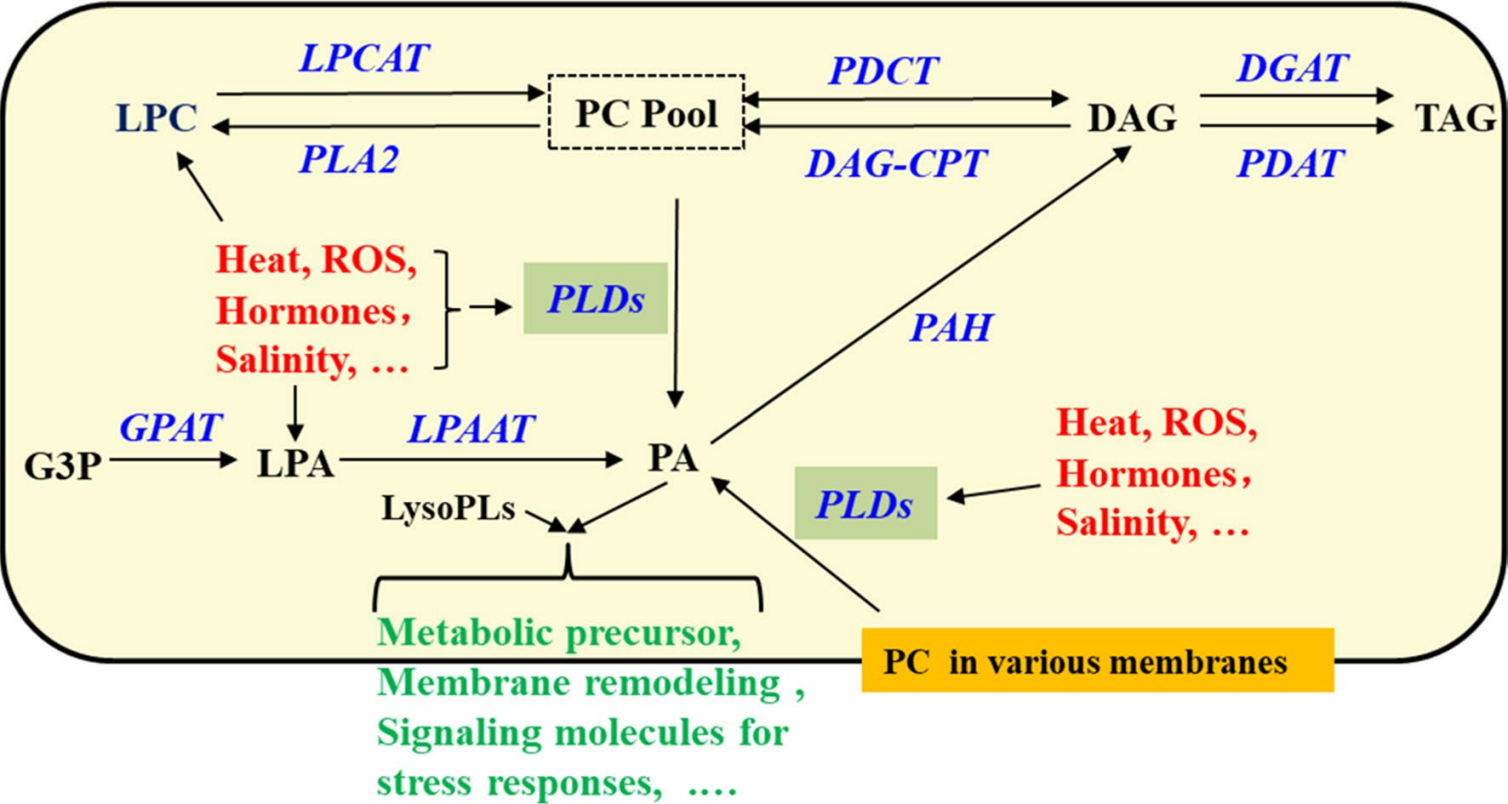
Schematic of the links between TAG and phospholipid metabolism involving *PLDα1. CCT* choline-phosphate cytidylyltransferase, *DAG* diacylglycerol, *DAG-CPT* diacylglycerol cholinephosphotransferase, *DGAT*, acyl-CoA: diacylglycerol acyltransferase, *G3P* glycerol-3-phosphate, *GPAT* glycerol-3-phosphate acyltransferase, *LPA* 2-lysophosphatidic acid, *LPAAT* 2-lysophosphatidic acid acyltransferase, *LPC* 2-lysophosphatidylcholine, *LPCAT* 2-lysophosphatidylcholine acyltransferase, *PA* phosphatidic acid, *PDAT* phospholipid:diacylglycerol acyltransferase, *PDCT* phosphatidylcholine:diacylglycerol cholinephosphotransferase, *PLA2* phospholipase A2, *PAH* phosphatidic acid phosphatase, *PLD* phospholipase D, *PC* phosphatidylcholine, *TAG* triacylglycerol

Seed development is a temperature-sensitive process more vulnerable than vegetative tissues to high temperature stress; high temperature and humidity conditions reduce lipid contents in soybean seeds, as compared with normal growth conditions [3]. However, *PLDα1KD* attenuated the reduction in lipid contents of soybean developing seeds compared with wild-type under the stress conditions, as well as under normal growth conditions. We showed higher total TAG content in the *PLDα1KD* transgenic lines at most developmental stages, with higher proportion of polyunsaturated fatty acid (PUFA) in TAG and PC as well. The higher levels of PCs are postulated as a consequence of down-regulation of *PLD*s and decreased transcripts of *PLA*s. The enhanced desaturation of PCs in *PLDα1KD* developing seeds is primarily attributable to increased *FAD2* and *FAD3* expression (Fig. 5a). The up-regulation of *FAD*s might further enhance acyl editing on PC or PE, which triggers an accelerated interconversion of PCs to DAGs in *PLDα1KD* to drive metabolic flux toward unsaturated TAG biosynthesis. Up-regulated *DAG:CPT*s and *PDCT*s, as well as down-regulated *CCT* in *PLDα1KD* developing seeds indicate an enhanced metabolic flux or cross-talk between the acyl-editing and Kennedy pathways in *PLDα1KD* seeds (Fig. 12). The *PLDα1* knockdown likely affected the responses of developing soybean seeds to high temperature and humidity conditions through modification of the levels of PAs and lysoPLs. These two signaling molecules accumulated to higher levels in developing wild-type seeds upon high temperature and humidity conditions, but significantly reduced in *PLDα1KD* developing seeds than wild-type. Levels of PAs and lysoPLs could be negatively related to seed viability and lipid stability, and lower levels of seed PAs and lysoPLs may have better seed viability and lipid stability in mature *PLDα1KD* soybean seeds.

### *PLDα1* knockdown promoted fatty acid unsaturation in both TAGs and phospholipids

There are increases in the total contents of both TAGs and unsaturation fatty acids in the developing seeds of *PLDα1KD* than wild-type cultivar under high temperature and humidity conditions. The desaturation of PCs is primarily attributable to FAD2 and FAD3 in the ER [48–50]. The increased transcripts of *FAD2s* and *FAD3s* in *PLDα1KD* seeds explain the increased unsaturated fatty acids in PCs and PEs, and TAG species, together with the decreased levels of PA, LPC, and LPE species in the *PLDα1KD* developing seeds. The difference in lipid contents between *PLDα1KD* and wild-type seeds under normal conditions was similar to that reported previously [9]. The high temperature and humidity decreased the contents of total lipids, especially fatty acid contents in seeds compared with these under normal conditions. However, our data showed that the differences in lipid contents between *GmPLDα1KD* and wild-type seeds under the stress conditions became much bigger than those under normal conditions. Meanwhile, the high temperature and humidity conditions affected the expression of genes involved in PLs and TAG synthesis pathways, which may eventually result in decreased lipid contents. Consistently, under these stress conditions, developing seeds of *GmPLDα1KD* displayed higher gene expression levels and total lipid contents than those of wild-type seeds, e.g., *GmFAD2*-*1A* and *GmFAD2*-*2A.* Mature *GmPLDα1KD* seeds also had better germination rates than did wild-type seeds. Therefore, suppression of *GmPLDα1* improved the expression of genes involved in PLs and TAG synthesis pathways under both high temperature and humidity stress and normal conditions.

### *PLDα1KD* enhanced the PDAT pathway and DAG-PC conversion toward TAG biosynthesis

It has been proposed that the DAG:CPT-catalyzed reaction provides an acyl editing mechanism for the production of polyunsaturated TAGs containing 18:2 and 18:3 through PCs [51]. The significant increases in unsaturation acyl chains of PCs in developing soybean seeds of *PLDα1KD* plants were attributable to the higher expression levels of *FAD*s. Since both PC and DAG play essential roles in acyl editing on glycerolipids, their interconversion is important for TAG synthesis [14, 28, 52]. The knockdown of *PLDα1* in the developing soybean seeds affected expression of all genes involved in the acyl editing in soybean developing seeds (Fig. 12). The up-regulation of both *PDCT*s and *DAG:CPT*s in consistent with an increased TAG contents in soybean developing seeds, since PCs are the major source of DAGs that further flux into TAGs by the action of PDATs [14]. Since PA–DAG–PC–DAG metabolic pathway primarily takes place in soybean developing seeds, PDCT thus becomes essential in determining the unsaturation of TAG. *PDCT* mutation results in a 40% decrease in polyunsaturated FAs in seed TAG without disrupting overall TAG levels [19]. PDAT’s substrate specificity on PC and DAG further enhanced the PC–DAG conversion and resulted in higher levels of unsaturated fatty acids in TAGs of developing seeds. The markedly increased PC/PE accumulation coincides with up-regulated *FAD2* and *3*, *PDCT* and *PDAT* genes suggest PLDα1 negatively affects acyl editing and bridges the phospholipid turnover and TAG biosynthesis.

PLA catalyzes PC or PE hydrolysis to generate free fatty acid and lysoPLs, such as LPC or LPE. PLAs and LPCATs can form a PC turnover cycle (Fig. 12). The lower transcripts of PLAs in *PLDα1KD* are consistent with higher levels of PCs and lower levels of LPC and LPE in *PLDα1KD* developing seeds, which is also consistent with previous report in Arabidopsis *PLDα1KD* plants [53]. The down-regulation of *PLDα1* and *PLA* genes may together lower PA, LPC and LPE levels [28]. In *pah1pah2* mutant, PC contents dramatically increased and TAG content decreased due to the increased PA contents through up-regulation of *CCT1* [54]. Our results suggest that PLA–LPCAT, PLD, and PDCT–DAG:CTP play important roles in acyl editing. *PLDα1KD* promotes the PDCT and DAG:CPT-catalyzed conversion between PCs and DAGs, through which extensive acyl desaturation on PCs by FADs is eventually reflected by the increased TAG unsaturation. Thus, the developing *PLDα1KD* soybean seeds have higher levels of di18:2 than these non-transgenic lines.

### Effects of high temperature and humidity on soybean seed development

While high temperature and humidity can significantly affect membrane compositions, altering membrane physiological properties and functions, how the stress affects phospholipid and TAG metabolism in developing seeds is largely unknown [5, 9]. High temperature stress causes significant increases in ROS-scavenging, lipid desaturating, oxidizing, and acylating genes, and in 18:3-TAG contents in wheat, while fatty acyl chain unsaturation in polar phospholipids decrease [5]. ROS plays various roles in regulating cell growth, development, and cell survival under stress. Generally, moderate levels of ROS may function as signals to promote plant growth and survival, whereas a large increase of ROS can induce plant cell death. Under physiologic conditions, the balance between generation and elimination of ROS maintains the proper function of redox-sensitive signaling proteins. However, under adversary environments, such as extreme heat, light, and cold conditions, plants produce more ROS or xenobiotics than normal levels, and activate ROS-scavenging enzymes and xenobiotics-detoxification enzyme GST to reduce ROS damage to plants.

### Possible mechanisms for the improvement of soybean seeds *by knockdown of GmPLDα1*

*PLDα1* and its product PA and PLA and its product LysPLs, have been reported in other plants to function in plant stress response to salt and drought stresses, oxidative stresses, and hormones [10, 27, 55]. Our study here indicates that high temperature and humidity environments significantly affect phospholipid and TAG metabolism in developing soybean seeds, and increase the contents of PAs and lysoPLs in wild-type seed, but the contents of PAs and lysoPLs were significantly reduced in *PLDα1KD* mutant seeds (Figs. 6, 7). The anti-deterioration effects of *PLDα1KD* mutation on developing soybean seeds under high temperature and humidity conditions in our study were similar to the better oil storability and seed viability of these naturally or artificially aged Arabidopsis and soybean *PLDα1*-antisense mutant seeds [7, 8]. Those results suggest that the lowered PAs and lysoPLs in *PLDα1KD* or knockout mutant seeds might be the major causes. The higher PA and lysoPL contents may increase membrane permeability during seed development and storage [7, 8, 10]. The variations in lipid contents and gene expression in two independent *PLDα1KD* lines may result from the variations of levels of PAs and lysoPLs because of different degrees of *PLD* suppression. It has been observed that different knockdown lines of *AtPLDα1* often showed variations in phenotypes [7, 10, 27]. Besides its metabolic function, PLDα1 is also involved in plant responses to salinity, drought, cold, and heat stresses [27] (Fig. 12). The distinct responses of *ROS*-scavenging genes in *PLDα1KD* and wild-type cultivars may also be associated to *GmPLDα1/PLA* and PA/lysPLs that could be critically involved in soybean adaptive response to high temperature and humidity [27, 34, 35, 55]. We posit that PA and lysoPLs generated by PLDs and PLAs under high temperature and humidity conditions could be the keys for the detrimental effects of high temperature and humidity [7–9]. *PLDα1KD* substantially decreases stress-induced production of PA and lysoPLs and thereby alleviates the negative impacts by these adverse conditions on developing soybean seeds.

## Conclusions

Genetically modified *PLDα1KD* soybean seeds have improved oil content, seed vigor, and resistance against aging and deterioration under high temperature and humility environments. The mechanism behind the phenomena was explored by examination of relevant metabolites and transcripts of lipid metabolic genes in WT and *PLDα1KD* soybean developing seeds grown under both stress and normal conditions. The higher TAG content with higher proportion of unsaturation degree in *PLDα1KD* than wild-type at most developmental stages are attributable to the lower expression levels of *PLDs*, *PLA2s*, *CCTs*, and *DGATs* and higher expression levels of *FADs*, *LPCAT*, *PDAT*, *PAH*, *PDCT*, and *DAG:CPT* in *PLDα1KD* soybean. Higher levels of ROS-scavenging and xenobiotics-detoxifying genes (*GST*, *SOD*, *CAT*, *POD*, *STI*, and *APX*) in *PLDα1KD* soybean developing seeds and lower ROS level in germinated *PLDα1KD* seeds than those in wild-type seeds may explain their tolerance against high temperature and humility stress in preventing pre-harvest seed deterioration. This study presents a valuable model illustrating the role of *PLD* in TAG synthesis and provides novel insights into the mechanistic details of lipid metabolic pathway changes upon the knockdown of *PLDs* during seed development under stress. Despite of the biotechnological application potential in genetic improvement of soybean production under stress, the field trials may be required to validate the advantages for quality soybean production, especially under frequently occurred heatwaves.

## Materials and methods

### Creation of *PLDα1KD* transgenic soybean

The forward and reverse sequences of 1151 bp from the conserved *GmPLDα1* cDNA in Williams 82 were used for making RNAi constructs, which were different from the forward 820 bp and reverse 1300 bp from conserved *GmPLDα1* sequence derived from cloning partial sequence in Fayette used previously [9]. The RNAi cassette was assembled into the *Not*I site of the soybean expression vector pBetaConSoyHyg, flanked on its 5′ end by the seed-specific promoter for the soybean α’ subunit of β-conglycinin gene [56] and on its 3′ end by the 3′UTR of the phaseolin gene [57]. The vector backbone, which was derived from pBluescript SK-(Stratagene), was engineered with a hygromycin B phosphotransferase gene [58] under control of the potato ubiquitin-3 promoter [59] for selection of transformed soybean embryos. Additional file 1: Figure S1 illustrates the cloning strategy and details of the organization of gene cassettes generated in the vector pBetaConSoyHyg for soybean transformation. The construct was introduced into soybean (*Glycine max* cv. Jack) somatic embryos using biolistic transformation as described [60]. Embryos were maintained after bombardment in SHaM media [61] with hygromycin selection [60]. Mature somatic embryos obtained following hygromycin selection were screened by immunoblotting analysis for PLDα1 levels. Embryos, representing independent transgenic events, that showed reductions in PLDα1 levels, were desiccated and used for regeneration of transgenic plants as described [60]. Regenerated plants were advanced to homozygosity (> *T*_3_ generation) for use in the described studies. One hundred forty-three putative tissues were recovered after selection. Ten transgenic soybean events were recovered and fully grown in the greenhouse. Using enzyme activity, PCR and western blotting, two successfully repressed transgenic soybean events (#1020 and #1048) were selected from ten transgenic events for analysis.

### Plant growth conditions and treatments

Wild-type Jack and transgenic soybean *PLDα1KD* lines were grown in a greenhouse with a normal (26 ± 3 °C/18 h day and 23 ± 3 °C/6 h night photoperiod, 45–65% humidity) or high temperature and high humidity (36 ± 3 °C/18 h day and 30 ± 3 °C/6 h night photoperiod, 70–85% humidity) conditions which were controlled by air condition and humidifier machine. The developing seeds were harvested at approximately 50 days after fertilization (DAF). The varieties for transcriptomic analyses at different developmental stages of soybean seeds were Williams 82 and Hokkaido Black 25- and 50-day seeds were collected and put into liquid nitrogen immediately after separating from plants. To test the suppression of *PLDα1* in transgenic soybean seeds, qRT-PCR, western blotting with PLDα1 antibody, and PLDα1 activity assays were performed using developing staged 3 seeds of T3 and T5 lines. The presence of the transgene in T5 line seeds was confirmed by PCR. Monitoring soybean PLD proteins in the soybean extract used antibodies against *Arabidopsis thaliana* PLDα1.

### Analysis of seed total fatty acids

Total fatty acids were extracted three times and analyzed with GC using triheptadecanoylglycerol as an internal standard using a method described previously [44, 62]. Analyses were carried out on an Agilent Technologies (USA) 7890A Network gas chromatograph, equipped with a flame-ionization detector (FID) and a split/splitless injector. Polyethylene glycerol:Agilent 19091 N-133 column (30 m × 0.25 mm i.d. 0.25 m film thickness) was used [44]. An HP ChemStation (Hewlett-Packard, Palo Alto, CA, USA) was used for instrument control and data analysis.

### Analysis of seed TAGs

Seed TAG and its fatty acid compositions were analyzed as described previously [44]. Briefly, total seed lipids from developing seeds were separated into TAG, DAG, and free fatty acids on a TLC plate, and TAG spots were visualized with iodine vapor and identified by their migration. The spots of TAG were scraped off TLC plates for determination of fatty acid contents and compositions by GC as described above.

### Immunoblotting and PLD activity assays

Proteins were extracted from immature or mature soybean seeds with extraction buffer containing 50 mm Tris–HCl (pH 7.5), 10 mm KCl, 1 mm EDTA, 0.5 mm phenyl methylsulfonyl fluoride and 2 mm DTT at 4 °C [63]. Proteins were incubated at boiling water bath for 10 min with a SDS-PAGE loading buffer containing 100 μL of 50 mm Tris–HCl (pH 6.8), 10 mm DTT, 2% SDS, 0.01% bromophenol blue and 10% glycerol. Twenty micrograms of denatured proteins were fractionated by 10% SDS-PAGE and transferred to a membrane. The membrane was blotted with Arabidopsis PLDα1 antibodies as described by [63]. Aliquots of 20 μg of native protein were used for the PLD enzyme assay as described previously [63].

### Phospholipid analysis

Phospholipid profiling was performed on soybean developing seeds with ESI-MS/MS as described previously [64]. In brief, five replicates of developing soybean seed samples were smashed in liquid nitrogen and transferred immediately to 3 mL of 85 °C hot isopropanol containing 0.01% butylated hydroxytoluene (BHT) for 15 min. Then, adding 1.5 mL of chloroform and 1.5 mL of methanol for mixing well and extraction on shaker for 30 min, and then 1.8 mL of H_2_O were added to each sample separately. After mixing and centrifugation, lower layer solvent containing lipids was transferred to a new tube. Repeat the extraction with 3 mL of chloroform/methanol (2:1) containing 0.01% BHT for five times for 10 min each time. The combined solvent extracts were washed with 1 mL of 1 M KCl and 1 mL of sterile ddH_2_O, successively, and then concentrated with nitrogen gas. The total extracts were transferred to 2.0 mL vial with Teflon-lined screw cap and dried completely. The methanol solved lipids were detected followed the published protocol [64]. Five duplicates for each genotype or treatments were analyzed.

### Quantitative reverse transcriptase-PCR (qRT-PCR) for gene expression analysis

qRT-PCR was done as previously described [65]. Briefly, total RNA was isolated from seeds with RNA isolation kit (Bioteke Corporation). The first strand of cDNA was synthesized according to the supplier’s instructions (M-MLV First Strand kit, Life Technologies, Invitrogen, USA) and real-time RT-PCR was executed with primers listed as Additional file 2: Table S1 using the BioRad iQ5 (Bio-Rad, USA) with an SYBR green MIX (Premix system, NEWBIO INDUSTRY). Soybean *ACTIN1* (Glyma.15g034000) was used as the internal positive control.

### Seed germination assay

After storing seeds for 3 months in high temperature and humidity conditions, seeds of *PLDα1KD*s and wild-type control harvested at the same time in same size were selected for assay. Seeds were sterilized with chloride gas and washed in distilled water three times before germinating in plates containing wet sterile filter papers. Seeds were germinated in an incubator at 25 °C in 16 h day/8 h night. Seed germination was scored when the radical was elongated about 0.5 cm.

### Analysis of seed hormones

Seed samples collected at different time during germination were immediately frozen in liquid nitrogen and stored at − 80 °C. Hormones were measured according to the method described previously, with modifications [66]. Briefly, the liquid nitrogen-frozen samples were lyophilized, and 0.1 g of samples was extracted with 750 μL methanol:ddH_2_O:acetic acid (80:19:1, v/v/v) for three times in the dark and 4 °C. The supernatants were combined and dried under stream of nitrogen. Prior to UPLC/ESI-MS/MS analysis, the extracts were suspended in 250 μL methanol and sonicated for 10 min, and spun at 12,000 g for 10 min, and the supernatant was transferred into UPLC vials. Chromatographic separation was performed using the Agilent LC-20AD system (Agilent, Santa Clara, CA, USA) equipped with AB Sciex QTRAP^®^ 5500 detector and a Zorbax × 300 SB-C18 (4.6 mm × 150 mm × 5 μm) column (Agilent, Palo Alto, CA, USA), as described previously [66].

### Bioinformatics’ analyses

Soybean proteins involved in lipid metabolism and their expression data were retrieved from Phytozome (http://phytozome.jgi.doe.gov/pz). For analyses, these genes were compared with homologues from *Arabidopsis thaliana* and other plant species retrieved from NCBI (http://www.ncbi.nlm.nih.gov/BLAST/). Phylogeny trees were constructed using the neighbor-joining tree method with MEGA6. The significance level of the neighbor-joining analysis was examined by bootstrap testing with 1000 repeats.

## Additional files

**Additional file 1: Figure S1.** Schematic procedure for construction of soybean GmPLDα1RNAi vector for plant transformation. **Figure S2.** Phylogenetic analysis of *PLD* genes from the soybean genome and their expression patterns. **Figure S3.** Mechanism of plant resistant to stress and gene expression of GmPLDαs and stress-related genes under high temperature and humidity condition in comparison to under normal conditions. **Figure S4.** Analyses of lipids in PLDα1KD and wild-type developing seeds under different growth conditions. **Figure S5.** Phylogenetic analysis of *FAD* genes from the soybean genome and their expression patterns. **Figure S6.** Phylogenic analysis of Acyl-CoA:lysophosphatic acid acyltransferase (*LPAAT*) genes and their expression profiles. **Figure S7.** Phylogenic analysis of phosphatidic acid hydrolase (*PAH*) genes and their expression profiles. **Figure S8.** Phylogenic analysis of acyl-CoA:diacylglycerol acyltransferase (*DGAT*) genes and their expression profiles. **Figure S9.** Phylogenic analysis of phospholipid:diacylglycerol acyltransferase (*PDAT*) genes and their expression profiles. **Figure S10.** Phylogenic analysis of choline/ethylamine kinase (*CEK*) genes and their expression profiles. **Figure S11.** Phylogenic analysis of CTP: phosphocholine cytidylyltransferase (*CCT*) genes and the expression profiles. **Figure S12.** Phylogenic analysis of diacylglycerol:cholinephosphotransferase (*DAG-CPT* or *AAAT*) genes and their expression profiles. **Figure S13.** Phylogenic analysis of phosphatidylcholine:diacylglycerol cholinephosphotransferase (*PDCT*) genes and their expression profiles. **Figure S14.** Phylogenetic analysis of *PLA* genes from the soybean genome and their expression patterns. **Figure S15.** Phylogenic analysis of 2-lysophosphatidylcholine acyltransferase (*LPCAT*) genes and their expression profiles.

**Additional file 2: Table S1.** The Quantitative RT-PCR primers used in this study. **Table S2.** *PLD* genes in soybean genome. **Table S3.** Expression pattern in different tissue. **Table S4.** Relative expression of *GmPLDɑ*s in different developing seeds after fertilization. **Data S1.** Protein sequences for phylogenic tree construction.
