# Supplementary material for: PLDα1-knockdown soybean seeds display higher unsaturated glycerolipid contents and seed vigor in high temperature and humidity environments
Source: Biotechnol Biofuels. 2019 Jan 4;12:9. doi: 10.1186/s13068-018-1340-4 (PMC6319013; doi:10.1186/s13068-018-1340-4)
Supplement: Supplementary file 1 — Additional file 1: Figure S1. Schematic procedure for construction of soybean GmPLDα1RNAi vector for plant transformation. Figure S2. Phylogenetic analysis of PLD genes from the soybean genome and their expression patterns. Figure S3. Mechanism of plant resistant to stress and gene expression of GmPLDαs and stress-related genes under high temperature and humidity condition in comparison to under normal conditions. Figure S4. Analyses of lipids in PLDα1KD and wild-type developing seeds under different growth conditions. Figure S5. Phylogenetic analysis of FAD genes from the soybean genome and their expression patterns. Figure S6. Phylogenic analysis of Acyl-CoA:lysophosphatic acid acyltransferase (LPAAT) genes and their expression profiles. Figure S7. Phylogenic analysis of phosphatidic acid hydrolase (PAH) genes and their expression profiles. Figure S8. Phylogenic analysis of acyl-CoA:diacylglycerol acyltransferase (DGAT) genes and their expression profiles. Figure S9. Phylogenic analysis of phospholipid:diacylglycerol acyltransferase (PDAT) genes and their expression profiles. Figure S10. Phylogenic analysis of choline/ethylamine kinase (CEK) genes and their expression profiles. Figure S11. Phylogenic analysis of CTP: phosphocholine cytidylyltransferase (CCT) genes and the expression profiles. Figure S12. Phylogenic analysis of diacylglycerol:cholinephosphotransferase (DAG-CPT or AAAT) genes and their expression profiles. Figure S13. Phylogenic analysis of phosphatidylcholine:diacylglycerol cholinephosphotransferase (PDCT) genes and their expression profiles. Figure S14. Phylogenetic analysis of PLA genes from the soybean genome and their expression patterns. Figure S15. Phylogenic analysis of 2-lysophosphatidylcholine acyltransferase (LPCAT) genes and their expression profiles. [file 13068_2018_1340_MOESM1_ESM.ppt]

## Slide 1
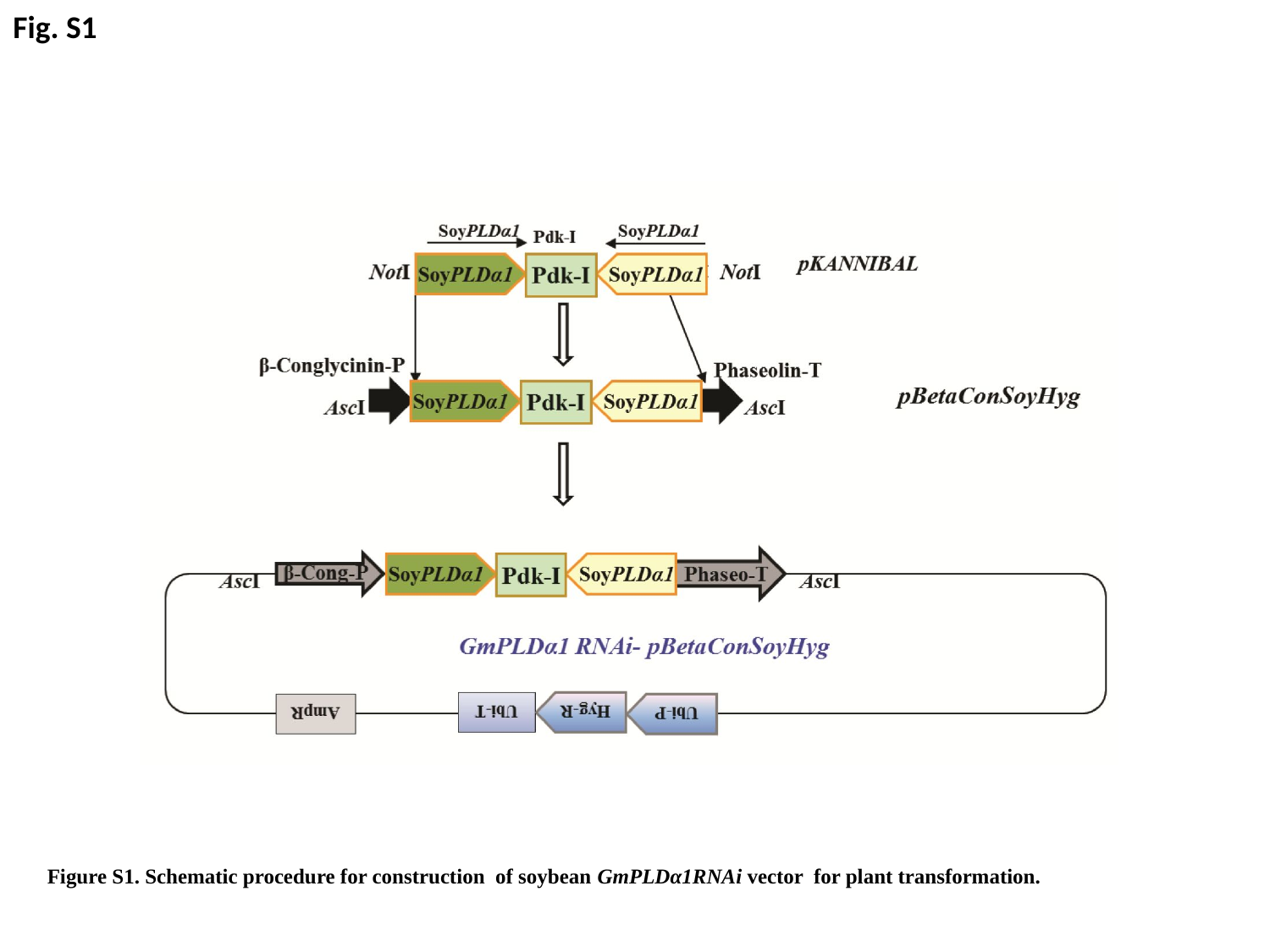

Fig. S1
Figure S1. Schematic procedure for construction of soybean GmPLDα1RNAi vector for plant transformation.

## Slide 2
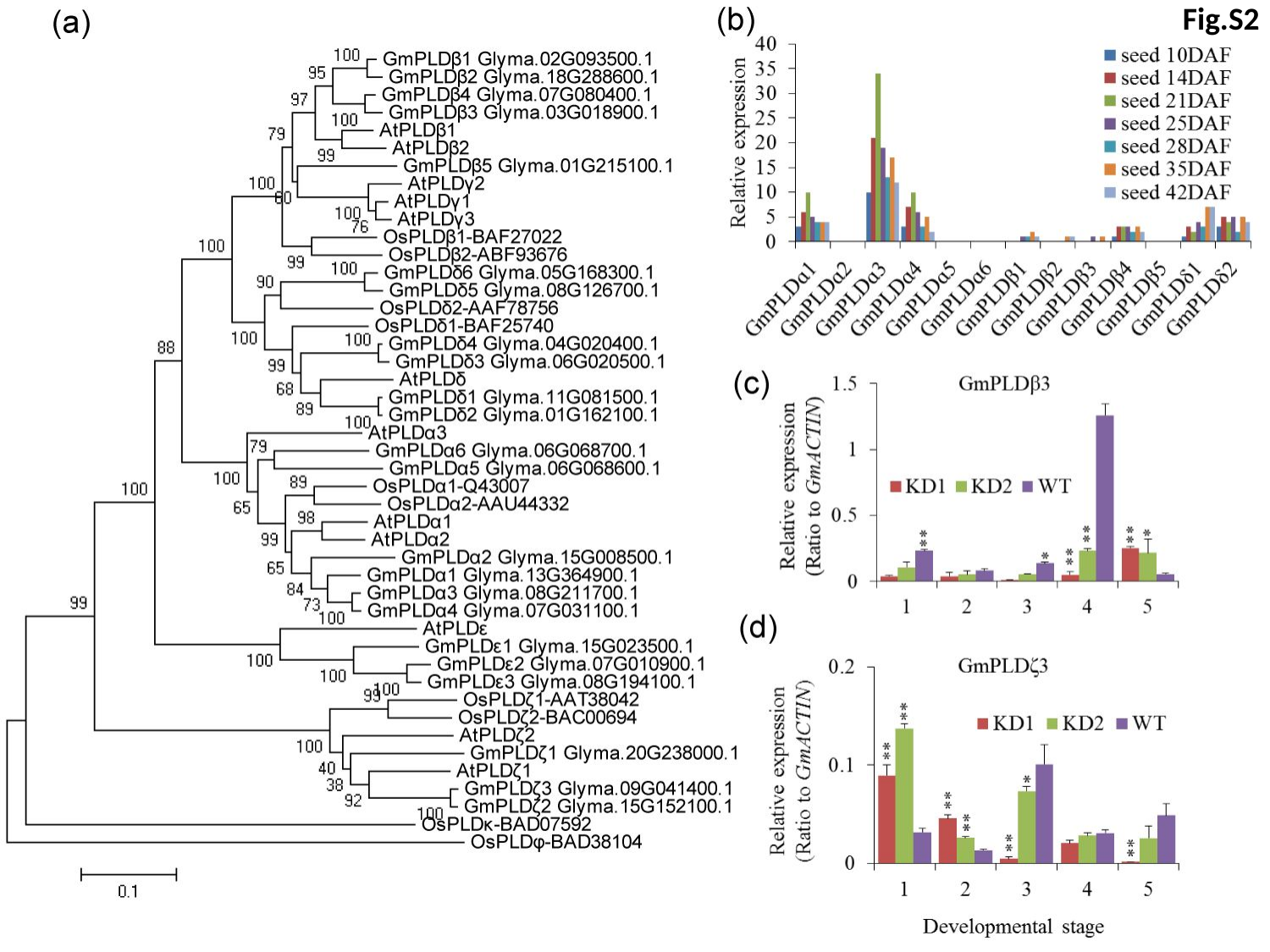

Fig.S2

## Slide 3
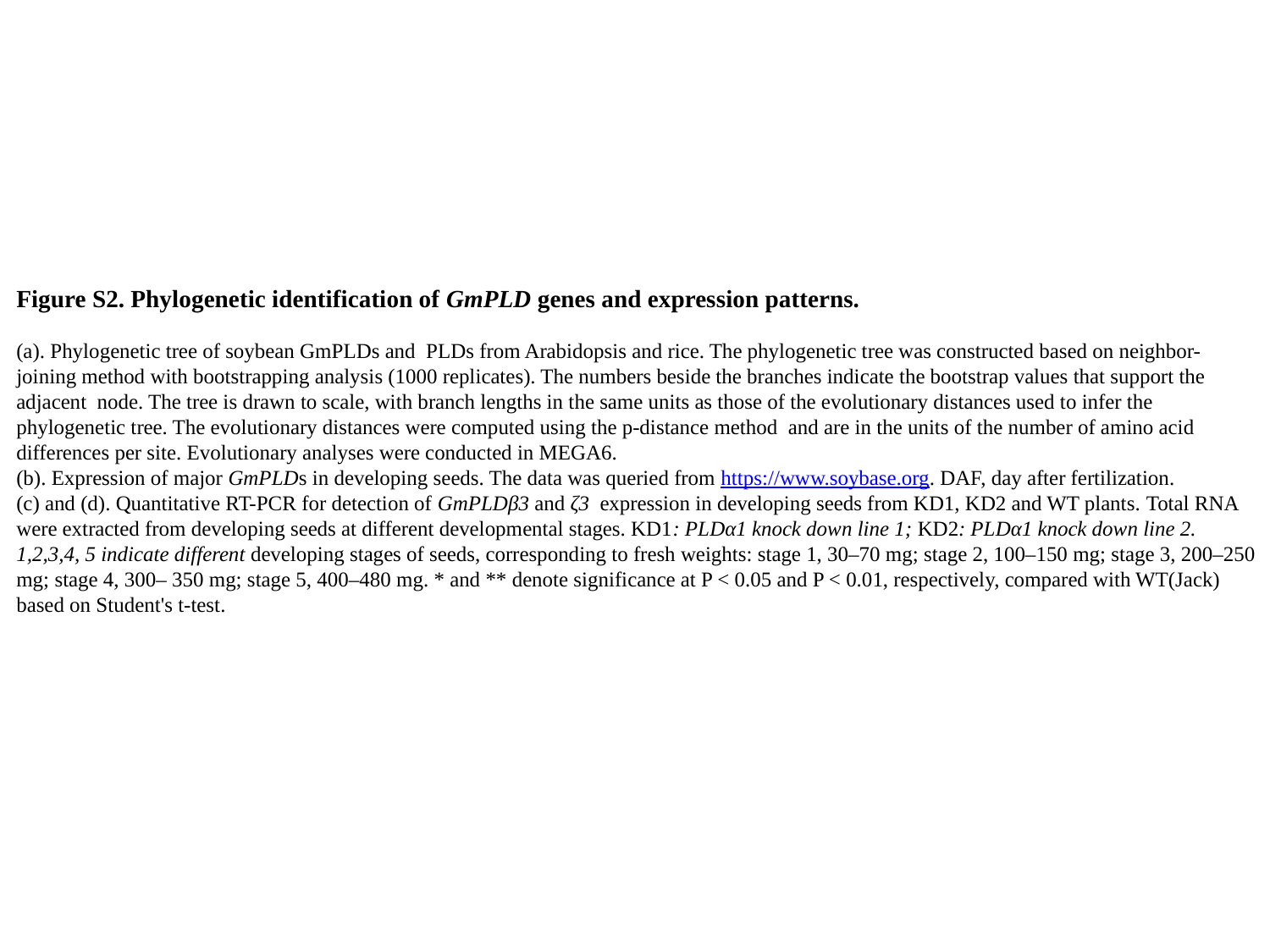

Figure S2. Phylogenetic identification of GmPLD genes and expression patterns.
(a). Phylogenetic tree of soybean GmPLDs and PLDs from Arabidopsis and rice. The phylogenetic tree was constructed based on neighbor-joining method with bootstrapping analysis (1000 replicates). The numbers beside the branches indicate the bootstrap values that support the adjacent node. The tree is drawn to scale, with branch lengths in the same units as those of the evolutionary distances used to infer the phylogenetic tree. The evolutionary distances were computed using the p-distance method and are in the units of the number of amino acid differences per site. Evolutionary analyses were conducted in MEGA6.
(b). Expression of major GmPLDs in developing seeds. The data was queried from https://www.soybase.org. DAF, day after fertilization.
(c) and (d). Quantitative RT-PCR for detection of GmPLDβ3 and ζ3 expression in developing seeds from KD1, KD2 and WT plants. Total RNA were extracted from developing seeds at different developmental stages. KD1: PLDα1 knock down line 1; KD2: PLDα1 knock down line 2. 1,2,3,4, 5 indicate different developing stages of seeds, corresponding to fresh weights: stage 1, 30–70 mg; stage 2, 100–150 mg; stage 3, 200–250 mg; stage 4, 300– 350 mg; stage 5, 400–480 mg. * and ** denote significance at P < 0.05 and P < 0.01, respectively, compared with WT(Jack) based on Student's t-test.

## Slide 4
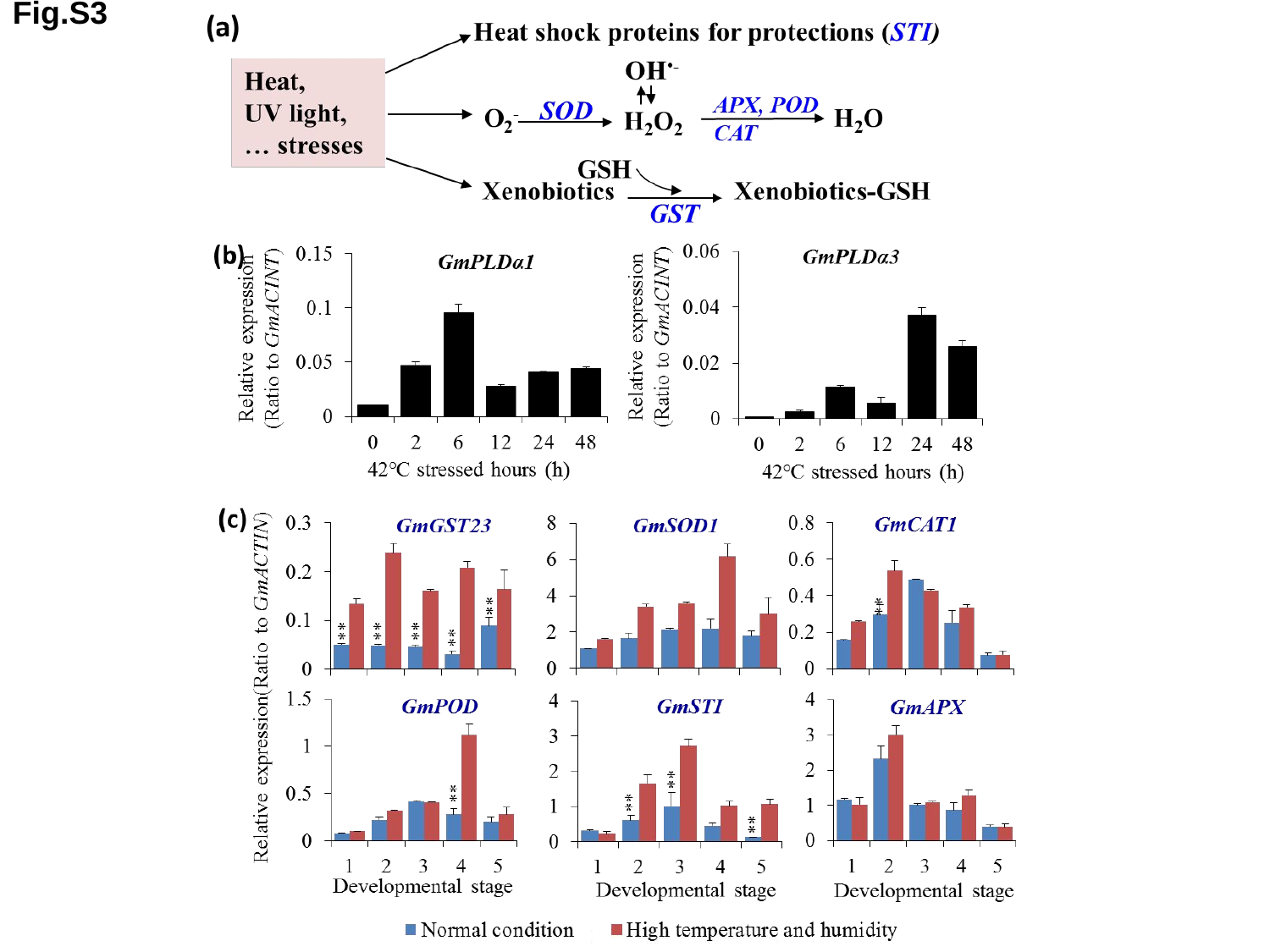

Fig.S3

## Slide 5
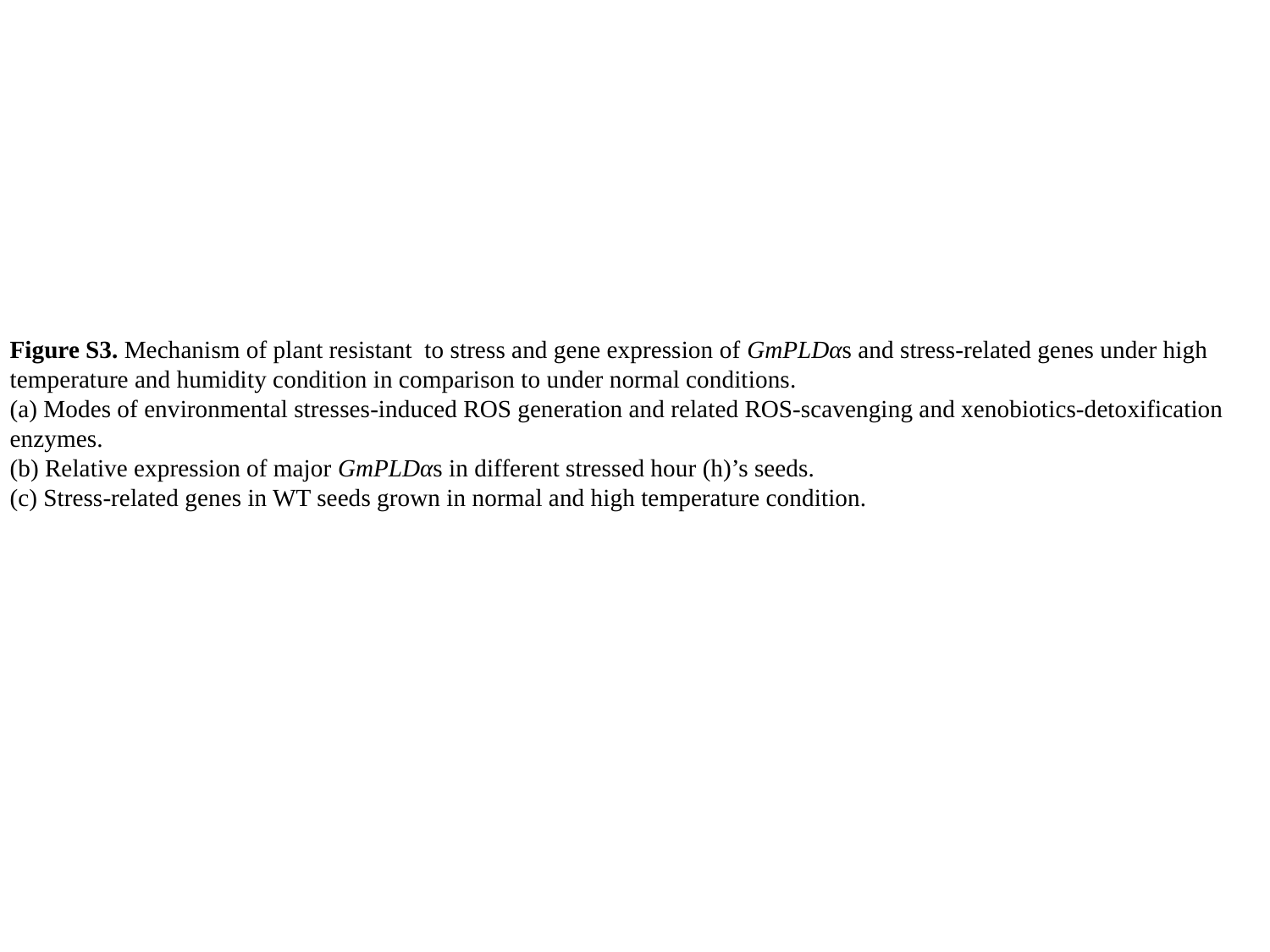

Figure S3. Mechanism of plant resistant to stress and gene expression of GmPLDαs and stress-related genes under high temperature and humidity condition in comparison to under normal conditions.
(a) Modes of environmental stresses-induced ROS generation and related ROS-scavenging and xenobiotics-detoxification enzymes.
(b) Relative expression of major GmPLDαs in different stressed hour (h)’s seeds.
(c) Stress-related genes in WT seeds grown in normal and high temperature condition.

## Slide 6
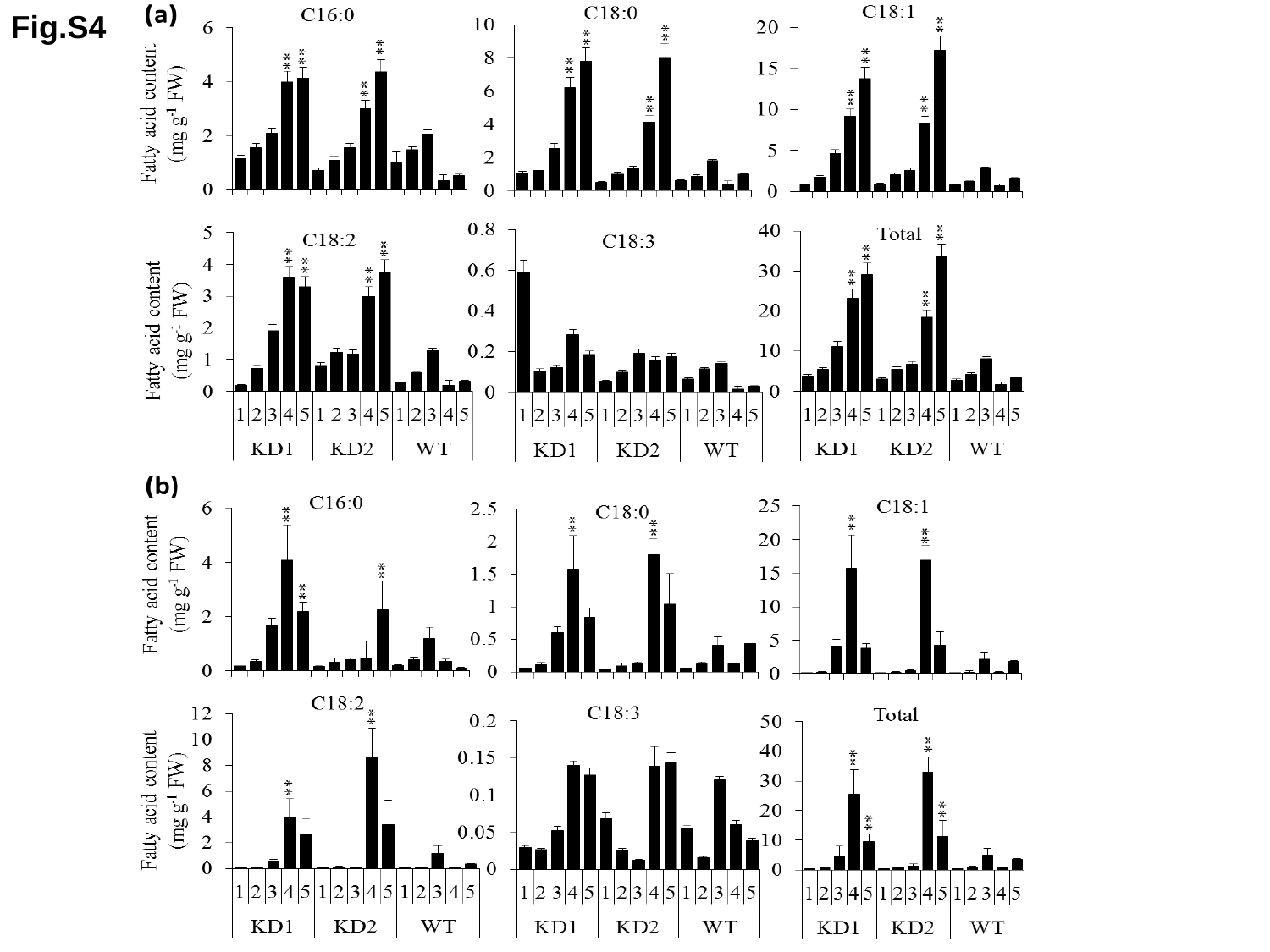

Fig.S4

## Slide 7
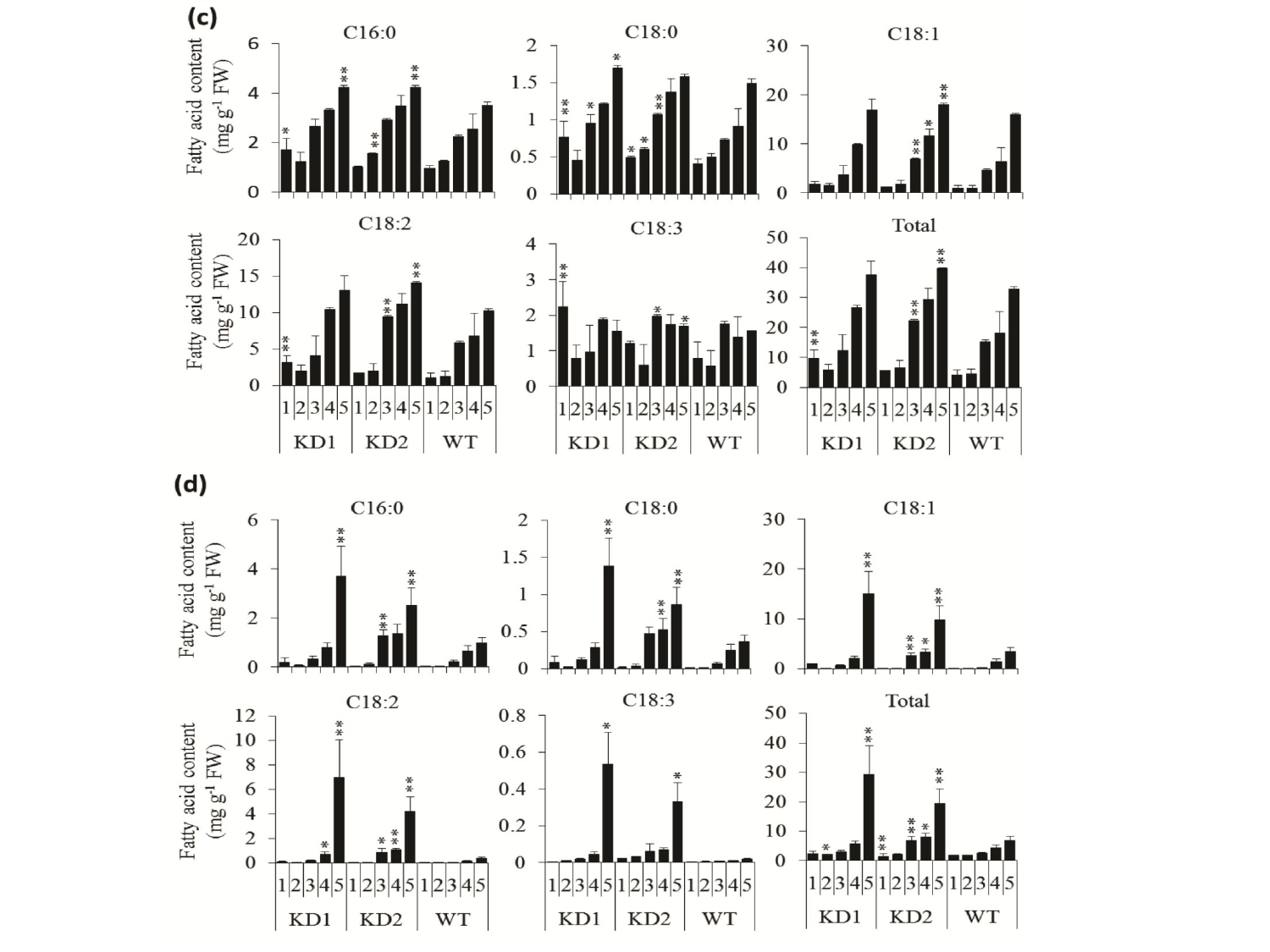

## Slide 8
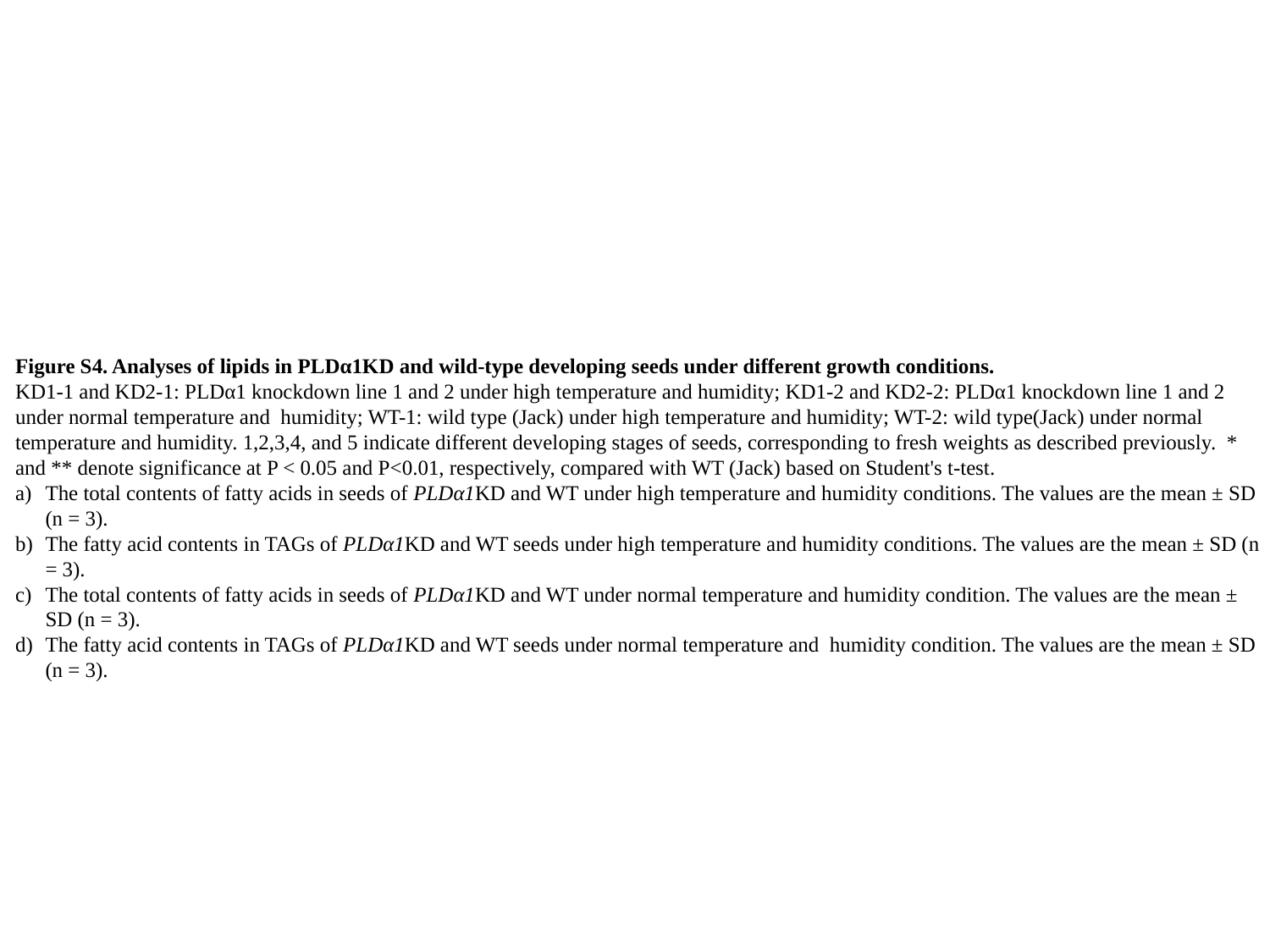

Figure S4. Analyses of lipids in PLDα1KD and wild-type developing seeds under different growth conditions.
KD1-1 and KD2-1: PLDα1 knockdown line 1 and 2 under high temperature and humidity; KD1-2 and KD2-2: PLDα1 knockdown line 1 and 2 under normal temperature and humidity; WT-1: wild type (Jack) under high temperature and humidity; WT-2: wild type(Jack) under normal temperature and humidity. 1,2,3,4, and 5 indicate different developing stages of seeds, corresponding to fresh weights as described previously. * and ** denote significance at P < 0.05 and P<0.01, respectively, compared with WT (Jack) based on Student's t-test.
The total contents of fatty acids in seeds of PLDα1KD and WT under high temperature and humidity conditions. The values are the mean ± SD (n = 3).
The fatty acid contents in TAGs of PLDα1KD and WT seeds under high temperature and humidity conditions. The values are the mean ± SD (n = 3).
The total contents of fatty acids in seeds of PLDα1KD and WT under normal temperature and humidity condition. The values are the mean ± SD (n = 3).
The fatty acid contents in TAGs of PLDα1KD and WT seeds under normal temperature and humidity condition. The values are the mean ± SD (n = 3).

## Slide 9
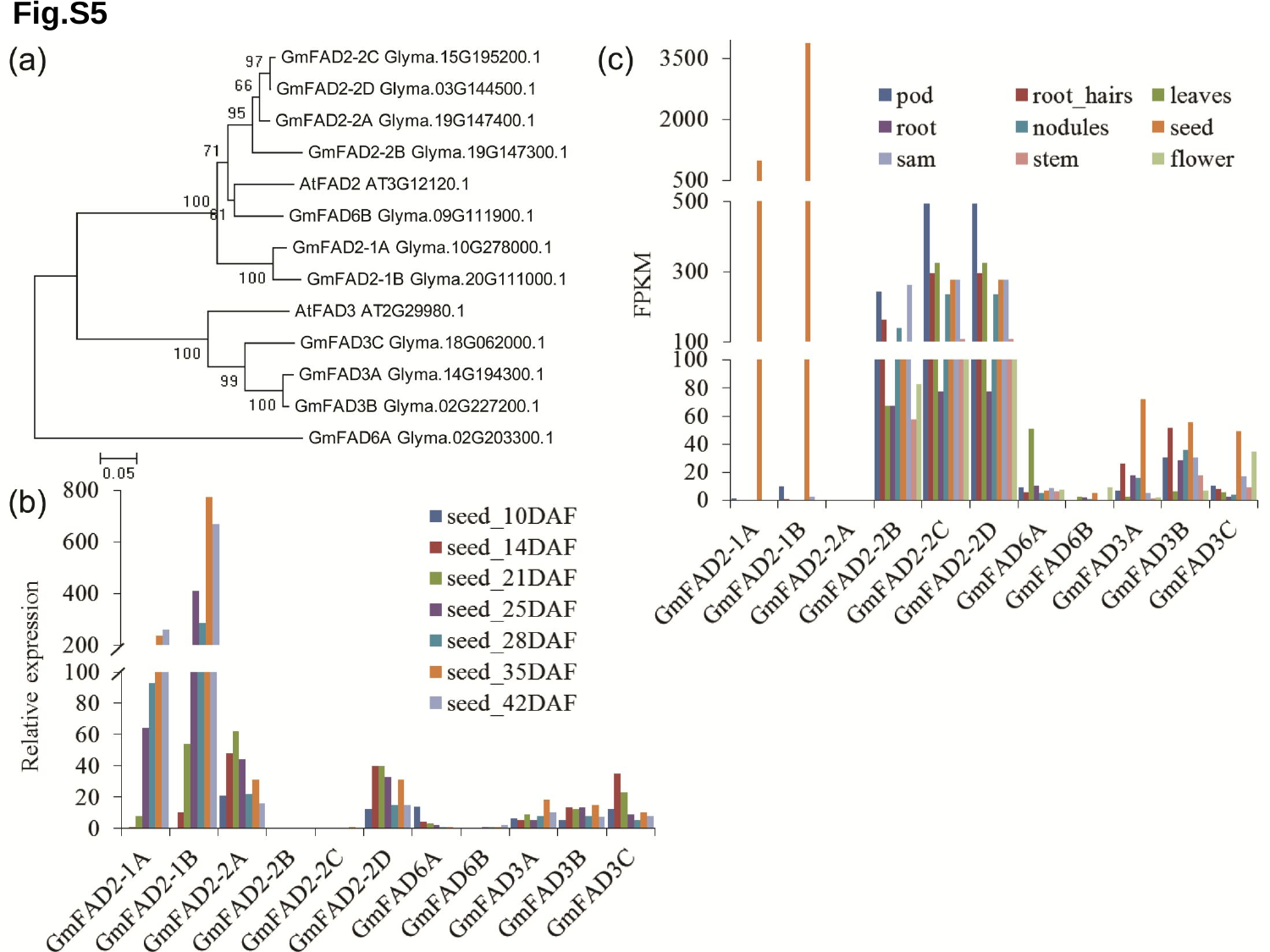

Fig.S5

## Slide 10
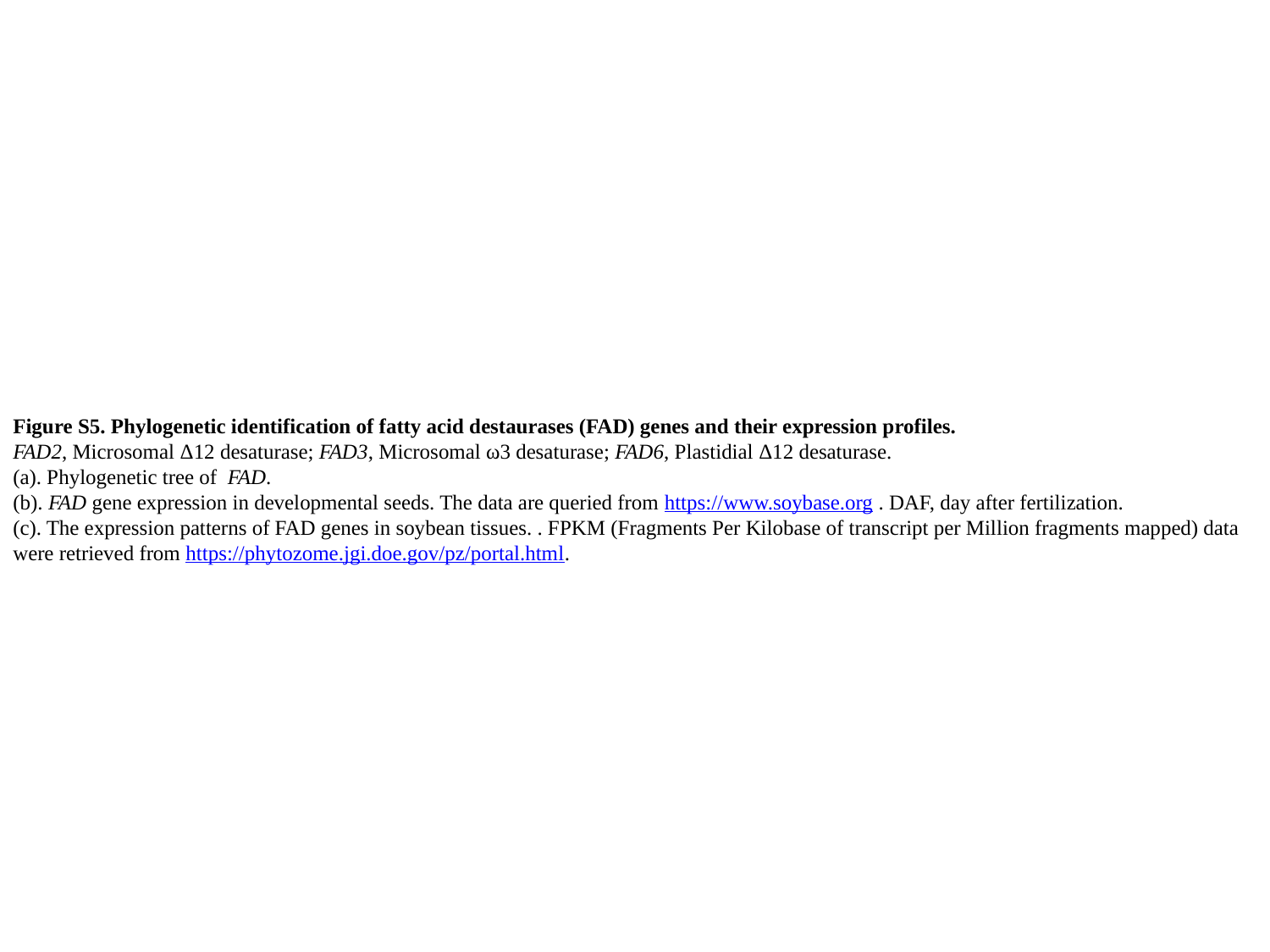

Figure S5. Phylogenetic identification of fatty acid destaurases (FAD) genes and their expression profiles.
FAD2, Microsomal Δ12 desaturase; FAD3, Microsomal ω3 desaturase; FAD6, Plastidial Δ12 desaturase.
(a). Phylogenetic tree of FAD.
(b). FAD gene expression in developmental seeds. The data are queried from https://www.soybase.org . DAF, day after fertilization.
(c). The expression patterns of FAD genes in soybean tissues. . FPKM (Fragments Per Kilobase of transcript per Million fragments mapped) data were retrieved from https://phytozome.jgi.doe.gov/pz/portal.html.

## Slide 11
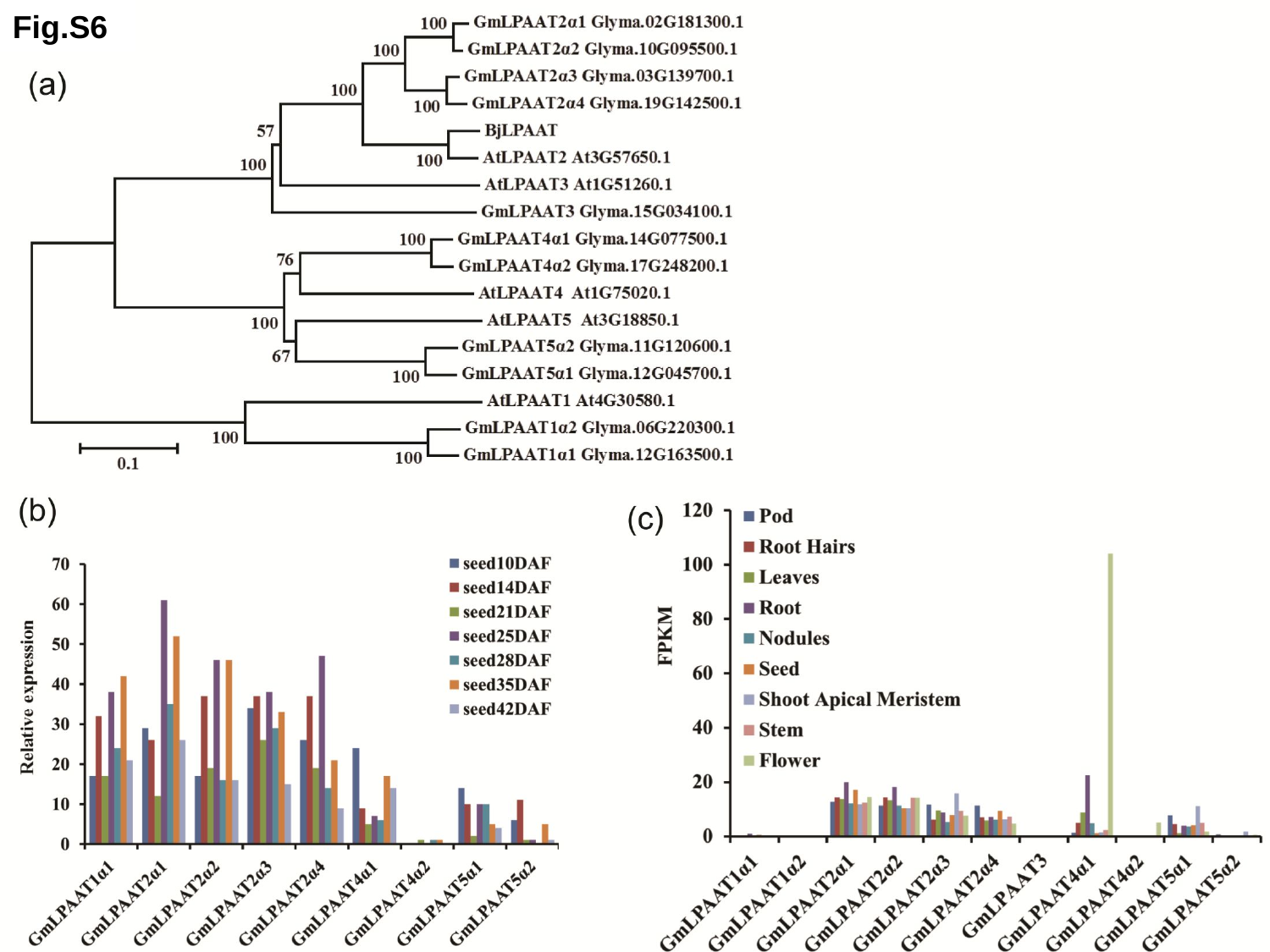

Fig.S6

## Slide 12
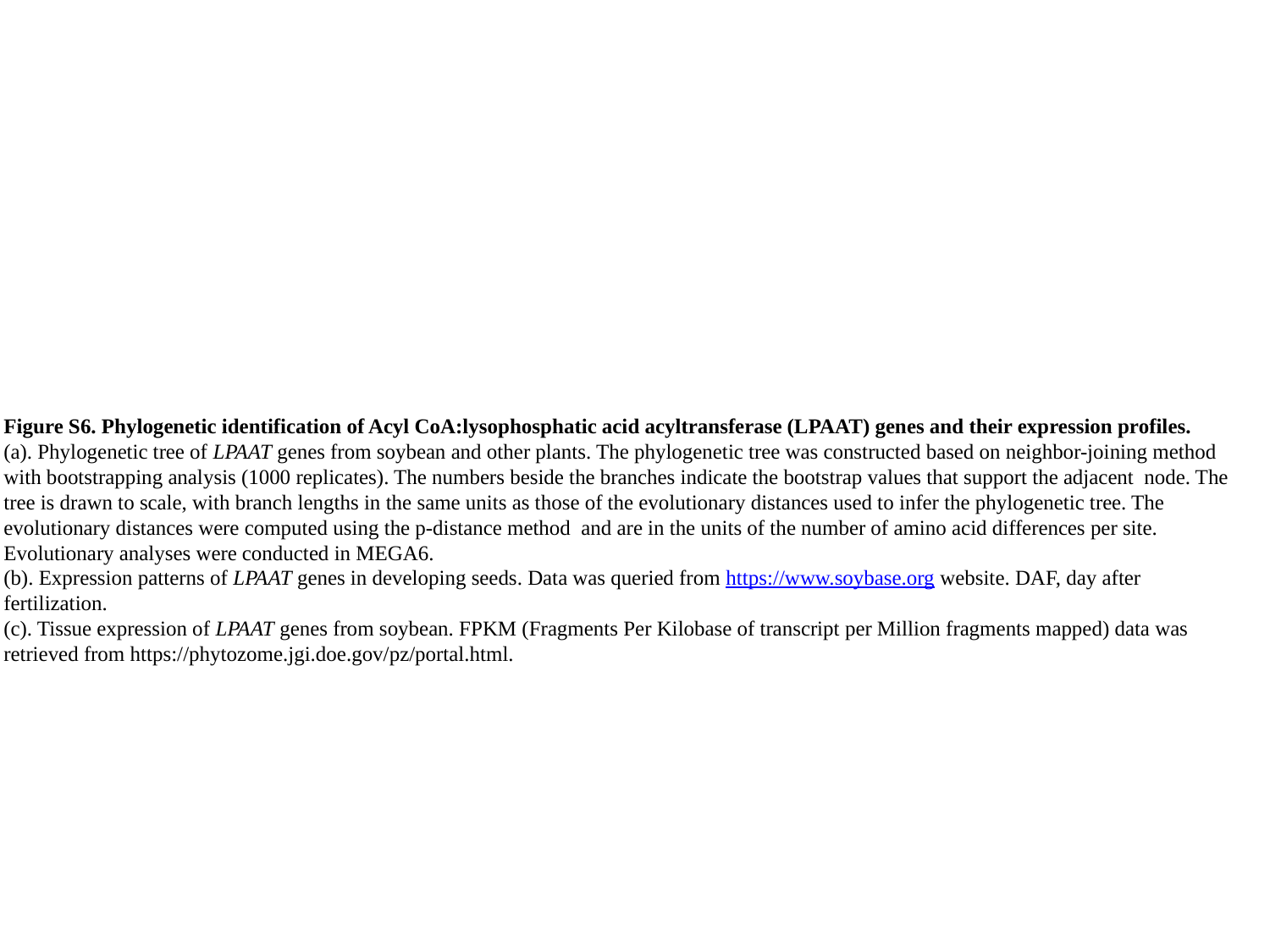

Figure S6. Phylogenetic identification of Acyl CoA:lysophosphatic acid acyltransferase (LPAAT) genes and their expression profiles.
(a). Phylogenetic tree of LPAAT genes from soybean and other plants. The phylogenetic tree was constructed based on neighbor-joining method with bootstrapping analysis (1000 replicates). The numbers beside the branches indicate the bootstrap values that support the adjacent node. The tree is drawn to scale, with branch lengths in the same units as those of the evolutionary distances used to infer the phylogenetic tree. The evolutionary distances were computed using the p-distance method and are in the units of the number of amino acid differences per site. Evolutionary analyses were conducted in MEGA6.
(b). Expression patterns of LPAAT genes in developing seeds. Data was queried from https://www.soybase.org website. DAF, day after fertilization.
(c). Tissue expression of LPAAT genes from soybean. FPKM (Fragments Per Kilobase of transcript per Million fragments mapped) data was retrieved from https://phytozome.jgi.doe.gov/pz/portal.html.

## Slide 13
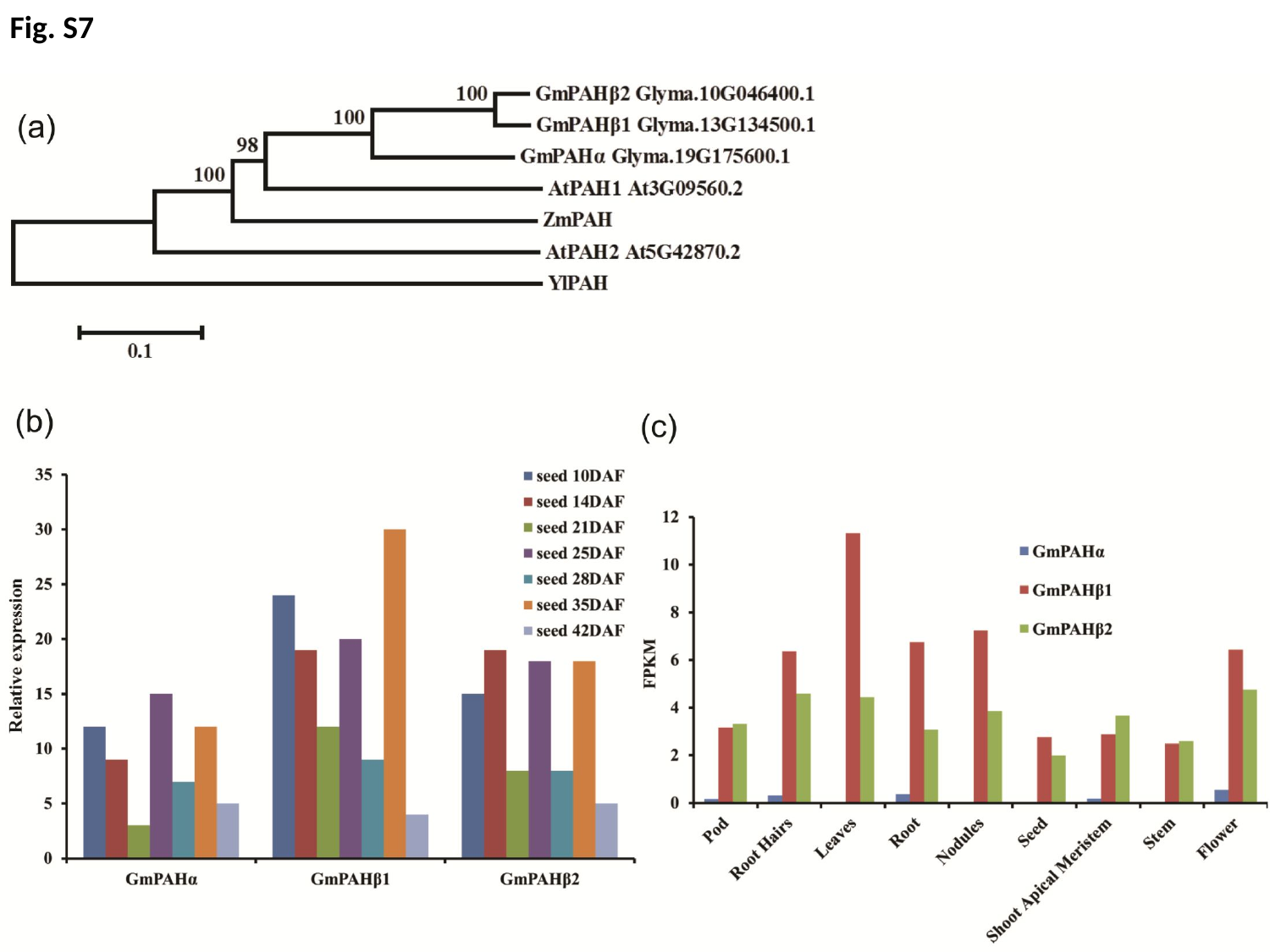

Fig. S7

## Slide 14
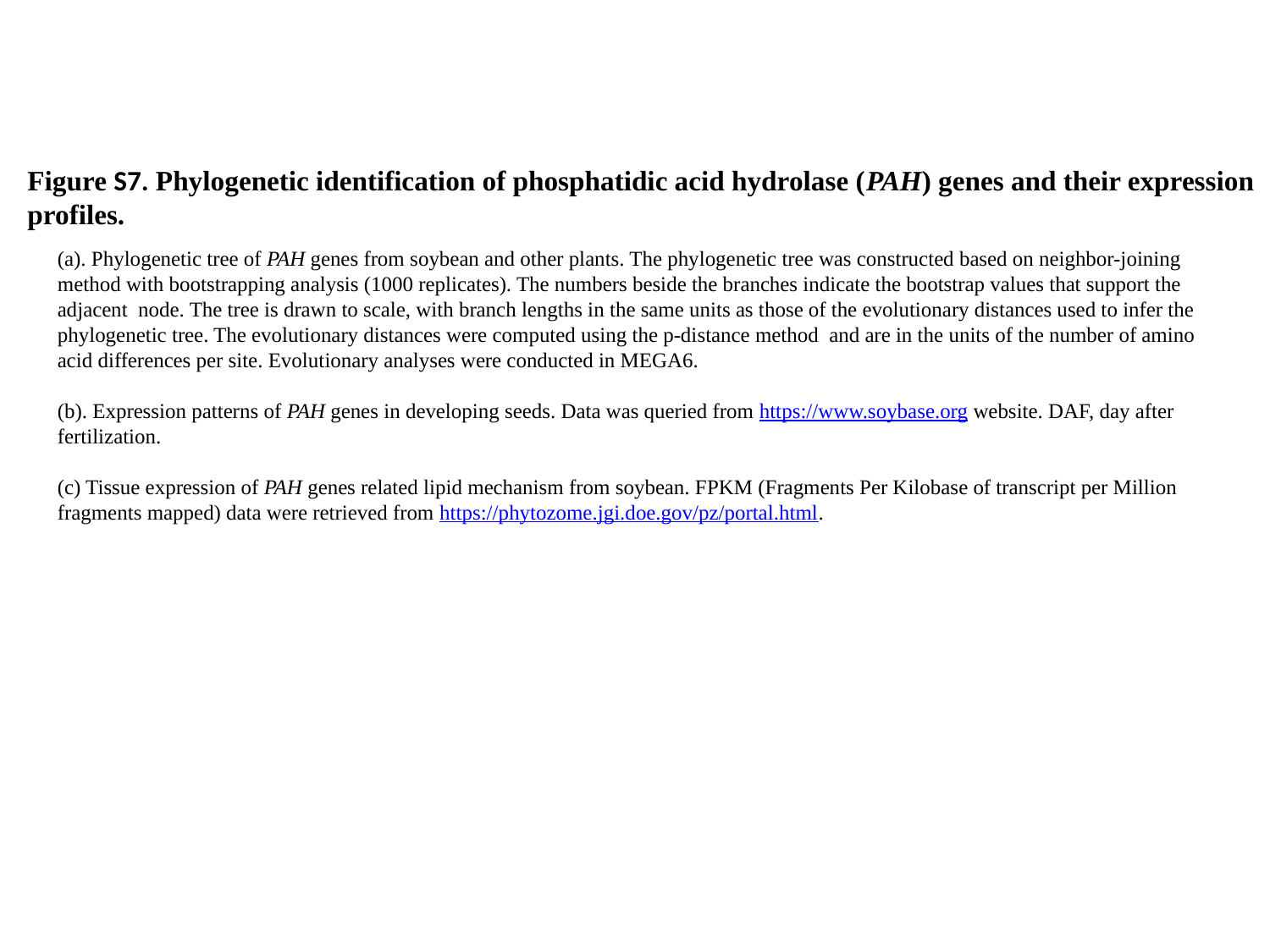

Figure S7. Phylogenetic identification of phosphatidic acid hydrolase (PAH) genes and their expression profiles.
(a). Phylogenetic tree of PAH genes from soybean and other plants. The phylogenetic tree was constructed based on neighbor-joining method with bootstrapping analysis (1000 replicates). The numbers beside the branches indicate the bootstrap values that support the adjacent node. The tree is drawn to scale, with branch lengths in the same units as those of the evolutionary distances used to infer the phylogenetic tree. The evolutionary distances were computed using the p-distance method and are in the units of the number of amino acid differences per site. Evolutionary analyses were conducted in MEGA6.
(b). Expression patterns of PAH genes in developing seeds. Data was queried from https://www.soybase.org website. DAF, day after fertilization.
(c) Tissue expression of PAH genes related lipid mechanism from soybean. FPKM (Fragments Per Kilobase of transcript per Million fragments mapped) data were retrieved from https://phytozome.jgi.doe.gov/pz/portal.html.

## Slide 15
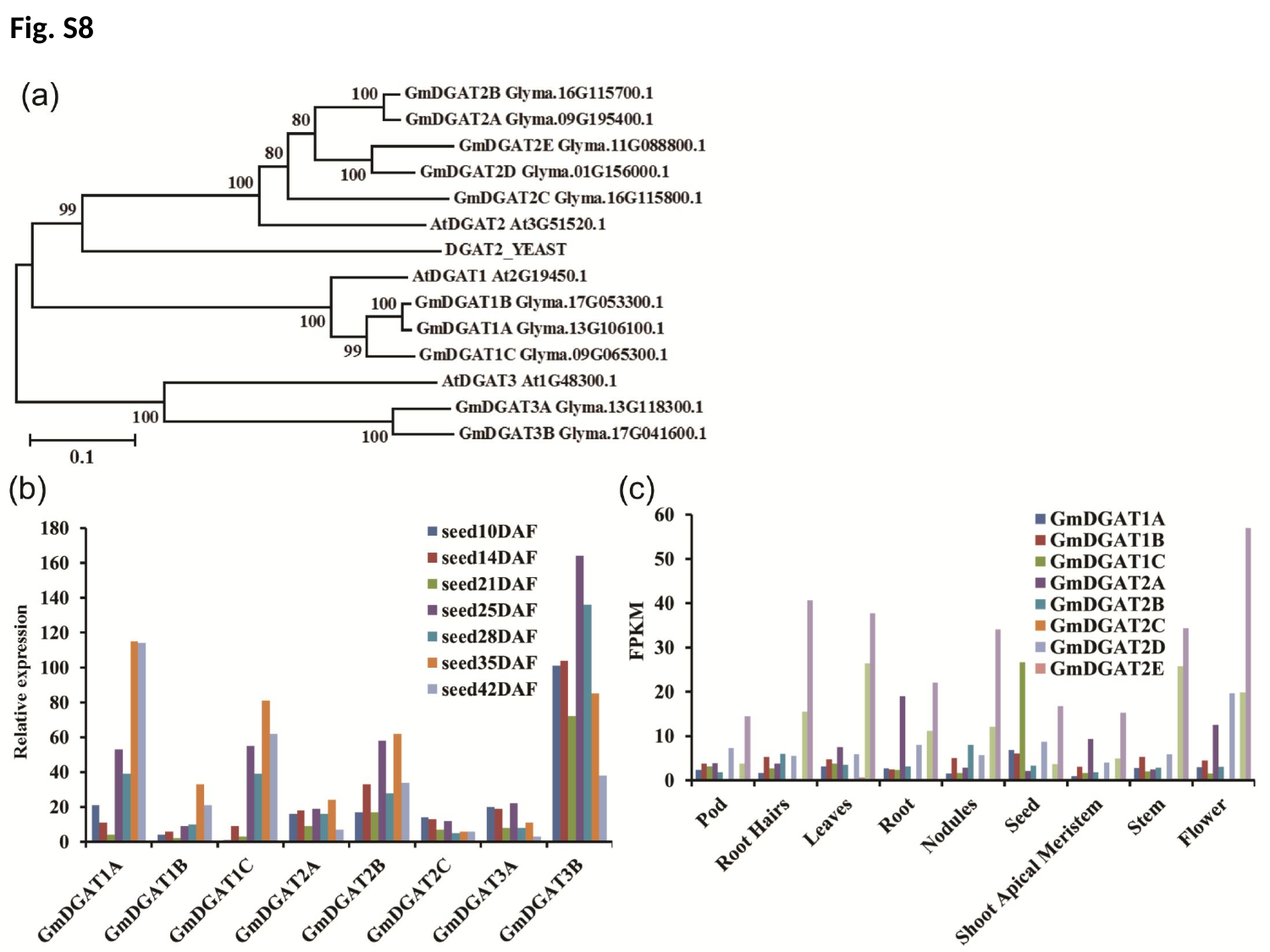

Fig. S8

## Slide 16
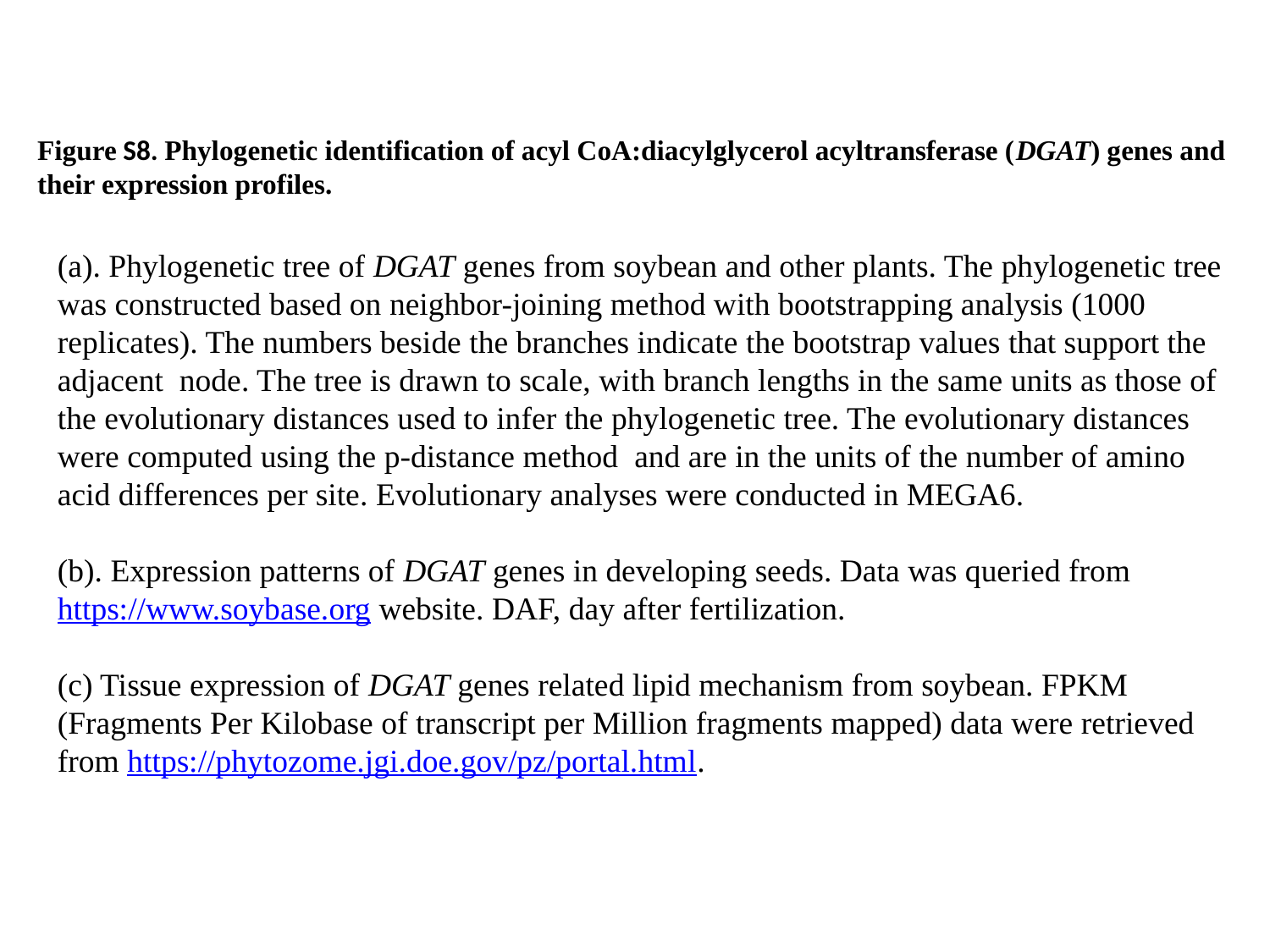

Figure S8. Phylogenetic identification of acyl CoA:diacylglycerol acyltransferase (DGAT) genes and their expression profiles.
(a). Phylogenetic tree of DGAT genes from soybean and other plants. The phylogenetic tree was constructed based on neighbor-joining method with bootstrapping analysis (1000 replicates). The numbers beside the branches indicate the bootstrap values that support the adjacent node. The tree is drawn to scale, with branch lengths in the same units as those of the evolutionary distances used to infer the phylogenetic tree. The evolutionary distances were computed using the p-distance method and are in the units of the number of amino acid differences per site. Evolutionary analyses were conducted in MEGA6.
(b). Expression patterns of DGAT genes in developing seeds. Data was queried from https://www.soybase.org website. DAF, day after fertilization.
(c) Tissue expression of DGAT genes related lipid mechanism from soybean. FPKM (Fragments Per Kilobase of transcript per Million fragments mapped) data were retrieved from https://phytozome.jgi.doe.gov/pz/portal.html.

## Slide 17
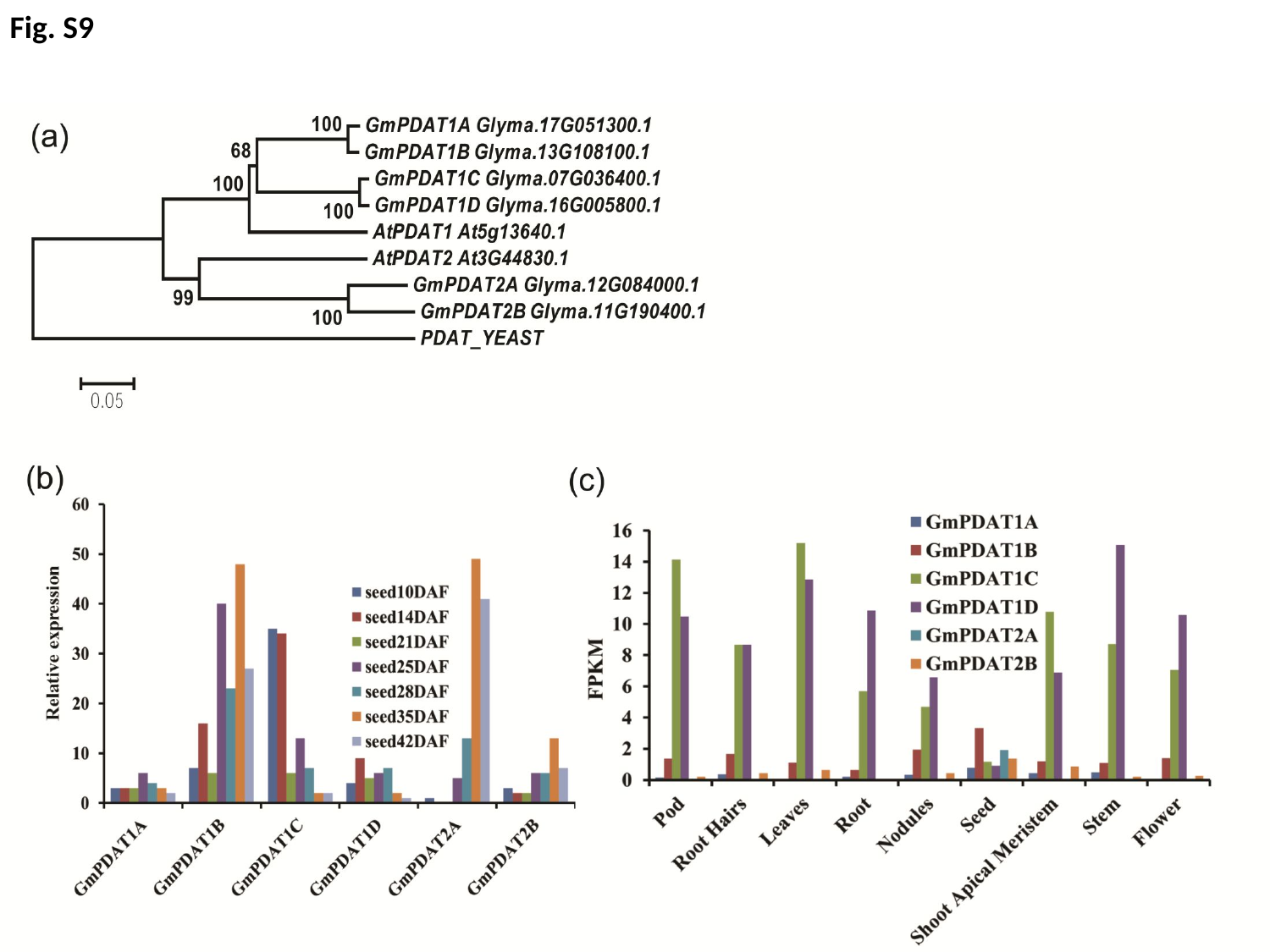

Fig. S9

## Slide 18
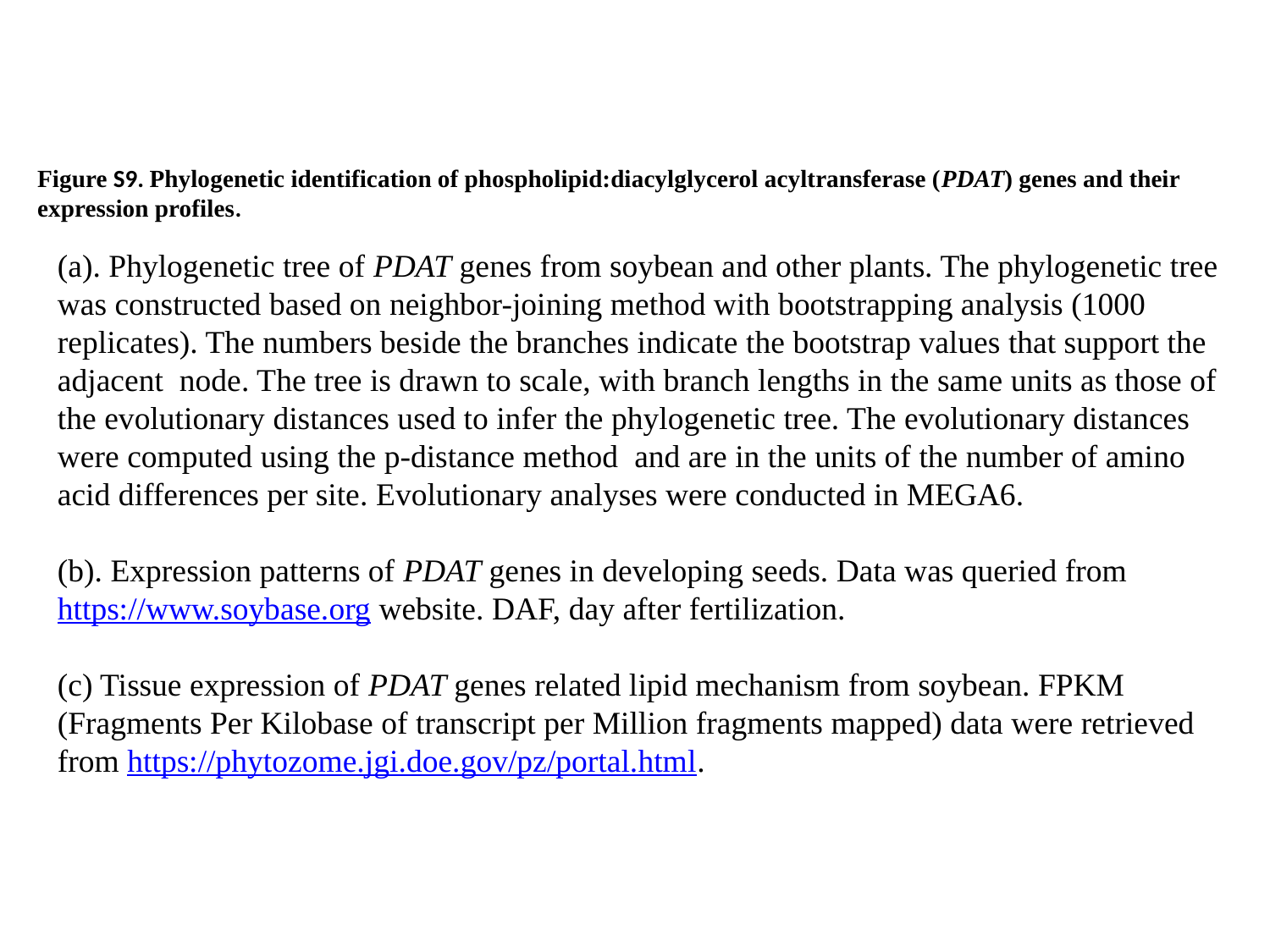

Figure S9. Phylogenetic identification of phospholipid:diacylglycerol acyltransferase (PDAT) genes and their expression profiles.
(a). Phylogenetic tree of PDAT genes from soybean and other plants. The phylogenetic tree was constructed based on neighbor-joining method with bootstrapping analysis (1000 replicates). The numbers beside the branches indicate the bootstrap values that support the adjacent node. The tree is drawn to scale, with branch lengths in the same units as those of the evolutionary distances used to infer the phylogenetic tree. The evolutionary distances were computed using the p-distance method and are in the units of the number of amino acid differences per site. Evolutionary analyses were conducted in MEGA6.
(b). Expression patterns of PDAT genes in developing seeds. Data was queried from https://www.soybase.org website. DAF, day after fertilization.
(c) Tissue expression of PDAT genes related lipid mechanism from soybean. FPKM (Fragments Per Kilobase of transcript per Million fragments mapped) data were retrieved from https://phytozome.jgi.doe.gov/pz/portal.html.

## Slide 19
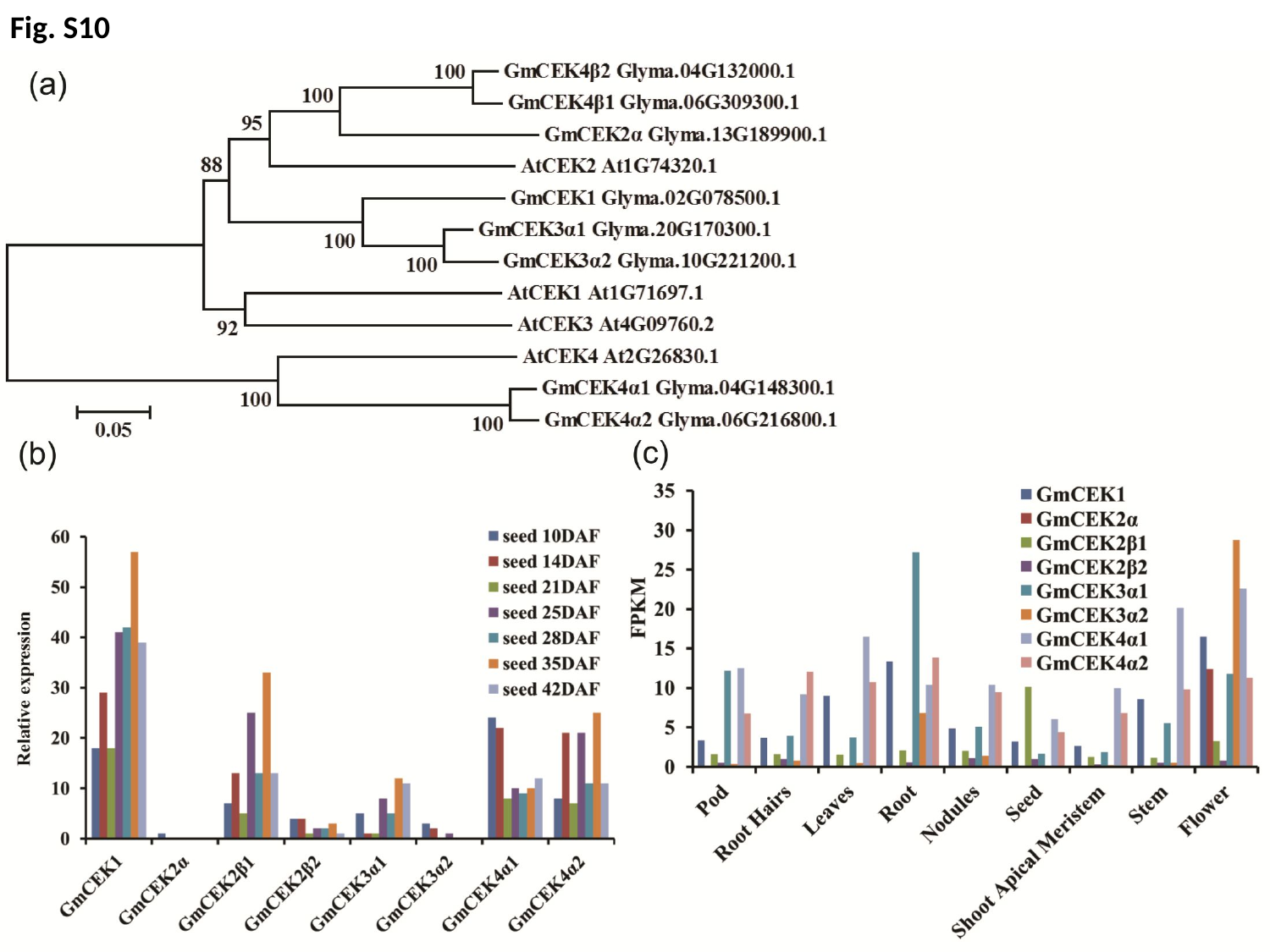

Fig. S10

## Slide 20
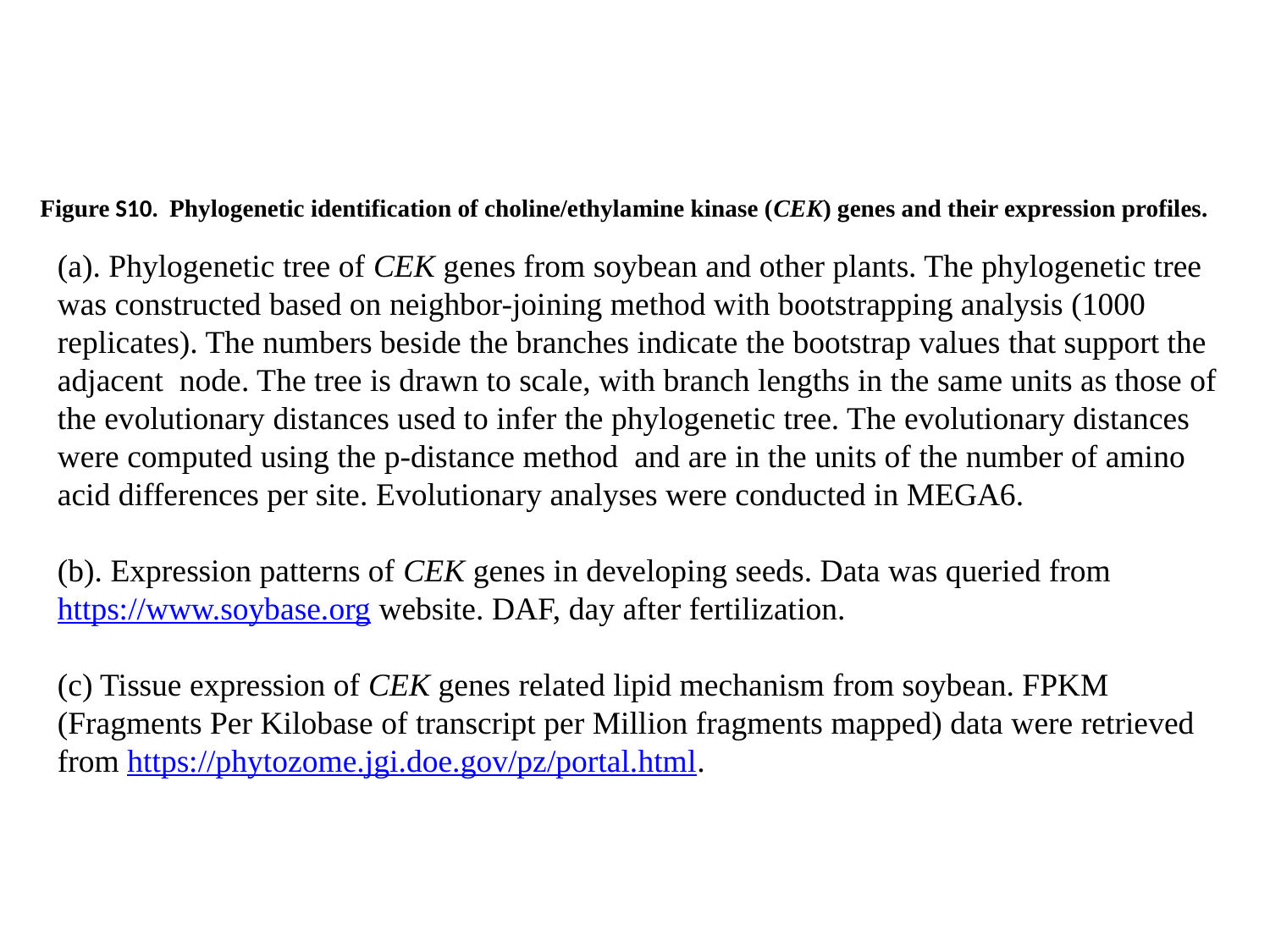

Figure S10. Phylogenetic identification of choline/ethylamine kinase (CEK) genes and their expression profiles.
(a). Phylogenetic tree of CEK genes from soybean and other plants. The phylogenetic tree was constructed based on neighbor-joining method with bootstrapping analysis (1000 replicates). The numbers beside the branches indicate the bootstrap values that support the adjacent node. The tree is drawn to scale, with branch lengths in the same units as those of the evolutionary distances used to infer the phylogenetic tree. The evolutionary distances were computed using the p-distance method and are in the units of the number of amino acid differences per site. Evolutionary analyses were conducted in MEGA6.
(b). Expression patterns of CEK genes in developing seeds. Data was queried from https://www.soybase.org website. DAF, day after fertilization.
(c) Tissue expression of CEK genes related lipid mechanism from soybean. FPKM (Fragments Per Kilobase of transcript per Million fragments mapped) data were retrieved from https://phytozome.jgi.doe.gov/pz/portal.html.

## Slide 21
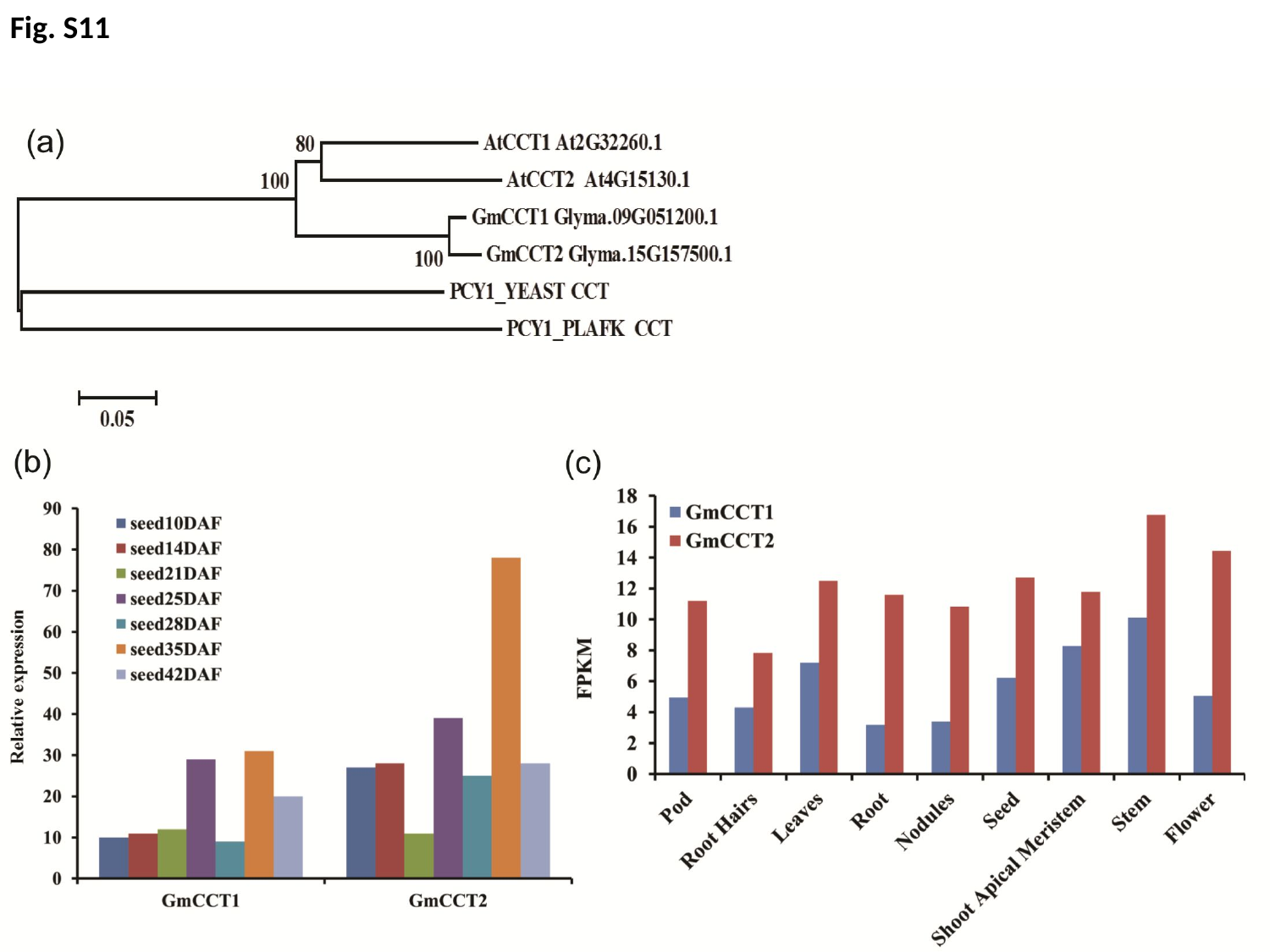

Fig. S11

## Slide 22
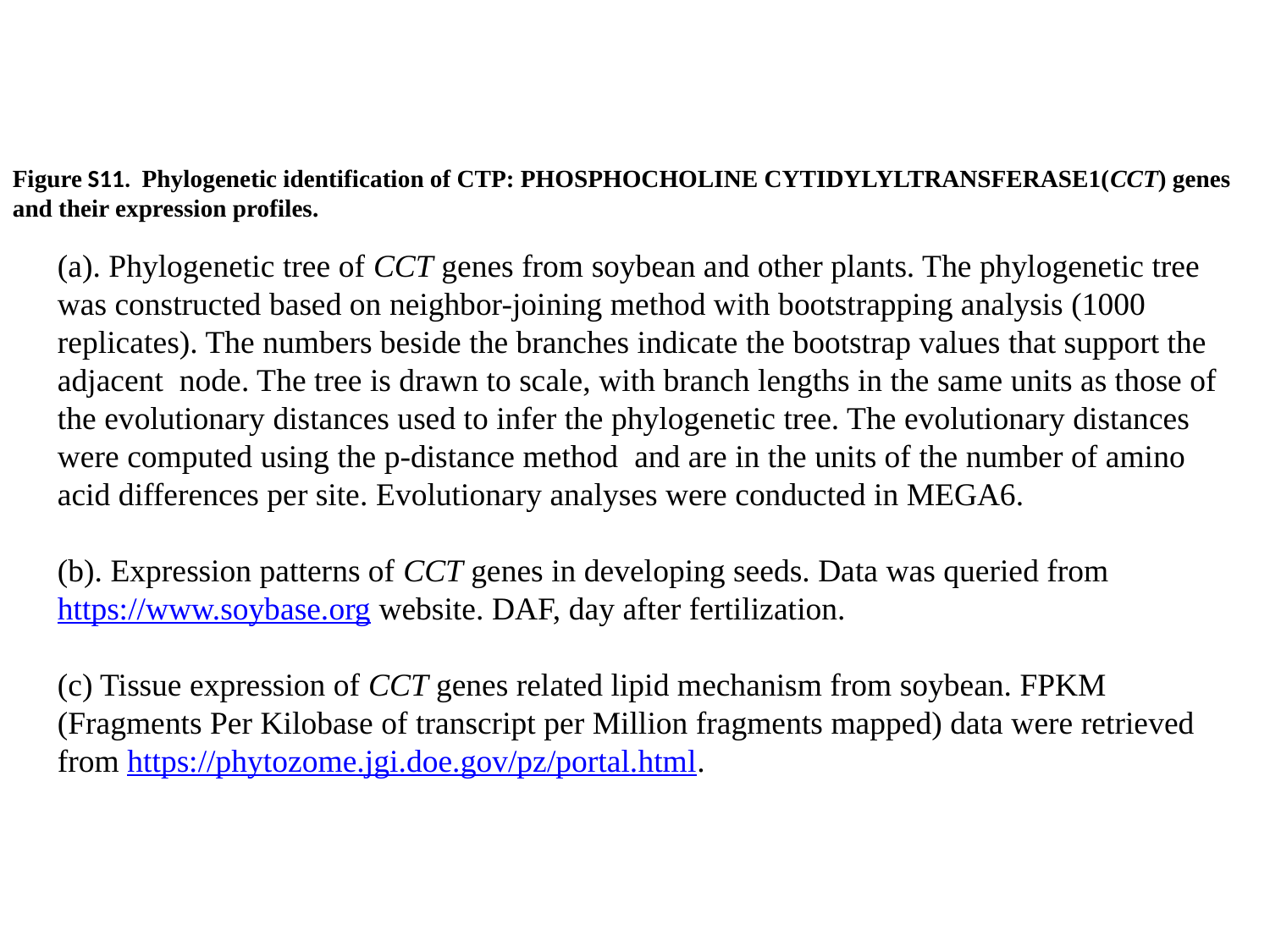

Figure S11. Phylogenetic identification of CTP: PHOSPHOCHOLINE CYTIDYLYLTRANSFERASE1(CCT) genes and their expression profiles.
(a). Phylogenetic tree of CCT genes from soybean and other plants. The phylogenetic tree was constructed based on neighbor-joining method with bootstrapping analysis (1000 replicates). The numbers beside the branches indicate the bootstrap values that support the adjacent node. The tree is drawn to scale, with branch lengths in the same units as those of the evolutionary distances used to infer the phylogenetic tree. The evolutionary distances were computed using the p-distance method and are in the units of the number of amino acid differences per site. Evolutionary analyses were conducted in MEGA6.
(b). Expression patterns of CCT genes in developing seeds. Data was queried from https://www.soybase.org website. DAF, day after fertilization.
(c) Tissue expression of CCT genes related lipid mechanism from soybean. FPKM (Fragments Per Kilobase of transcript per Million fragments mapped) data were retrieved from https://phytozome.jgi.doe.gov/pz/portal.html.

## Slide 23
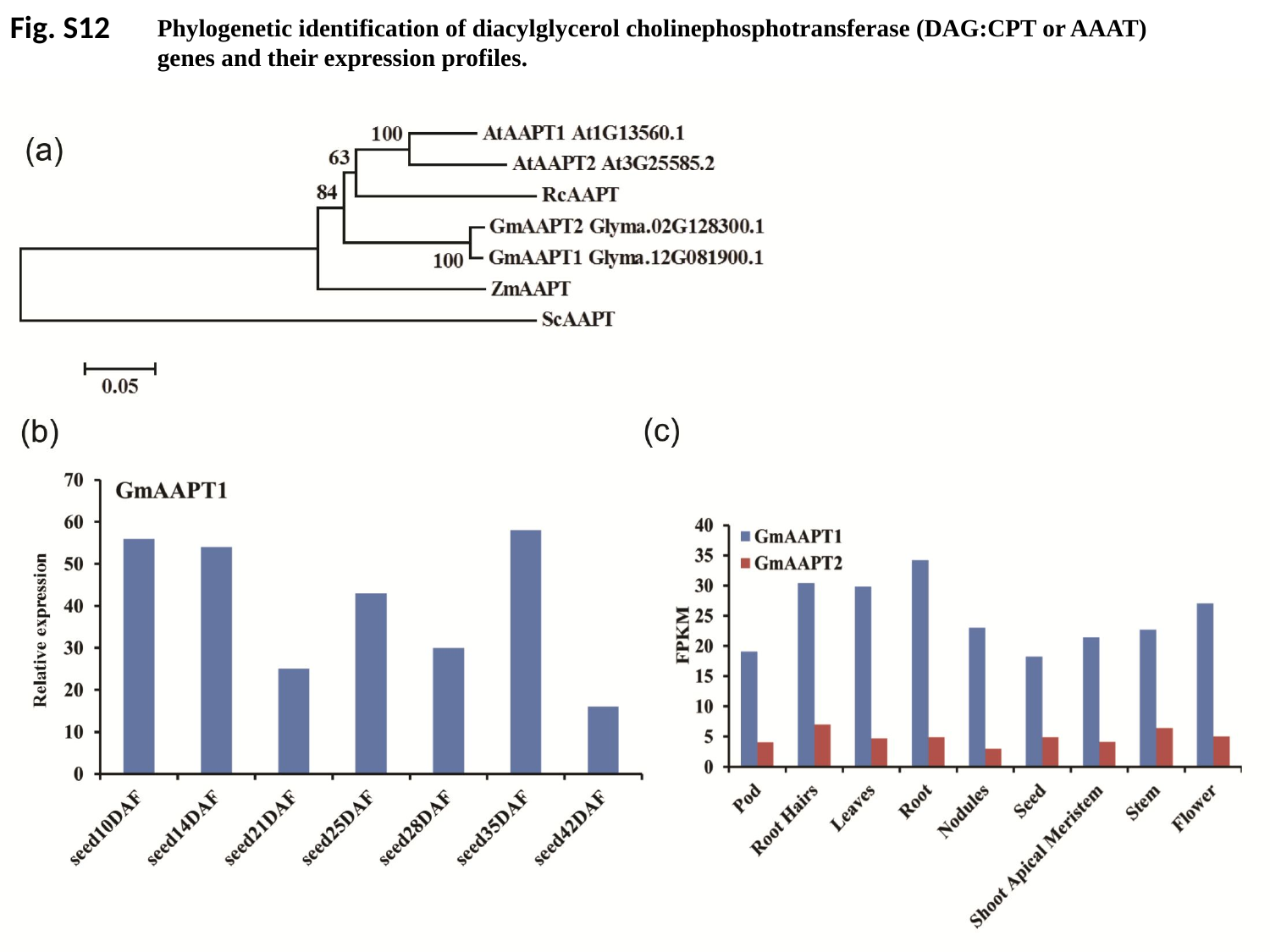

Fig. S12
Phylogenetic identification of diacylglycerol cholinephosphotransferase (DAG:CPT or AAAT) genes and their expression profiles.

## Slide 24
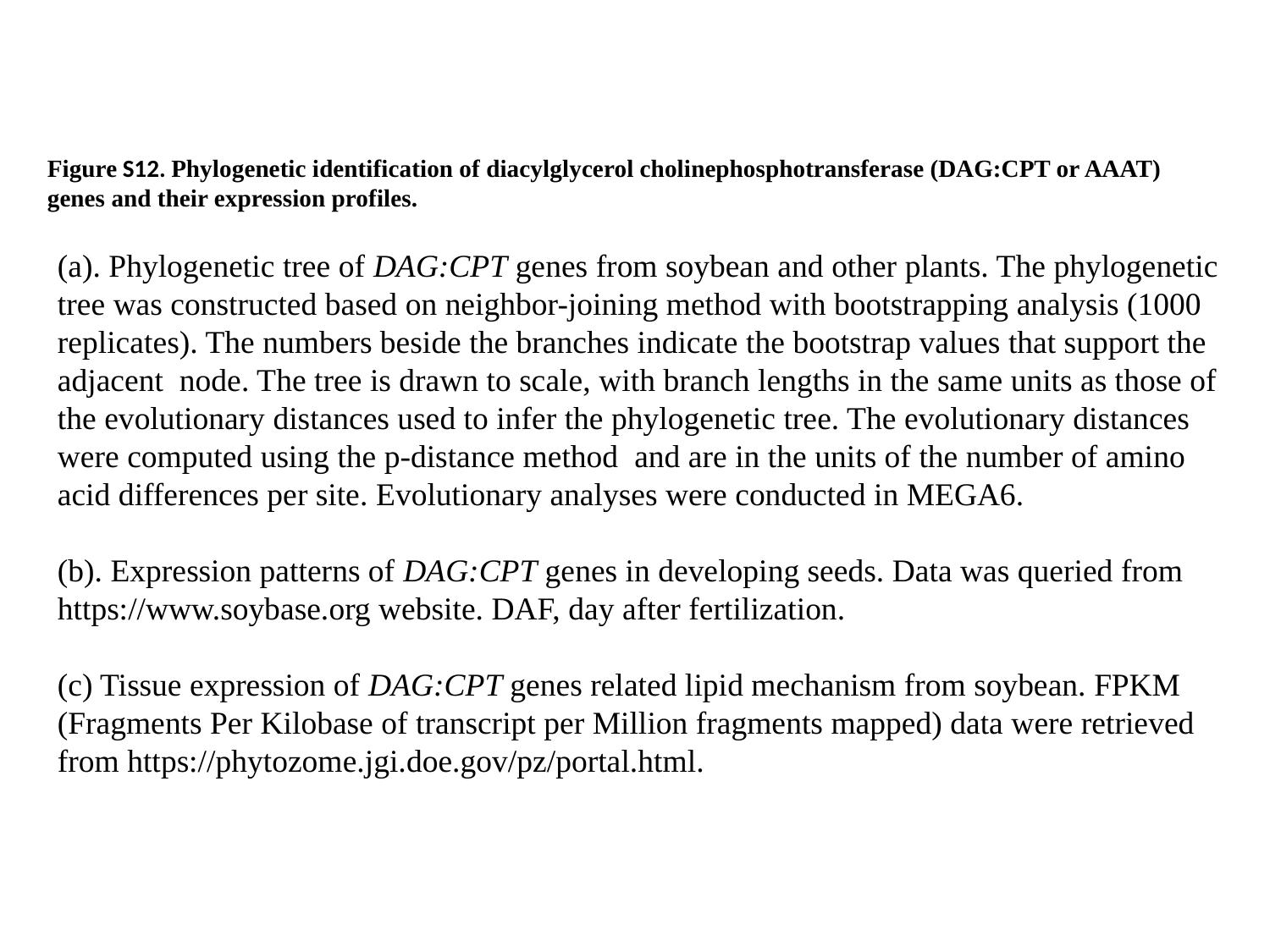

Figure S12. Phylogenetic identification of diacylglycerol cholinephosphotransferase (DAG:CPT or AAAT) genes and their expression profiles.
(a). Phylogenetic tree of DAG:CPT genes from soybean and other plants. The phylogenetic tree was constructed based on neighbor-joining method with bootstrapping analysis (1000 replicates). The numbers beside the branches indicate the bootstrap values that support the adjacent node. The tree is drawn to scale, with branch lengths in the same units as those of the evolutionary distances used to infer the phylogenetic tree. The evolutionary distances were computed using the p-distance method and are in the units of the number of amino acid differences per site. Evolutionary analyses were conducted in MEGA6.
(b). Expression patterns of DAG:CPT genes in developing seeds. Data was queried from https://www.soybase.org website. DAF, day after fertilization.
(c) Tissue expression of DAG:CPT genes related lipid mechanism from soybean. FPKM (Fragments Per Kilobase of transcript per Million fragments mapped) data were retrieved from https://phytozome.jgi.doe.gov/pz/portal.html.

## Slide 25
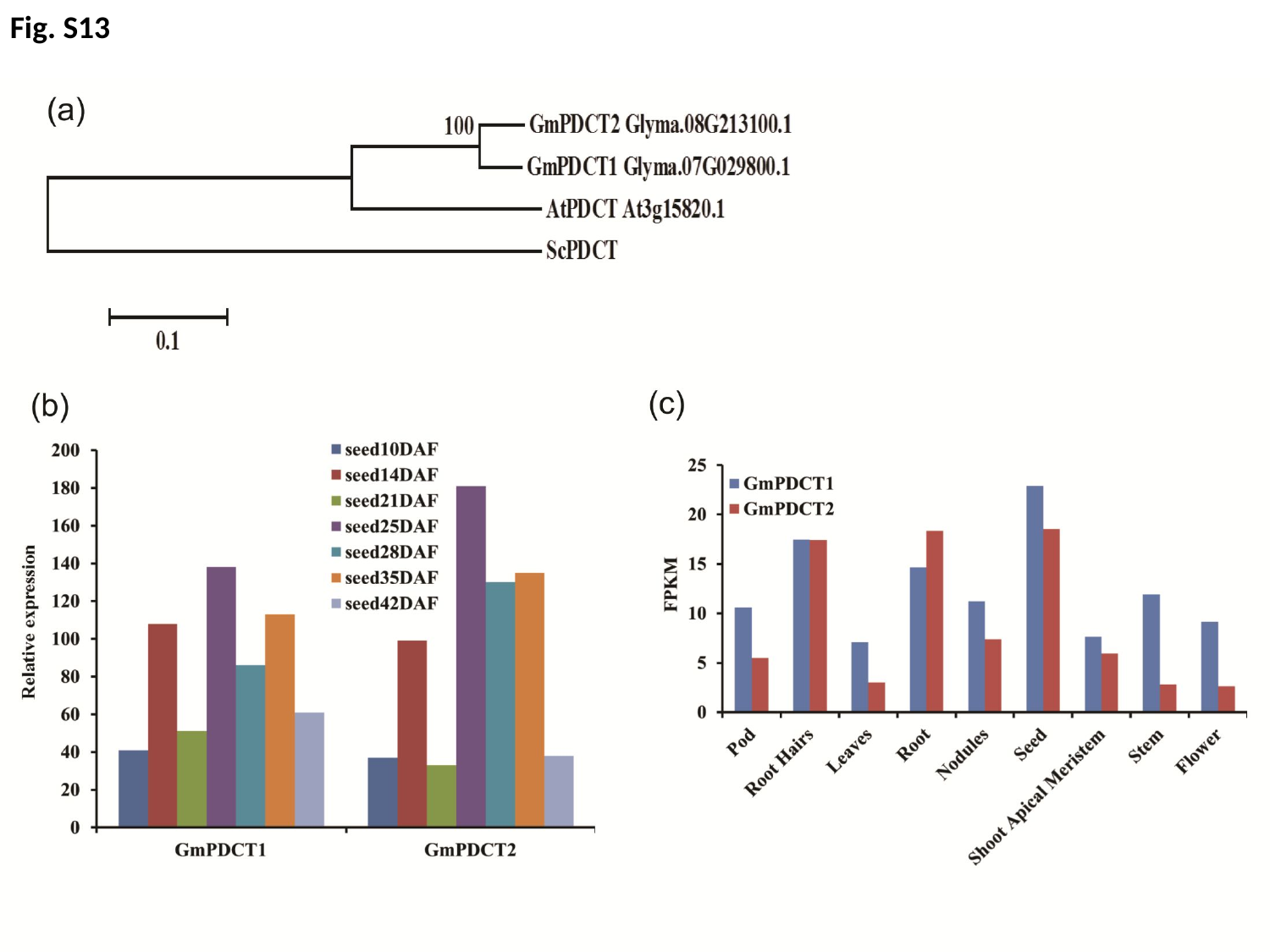

Fig. S13

## Slide 26
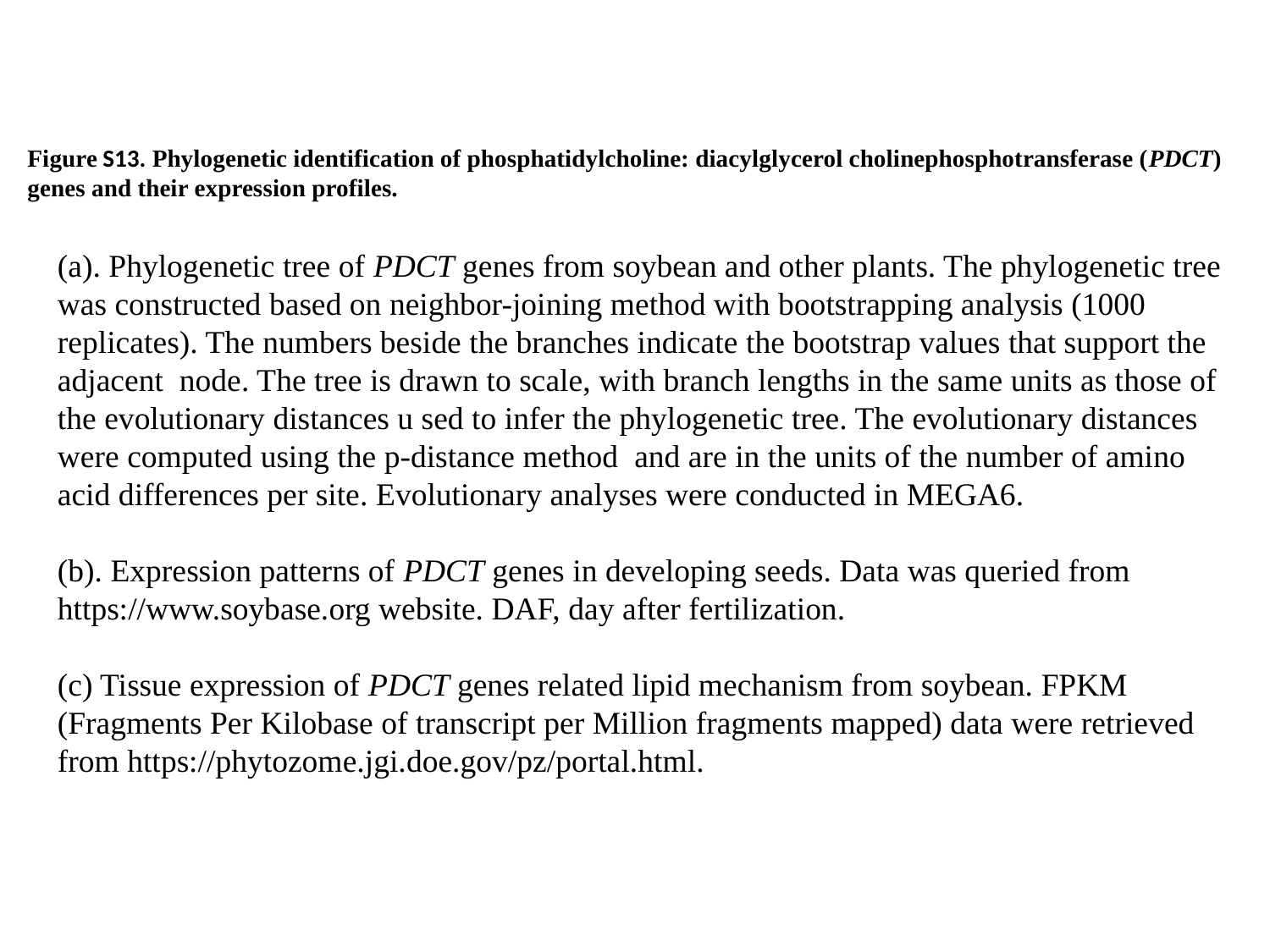

Figure S13. Phylogenetic identification of phosphatidylcholine: diacylglycerol cholinephosphotransferase (PDCT) genes and their expression profiles.
(a). Phylogenetic tree of PDCT genes from soybean and other plants. The phylogenetic tree was constructed based on neighbor-joining method with bootstrapping analysis (1000 replicates). The numbers beside the branches indicate the bootstrap values that support the adjacent node. The tree is drawn to scale, with branch lengths in the same units as those of the evolutionary distances u sed to infer the phylogenetic tree. The evolutionary distances were computed using the p-distance method and are in the units of the number of amino acid differences per site. Evolutionary analyses were conducted in MEGA6.
(b). Expression patterns of PDCT genes in developing seeds. Data was queried from https://www.soybase.org website. DAF, day after fertilization.
(c) Tissue expression of PDCT genes related lipid mechanism from soybean. FPKM (Fragments Per Kilobase of transcript per Million fragments mapped) data were retrieved from https://phytozome.jgi.doe.gov/pz/portal.html.

## Slide 27
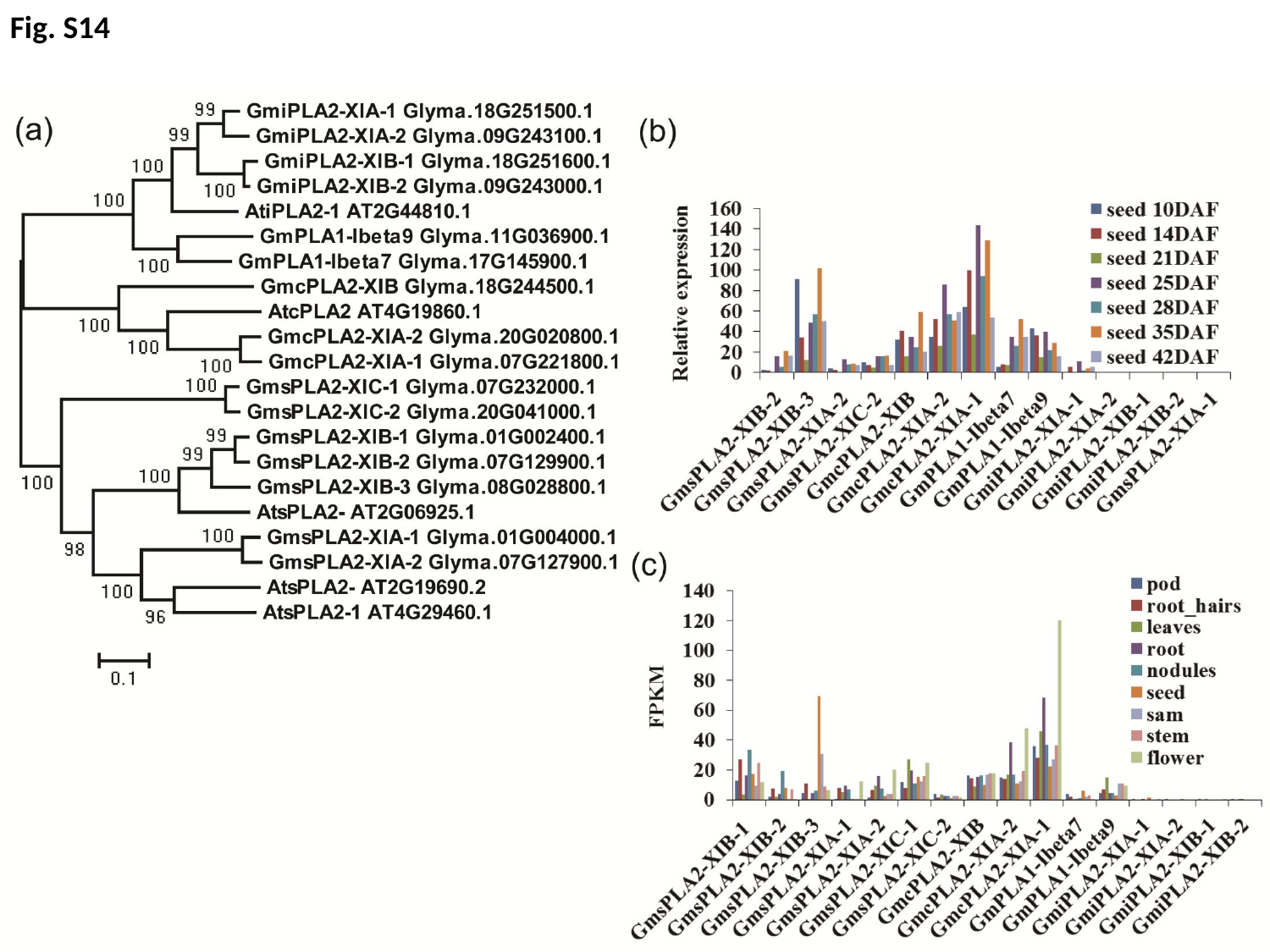

Fig. S14

## Slide 28
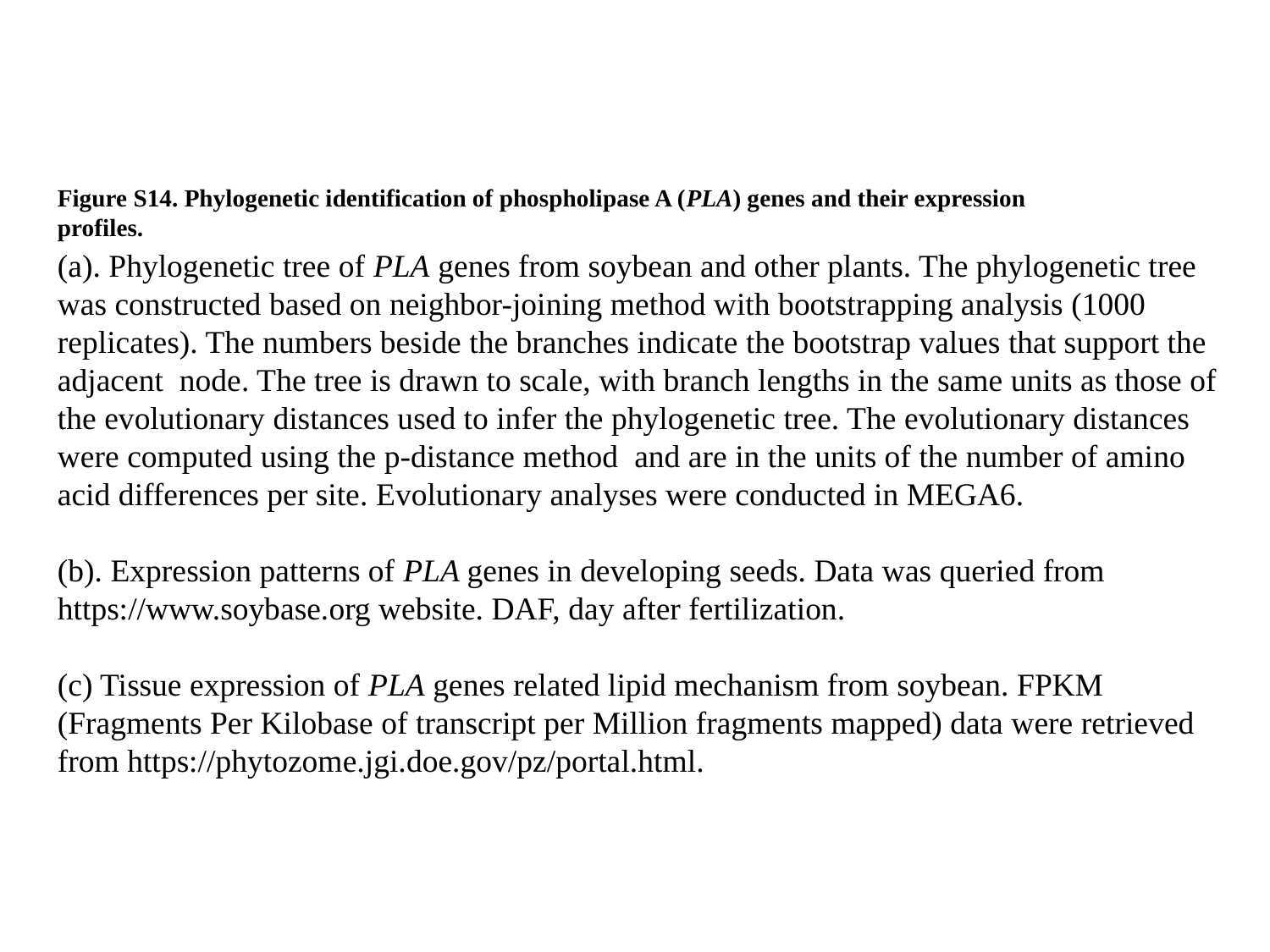

Figure S14. Phylogenetic identification of phospholipase A (PLA) genes and their expression profiles.
(a). Phylogenetic tree of PLA genes from soybean and other plants. The phylogenetic tree was constructed based on neighbor-joining method with bootstrapping analysis (1000 replicates). The numbers beside the branches indicate the bootstrap values that support the adjacent node. The tree is drawn to scale, with branch lengths in the same units as those of the evolutionary distances used to infer the phylogenetic tree. The evolutionary distances were computed using the p-distance method and are in the units of the number of amino acid differences per site. Evolutionary analyses were conducted in MEGA6.
(b). Expression patterns of PLA genes in developing seeds. Data was queried from https://www.soybase.org website. DAF, day after fertilization.
(c) Tissue expression of PLA genes related lipid mechanism from soybean. FPKM (Fragments Per Kilobase of transcript per Million fragments mapped) data were retrieved from https://phytozome.jgi.doe.gov/pz/portal.html.

## Slide 29
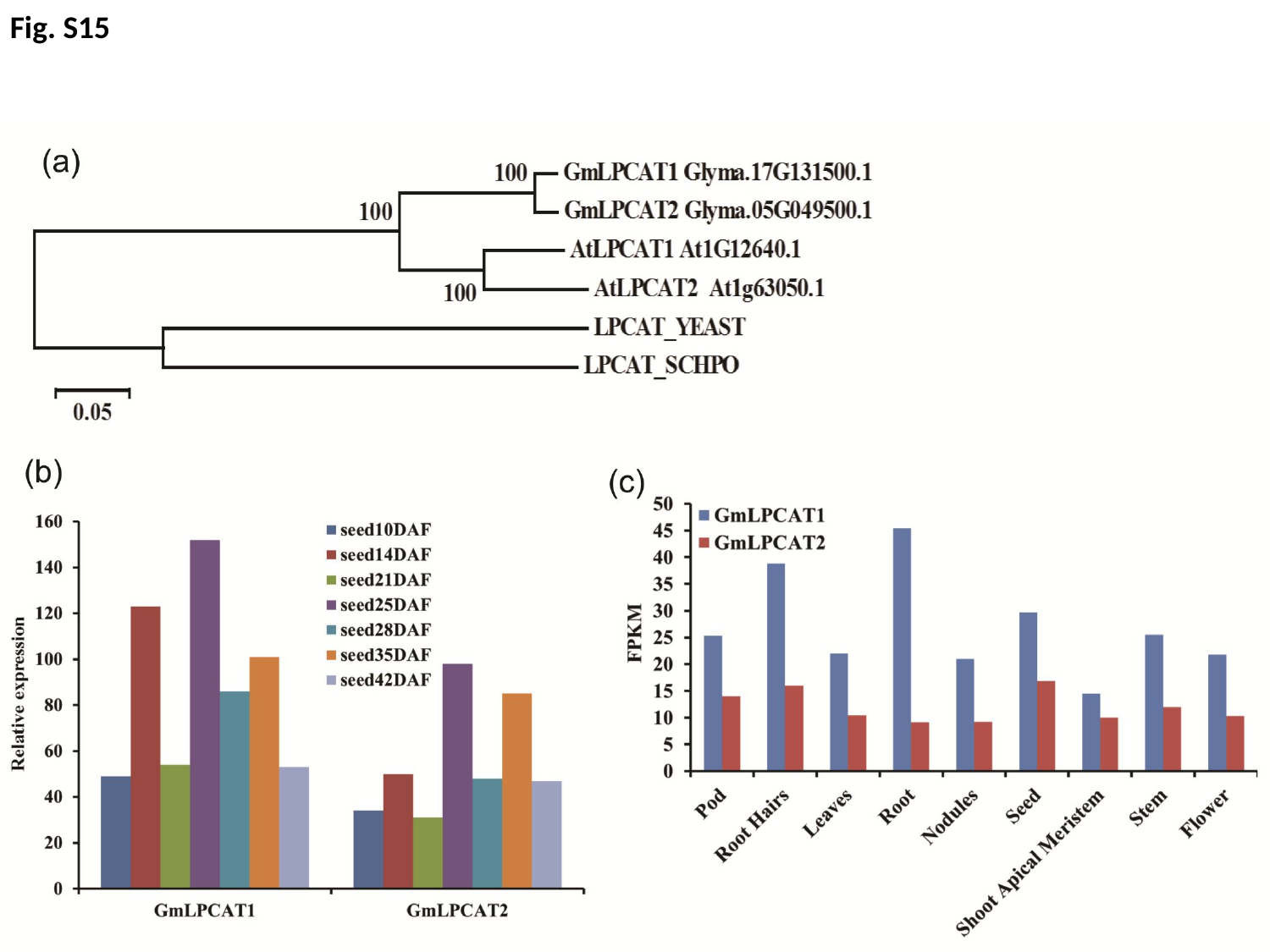

Fig. S15

## Slide 30
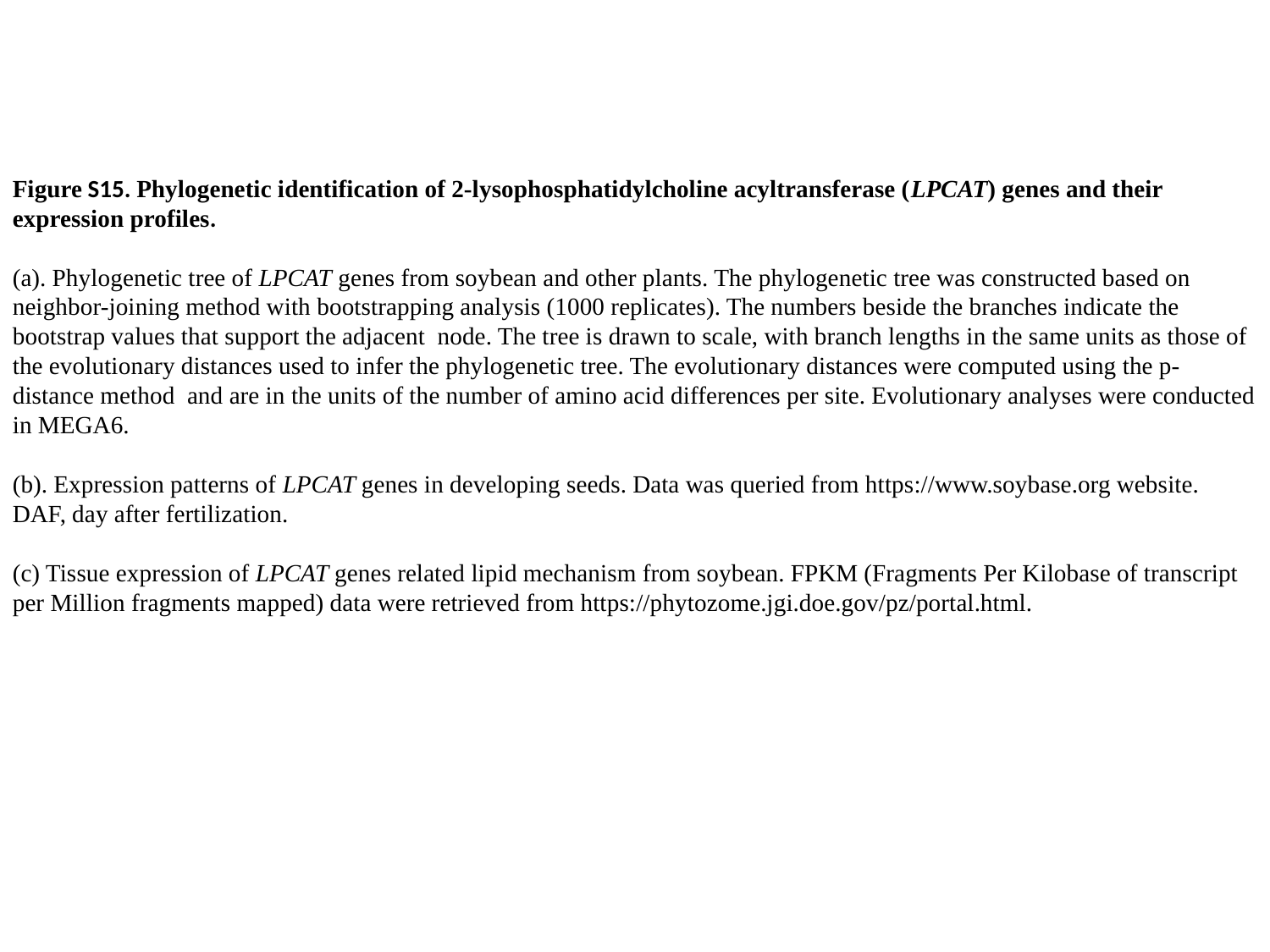

Figure S15. Phylogenetic identification of 2-lysophosphatidylcholine acyltransferase (LPCAT) genes and their expression profiles.
(a). Phylogenetic tree of LPCAT genes from soybean and other plants. The phylogenetic tree was constructed based on neighbor-joining method with bootstrapping analysis (1000 replicates). The numbers beside the branches indicate the bootstrap values that support the adjacent node. The tree is drawn to scale, with branch lengths in the same units as those of the evolutionary distances used to infer the phylogenetic tree. The evolutionary distances were computed using the p-distance method and are in the units of the number of amino acid differences per site. Evolutionary analyses were conducted in MEGA6.
(b). Expression patterns of LPCAT genes in developing seeds. Data was queried from https://www.soybase.org website. DAF, day after fertilization.
(c) Tissue expression of LPCAT genes related lipid mechanism from soybean. FPKM (Fragments Per Kilobase of transcript per Million fragments mapped) data were retrieved from https://phytozome.jgi.doe.gov/pz/portal.html.
