# Supplementary material for: PLDα1-knockdown soybean seeds display higher unsaturated glycerolipid contents and seed vigor in high temperature and humidity environments
Source: Biotechnol Biofuels. 2019 Jan 4;12:9. doi: 10.1186/s13068-018-1340-4 (PMC6319013; doi:10.1186/s13068-018-1340-4)
Supplement: Supplementary file 2 — Additional file 2: Table S1. The Quantitative RT-PCR primers used in this study. Table S2. PLD genes in soybean genome. Table S3. Expression pattern in different tissue. Table S4. Relative expression of GmPLDɑs in different developing seeds after fertilization. Data S1. Protein sequences for phylogenic tree construction. [file 13068_2018_1340_MOESM2_ESM.doc]

**Additional files 2 for:**

**PLDα1 knockdown soybean seeds display higher unsaturated glycerolipid contents and seed vigor in high temperature and humidity environments**

Gaoyang Zhang^1^, Sung-Chul Bahn^2^, Geliang Wang^2^, Yanrui Zhang^1^, Beibei Chen^3^, Yuliang Zhang^4^， Xuemin Wang^2^, Jian Zhao*^1^

**Contents：**

Table S1. The Quantitative RT-PCR primers used in this study.

Table S2. *PLD* genes in soybean genome.

Table S3. Expression pattern in different tissue.

Table S4. Relative expression of *GmPLDɑ*s in different developing seeds after fertilization.

Data S1. Protein sequences for phylogenic tree construction.

**Table S1.The Quantitative RT-PCR primers used in this study.**

| Gene name | Forward primer sequence(5'--3') | Reverse primer sequence(5'--3') | Length(bp) |
| --- | --- | --- | --- |
| GmPLDα1 | GTTGAAGGGAGAGAAGGATAC | AAGGGGTGTTGACGAAGGAAG | 182 |
| GmPLDα3 | CACGAAGCGATTAGATTG | CGAAGGGAAAACGAAAGA | 193 |
| GmPLDα4 | AGATGACTGTGCATTCCCA | CGAAGAGAAAACGAAGAAGA | 188 |
| GmPLDα5 | ATCACATTAAGGCGTAAGAG | CAGTCGTTGAGAGTTGGTAG | 186 |
| GmPLDα6 | CGATCCCTGCTTTATTTAG | CCAGTATGTAGTCTATCAAC | 189 |
| GmPLDα | AGGTCAATGGATGGTGCTAGGG | TCACTTTCTGGCTGGAGGAAGG | 167 |
| GmPLDβ1 | ATGGCTCATTTGGTTTATGGTG | TTCGGGACAACATGGAAACC | 214 |
| GmPLDδ1 | AGTGAATGAGATTGCCGAA | AGTGAGCTCCCAATATCTTA | 173 |
| GmPLDδ2 | GTCTCAACAGCATATAAATAT | TTTATTCACTTTATGCACACAC | 303 |
| GmFAD2-1A | TAATGCCGCAGTAGAGGACGA | GCAGTGTGGTGGAATTGCTT | 201 |
| GmFAD2-1B | TGTAATGTTTCAGCGTTGG | AGTGAATGGTGGCTTTGTG | 150 |
| GmFAD2-2A | TTGGTGTCACTGCTCATCG | CATCTTCACACAATCTGA | 158 |
| GmFAD3B | GCACTATCCCATAAGAGTAT | TGTGTCTTTAACCATTGCTG | 143 |
| GmFAD3C | GTTATCTAAGGGGTGGTCTT | CACGATAATACTTTCCTAGC | 171 |
| GmFAD6A | CTGCTCACACATAATGGCTTG | TGGTTGTACTGGAATTGCC | 223 |
| GmPAHβ1 | ACTTTGCGACACTAATGGTGATG | AGCCCCTTTTGAATCAGTTTTTA | 142 |
| GmPAHβ2 | AATCTAGACGTTGTGACACTAACGG | TCTTCTTGATTTTTAATCTCCACG | 124 |
| GmDGAT1A | ACTCCATCAGCAGCGACGC | GTGACTCTGCAACTGACGGA | 161 |
| GmDGAT1C | CAACGTCTCAAATACTTCCATC | TATGAGTTAAATTCTCCAAC | 166 |
| GmDGAT3B | GCATCTCCACCGCAGGATGGAT | TGATTCTGGTTGGTGGCTAT | 213 |
| GmPDAT1B | CACAAAAGCCAAAGTACCAT | GGATTTCAGATTCGGACACG | 183 |
| GmLPAAT2α1 | CGCAGAGGAAAAGAAAAGAC | CCTGAATGAGGTTAACGATGAG | 190 |
| GmLPAAT2α2 | TAGATCACACACATTACCTG | CAAGGACTGAACACATACGAG | 187 |
| GmPDCT1 | CGACGGCGCTAAAGGCGT | GCAGGAGTGCTTGGAAGAG | 170 |
| GmPDCT2 | CTATCCACAACCCACAGAGCAC | TCAGACTCTTGCCCTCTCACTC | 133 |
| GmDAG-CPT1 | GGCAAGGCTTTGGTCAGTACTA | GTGGCTGAATTGAAACACTCG | 124 |
| GmDAG-CPT2 | ATCTCACTGAAGCCCTAATCCC | CAAACGGAATGAGACTCAGGC | 149 |
| GmCCT1 | CAGAACAGAGTGTGATGGAAG | CTCATCATTGCAGCATCCAAC | 191 |
| GmCCT2 | GTCCAAATCAAATGGTCATCGT | GCATAGACTCGAACAGGAGGAG | 183 |
| GmPLA2-XIB-1 | GACACTAGAAAACAAACCACCA | GTAGGACAAGGAGATGAATC | 181 |
| GmPLA2-XIB-3 | CACTCCCATCTTTCTTTCTGAG | GTGGAAAGAAGGAAAAGGTGAAG | 167 |
| GmLPCAT1 | AGAAGACATGACAGAGTACG | GAAGCGAAGAAGAAGAAGAAG | 185 |
| GmLPCAT2 | GAGGTTCAATCGAGGTGTGTTC | AGCAGAGGAGGAAGCGTAGCA | 155 |
| GmPLDβ3 | GTTCTTTACAACATCCATAT | TATGTGGCTAGAATAAGGAG | 170 |
| GmPLDζ3 | GCCATCGTCTCCGTCTCTCG | CATTCTTTAACCTGCTCTTGT | 197 |
| GmACIN | ATGGCAGACACCGAGGACA | CCGACCAACAATGCTAGGAA | 117 |

| **Table S2. PLD genes identified in soybean genome. Chr, Chromosome.** | | | | | | | |
| --- | --- | --- | --- | --- | --- | --- | --- |
|  | Photozome locus9.1 | Photozome locus10.0 | Chr | Protein length | NCBI number | Gene ID | Protein ID |
| GmPLDα1 | Glyma13g44170 | Glyma.13G364900.1 | 13 | 807 | XM_003543629.2 | 100784434 | XP_003543677.1 |
| GmPLDα2 | Glyma15g01120 | Glyma.15G008500.1 | 15 | 711 | KN671036.1 | 734313439 | KHN01336.1 |
| GmPLDα3 | Glyma08g22600 | Glyma.08G211700.1 | 8 | 788 | XM_003531662.2 | 100786410 | XP_003531710.1 |
| GmPLDα4 | Glyma07g03490 | Glyma.07G031100.1 | 7 | 809 | XM_003528286.2 | 100794849 | XP_003528334.1 |
| GmPLDα5 | Glyma06g07220 | Glyma.06G068600.1 | 6 | 826 | XM_006581294.1 | 100791688 | XP_006581357.1 |
| GmPLDα6 | Glyma06g07230 | Glyma.06G068700.1 | 6 | 821 | XM_006581295.1 | 100794115 | XP_006581358.1 |
| GmPLDβ1 | Glyma02g10360 | Glyma.02G093500.1 | 2 | 1106 | XM_003519977.2 | 100791059 | XP_003520025.1 |
| GmPLDβ2 | Glyma18g52560 | Glyma.18G288600.1 | 18 | 1097 | XM_003551775.2 | 100801286 | XP_003551823.1 |
| GmPLDβ3 | Glyma03g02120 | Glyma.03G018900.1 | 3 | 759 | XM_003529924.2 | 100805294 | XP_003529972.1 |
| GmPLDβ4 | Glyma07g08740 | Glyma.07G080400.1 | 7 | 1047 | XM_003529924.2 | 100805294 | XP_003529972.1 |
| GmPLDβ5 | Glyma01g42420 | Glyma.01G215100.1 | 1 | 853 | XM_003517402.2 | 100786241 | XP_003517450.1 |
| GmPLDδ1 | Glyma11g08640 | [Glyma.11G081500.1](http://phytozome.jgi.doe.gov/pz/portal.html#!gene?search=1&detail=1&crown&method=0&searchText=transcriptid:30531269) | 11 | 866 | XM_006590675.1 | 100814486 | XP_006590738.1 |
| GmPLDδ2 | Glyma01g36680 | Glyma.01G162100.1 | 1 | 864 | XM_003516489.2 | 100801787 | XM_003516489.2 |
| GmPLDδ3 | Glyma06g02310 | Glyma.06G020500.1 | 6 | 847 | XM_006581082.1 | 100789389 | XP_006581145.1 |
| GmPLDδ4 | Glyma04g02250 | Glyma.04G020400.1 | 4 | 847 | XM_003523206.2 | 100795859 | XP_003523254.1 |
| GmPLDδ5 | Glyma08g13350 | Glyma.08G126700.1 | 8 | 857 | XM_003532746.2 | 100810824 | XP_003532794.1 |
| GmPLDδ6 | Glyma05g30190 | Glyma.05G168300.1 | 5 | 857 | XM_003524186.2 | 100779740 | XP_003524234.1 |
| GmPLDε1 | Glyma15g02710 | Glyma.15G023500.1 | 15 | 759 | XM_003546621.2 | 100807431 | XP_003546669.1 |
| GmPLDε2 | Glyma07g01310 | Glyma.07G010900.1 | 7 | 769 | XM_006582957.1 | 100789235 | XP_006583020.1 |
| GmPLDε3 | Glyma08g20710 | Glyma.08G194100.1 | 8 | 776 | XM_006585458.1 | 100812642 | XP_006585521.1 |
| GmPLDζ1 | Glyma20g38200 | Glyma.20G238000.1 | 20 | 1120 | NM_001288593.1 | 100796914 | NP_001275522.1 |
| GmPLDζ2 | Glyma15g16270 | Glyma.15G152100.1 | 15 | 1123 | XM_003546322.2 | 100780514 | XP_003546370.1 |
| GmPLDζ3 | Glyma09g04620 | Glyma.09G041400.1 | 9 | 1126 | XM_003534784.2 | 100782187 | XP_003534832.1 |

**Table S3. Expression pattern of *GmPLDɑs* in different tussue. Data are retrieved from https://phytozome.jgi.doe.gov/pz/portal.html.**

|  | Pods | Root hairs | Leaves | Nodules | Root | Seed | Sam | Stem | Flower |
| --- | --- | --- | --- | --- | --- | --- | --- | --- | --- |
| GmPLD1 | 63.398 | 71.736 | 122.139 | 80.232 | 195.273 | 22.16 | 64.519 | 68.54 | 70.273 |
| GmPLD2 | 1.318 | 0.431 | 0.916 | 2.684 | 0.054 | 0 | 0.038 | 14.068 | 0.124 |
| GmPLD3 | 77.048 | 91.977 | 33.822 | 49.196 | 89.537 | 67.934 | 71.601 | 82.977 | 87.48 |
| GmPLD4 | 18.984 | 38.581 | 15.472 | 25.171 | 34.236 | 22.718 | 33.323 | 38.166 | 38.767 |
| GmPLD5 | 0.881 | 1.339 | 2.045 | 1.985 | 0.564 | 0.078 | 0.432 | 1.743 | 1.254 |
| GmPLD6 | 0.2 | 0.433 | 0.316 | 0.601 | 0.233 | 0.032 | 0.267 | 0.481 | 0.545 |
| GmPLDβ1 | 5.652 | 2.425 | 2.561 | 1.785 | 4.242 | 4.461 | 1.137 | 2.962 | 3.829 |
| GmPLDβ2 | 5.713 | 2.495 | 4.169 | 4.112 | 3.747 | 1.409 | 1.745 | 4.741 | 3.812 |
| GmPLDβ3 | 0.311 | 0.525 | 2.308 | 0.743 | 1.23 | 0.6 | 0.546 | 1.707 | 0.704 |
| GmPLDβ4 | 6.408 | 5.72 | 12.378 | 5.773 | 6.834 | 8.236 | 5.64 | 10.237 | 8.45 |
| GmPLDβ5 | 0.795 | 3.067 | 44.344 | 6.486 | 12.994 | 0.458 | 2.53 | 2.335 | 3.864 |
| GmPLDδ1 | 14.149 | 5.752 | 4 | 6.346 | 12.195 | 13.592 | 8.99 | 13.215 | 3.962 |
| GmPLDδ2 | 27.94 | 7.516 | 3.803 | 10.645 | 8.36 | 9.378 | 6.507 | 12.249 | 9.158 |
| GmPLDδ3 | 2.687 | 2.846 | 0.057 | 1.308 | 1.923 | 0.577 | 1.953 | 1.163 | 1.868 |
| GmPLDδ4 | 0.228 | 1.207 | 0.078 | 0.39 | 1.552 | 0.499 | 2.086 | 0.765 | 9.402 |
| GmPLDδ5 | 0.013 | 0 | 1.303 | 0.006 | 0 | 0 | 0.035 | 0.012 | 0.093 |
| GmPLDδ6 | 0.89 | 0.2 | 1.376 | 0.5 | 0.334 | 1.263 | 0.62 | 0.653 | 2.416 |
| GmPLDε1 | 0.123 | 0.307 | 0.277 | 1.007 | 0.205 | 0.058 | 0.652 | 0.263 | 5.008 |
| GmPLDε2 | 0.254 | 0.425 | 1.427 | 1.598 | 0.151 | 0.289 | 1.006 | 2.634 | 1.532 |
| GmPLDε3 | 3.547 | 3.109 | 5.736 | 3.458 | 4.958 | 3.576 | 2.186 | 3.592 | 10.852 |
| GmPLDζ1 | 2.669 | 0.637 | 4.201 | 1.003 | 0.636 | 2.253 | 0.563 | 0.666 | 22.427 |
| GmPLDζ2 | 5.791 | 1.857 | 7.384 | 2.079 | 2.75 | 3.101 | 1.476 | 2.246 | 8.211 |
| GmPLDζ3 | 3.547 | 3.109 | 5.736 | 3.458 | 4.958 | 3.576 | 2.186 | 3.592 | 10.852 |

**Table S4. Relative expression of *GmPLDɑs* in different developing seeds after fertilization. DAF, day after fertilization. Data are retrieved from https://www.soybase.org/soyseq/heatmap/index.php.**

|  | seed 10DAF | seed 14DAF | seed 21DAF | seed 25DAF | seed 28DAF | seed 35DAF | seed 42DAF |
| --- | --- | --- | --- | --- | --- | --- | --- |
| GmPLDα1 | 3 | 6 | 10 | 5 | 4 | 4 | 4 |
| GmPLDα2 | 0 | 0 | 0 | 0 | 0 | 0 | 0 |
| GmPLDα3 | 10 | 21 | 34 | 19 | 13 | 17 | 12 |
| GmPLDα4 | 3 | 7 | 10 | 6 | 3 | 5 | 2 |
| GmPLDα5 | 0 | 0 | 0 | 0 | 0 | 0 | 0 |
| GmPLDα6 | 0 | 0 | 0 | 0 | 0 | 0 | 0 |
| GmPLDβ1 | 0 | 0 | 0 | 1 | 1 | 2 | 1 |
| GmPLDβ2 | 0 | 0 | 0 | 0 | 0 | 1 | 1 |
| GmPLDβ3 | 0 | 0 | 0 | 1 | 0 | 1 | 0 |
| GmPLDβ4 | 1 | 3 | 3 | 3 | 2 | 3 | 2 |
| GmPLDβ5 | 0 | 0 | 0 | 0 | 0 | 0 | 0 |
| GmPLDδ1 | 1 | 3 | 2 | 4 | 3 | 7 | 7 |
| GmPLDδ2 | 3 | 5 | 4 | 5 | 2 | 5 | 4 |
| GmPLDδ3 | 1 | 0 | 0 | 0 | 0 | 0 | 0 |
| GmPLDδ4 | 0 | 0 | 0 | 0 | 0 | 0 | 0 |
| GmPLDδ5 | 0 | 0 | 0 | 0 | 0 | 0 | 0 |
| GmPLDδ6 | 0 | 0 | 2 | 1 | 1 | 1 | 0 |
| GmPLDε1 | 1 | 1 | 1 | 1 | 0 | 1 | 1 |
| GmPLDε2 | 0 | 0 | 0 | 0 | 0 | 0 | 0 |
| GmPLDε3 | 0 | 0 | 0 | 0 | 0 | 0 | 0 |
| GmPLDζ1 | 0 | 0 | 0 | 1 | 0 | 0 | 0 |
| GmPLDζ2 | 0 | 0 | 0 | 0 | 0 | 0 | 0 |
| GmPLDζ3 | 0 | 0 | 1 | 1 | 0 | 1 | 1 |

**Supplemental Data S1. Protein s**equences for **phylogenic tree construct**. Sequences were retrieved from [https://phytozome.jgi.doe.gov/pz/portal.html#](https://phytozome.jgi.doe.gov/pz/portal.html) and <https://www.ncbi.nlm.nih.gov/>.

PLD protein sequence for phylogenic tree construct

>GmPLDɑ1 Glyma.13G364900.1

MAQILLHGTLHATIYEVDKLKIGGGNFLTKIVQNIEETVGIGKGVTKLYATIDLEKARVGRTRIIEKEIKNPRWYESFHIYCAHMASNIIFTVKDDNPIGATLIGRAYVPVQEILHGEEIDRWVEILDEHKNPIHGHSKIHVKLQYFDVSKDRNWALGIRSPKFPGVPYTFFSQRRGCKVSLYQDAHVPDNFVPKIQLSGGQTYQAHRCWEDVFDAITKAQHLIYITGWSVYTEISLVRDSRRPKPGGDETLGELLKKKAREGVRVLMLVWDDRTSVPLLKKDGLMATHDQETEEYFRGTEVHCVLCPRNPDDGGSFVQDLEISTMFTHHQKIVVVDGELPSGDSNKRRIVSFVGGIDLCDGRYDTQFHSLFRTLDTAHHDDFHQPNFGGSSIKKGGPREPWHDIHSRLEGPIAWDVLFNFEQRWRKQGGKDLLVPLRDLEDVIIPPSPVTYIDDHETWNVQLFRSIDGGAAFGFPETPEDAARVGLVSGKDNIIDRSIQDAYVNAIRRAKNFIYIENQYFLGSSYDWSADGIKPEAIDALHIIPKELSLKIVSKIEAGERFSVYVVVPMWPEGVPESASVQAILDWQRRTMDMMYKDVVQALRAKGIVENPRNYLTFFCLGNREVKKQGEYEPPERPDPDTDYIRAQEARRFMIYVHAKMMIVDDEYIIVGSANINQRSMDGARDSEIAMGAYQPFHLAARQPARGQIHGFRMSLWYEHLGLLHDSFLHPENEECIKKVNQIADKYWDIYSSESLEHDLPGHLLRYPIGVSNEGVVTELPGFEFFPDTKARVLGDKVDYLPPILTT*

>GmPLDɑ3 Glyma.08G211700.1

MAQILLHGTLHATVFEVDRLNAGGGGGNFFSKLKQNFEETVGIGKGVTKLYATIDLEKARVGRTRIIENEHTNPRWYESFHIYCAHMASNIIFTVKDDNPIGATLIGRAYVPVSEVLDGEEIDRWVEILDEEKNPIQEGSKIHVKLQYFDVTKDRNWARGIRSPKFPGVPYTFFSQRQGCKVSLYQDAHVPDNFVPKIPLAGGKNYEAHRCWEDIFDAITNARHFIYITGWSVYTEISLVRDSRRPKPGGDQTLGELLKKKANEGVKVLMLVWDDRTSVGTEVHCVLCPRNPDDGGSIVQDLQISTMFTHHQKIVVVDGAMPGEGSDRRRIVSFVGGIDLCDGRYDTAFHSLFRTLDTAHHDDFHQPNFPGAAITKGGPREPWHDIHSRLEGPIAWDVLFNFEQRWRKQGGKDVLVPLRELEDVIIPPSPVTFPEDHETWNVQLFRSIDGGAAFGFPETPEDAARAGLISGKDNIIDRSIQDAYINAIRRAKNFIYIENQYFLGSSFAWSADDIKPEDIGALHLIPKELSLKIVSKIEAGERFAVYVVVPMWPEGVPESASVQAILDWQKRTMEMMYKDIIQALRAKGIDEDPRNYLTFFCLGNREVKKPGEYEPSEQPDPDSDYQRAQEARRFMIYVHTKMMIVDDEYIIVGSANINQRSMDGARDSEIAMGAYQPYHLATRQPARGQIHGFRMSLWYEHLGMLHDSFLQPESDECINKVNQVADKYWDLYSNESLEHDLPGHLLRYPIGVASEGDVTELPGFEFFPDTKARILGGKADYLPPILTT*

>GmPLDɑ4 Glyma.07G031100.1

MAQILLHGTLHATIFEVDRLNAGGGGGNFFSKLKQNFEETVGIGKGVTKLYATIDLEKARVGRTRIIENEHTNPRWYESFHIYCAHMASNIVFTVKDDNPIGATLIGRAYVPVSEVLDGEEIDRWVEILDEEKNPIHEGSKIHVKLQYFDVTKDRNWARGIRSPKFPGVPYTFFSQRQGCKVSLYQDAHVPDNFVPKIPLAGGKNYEAHRCWEDIFDAITDAKHFIYITGWSVYTEISLVRDSRRPKPGGDQTLGELLKKKASEGVKVLMLVWDDRTSVGLLKKDGLMATHDEETAQFFDGTEVHCVLCPRNPDDGGSIVQDLQISTMFTHHQKIVVVDGAMPGGGSDRRRIVSFVGGIDLCDGRYDTAFHSLFRTLDTAHHDDFHQPNFPGAVITKGGPREPWHDIHSRLEGPIAWDVLFNFEQRWRKQGGKDVLVPLRELEDVIISPSPVTFLEDHETWNVQLFRSIDGGAAFGFPETPEDAARAGLISGKDNIIDRSIQDAYINAIRRAKNFIYIENQYFLGSSFAWSADDIKPADIGALHLIPKELSLKIVSKIEAGERFAVYVVVPMWPEGVPESASVQAILDWQKRTMEMMYRDIIQALRAKGIEEDPRNYLTFFCLGNREVKKPGEYEPSEQPDPDSDYQRAQEARRFMIYVHTKMMIVDDEYIIVGSANINQRSMDGARDSEIAMGAYQPYHLATRQPARGQIHGFRMSLWYEHLGMLHDSFLQPESEECINKVNQVADKYWDLYSSESLEHDLPGHLLRYPIGIASEGDVTELPGFEFFPDTKARILGGKADYLPPILTT*

>GmPLDɑ2 Glyma.15G008500.1

MTQILLHGTLQVTIYELDNLKAGSGGNILTKLVHNIEETIGIGKGITKLYATIDLEKARVGRTRIIEKEHTNPKWYESFHIYCAHMASSIIFTVKDDNPIGATLIGRAYRQGCKVSLYQDAHVPDNFVPKIPLAGGHTYQPHRCWEDVFDAINNAQHLIYITGWSVYTEITLVRDSRRPKRRRCNTWVLMLVWDDRTSVPLLKEDGLMATHDEDTENYFYDSEVHCVLCPRNPDDGGSVVQDVEIFSMFSHHQKIVVVDSALPNGRSDKRRIVSFVGGIDFCDGRYDTQFHSLFRTLGTVHHDDFHQPNFSHTSIKKGGPREPWHDIHSRLEGPIAWDVLFNFEQRWKKQGGKDLLIPPKDLENVIIPPSVVTYPEDHEAWNVQLFRSIDGGAAFGFPETPEEAARSGLISGKDNIIDRGIQDAYIKAIRRAKNFIYIENQYFLGSCYAWSADGIKPEDIGALHLIPRELSLKIVSKIEAWERFSVYIVVPMWPEGYPEKGTVQAILDWQRRTMDMMYKDVVGALKGKGNEEDPLNYLTFFCLVNRELKKEGEYVPPERPDPHTDYMRAQVSRRFMIYVHAKMMIVDDEYIIIGSANINQRSMDGARDSEVAMAAYQPYHLATKQPARGQIHGFRMSLWYEHLGLLHDSFLHPESEECIEKQDLPGHLLRYPVAISSQGNVTQLQAFEFFPDTKAPILGAKSDYIPPILTT*

>GmPLDɑ5 Glyma.06G068600.1

MPHLLHGRLDVIIYEVDTLPTLNDCNLNLCSKGTSRSVGKRFLSQLKSCFLCQCQCQPEFVETGLYATIDLDKARVGRTKLLNDQSSNPTWDETFHVYCAHLISHVIFTVKQKDPIDATLIGRAYVPVEQVVNGNIVDEWVQILDEDHNPIPSESKIHVKMQFSSVRNDINWSQGIRSPRFQGVPHTFFSQKNGCKVTLYQDAHVSDGFVPWIPLSGGKPYEHRKCWEDIYNAIMDARNFIYITGWSVYSEITLIRDPMKPTTRITLGELLKMKAEEGVKVLMLVWDDRTSVPDFKKDGLMATHDQETADYFKNTKVKCVLCPRNPDDGKSIVQGFETSTMFTHHQKTIVVDTQVAMGQQGQKRTIVSFVGGIDLCDGRYDTQEHPLFSTLDTVHKDDFHQPNFPGASIKKGGPREPWHDIHCKLEGSVAWDVLYNFQQRWEKQVGNQLLFSSSKLDEYFVPRSTVVTTNENETWNVQLFRSIDGGAASGFPPDPEEAAELGLVSGKDNIIDRSIQDAYISAIRRAKNFIYIENQYFLGSSYGWQASDIVVEDIGALHLIPKELSLKIVSKIEAGERFSVYVVIPMWPEGIPESGSVQAILDWQRRTMEMMYADIAKAIQRKRIQANPRDYLTFFCLGNREGKKDMEYTPTEAPEPDTDYARAQKARRFMIYVHAKMMIVDDEYIIIGSANINQRSMDGERDTEIAMGAFQPRHIAYNGAPRGQIYGFRRALWCEHLGDHGDTNIFDNPESVDCIRLVNHLAETNWDIYSKETFDEYREFHHLMRYPIEVTNNGAITILQGLEHFPDTKAKILGSQSVYLRPILTT*

>GmPLDɑ6 Glyma.06G068700.1

MPQLLHGTLKVTIFEVDRLHTGCHLDFCQKGTTHKGKRFLAQVKGCLLCRPEIVGTRLYATIDLDKARVGRTRMIGNQPSHPRWNETFEIYCAHQISKIIFTVKDGNPIGATLIGRASVPVEQVRKGPIVKRWVEILDEEDQRPVPGHAKICVSVQFYDVTDDTTCLWSQGISMPFFGVPRTFFNQREGCNVTLYQDAHVPRGIGVVPYIPISEEKDYMPAMCWEDINKAINEAKYFIYITGWAVYTEITLVRDKDESETLGELLKRKADQGVKVLLLIWNDRTSVPELKDGFMATHDQETAGYFRGTKVQCVLCPRNPDDGRSIVQGLKTSTMFTHHQKSVVVDGHVVGSEKRSVISFIGGIDLCDGRYDTRDHPLFSTLNTMHRNDFHQPTFPNASIDKGGPREPWHDIHCKLEGPIAWDVLYNFEQRWEKQVGKKLLYSLDDLDEILVHPSEAQKSEVGVEETWNVQLFRSIDGGAASGFPQTPKEVSELGLVSGKDNVIERSIQDAYIHAIRRAKNFIYIENQYFLGSSYGWQASGDIVVEDIGALHLIPKEISLKIASKIEAKERFSVYIVIPMWPEGVPSSDSVQAILDWQKRTMEMMYSDITDALKKTGIQARPRDYLTFFCLGKRENKDPGDYTPLEKPEPDSDYGRAQNSRRFMIYVHSKMMIVDDEYIIVGSANINERSMEGARDTEIAMGAFQPRHLASSGRPKGEIYRFRRALWYEHLGDDGFGSKIFDFPEHLECINHVNKLAEANWDMYSMETFVENKRQFHHLMCYPIQVTNDGAITNLPGFEYFPDTKARILGCKSKLIPSILTT*

>GmPLDδ1 Glyma.11G081500.1

MADTGDDNGVTYLHGDLDLKIIEARHLPNMDIFSERLRRCVTACDTIKFHSDAPAAADGDGGSQRTRTHHHRRIITSDPYVTVSVPQATVARTRVLKNAQNPVWKEQFHIPLAHPVVDLEFRVKDDDVFGAQSMGTVKVPARRIATGAKISEWFPVLLPSGKPPKPDTALHVEMQFTPVSENLLYQRGIAADPEHNGVRHTYFPVRKGSSVRLYQDAHCTESGEGKIPEIKLENGNVYRHGKCWEDICYAISEAHHMVYLVGWSIYHKVRLVREPTRPLPRGGDLTLGELLKYKSEEGVRVLLLVWDDKTSHDKVFLKTAGVMGTHDEETRKFFKHSSVMCVLSPRYASSKMSFLKQQVVGTVFTHHQKCVIVDTQAAGNNRKITAFIGGLDLCDGRYDTPEHRLFRNLDDVFDGDFHNPTFPAGTRVPRQPWHDLHCRIDGPAAYDVLINFEQRWRKATKWKEFAILFKKTSQWHDDALIRIERISWILSPSGAATLKDKSDYYTVPEDDPLVWVSSEDDPENWHVQIFRSIDSGSLKGFPKRVDIALSQNLICAKNLVIDKSIQTAYIQAIRSAQHFIYIENQYFIGSSYAWPAYKDAGADNLIPMELALKIASKIRAKERFAVYIILPMWPEGDPKTGAMQEILFWQGQTMQMMYDVVARELKSMQLTDVHPQEYLNFYCLGNREHFNEDSSSTNGAQVSTAYKYRRFMIYVHAKGMIVDDEYVIIGSANINQRSMAGTKDTEIAMGAYQPHYTWSAKKRHPHGQIYGYRMSLWGEHLGMLDETFEEPGRLECVEKVNEIAENNWKLFASEDFSLLQGHLLKYPVQVDSDGKIRSLPDCENFPDAGGKILGAHSTTIPDILTT*

>GmPLDβ5 Glyma.01G215100.1

MAHLVYGETPSFGASHHGQAQQIVPFQTTSSSLRILLLHGNLEIWVNEARNLPNMDMFHKKTGEMVSMLSRKLGGKIEGHMSKAGTSDPYVTVSVAGAVIARTFVIRNSENPVWTQHFNVPVAHLASEVHFVVKDSDIVGSQIIGAVGIPVEHLCSGTRVEGFFPILGANGKPCKGGSVLSLSIQYTPVEKVPLYSHGVGAGPDYEGVPGTYFPLRKGGKVTLYQDAHVEEGCLPSLKVDGYVNYKHGSCWHDIFDAISEARRLVYIVGWSVYYNVSLIRDSANGKSYTLGDLLKAKSQEGVRVLLLVWDDPTSKSMLGFKTVGLMNTHDEDTRQFFKNSSVRVLLCPRAGGKGHSWVKTQEAGTIYTHHQKTVIVDADAGQNKRKIKAFIGGLDLCVGRYDTPNHSIFRTLQTTHKDDYHNPNFEGPVTGCPRQPWHDLHSQVDGPAAYDILTNFEERWLRALKMHRFQKMKSSHDDSLLKIDRIPDIVGIDEVPCQNENNRETWHAQVFRSIDSNSVKGFPKEPQDAIRRNLVCGKNVLIDMSIHSAYVKAIRAAQKFIYIENQYFLGSSYNWDSYKDLGANNLIPMEIALKIANKIKQHERFSVYIVIPMWPEGVPTSTATQRILFWQFKTMQMMYETIYKALQEAGLDNKYEPQDYLNFFCLGNREIPDNENVLNDVKTTGENKPQALTKKNRRFMIYVHSKGMIVDDEYVLLGSANINQRSMEGTRDTEIAMGAYQPNHTWAKKQSKPHGQVHGYRMSLWSEHIGAVEECFEEPESLECVRRIRSLSEFNWRQYAAEEVTEMKSHLLKYPLEVDSKGKVKPLFGCEAFPDVGGNISGTFTLLKENLTI*

>GmPLDδ2 Glyma.01G162100.1

MADTGGGDDNGVTYLHGDLDLKIIEARHLPNMDIFSERLRRCVTACDTIKFHSEAPAGGGGAGQRKHHHPRIITSDPYVTVSVPQATVARTRVLKNSPNPVWEEQFNIPLAHPVVDLEFRVKDDDVFGAQTMGTVKVPARRIATGAKISEWFPILLPSGKPPKPDTALHVEMKFTPVWENLLYQRGIAADPEHNGVRHTYFPVRKGSSVRLYQDAHCTESGEGKLPEIKLENGNVYRHEKCWEDICYAISEAHHMVYLVGWSIYHKVRLVREPTRPLPRGGDLTLGELLKYKSEEGVRVLLLVWDDKTSHDKVFLKTAGVMGTHDEETRKFFKHSSVMCVLSPRYASNKMSFLKQQVVGTVFTHHQKCVIVDTQAAGNNRKITAFIGGLDLCDGRYDTPEHRLFRNLDDVFDGDFHNPTFSAGTRVPRQPWHDLHCRIDGPAAYDVLINFEQRWRKATKWKEFAILFKKSSQWHDDALIRIERISWILSPSGAATLKDKSDDYTVPEDDPLVWVSSEDDPENWHVQIFRSIDSGSLKGFPKRVDVALSQNLICAKNLVIDKSIQTAYIQAIRSAQHFIYIENQYFIGSSYAWPAYKDAGADNLIPMELALKIASKIRAKERFAVYIVLPMWPEGDPKTGAMQEILFWQGQTMQMMYDAVARELKSMQLTDVHPQDYLNFYCLGNREHFNEDSSSTNGAQVSTAYKYRRFMIYVHAKGMIVDDEYVIIGSANINQRSMAGTKDTEIAMGAYQPHYTWSAKKRHPHGQIYGYRMSLWGEHLGMLDETFEEPERLECVHKVNKIADNNWKLFASEDFSLLQGHLLKYPVQVDSDGKIRSLPDCENFPDAGGKILGAHSTTIPDILTT*

>GmPLDδ3 Glyma.06G020500.1

MPHNPDTVVYLHGTLDLVIEDARFLPNMDMLSERVRRFFSALNTCSASITGKRKQRHARHRHRKIITSDPYVTVCLAGATVARTRVISNSQNPTWDEHFKIPLAHPASQVEFYVKDNDMFGADLIGVATVSAERILSGEAIRDWFPIIGTFGKPPKPDCAVRLAMKFTRCEDSFMYRSRTETDPDRFVVRDSYFPVRHGGSVTLYQDAHVPDSMLPEVELEDGVVFEHGKCWEDICHAILGAHHLVYIVGWSIYHKVKLVREPTKALPSGGNLNLGELLKYKSQEGLRVLLLVWDDKTSHSKFGINTSGVMQTHDEETRKFFKHSSVRCLLSPRYASSKLSIFKQQVVGTLFTHHQKCVIVDTQAHGNNRKITAFIGGLDLCDGRYDTPEHRILRDIDTVYQDDYHNPTFCAGTKGPRQPWHDLHCKIEGPAAYDILTNFEQRWRKATKWSELGRKLKRVSHWNDDSLIKLERIFWILSPSESTPVDDPELWVSKEDDPENWHVQVFRSIDSGSLKGFPKDVVVAETQNLVCAKNLVIDKSIQTAYIHAIRSAQHFIYIENQYFIGSSFAWPAYKEAGADNLIPVELALKIVSKIRSKERFTVYIVIPMWPEGSPSSTSVQEILFWQGQTMKMMYEIIALELKSMQLDSHPQDYLNFYCLGNREQLTTEVSSSSNSPSDNGETVSASQKFRRFMIYVHAKGMIVDDEYVILGSANINQRSLAGSRDTEIAMGAHQPHHTWSQKKRHPHGQVYGYRMSLWAEHMETIEACFKEPESLECVKSVNKIAEDNWKKYTADDYTPLQGHIMKYPVCVNAYGKVKSLTGFESFPDVGGKVLGSRSTLPDALTT*

>GmPLDβ1 Glyma.02G093500.1

MDNYGSSSPYRYPSPYMYPPNPHQPYPPPPGSAPDPYAQHVPYQPYPYLSSHSFNYSYPPPPRPSSHSGHFEYSYTPPPHPSDFPYPPPPYYAHPPSYPYPYHVPPPNHDPSKPSLSYHASFQHGSSHYYYQQPNQAYSASAPEVQPDIHSHTNSYSGPYWQENTSTAADEVSQASDSSKPSQGSAYPPLDDLMSNVRLSDGQPTAPASPPAPARQPFMHSISVPKLQQKREEFYGYSNNSFSGWGSSYHSQVDSSRLSDFSGSFNESMHSQSLQIVPVQNKGSLRVLLLHGNLDIWIHEAKNLPNMDMFHKTLGDMFGKLPGSVGNKIEGTMNKKITSDPYVSISVSNAVIGRTYVISNSENPVWLQHFYVPVAYHAAEVHFLVKDNDIVGSQLIGIVAIPVEQIYSGAVVEGTFPILNNNGKPCKQGAVLTLSIQYIPMEKLSIYHQGVGAGPEYIGVPGTYFPLRRGGTVTLYQDAHVPDGSLPNVLLDSGMYYVNGKCWQDIFDSISQARRLIYITGWSVWHKVRLVRDAAGYASDYTLGDLVKSKSQEGVRVLLLIWDDPTSRSIFGYKTDGVMATHDEETRRFFKHSSVQVLLCPRSGKRHSWIKQKEVGTIYTHHQKTVIVDADAGNNRRKIIAFVGGLDLCDGRYDTPHHPLFRTLNTIHKDDYHNPTFTGNIGGCPREPWHDLHSKIDGPAAYDVLTNFEERWLKASKPHGIKKLKISYDDALLRLERIPDVIGINDAPSVGEDNPEVWHVQIFRSIDSNSVKGFPKDPKDATSKNLVCGKNVLIDMSIHTAYVKAIRAAQHYIYIENQYFIGSSYNWSQHKDLGANNLIPMEIALKIAEKIKANERFAVYVVIPMWPEGVPTGAATQRILFWQNKTMQMMYETIYKALVEAGLEAAFSPQDYLNFFCLGNREAMNLYDNAGVTGAPPPANSPQAASRNSQRFMIYVHSKGMIVDDEYVILGSANINQRSMEGTRDSEIAMGAYQPHHTWARKQSYPHGQIHGYRMSLWAEHTGTIEECFLQPESLECVRRVKAMGEMNWKQFSAKEATEMKGHLLKYPVEVDRNGKVRPLQDCEEFPDVGGKIVGSFLAMKENLTI*

>GmPLDβ4 Glyma.07G080400.1

MDNYGSSQHPYGYPNPYSYPPHDPYAQPPYPYPYPYPYPYPHPTNPPHNNNSNYPYPPPPPPPHSSSPYSSPINYSSYPPPPPPPPPPPSTSSHGSFDYPMPQPPHTYPYPHRVPPEWSVTTGGGVSHSHASDSYNPPHSAAYPTLDDLMSNDRLSDNNNLPSAPPLTHSPPILYLDRRDEFYGYSSYSSSSLDQGDPSRLSDNNDDSVNSESLQIVPAQHKGSLRVLLLHGNLDIWVHGAKNLPNMDMFHKTLEDMIGRFPGTVASNKIEGTVSRKITSDPYVTISVSNAVIGRTFVISNSENPVWEQHFYVPVAHHAAEVHFVVKDSDVVGSQLIGVVAIPVEKIYSGQKVQGTYPILNSNGKPCKPGAVLMVSIQYIPMHTLIMYYQGVGAGPDYIGVPGTYFPLRKGGTVTLYQDAHVPDGCLPNVVLDNGVYYAHGKCWLDIFDAINRAKRLIYITGWSVWHKVRLVRDPGNPSKFTLGDILRSKSSEGVRVLLLIWDDPTSRSILGYKVDGVMATHDEETRRFFKHSSVHVLLCPRIAAKRHSWAKQKEVGTIYTHHQKTVIVDADAGNNQRKIVAFVGGLDLCDGRYDTPHHPLFRTLQTLHKDDYHNPTFTGNTGGCPREPWHDLHSKIDGPAAYDILKNFEERWLRAAKPKGIQKLRSSYDDALLKLDRIGDIISSSNAPSVGDDNPESWHVQIFRSIDSSSVKGFPKEPKDASSMNLVCGKNVLIDMSIHTAYVKAIRAAQHYIYIENQYFIGSSYNWSQHKDLGANNLIPMEIALKIAAKIRANERFAVYIVIPMWPEGVPTGAATQRILFWQHKTMQMMYETIYKALVEVGLETAFSPQDYLNFFCLGNREAIDMYENITVSGTPPPANSPQAFSRNNRRFMIYVHSKGMIVDDEYVILGSANINQRSMEGTRDTEIAMGAYQPHHTWARSQYHPRGQIHGYRMSLWAEHTGTIEDCFLQPESLECVSRIRTMGELNWKQFASNDVTEMTGHLLKYPVEVDRKGKVRSLPGHEEFPDVGGKIVGSFIAIQENLTI*

>GmPLDβ2 Glyma.18G288600.1

MDNYGSSSPYRYPNQYMYPPNPHQPYPPPPGSAPDPYAQHVPYQPYPYLSSHSFNYSYPPPPRSSSHSGHFEYSYPPPHPPPSYANPPYPYPYHVPPPNHDPPKPSLSHHASFQHEPSHYYYQQPNDAYSASAPQVHPDVHLRTNSFSGPYWHENTSTAGDEVSQTSDNSKPSQGSAYPSLDDLMSNVRLSDDQPTAPASPPAPAGQPFMHSISVPKLQQKREEFYGYSNNSFSGWGSSYHSRVDSSRLSDFSGSFNESVHSQSLQIVPVQNKGSLRVLLLHGNLDIWVHEAKNLPNMDMFHKTLGDMFGKLPGSVGNKIEGTMNKKITSDPYVSISVSNAVIGRTYVISNSENPVWLQHFYVPVAYHAAEVHFLVKDSDIVGSQLIGIVAIPVEKIYSGEVVEGTFPILNNNGKPCKQGAVLTLSIQYIPMEKLSIYHQGVGAGPEYIGVPGTYFPLRRGGTVTLYQDAHVPDGSLPNVLLDSGMYYVNGKCWQDIFDSISQARRLIYITGWSVWHKVRLVRDAAGYASDYTLGDLLRSKSQEGVRVLLLIWDDPTSRSILGYKTDGVMATHDEETRRFFKHSSVQVLLCPRSGKRHSWIKQKEVGTIYTHHQKTVIVDADAGNNRRKIIAFVGGLDLCDGRYDTPHHPLFRTLNTIHKDDYHNPTFTGNAGGCPREPWHDLHSKIDGPAAYDVLTNFEERWLKASKPHGIKKLKISDDDALLRLERIPDVIGINDAPSVGEDDPEVWHAQIFRSIDSNSVKRFPKDPKDATSKNLVCGKNVLIDMSIHTAYVKTIRAAQHYIYIENQYFIGSSYNWSQHKDLGANNLIPMEIALKIAEKIKANERFAVYVVIPMWPEGVPTGAATQRILFWQNKTMQMMYETIYKALVEAGLEAAFSPQDYLNFFCLGNREAGNLYDNVSMTGAPPPANSPQAASRNSQRFMIYVHSKGMIVDDEYVILGSANINQRSMEGTRDSEIAMGAYQPHHTWARKQSYPHGQIHGYRMSLWAEHTGTIEECFLKPESLECVRRVRAMGEMNWKQFSANEATEMKGHLMKYPVEVDRKGKVRPLQDCEEFPDVGGKIVGSFLAMKENLTI*

>GmPLDβ3 Glyma.03G018900.1

MDNYGSLQHPYGYPNPPHDLYAQPPYPYPYPPNPPYTNNFNYSYPPPPPPPHSSSPYSSHIDYSSYPPPPSTSSHGSFDYPMPQPPHTYLYPNEFYSYSSNSSSSLDQEDPSRVSDNSDDSVHSQSLQIVPAQLKGSLRVMLLHGNLDIWVHGATNLPNMDMFHKTLEDMFGRLPGNVQHFYNVPVAHHAAEVHFVVKDSDVVGSQLIGVVAIPVEQIYSGNGKPCKPGAVLTVSIQYIPMEKLTIKGGTVTLYQDAHVPNGCLPNVVLENGMYCAHGQSKSSEEGVRVLLLVWDDPTSRNILGYKVDGVMATRDEETRRFFKHSSMQVLLCPRIAGKRNSWVKQQFYNFSSGTIYTHHQKTVIVDADAGNNKRKIVAFVGGLDLCDGRYDTPHHPMFRTLQTLHKDDYHNPTFTGNTGGCPREPWHDLHSKIDGPAAYDILKNFEERWLRAAKPKGIQKIRGIISASNAPSVGDDNPESWHVQIFRSIDSNSVKGFPKEPKNASSMNLVCGKNVLIDMSIHTAYVKAIRVAQHYIYIENQYFIGSSYNWSQHKDLGANNLIPMEIALKIAAKIRANERFAGVPTGAATQRILFWQHKTMQMMYETIYKALVEVGLEAAFSPQDYLIFFCLGNREAIDMYENITVSGTPPPANTVIISILIAFSRTNPRFMIYVHSKGMIVDDEYVILGSANINQRSIEGTRDTEIAMGAYQPHHTWARRQYYPRGQGKVRSLPGHEEFPDVGGKIVGSFIAIQENLTI*

>GmPLDζ3 Glyma.09G041400.1

MATEQLMSSAGGGSSRYVQMKSSPPPSPPAAAAEEISSVPSFRHSGAEANRIFEELPKAAIVSVSRPDASDISPMQLSYTIQVQYKQFKWELTKKAHQVFILHFALKKRAFIEEIHEKQEQVKEWLQNLGIGEHTAMAQDDDEGDDETVPLHTEETHESAKDRDVPSSAALPIIRPALGRQHSIADRAKRAMQGYLNHFLGNISIVNSREVCKFLEVSKLSFSPEYGPKLKEEYVMVKHLPKIQKDDDSRKCCLSDCFSCCNDNWQKVWAVLKPGFLALLADPFDTQPLDIIVFDVLPASDGNGDGRLSLASEMKERNPLRHSFKVTCGIRSIRIRVKSSSKVKDWVAAINDAGLRPPEGWCHPHRYGSFAPPRGLVEDGSQAQWFIDGRAAFEAIAFSIEAAKSEIFICGWWLCPELYLRRPFHTHASSRLDNLLEAKAKQGVQIYILLYKEVALALKINSVYSKKKLLSIHENVRVLRYPDHFSTGVYLWSHHEKLVIIDNHICFIGGLDLCFGRYDTSEHKVGDFPPLTWPGKDYYNPRESEPNSWEDTMKDELEREKYPRMPWHDVHCALWGPPCRDIARHFVQRWNYAKRNKAPYEQAIPLLMPQHHMVIPHYLGRSREIQIESRNTDNHRVLKREDSFSSSSQDQDIPLLLPQEPDGLDTHEGDQKLNGVISFSHHLDKPRRISSGLPFSFRKAKIVAVGPDTPMKGFVDDLDSEHGLEKMSLDRVAHFDLQSTKPQWWETQERGDQGGFAEESGQVGPLASCRCQVIRSVSQWSAGTSQTEESIHNAYCSLIEKAEYFIYIENQFFISGLSGDEMIRNRVLEALYRRIMRAYNDKKSFRVIVVIPLLPGFQGGLDDSGAASVRAIMHWQYRTICRGQNSIMHNLYELLGSKIHDYISFYGLRSYGRLSNGGPVATSQVYVHSKIMIVDDCITLIGSANINDRSLLGSRDSEIGIVLEDREFIGSYMDGKPWKAGKFSLTLRLSLWSEHLGLPIGEVNQIMDPVVESTYRDIWMATAKTNTTIYQDVFSCVPNDLIHTRLAFRQSVAFWKERIGHTTIDLGIAPEKLESYYDGGITNTDPLERLASVKGHLVSFPLEFMCQESLRPAFNESEYYATQVFH*

>GmPLDζ2 Glyma.15G152100.1

MATEQLMSGGGSRYVQMKSSPPSSPPAAAAEEMSSVPSFRHSGAEANRIFEELPKASIVSVSRPDASDISPMQLSYTIQVQYKQFKWELTKKAHQVFILHFSLKKRAFIEEIHEKQEQVKEWLQNLGIGEHTAMVQDDDEGDDETVPLHTEETHESAKDRDVPSSAALPIIRPALGRQHSIADRAKRAMQGYLNHFLGNISIVNSHEVCKFLEVSKLSFSPEYGPKLKEEYVMVKHLPKIQKDDDSRKCCLSDCFSCCNDNWQKVWAVLKPGFLALLADPFDTQPLDIIVFDVLPASDGNGDGRLSLASEMKERNPLRHSFKVTCGIRSIRIRVKSSSKVKDWVAAINDAGLRPPEGWCHPHRYGSFAPPRGLVEDGSQAQWFIDGRAAFEAIASSIEAAKSEIFICGWWLCPELYLRRPFHTHASSRLDNLLEAKAKQGVQIYILLYKEVALALKINSVYSKKKLLSIHENVRVLRYPDHFSTGVYLWSHHEKLVIIDNHICFIGGLDLCFGRYDTSEHKVGDFPPLIWPGKDYYNPRESEPNSWEDTMKDELEREKYPRMPWHDVHCALWGPPCRDIARHFVQRWNYAKRNKAPYEQAIPLLMPQHHMVIPHYLGRSREIQIASRNIDNHRVLKREDSFSSSSQDQDIPLLLPQESDGLDTHEGDQKLNGVISFSHQLDKPRRISSGLPFSFRKAKIVAVGPDTPMKGFVDDLDSEHDREKMSLDRVAHIDLQSTNPEWWETQERGDQGGFAEESGQVGPLASCRCQVIRSVSQWSAGTSQTEESIHNAYCSLIEKAEYFIYIENQFFISGLSGDEMIRNRVLEALYRRIMRAYNDKKSFRVIVVIPLLPGFQGGLDDSGAASVRAIMHWQYRTICRGQNSILHNLYELLGSKIHDYISFYGLRSYGRLSNGGPVATSQVYVHSKIMIVDDCITLIGSANINDRSLLGSRDSEIGIVLEDREFIGSYMDGKPWKAGKFSLTLRLSLWSEHLGLPIGEVNQIMDPVVESTYRDIWMATAKTNTTIYQDVFSCVPNDLIHTRFSFRQSVAFWKERIGHTTIDLGIAPEKLESYHDGGIKNTDPLERLASLKGHLVSFPLEFMCQESLRPAFNESEYYATQVFH*

>GmPLDζ1 Glyma.20G238000.1

MYGKETDLGPFCRRFEEGAEQFRLDVAQNPNDTKEFIWCFLCEAQLFGVDEARKRYLEVGRDPWPVMREAYNMFIDGGDPEKLVAAFSGSREGEYFYASLYAGLYYESEFKWRLVKKASQLLYLQFCLRKRALIEDFHDKQEQLKEWLHNLGIVDQTVMVQDDEEPDDGAVPLHHEDSVKNRYVPSVAALSIIRPSIGGQQTIADRAKVAMQGYLNRFLGNLDIVNSQEVCRFLEVSRLSFLQEYGPKLKEGYVMVKHLSNISQDSDVSCFPCNWFHCCNNSWKKVWSVLKPGFLAFLDDPFNNKPLDIMIFDILPYSNGDGGTKIFLADPVKERNPLRYTFKVTSGNRSILLRTTSSAKVKAWVTAINEASLRPLEGWCCPHRFGSFAPIRGLTEDGSQAQWFVDGQAAFEAIATSIQDAKSEIFITGWWLCPELYLRRPFDSFSTSRLDSLLEEKANQGVQIYVLLYKEVSLALKINSLYSMRRLFKIHENVRVLRYPDHFAARVYLWSHHEKLVIIDYKICYIGGLDLCFGRYDTPEHKVGDCPSVIWPGKDYYNPRESEPNSWEDTMKDELDRKKYPRMPWHDVHCALWGPPCRDIARHFVQRWNHAKRTKAPNEHGIPLLMPHHHMVLPHYMGRSKEIDIDEKKDKDKRKGIGRQDSFSSESPMQDIPLLLPQEADGLDTSNGDHTNLSENFPLSQKLEHETLVSDTQMKGFQDEVVPLNLGAQPVANALDDWWETPEETNDDITLEYGEVGPRTTCHCQVIRSVSQWSAGTSQPEESIHTAYCSLIEKAKHFIYIENQFFISGLAGDDIILNRVLEALYRRILQAHKDQKDFRVIIVMPLLPGFQGGLDDGGAATVRALTHWQYRTISRENHSILDNLEAILGPKTQDYISFYGLRSHGRLYENGPVATSQVYVHSKLMIIDDRIAFIGSSNINDRSLLGLRDSEIGVLIEDKEYVDSLMNGKPWKAGKFSYSLRCSLWSEHLGLHAGEISKISDPVADTTYKDLWSATAKENTRIYHEVFACIPNNQIHSRAALRQSMVHWKEKLGHTTIDMGIAPDKLVCHENGEIKIIDPIDRLKSVKGHLVSFPLEFMREEDLRPAVIESEFYVAPQVYH*

>OsPLDalpha1-Q43007

MAQMLLHGTLHATIFEAASLSNPHRASGSAPKFIRKFVEGIEDTVGVGKGATKVYSTIDLEKARVGRTRMITNEPINPRWYESFHIYCAHMASNVIFTVKIDNPIGATNIGRAYLPVQELLNGEEIDRWLDICDNNREPVGESKIHVKLQYFDVSKDRNWARGVRSTKYPGVPYTFFSQRQGCKVTLYQDAHVPDNFIPKIPLADGKNYEPHRCWEDIFDAISNAQHLIYITGWSVYTEITLVRDSNRPKPGGDVTLGELLKKKASEGVRVLMLVWDDRTSVGLLKRDGLMATHDEETENYFHGSDVNCVLCPRNPDDSGSIVQDLSISTMFTHHQKIVVVDHELPNQGSQQRRIVSFVGGLDLCDGRYDTQYHSLFRTLDSTHHDDFHQPNFATASIKKGGPREPWHDIHSRLEGPIAWDVLYNFEQRWRKQGGKDLLLQLRDLSDTIIPPSPVMFPEDRETWNVQLFRSIDGGAAFGFPDTPEEAAKAGLVSGKDQIIDRSIQDAYIHAIRRAKNFIYIENQYFLGSSYAWKPEGIKPEDIGALHLIPKELALKVVSKIEAGERFTVYVVVPMWPEGVPESGSVQAILDWQRRTMEMMYTDITEALQAKGIEANPKDYLTFFCLGNREVKQAGEYQPEEQPEADTDYSRAQEARRFMIYVHTKMMIVDDEYIIIGSANINQRSMDGARDSEIAMGGYQPYHLATRQPARGQIHGFRMALWYEHLGMLDDVFQRPESLECVQKVNRIAEKYWDMYSSDDLQQDLPGHLLSYPIGVASDGVVTELPGMEYFPDTRARVLGAKSDYMPPILTS

>AtPLDalpha1

MAQHLLHGTLHATIYEVDALHGGGVRQGFLGKILANVEETIGVGKGETQLYATIDLQKARVGRTRKIKNEPKNPKWYESFHIYCAHLASDIIFTVKDDNPIGATLIGRAYIPVDQVINGEEVDQWVEILDNDRNPIQGGSKIHVKLQYFHVEEDRNWNMGIKSAKFPGVPYTFFSQRQGCKVSLYQDAHIPDNFVPRIPLAGGKNYEPQRCWEDIFDAISNAKHLIYITGWSVYAEIALVRDSRRPKPGGDVTIGELLKKKASEGVRVLLLVWDDRTSVDVLKKDGLMATHDEETENFFRGSDVHCILCPRNPDDGGSIVQSLQISTMFTHHQKIVVVDSEMPSRGGSEMRRIVSFVGGIDLCDGRYDTPFHSLFRTLDTVHHDDFHQPNFTGAAITKGGPREPWHDIHSRLEGPIAWDVMYNFEQRWSKQGGKDILVKLRDLSDIIITPSPVMFQEDHDVWNVQLFRSIDGGAAAGFPESPEAAAEAGLVSGKDNIIDRSIQDAYIHAIRRAKDFIYVENQYFLGSSFAWAADGITPEDINALHLIPKELSLKIVSKIEKGEKFRVYVVVPMWPEGLPESGSVQAILDWQRRTMEMMYKDVIQALRAQGLEEDPRNYLTFFCLGNREVKKDGEYEPAEKPDPDTDYMRAQEARRFMIYVHTKMMIVDDEYIIIGSANINQRSMDGARDSEIAMGGYQPHHLSHRQPARGQIHGFRMSLWYEHLGMLDETFLDPSSLECIEKVNRISDKYWDFYSSESLEHDLPGHLLRYPIGVASEGDITELPGFEFFPDTKARILGTKSDYLPPILTT

>AtPLDgamma1

MAYHPAYTETMSMGGGSSHGGGQQYVPFATSSGSLRVELLHGNLDIWVKEAKHLPNMDGFHNRLGGMLSG

LGRKKVEGEKSSKITSDPYVTVSISGAVIGRTFVISNSENPVWMQHFDVPVAHSAAEVHFVVKDSDIIGS

QIMGAVGIPTEQLCSGNRIEGLFPILNSSGKPCKQGAVLGLSIQYTPMERMRLYQMGVGSGNECVGVPGT

YFPLRKGGRVTLYQDAHVDDGTLPSVHLDGGIQYRHGKCWEDMADAIRQARRLIYITGWSVFHPVRLVRR

TNDPTEGTLGELLKVKSQEGVRVLVLVWDDPTSRSLLGFKTQGVMNTSDEETRRFFKHSSVQVLLCPRSG

GKGHSFIKKSEVGTIYTHHQKTVIVDAEAAQNRRKIVAFVGGLDLCNGRFDTPKHPLFRTLKTLHKDDFH

NPNFVTTADDGPREPWHDLHSKIDGPAAYDVLANFEERWMKASKPRGIGKLKSSSDDSLLRIDRIPDIVG

LSEASSANDNDPESWHVQVFRSIDSSSVKGFPKDPKEATGRNLLCGKNILIDMSIHAAYVKAIRSAQHFI

YIENQYFLGSSFNWDSNKDLGANNLIPMEIALKIANKIRAREKFAAYIVIPMWPEGAPTSNPIQRILYWQ

HKTMQMMYQTIYKALVEVGLDSQFEPQDFLNFFCLGTREVPVGTVSVYNSPRKPPQPNANANAAQVQALK

SRRFMIYVHSKGMVVDDEFVLIGSANINQRSLEGTRDTEIAMGGYQPHYSWAMKGSRPHGQIFGYRMSLW

AEHLGFLEQGFEEPENMECVRRVRQLSELNWRQYAAEEVTEMSGHLLKYPVQVDRTGKVSSLPGCETFPD

LGGKIIGSFLALQENLTI

>AtPLDbeta1

MDNHGPRYPYPYGQYPYPYPYPAPYRPPSSEPYPPPPTNQYSAPYYPYPPPPYATPPPYASPPPPHQHTS

GSHSGPLDYSHNPQPSSLAAAPPEYHRHSFDYQPSPYPYQPQGNFGAYGPPPPHYSYQEPAQYPPPETKP

QEPLPPPQQTQGFQEYRRQDCLSTGGTGHDNVSNSGSSYPPVDELLGGLHISTNQPGPSVPQLSSLPSNS

WQSRPGDLYGYPNSSFPSNSHLPQLGRVDSSSSYYASTESPHSADMQMTLFGKGSLKVLLLHGNLDIWIY

HAKNLPNMDMFHKTLGDMFGRLPGKIEGQLTSKITSDPYVSVSVAGAVIGRTYVMSNSENPVWMQHFYVP

VAHHAAEVHFVVKDSDVVGSQLIGLVTIPVEQIYSGAKIEGTYPILNSNGKPCKPGANLSLSIQYTPMDK

LSVYHHGVGAGPDYQGVPGTYFPLRKGGTVRLYQDAHVPEGMLPGIRLDNGMSYEHGKCWHDMFDAIRQA

RRLIYITGWSVWHKVKLIRDKLGPASECTLGELLRSKSQEGVRVLLLIWDDPTSRSILGYKTDGVMATHD

EETRRFFKHSSVQVLLCPRNAGKRHSWVKQREVGTIYTHHQKNVIVDADAGGNRRKIIAFVGGLDLCDGR

YDTPQHPLFRTLQTIHKDDFHNPTFTGNLSGCPREPWHDLHSKIDGPAAYDVLTNFEERWLKAAKPSGIK

KFKTSYDDALLRIDRIPDILGVSDTPTVSENDPEAWHVQIFRSIDSNSVKGFPKDPKDATCKNLVCGKNV

LIDMSIHTAYVKAIRAAQHFIYIENQYFIGSSYNWNAHKDIGANNLIPMEIALKIAEKIRANERFAAYIV

IPMWPEGVPTGAATQRILYWQHKTIQMMYETIYKALVETGLEGAFSPQDYLNFFCLGNREMVDGIDNSGT

GSPSNANTPQALSRKSRRFMVYVHSKGMVVDDEYVVIGSANINQRSMEGTRDTEIAMGAYQPQHTWARKH

SGPRGQIYGYRMSLWAEHMATLDDCFTQPESIECVRKVRTMGERNWKQFAAEEVSDMRGHLLKYPVEVDR

KGKVRPLPGSETFPDVGGNIVGSFIAIQENLTI

>OsPLDbeta1-BAF27022

MEGGNHGGGGYPYPPPQQYPYPYGQYPYQYPPPQQQPPPPSAYLSPSRSFHGYPSAPPPQPQPQPYAHHS

APLQPYPPPPQHHAYPPPQPHPPSPYVYDPYHAPAAAYPSYPSPNPSPSISPSSSFHHHPEPPSPSPSAP

SYPSIADGLANMHVSDRHDYPPPPSPAAVPAASSPSVLPPSASFPGGGSSHGGGGMQMVPYGPPAGGSQH

GGMQMVAYGSPAGGSQHGSVRPSLKVVLLHGTLDVWVYDARNLPNKDLFSKRVGDLLGPRLIGAVGSKMS

SANMTSDPYVTIQVSYATVARTYVVPNNENPVWTQNFLVPVGHDAAEVEFVVKDNDVFGAQLIGTVSIPA

EKLLFGERINGIYDVLESNGKPCAQGAVLRLSIQYIPVAQLKMYHHGVIAGPDSLGVPNTYFPMRRGNRV

TLYQDAHVPDGCLPDFCLDHGMRYQHGQCWRDIYDAICQARRLIYIVGWSVFHTIHLIREGVEKMPSLGE

LLKMKSQEGVRVLLLVWDDPTSRSILGIKTDGFMGTRDEETRRFFKHSSVQVLLCPRSAGKRHSWVKQQE

TGTIFTHHQKTVILDADAGNHKRKIIAFVGGLDLCGGRYDTPSHPLFRSLQTVHKEDYYNPNFATVDARG

PREPWHDLHSKIDGPAAYDVLQNFQERWLKASKRHGIKKLGKSYDDALLSIERIPDFISINDAIYFSDND

PETWHVQVFRSIDSNSAKGFPKDPREATRKNLVCGKNVLIDMSIHTAYVNAIRGAQHFIYIENQYFIGSS

FNWDSNKDIGANNLIPIEIALKIANKIKAKERFSAYIVIPMWPEGNPTGAPTQRILYWQHKTMQMMYETI

YRALKEEGLDDLYEPQDYLNFFCLGNREVADSPSTSNSTSTPQEQARKHRRFMVYVHSKGMIVDDEYVII

GSANINQRSMEGIRDTEIAMGAYQPQYTWASKVSAPRGQIYGYRMSLWAEHIGVVEEGFNYPETMECMRR

VRQIGEQNWERFVDNEVTEMRGHLMKYPVSVDRKGKVKPLPGCTSFPDMGGNICGSFRAIQENLTI

>OsPLDdelta1-BAF25740

MESSAGGGGGESPPPAKPVLLHGDLDLWVVEARLLPNMDMFSEHVRRCFAACKPPTSCATARQPRHARGH

HRRKIITSDPYVTLSVAGAVVARTRVIPNDQDPVWDERFAVPLAHYAAALEFHVKDNDTFGAQLIGTVTI

PADRVASCQEVEDWFPIIGNNGRPYKPDTALRLRLRFNPAADNPLYRRGIPGDPDHQGIKDSYFPLRHGG

RVTLYQDAHYREGDLPEIELDEGGKVFDHNACWEDICHAILEAHHMIYIVGWSVYDKVRLVREPSPSRPL

PEGGDLNLGELLKFKSQEGVRVCLLVWDDKTSHDKLFIKTGGVMATHDEETRKFFKHSSVICVLSPRYAS

SKLSIFKQQVVGTLFTHHQKCVLVDTQAWGNKRKITAFIGGLDLCDGRYDTPEHRLFKDLDTVFDNDYHN

PTFPSGAKGGPRQPWHDLHCRIDGPAAYDVLKNFEQRWRKATKWRERFRKVSHWKDDALIKLERISWILS

PSPTIPNDDISLRVSKEEDPENWHVQVFRSIDSGSLKGFPSDCKEASKQNLICRKDLIIDKSIHTAYVRA

IRSAQHFIYIENQYFLGSSYAWPSYVNSGADNLVPIELALKIASKIRAGERFAVYVVIPMWPEGVPTAAS

VQEILFFQTMEMMYRIIAQELKAMNIKNAHPQDYLNFYCLGNREESSSSNGSPESNDKSAAALARKYRRF

MIYVHAKGMIVDDEYVILGSANINQRSLAGSRDTEIAMGAYQPHHTWSTKGGHPRGQVYGYRTSLWAEHL

GMVDDLFKDPSSLECVNYVNEIAEENWRRFTAEQLITLQGHLLKYPVKVEADGKVGPLPEHECFPDVGGK

ILGAPTSLPDTLTM

>OsPLDdelta2-AAF78756

MGKSSADLSASSESAVLLHGDLDIWITEAKCLPNMDIMSERMRRFFTGYGACGSSCAGDNARRGGVGVRP

KKIITSDPYVSVCLAGATVAQTRVIPNSENPRWEERFRVEVAHAVSRLEFHVKDNDVFGAQLIGVASLPV

DRILSGAPAEGWFPIDGHCSSNPMRPPPELRLSVQYRPIDDNPLYRGGAGAVPNAYFPLRRGGGVTLYQD

AHVADGGLPAIQIAGGRAYEHGRCWEDICHSIVEAHHLVYMVGWSIYHPVKLVREPTRALPGETPSTLGE

LLKKKAREGVRIVILLWDDKTSHDKFLLKTDGVMHTHDEETKKFFRHSGVHCVLVPRYASTKLSIFKQQV

VGTLFTHHQKCVLVDTQATGNNRKITAFIGGLDLCDGRYDTPEHRLFKDLDTAFNKDFHNPTFPVSSPVN

SYGPRQPWHDLHCKIEGPAAFDILTNFEQRWRKATKWKVNLKKVASWHHDTLIKINRMSWIVTPAADEAN

AHVCEEKDPENWHVQVFRSIDSGSVKGFPKIVQEAESQNLVCAKNLKIDKSIHSAYVKAIRSAQHFIYIE

NQYFIGSSFLWSSHKSAGADNLIPVELALKIASKIKANEQFAVYIVLPMWPEGIPTAAPMQQILFWQGQT

MSMMYKIIADALRMQGLVEAHPQDYLNFYCLGKREVAAGDSMSQTSLCNDNSTLRSAQKLRRFMIYVHSK

GMVVDDEYVIIGSANINQRSMEGCRDTEIAMGAYQPHYKWSADHDQGPPRGQVYGYRMSLWAEHLGAVEE

CFGRPETGECVRRVREMAEENWRAYVSPEMEETKGHLMCYPLKVDKDGRVRALPGHDCFPDVGGKVLGTQ

TSLPNALTT

>AtPLDdelta

MAEKVSEDVMLLHGDLDLKIVKARRLPNMDMFSEHLRRLFTACNACARPTDTDDVDPRDKGEFGDKNIRS

HRKVITSDPYVTVVVPQATLARTRVLKNSQEPLWDEKFNISIAHPFAYLEFQVKDDDVFGAQIIGTAKIP

VRDIASGERISGWFPVLGASGKPPKAETAIFIDMKFTPFDQIHSYRCGIAGDPERRGVRRTYFPVRKGSQ

VRLYQDAHVMDGTLPAIGLDNGKVYEHGKCWEDICYAISEAHHMIYIVGWSIFHKIKLVRETKVPRDKDM

TLGELLKYKSQEGVRVLLLVWDDKTSHDKFGIKTPGVMGTHDEETRKFFKHSSVICVLSPRYASSKLGLF

KQQASPSSSIYIMTVVGTLFTHHQKCVLVDTQAVGNNRKVTAFIGGLDLCDGRYDTPEHRILHDLDTVFK

DDFHNPTFPAGTKAPRQPWHDLHCRIDGPAAYDVLINFEQRWRKATRWKEFSLRLKGKTHWQDDALIRIG

RISWILSPVFKFLKDGTSIIPEDDPCVWVSKEDDPENWHVQIFRSIDSGSVKGFPKYEDEAEAQHLECAK

RLVVDKSIQTAYIQTIRSAQHFIYIENQYFLGSSYAWPSYRDAGADNLIPMELALKIVSKIRAKERFAVY

VVIPLWPEGDPKSGPVQEILYWQSQTMQMMYDVIAKELKAVQSDAHPLDYLNFYCLGKREQLPDDMPATN

GSVVSDSYNFQRFMIYVHAKGMIVDDEYVLMGSANINQRSMAGTKDTEIAMGAYQPNHTWAHKGRHPRGQ

VYGYRMSLWAEHLGKTGDEFVEPSDLECLKKVNTISEENWKRFIDPKFSELQGHLIKYPLQVDVDGKVSP

LPDYETFPDVGGKIIGAHSMALPDTLTT

>AtPLDalpha1

MELEEQKKYFHGTLEITIFDATPFSPPFPFNCICTKPKAAYVTIKINKKKVAKTSSEYDRIWNQTFQILC

AHPVTDTTITITLKTRCSVLGRFRISAEQILTSNSAVINGFFPLIADNGSTKRNLKLKCLMWFRPAYLEP

GWCRALEEASFQGIRNASFPQRSNCRVVLYQDAHHKATFDPRVDDVPFNARNLWEDVYKAIESARHLVYI

AGWALNPNLVLVRDNETEIPHAVGVTVGELLKRKSEEGVAVRVMLWNDETSLPMIKNKGVMRTNVERALA

YFRNTNVVCRLCPRLHKKLPTAFAHHQKTITLDTRVTNSSTKEREIMSFLGGFDLCDGRYDTEEHSLFRT

LGTEADFYQTSVAGAKLSRGGPREPWHDCHVSVVGGAAWDVLKNFEQRWTKQCNPSVLVNTSGIRNLVNL

TGPTEENNRKWNVQVLRSIDHISATEMPRGLPVEKSVHDGYVAAIRKAERFIYIENQYFMGSCDHWESKN

DKICSGCTNLIPVEIALKIAAKIRARERFAVYIVIPMWPEGPPESETVEEILHWTRETMSMMYQIIGEAI

WEVGDKSHPRDYLNFFCLANREEKRDGEFEAVSSPHQKTHYWNAQRNRRFMVYVHSKLMIVDDTYILIGS

ANINQRSMDGCRDTEIAIGCYQTNTNNTNEIQAYRLSLWYEHTGGKITADDLSSSEPESLECVRGLRTIG

EQMWEIYSGDKVVDMLGIHLVAYPISVTGDGAVEEVGDGCFPDTKTLVKGKRSKMFPPVLTT

>AtPLDzeta1

MASEQLMSPASGGGRYFQMQPEQFPSMVSSLFSFAPAPTQETNRIFEELPKAVIVSVSRPDAGDISPVLL

SYTIECQYKQFKWQLVKKASQVFYLHFALKKRAFIEEIHEKQEQVKEWLQNLGIGDHPPVVQDEDADEVP

LHQDESAKNRDVPSSAALPVIRPLGRQQSISVRGKHAMQEYLNHFLGNLDIVNSREVCRFLEVSMLSFSP

EYGPKLKEDYIMVKHLPKFSKSDDDSNRCCGCCWFCCCNDNWQKVWGVLKPGFLALLEDPFDAKLLDIIV

FDVLPVSNGNDGVDISLAVELKDHNPLRHAFKVTSGNRSIRIRAKNSAKVKDWVASINDAALRPPEGWCH

PHRFGSYAPPRGLTDDGSQAQWFVDGGAAFAAIAAAIENAKSEIFICGWWVCPELYLRRPFDPHTSSRLD

NLLENKAKQGVQIYILIYKEVALALKINSVYSKRRLLGIHENVRVLRYPDHFSSGVYLWSHHEKLVIVDN

QVCFIGGLDLCFGRYDTFEHKVGDNPSVTWPGKDYYNPRESEPNTWEDALKDELERKKHPRMPWHDVHCA

LWGPPCRDVARHFVQRWNYAKRNKAPYEDSIPLLMPQHHMVIPHYMGRQEESDIESKKEEDSIRGIRRDD

SFSSRSSLQDIPLLLPHEPVDQDGSSGGHKENGTNNRNGPFSFRKSKIEPVDGDTPMRGFVDDRNGLDLP

VAKRGSNAIDSEWWETQDHDYQVGSPDETGQVGPRTSCRCQIIRSVSQWSAGTSQVEESIHSAYRSLIDK

AEHFIYIENQFFISGLSGDDTVKNRVLEALYKRILRAHNEKKIFRVVVVIPLLPGFQGGIDDSGAASVRA

IMHWQYRTIYRGHNSILTNLYNTIGVKAHDYISFYGLRAYGKLSEDGPVATSQVYVHSKIMIVDDRAALI

GSANINDRSLLGSRDSEIGVLIEDTELVDSRMAGKPWKAGKFSSSLRLSLWSEHLGLRTGEIDQIIDPVS

DSTYKEIWMATAKTNTMIYQDVFSCVPNDLIHSRMAFRQSLSYWKEKLGHTTIDLGIAPEKLESYHNGDI

KRSDPMDRLKAIKGHLVSFPLDFMCKEDLRPVFNESEYYASPQVFH

>OsPLDzeta1-BAC00694

MSGGRSAAREGEEAEEEEVEEYGGGYVRMPQEPEGEAAAAGAGSFLRLPESAGAFDELPRARIVGVSRPD

AGDITPMLLSYTVEVQYKQFRWLLYKKASQVLYLHFALKRRAFLEEFHEKQEQVKEWLQNLGIGEHIPVV

HDDDEADDVHVPSQHDEHSVKNRNVPSSAVLPVIRPALGRQQSVSDRAKVAMQEYLNHFLGNMEIVNSRE

VCKFLEVSLLSFLPEYGPKLKEDYVTVGHLPKIDEGSSNICCLCGCFSFCNSSWQKVWAVLKPGFLALLQ

DPFDPKLLDIVIFDVSPHMDRNGEGQSTLAREIKEHNPLHFAFEVSSGGRTIKLRTRSSAKVKDWVSAIN

TARRPPEGWSHPHRFGSFAPPRGLTEDGSVVQWFLDGQAAFNAIASSIEEAKSEIFITDWWLCPELYLRR

PFHHHESSRLDILLESRAKQGVQIYILLYKEVSLALKINSMYSKQRLLNIHENVKVLRYPDHFSTGIYLW

SHHEKIVIVDNQVCYIGGLDLCFGRYDTPEHKVVDVPPSIWPGKDYYNPRESEPNSWEDTMKDELDRTKY

PRMPWHDVQCALYGPACRDIARHFVQRWNYAKRNKAPNEQAIPLLMPQHHMVIPHYMGKIKESNEEVSKQ

THVEDIKGQKLSSLKAPASCQDIPLLLPHEPDHQASNNGELDHPNKTHWKQPIPNRKAKQDTSLQDLQMK

GFVDNLGTPDVSSVIGHYDTSKQNVHHMDNEWWETQERGDQVDYVLDIGEVGPRATCCCQVVRSVGPWSA

GTTQIEGSIHNAYFSLIEKAEHFVYIENQFFISGLSGDDTIKNRVLEALYRRILRAEKEKRCFRVIIVIP

LLPGFQGGIDDGGAASVRAIMHWQYRTICRGPNSILKNLYDVVGSKAHDYISFYGLRAHGRLGDGGPLVT

NQIYVHSKLMIIDDRMTLIGSANINDRSLLGSRDSEIGMIIEDKEVVSSIMDGRHWEAGKFSLSLRLSLW

AEHLGLHPGEVSQIMDPVDDLTYNNIWMGTAKANTKIYQNVFSCVPNDHIHSRSQFRQGFAHRKEKIGHT

TIDLGVAVEITETHKDGDLAGTDPMEKLQAVRGHLVSFPLEFMCQEDLRPFFGESEYYTSPQVFH

>OsPLDphi-BAD38104

MRSSARGRGRLLLLLLLAAGAPAAPAAAEVATCKAWLVQSIPTDMPHLRRVPGVLSTADVLQWLSGNATK

SLDVLAQYWQFLAQPKNPKSGDYGYSESEMVRFGADKGQRVYKALEKAADRKIKIRIVQHSGFAPDFDKE

SADLAAGRPNVQNVTLLFGDWWGSGVVHAKVWISDKKDVYIGSANNDWKSLSQVKELGIYFADCPQIAKT

VEIYFQNLWKLSTLNSTAYTKVAWDKQWQAFRKVPCWSHFLKPEERCRSPLPPSVDVPYVDGYPSLANPK

MLDFSFETPGYKSSTKEHHLSYLSYAPPELSFDKFQADEQGWLDTIKSVKFGGVVRISTMDWLGQSQYAT

QTVFWPSLSSAISEVIFSKNATVRILVAYWTHFIPNTEKYLKSLLYSNILCTSSSYNHCMGKVEIKYYVV

PGYNKTGPALAQGAATGNRYPDFTRVNHGKYAVSDVRANIGTSNLIWDYFYTTAGVSFGTYSPAIVSQLQ

DIFDADWFSPYTVPVKPLEASA

**FAD protein sequence for phylogenic tree construct**

**>AtFAD2 AT3G12120.1**

**MGAGGRMPVPTSSKKSETDTTKRVPCEKPPFSVGDLKKAIPPHCFKRSIPRSFSYLISDIIIASCFYYVATNYFSLLPQPLSYLAWPLYWACQGCVLTGIWVIAHECGHHAFSDYQWLDDTVGLIFHSFLLVPYFSWKYSHRRHHSNTGSLERDEVFVPKQKSAIKWYGKYLNNPLGRIMMLTVQFVLGWPLYLAFNVSGRPYDGFACHFFPNAPIYNDRERLQIYLSDAGILAVCFGLYRYAAAQGMASMICLYGVPLLIVNAFLVLITYLQHTHPSLPHYDSSEWDWLRGALATVDRDYGILNKVFHNITDTHVAHHLFSTMPHYNAMEATKAIKPILGDYYQFDGTPWYVAMYREAKECIYVEPDREGDKKGVYWYNNKL***

**>AtFAD3 AT2G29980.1**

**MVVAMDQRTNVNGDPGAGDRKKEERFDPSAQPPFKIGDIRAAIPKHCWVKSPLRSMSYVVRDIIAVAALAIAAVYVDSWFLWPLYWAAQGTLFWAIFVLGHDCGHGSFSDIPLLNSVVGHILHSFILVPYHGWRISHRTHHQNHGHVENDESWVPLPERVYKKLPHSTRMLRYTVPLPMLAYPLYLCYRSPGKEGSHFNPYSSLFAPSERKLIATSTTCWSIMFVSLIALSFVFGPLAVLKVYGVPYIIFVMWLDAVTYLHHHGHDEKLPWYRGKEWSYLRGGLTTIDRDYGIFNNIHHDIGTHVIHHLFPQIPHYHLVDATKAAKHVLGRYYREPKTSGAIPIHLVESLVASIKKDHYVSDTGDIVFYETDPDLYVYASDKSKIN***

**>GmFAD2-1A Glyma.10G278000.1**

**MGLAKETTMGGRGRVAKVEVQGKKPLSRVPNTKPPFTVGQLKKAIPPHCFQRSLLTSFSYVVYDLSFAFIFYIATTYFHLLPQPFSLIAWPIYWVLQGCLLTGVWVIAHECGHHAFSKYQWVDDVVGLTLHSTLLVPYFSWKISHRRHHSNTGSLDRDEVFVPKPKSKVAWFSKYLNNPLGRAVSLLVTLTIGWPMYLAFNVSGRPYDSFASHYHPYAPIYSNRERLLIYVSDVALFSVTYSLYRVATLKGLVWLLCVYGVPLLIVNGFLVTITYLQHTHFALPHYDSSEWDWLKGALATMDRDYGILNKVFHHITDTHVAHHLFSTMPHYHAMEATNAIKPILGEYYQFDDTPFYKALWREARECLYVEPDEGTSEKGVYWYRNKY***

**>GmFAD2-1B Glyma.20G111000.1**

**MGLAKETIMGGGGRVAKVEIQQKKPLSRVPNTKPPFTVGQLKKAIPPHCFQRSLLTSLSYVVYDLSLAFIFYIATTYFHLLPHPFSLIAWPIYWVLQGCILTGVWVIAHECGHHAFSKYPWVDDVMGLTVHSALLVPYFSWKISHRRHHSNTGSLDRDEVFVPKPKSKVAWYTKYLNNPLGRAASLLITLTIGWPLYLAFNVSGRPYDGFASHYHPYAPIYSNRERLLIYVSDVALFSVTYLLYRVATMKGLVWLLCVYGVPLLIVNGFLVTITYLQHTHYALPHYDSSEWDWLRGALATMDRDYGILNKVFHHITDTHVAHHLFSTMPHYHATEATNAMKPILGEYYRFDDTPFYKALWREARECLYVEPDEGTSEKGVYWYRNKY***

**>GmFAD2-2A Glyma.19G147400.1**

**MGAGGRTAVPPANRKSEADPLKRVPFEKPQFSLSQIKKAIPPHCFQRSVLRSFSYVVYDLTIAFCLYYVATHYFHLLPGPLSFVAWPIYWAVQGCILTGVWVIAHECGHHAFSDYQLLDDIVGLILHSALLVPYFSWKYSHRRHHSNTGSLERDEVFVPKQKSSIMWYSKYLNNPPGRVLTLAVTLTLGWPLYLAFNVSGRPYDRFACHYDPYGPIYSDRERLQIYISDAGVLAVCYGLFCLAMAKGLAWVVCVYGVPLLVVNGFLVLITFLQHTHPALPHYTSSEWDWLRGALATVDRDYGILNKVFHNITDTHVAHHLFSTMPHYHAMEATKAIKPILGEYYRFDGTPFVKAMWREARECIYVEPDQSTQSKGVFWYNNKL***

**>GmFAD2-2B Glyma.19G147300.1**

**MGDTMKRVPIEKPPFTLSQIKKAIPPHFFQRSVLRSFSYLIYDLTIAFCLYYIATDYFHNLPHPLTFLAWPIYWAVQGFTLAGLWVIAHDCGHHAFRDYQLLDDNVGLVLHSALLVPYFSWKYSHRRHHSNTGSLERDEVFVPKQKSSTHVVHHLFSTMPHYHAMDATKAIKPILGEYYRFDETPFVKAMWREARECIYVEPDTENKGVFWYNNKL***

**>GmFAD2-2C Glyma.15G195200.1**

**AVQGCILTGVWVIAHECGHHAFSDYQLLDDIFGLVLHSGLLVPYFSWKYSHRRHHSNTGSLERDEVFVPKQKSCIKWYSKYLNNPPGRVLTLAVTLTLGWPLYLALNVSGRPYDRFAYHYDPYGPIYSDRERLQIYISDAGVLAVCYGLFRLAMAKGLAWVVCVYGVPLLVVNGFSVLITFLQHTQPALPHYTSSEWDWLRGALATVDRDYGILNKVFHNITDTHVAHHLFSTMPHYHAMEATKAIKPILGEYYRFDETPFVKAMWREARECIYVEPDQSTESKGVFWYNNKL***

**>GmFAD2-2D Glyma.03G144500.1**

**MGAGGRTDVPPANRKSEVDPLKRVPFEKPPFSLSQIKKVIPPHCFQRSVFRSFSYVVYDLTIAFCLYYVATHYFHLLPSPLSFLAWPIYWAVQGCILTGVWVIAHECGHHAFSDYQLLDDIVGLVLHSGLLVPYFSWKYSHRRHHSNTGSLERDEVFVPKQKSCIKWYSKYLNNPPGRVLTLAVTLTLGWPLYLALNVSGRPYDRFACHYDPYGPIYSDRERLQIYISDAGVLAVCYGLFRLAMAKGLAWVVCVYGVPLLVVNGFLVLITFLQHTHPALPHYTSSEWDWLRGALATVDRDYGILNKVFHNITDTHVAHHLFSTMPHYHAMEATKAIKPILGEYYRFDETPFVKAMWREARECIYVEPDQSTESKGVFWYNNKL***

**>GmFAD6A Glyma.02G203300.1**

**MACTLADSLLLFKGSYQKPVLRRDIAARYSPGIFSLNSNGLIQKRFRRQRNFVTRNKVTVIHAVAIPVQPAPVESAEYRKQLAEDYGFRQVGEPLPDDVTLKDVINSLPKEVFEIDDVKAWKSVLISVTSYALGLFMISKAPWYLLPLAWVWTGTAITGFFVIGHDCAHRSFSSNKLVEDIVGTLAFMPLIYPYEPWRFKHDRHHAKTNMLREDTAWHPVWKDEFESTPLLRKAIIYGYGPFRCWMSIAHWLMWHFDLKKFRPSEVPRVKISLACVFAFIAIGWPLIIYKTGIMGWIKFWLMPWLGYHFWMSTFTMVHHTAPHIPFKYSEEWNAAQAQLNGTVHCDYPKWIEILCHDINVHIPHHISPRIPSYNLRAAHKSLQENWGQYLNEASWNWRLMKTIMTVCHVYDKEQNYVAFDELAPEDSRPITFLKETMPDYA***

**>GmFAD6B Glyma.09G111900.1**

**MGGGGRSSATLKHQNSIKNHSKKKRVPHAKPPFTLSQLKKAISPHCFHRSTFRSFSYVLYDLTIASCLFYAAVNYIPTLPHENLSLLAWPLYWFIQGSILTGVWVIAHECGHHAFSDHQWLDDLVGLILHSLLLVPYFSWKYSHRRHHSNTGSLERDEVFVPKTKSSMGWYSKYLNNSPGRVLTLAITLTLGWPLYLAFNVSGRSYERFACHYDPYGPIYSNRERLQIYVSDAGILAVCFGLYKAVLAKGLVWVVCVYGVPLLVVNGFLVLITFLQHTHPAVPHYDSSEWDWLRGALATVDRDYGILNKVLHNITDTHVAHHLFSTMPHYHAMEATKAIKPILGEYYHFDETPIYKAMWREAKECMYVEPDKGSNGKGVYWYNNKL***

**>GmFAD3A Glyma.14G194300.1**

**MVKDTKPLAYAANNGYQKEAFDPSAPPPFKIAEIRVAIPKHCWVKNPWRSLSYVLRDVLVIAALMAAASHFNNWLLWLIYWPIQGTMFWALFVLGHDCGHGSFSDSPFLNSLVGHILHSSILVPYHGWRISHRTHHQNHGHIEKDESWVPLTEKIYKNLDNMTRLVRFTVPFPLFVYPIYLFSRSPGKEGSHFNPYSNLFPPSERKGIAISTLCWVTMFSMLIYLSFITSPVLLLKLYGIPYWIFVMWLDFVTYLHHHGHHQKLPWYRGKEWSYLRGGLTTVDRDYGWINNIHHDIGTHVIHHLFPQIPHYHLVEATQAAKSVLGEYYREPERSAPLPFHLIKYLIQSMRQDHFVSDTGDVVYYQTDSLHLHSHRD***

**>GmFAD3B Glyma.02G227200.1**

**MVKDTKPLAYAANNGYQQKGSSFDFDPSAPPPFKIAEIRASIPKHCWVKNPWRSLSYVLRDVLVIAALVAAAIHFDNWLLWLIYCPIQGTMFWALFVLGHDCGHGSFSDSPLLNSLVGHILHSSILVPYHGWRISHRTHHQNHGHIEKDESWVPLTEKIYKNLDSMTRLIRFTVPFPLFVYPIYLFSRSPGKEGSHFNPYSNLFPPSERKGIAISTLCWATMFSLLIYLSFITSPLLVLKLYGIPYWIFVMWLDFVTYLHHHGHHQKLPWYRGKEWSYLRGGLTTVDRDYGWINNIHHDIGTHVIHHLFPQIPHYHLVEATQAAKPVLGDYYREPERSAPLPFHLIKYLIQSMRQDHFVSDTGDVVYYQTDSLLLHSQRD***

**>GmFAD3C Glyma.18G062000.1**

**MVQAQPLQHVGNGAGKEDQAYFDPSAPPPFKIANIRAAIPKHCWEKNTLRSLSYVLRDVLVVTALVAAAIGFNSWFFWPLYWPAQGTMFWALFVLGHDCGHGSFSNSPLLNSIVGHILHSSILVPYHGWRISHRTHHQNHGHVEKDESWVPLTEKVYKNLDNMTRMMRFTLPFPIFAYPFYLWSRSPGKEGSHFNPYSNLFSPGERRDVLTSTLCWGIMLSVLLYLSLTMGPLFMLKLYGVPYLIFVMWLDFVTYLHHHGYKQKLPWYRGQEWSYLRGGLTTVDRDYGWINNIHHDIGTHVIHHLFPQIPHYHLIEATKAAKAVLGKYYREPQKSGPLPLHLIKYLLHSISQDHFVSDSGDIVYYQTDSQLHKDSWTQSN***

**LPAAT protein sequence for phylogenic tree construct**

**>GmLPAAT2α1 Glyma.02G181300.1**

**MTAVVVVPLGLLFFASGLIVNLIQAICYVVVRPVSKNLYRRMNRVVAELLWLELVWIIDWWAGVKVQVFTDPETFHSMGKEHALVISNHRSDIDWLVGWVLAQRSGCLGSTLAVMKKSSKFLPVIGWSMWFSEYLFLERSWAKDERTLKSGLQQLRDFPLPFWLALFVEGTRFTQAKLLAAQEYAASAGLPVPRNVLIPRTKGFVSAVNHMRSFVPAIYDVTVAIPKSSPAPTMLRLFRGKSSLVHVHIKRHAMKDLPEEDEAVAQWCRDVFVAKDALLDKHIAEDTFSDQELQDTGRPVKSLVVVILWACVVVTGVVKFLQWSSLLSSWKGVAFSAFGLGVVTLLMHILIMFSQSERSTPSKVAPTKKSKNSEQLEARDNKQD***

**>GmLPAAT2α2 Glyma.10G095500.1**

**MTAVVVVPLGLLFFASGLIVNLIQAICYVVVRPVSKNLYRRMNRVVAELLWLELVWIIDWWAGVKVQVFTDPETFRSMGKEHALVISNHRSDIDWLVGWVLAQRSGCLGSTLAVMKKSSKFLPVIGWSMWFSEYLFLERSWAKDERTLKSGLQQLRDFPLPFWLALFVEGTRFTQAKLLAAQEYAASAGLPVPRNVLIPRTKGFVSAVNHMRSFVPAIYDVTVAIPKSSPAPTMLRLFRGKSSVVHVHIKRHAMKDLPEEDEAVAQWCRDMFVAKDTLLDKHIAEDTFSDQELQDTGRPIKSLVVVISWACVVVMGVVKFLQWSSLLSSWKGVAFSAFGLGVVTLLMHILIMFSQSERSTPTKVAPAKSKNSEQLEARDNKQD***

**>GmLPAAT2α3 Glyma.03G139700.1**

**MAIAAAAVVVPLGLLFFASGLLVNLIQAICYVVVRPVSKSLYRRINRVVAELLWLELVWLIDWWAGVKVQIFTDHETFRLMGKEHALVISNHRSDIDWLVGWVSAQRSGCLGSTLAVMKKSSKFLPVIGWSMWFSEYLFLERSWAKDESTLKSGIQRLSDFPLPFWLALFVEGTRFTQAKLLAAQEYATSTGLPVPRNVLIPRTKGFVSAVSHMRSFVPAIYDVTVAIPKSSPAPTMLRLFKGQPSVVHVHIKRHLMKELPDTDEAVAQWCRDIFVAKDALLDKHMAEGTFSDQELQDTGRPIKSLLVVISWACLVVAGSVKFLQWSSLLSSWKGVAFSAFGLAVVTALMQILIQFSQSERSNPAKIVPAKSKNKGS***

**>GmLPAAT2α4 Glyma.19G142500.1**

**MAIAAAAVVVPLGLLFFASGLLVNLIQAICYVVVRPVSKNLYRRINRVVAELLWLELVWLIDWWAGVKVQIFTDHETFHLMGKEHALVISNHRSDIDWLVGWVSAQRSGCLGSTLAVMKKSSKFLPVIGWSMWFSEYLFLERSWAKDESTLKSGIQQLSDFPLPFWLALFVEGTRFTQAKLLAAQEYATSTGLSVPRNVLIPRTKGFVSAVSHMRSFVPAIYDITVAIPKSSPAPTMLRLFKGQPSVVHVHIKRHLMKELPETDEAVAQWCRDIFVAKDVLLDKHIAEDSFSDQDLQDTGRPIKSLLVVISWVCLVVAGSVKFLQRSSLLSSWKGVAFSAFGLAVVTALMQILIQFSQSERSNPAKIARAKSKNKGGQLEARNDKQQ***

**>GmLPAAT3 Glyma.15G034100.1**

**MGAKHFLEKIIGYVGGEIADEDGVFQGGIGPALADAEGGPIEPEGLVGGVVGLENPFRCGVGDELDEAVALGLARELVADDFDGDHLAGGGEAVAQAVFFVLLRPLSKNCYSRINKLLTESLWLELIWLIDWWAGIKIELYTDSETLQLMGKENALVICNHRSDIDWLIGWVLAQRSGCLGSTVAIMKKEVKFLPVLGWSMWFAEYIFLERDWAKDETSLKSGFRHLEHMPFPFWLALFVEGTRFTQTKLLQAQEFAASKGLPIPRNVLIPRTKLTIFLQGFVTAVQSLRPFVPAIYDCTYAVPKSEASPTLVRIFKGISCPVKVQIKRHKMEELPETDDGIGQWCKDAFVAKDALLEKYSTTEIFSEQDLQQIRRHKTSILVVVCWLCLLGFLVYEFFQWTSLLSSWEGILFTVLFLLLVTVIMEIFIHSSQSERSKPPMVLPTQDPMKQKLLQT***

**>GmLPAAT4α1 Glyma.14G077500.1**

**MEVCEPVKSENRLKHRPLTPFRLLRGLICLVVFLSTALMCLVYFVPVAVVGLRLFSVRCSRKTVSFIFGLWLSLWPSLFEKINKTKVVFSGDSVPMKERVLLIANHRTEVDWMYLWDLALRKGRLGCIKYILKSSLMKLPIFGWGFHILEFIAVERKWEIDEQILQQKLSTFKDPQDPLWLALFPEGTDYTDQKSKTSKKFAAEAGLPVLTNVLLPKTKGFHACLEALRGSLDAVYDVTIAYKNQCPSFLDNVFGVDPSEVHLHVRRIPVEEIPASETKAASWLIDTFQIKDQLLSDFKIQGHFPNQLNENEISRFKSLLSFMVIVSFTAMFIYFTFFSVIWFKLYVGLSCAYLTLATRFNFQLMPLSSYVHALHNSKNQKSE***

**>GmLPAAT4α2 Glyma.17G248200.1**

**MEVCEPVKSENRLKHRPLTPIRFLRGLIGLVVFLSTALMCLVYFVPVAVVGLRLFSIRCSRKTVSFIFGLWLSLWPSLFEKINKTKVVFSGDGVPMKERVLLIANHRTEVDWMYLWDLALRKGRLGCIKYILKSSLMKLPIFGWGFHILEFIAVERKWEIDEQILQQKLSTLKDPQDPLWLALFPEGTDYTDQKSKNSQKFAAEAGLPVLTNVLLPKTKGFHACLEALRASLDAVYDVTIAYKNQCPSFLDNVFGVDPPEVHLHVRRIPVEEIPASETKAASWLIDTFQIKDQLLSDFKIQGHFPNQLNENEISTFKSLVSFMVIVSFTAMFTYFTFFSVIWFKLYVGLSCAYLSLATRFNFQLMPLTYYVHALHKSKNQKSE***

**>GmLPAAT5α2 Glyma.11G120600.1**

**MAVLKPVSSCFGAKYQALAPWRILRGLVCLLVLLSTAFIMLVFFGFISAVIMRLFSVIYSRRATSFFFGAWLALWPFLFEKINKTKVVFSGDIVPSRERILLIANHRTEVDWMYLWDLALRKGCLGYIKYILKSSLMRLPVFGWAFHILEFIPVERKWEADESIMRYMLSTFKDPQDPLWLALFPEGTDFTEQKCLRSKKYAAEHGLPVLKNVLLPKTKGFCACLQELRSSLAAVYDVTIGYKYRCPSFLDNVFGVDPSEVHIHICRFPLDSIPVSEEEMSMWLINRFQTKDQLLSNFQTQGQFPDQAAERDLSAVKSILNCMTIVTVTGTTMYYCFSSFWFKLYVSLVCAYLVPATYFNTRPQPILSFLKVIAR***

**>GmLPAAT5α1 Glyma.12G045700.1**

**MAVLTPVSSCFGAKYQALAPLRILRGLLCLLVLLSTAFMMLVFFGFISAVVVRVFSVSYSRRATSFIFGAWLALWPFLFEKINKTKVVFSGDIVPSRERILLIANHRTEVDWMYLWDLALRKGCLGYIKYILKSSLMRLPVFGWAFHILEFIPVKRKWEADESIMRHMLSTFKDPQDPLWLALFPEGTDFTEQKCLRSQKYAAEHKLPVLKNVLLPRTKGFCACLQELRSSLTAVYDVTIGYKYRCPSFLDNVFGVDPSEVHIHIHRFPLDSIPVSEDEISMWLINRFQTKDQLLSNFQTQGQFPDQASERDLSAVTSILNCMTIVTVTGTMMYYSFASFWFKLYVSLVCAYLVPATYFNTRPQPILSFLKMRAR***

**>GmLPAAT1α2 Glyma.06G220300.1**

**MEVTPLSSPSPIHRLHLRHKEARFLAVPSTLLCTRRGTTTYVHPILRTSHNCQSPPCSLQAISKKHENVSWLSVSPKLHVQNKFPRDVVVRSELTAAGSAGDGYLLPELKLESKVRGVCFYVVTAFSAIFLFMLMLVGHPSVLLFDRYRRMFHHFVAKVWAALTVAPFYKIEFEGLENLPPPDTPAVYVSNHQSFLDIYTLLTLGRSFKFISKTGIFLFPIIGWAMFLLGVIPLKRMDSRSQLDCLKRCMDLIKKGASVFFFPEGTRSKDGKLGTFKKGAFSVAAKTNAPVVPISLIGTGQIMPAGKEGIVNLGSVKVVIHKPIVGKDPDMLCKEARKTIASVLTQS***

**>GmLPAAT1α1 Glyma.12G163500.1**

**MEVTPLSSPSPIHGLHLRHKEARFLAVASTLYFDGIPFILQLRTHRGTTTYKHPILRTSHNAPQCSLRAISKKHENVAWLSVSPKLHVQNKFPRDVVVRSELTAAGSAGDGYFLPELKVESKVRGVCFYVVTAFCAIFLFMMMLVGHPSVLLFDRYRRKFHHFIAKVWATLTVAPFYKIKFEGLENLPPPDTPAVYVSNHQSFLDIYTLLTLGRSFKFISKTGIFLFPIIGWAMFLLGIIPLKRMDSRSQLDCLKRCMDLIKKGASVFFFPEGTRSKDGRLGTFKKGAFSVAAKTNAPVVPITLIGTGQIMPAGKEGIVNIGSVKVVIHKPIVGKDPDMLCKEARKTIASVLTQS***

**>AtLPAAT1 AT4G30580.1**

**MDVASARSISSHPSYYGKPICSSQSSLIRISRDKVCCFGRISNGMTSFTTSLHAVPSEKFMGETRRTGIQWSNRSLRHDPYRFLDKKSPRSSQLARDITVRADLSGAATPDSSFPEPEIKLSSRLRGIFFCVVAGISATFLIVLMIIGHPFVLLFDPYRRKFHHFIAKLWASISIYPFYKINIEGLENLPSSDTPAVYVSNHQSFLDIYTLLSLGKSFKFISKTGIFVIPIIGWAMSMMGVVPLKRMDPRSQVDCLKRCMELLKKGASVFFFPEGTRSKDGRLGSFKKGAFTVAAKTGVAVVPITLMGTGKIMPTGSEGILNHGNVRVIIHKPIHGSKADVLCNEARSKIAESMDL***

**>AtLPAAT2 AT3G57650.1**

**MVIAAAVIVPLGLLFFISGLAVNLFQAVCYVLIRPLSKNTYRKINRVVAETLWLELVWIVDWWAGVKIQVFADNETFNRMGKEHALVVCNHRSDIDWLVGWILAQRSGCLGSALAVMKKSSKFLPVIGWSMWFSEYLFLERNWAKDESTLKSGLQRLSDFPRPFWLALFVEGTRFTEAKLKAAQEYAASSELPIPRNVLIPRTKGFVSAVSNMRSFVPAIYDMTVTIPKTSPPPTMLRLFKGQPSVVHVHIKCHSMKDLPESDDAIAQWCRDQFVAKDALLDKHIAADTFPGQQEQNIGRPIKSLAVVLSWACVLTLGAIKFLHWAQLFSSWKGITISALGLGIITLCMQILIRSSQSERSTPAKVVPAKPKDNHHPESSSQTETEKEK***

**>AtLPAAT3 AT1G51260.1**

**MKIPAALVFIPVGVLFLISGLIVNIIQLVFFIIVRPFSRSLYRRINKNVAELLWLQLIWLFDWWACIKINLYVDAETLELIGKEHALVLSNHRSDIDWLIGWVMAQRVGCLGSSLAIMKKEAKYLPIIGWSMWFSDYIFLERSWAKDENTLKAGFKRLEDFPMTFWLALFVEGTRFTQEKLEAAQEYASIRSLPSPRNVLIPRTKGFVSAVSEIRSFVPAIYDCTLTVHNNQPTPTLLRMFSGQSSEINLQMRRHKMSELPETDDGIAQWCQDLFITKDAQLEKYFTKDVFSDLEVHQINRPIKPLIVVIIWLGFLVFGGFKLLQWLSIVASWKIILLFVFFLVIATITMQILIQSSESQRSTPAKRPLQEQLISA***

**>AtLPAAT4 AT1G75020.1**

**MEVCGDLKSDNLKNRPLTPLRILRGLMILLVFLSTAFMFLLYFAPIAALGLRLLSVQQSRKVVSLIFGLWLALWPYLFETVNGTTVVFSGDIIPVEKRVLLIANHRTEVDWMYLWNIALRKGCLGYIKYVLKSSLMKLPIFGWGFHVLEFIPVERKREVDEPVLLQMLSSFKDPQEPLWLALFPEGTDFTEEKCKRSQKFAAEVGLPALSNVLLPKTRGFGVCLEVLHNSLDAVYDLTIAYKPRCPSFMDNVFGTDPSEVHIHVRRVLLKEIPANEAESSAWLMDSFKLKDKLLSDFNAQGKFPNQRPEEELSVLKCIATFAGVISLTVVFIYLTLYSHSCFKVYACLSGTYLTFATYYKFQPSPGCFREDSCKVKNH***

**>AtLPAAT5 AT3G18850.1**

**MEKKSVPNSDKLSLIRVLRGIICLMVLVSTAFMMLIFWGFLSAVVLRLFSIRYSRKCVSFFFGSWLALWPFLFEKINKTKVIFSGDKVPCEDRVLLIANHRTEVDWMYFWDLALRKGQIGNIKYVLKSSLMKLPLFGWAFHLFEFIPVERRWEVDEANLRQIVSSFKDPRDALWLALFPEGTDYTEAKCQRSKKFAAENGLPILNNVLLPRTKGFVSCLQELSCSLDAVYDVTIGYKTRCPSFLDNVYGIEPSEVHIHIRRINLTQIPNQEKDINAWLMNTFQLKDQLLNDFYSNGHFPNEGTEKEFNTKKYLINCLAVIAFTTICTHLTFFSSMIWFRIYVSLACVYLTSATHFNLRSVPLVETAKNSLKLVNK***

**>BjLPAAT**

**MAMAAAVIVPLGILFFISGLVVNLLQAVCYVLVRPMSKNTYRKINRVVAETLWLELVWIVDWWAGVKIQVFADDETFNRMGKEHALVVCNHRSDIDWLVGWILAQRSGCLGSALAVMKKSSKFLPVIGWSMWFSEYLFLERNWAKDESTLQSGLQRLNDFPRPFWLALFVEGTRFTEAKLKAAQEYAASSELPVPRNVLIPRTKGFVSAVSNMRSFVPAIYDMTVAIPKTSPPPTMLRLFKGQPSVVHVHIKCHSMKDLPEPEDEIAQWCRDQFVAKDALLDKHIAADTFPGQKEQNIGRPIKSLAVVVSWACLLTLGAMKFLHWSNLFSSWKGIALSAFGLGIITLCMQILIRSSQSERSTPAKVAPAKPKDNHQSGPSSQTEVEEKQK**

**DGAT protein sequence**

**>GmDGAT2B Glyma.16G115700.1**

**MQRTAAATEEPRRSSGDASAAEGEKVFKGSEVFGDTSPNYLKTILALALWLGTIHFNAALVLFAIFFLSLHKAFLLFGLLFVLMVIPVDEKSKFGRKLSRRKKKIWVCFRYICKHVCAYFPITLHVEDMKAFHPSRAYVFGYEPHSVLPIGVVALADNTCFMPLPKIKVLASSAIFYTPFLRHIWTWLGLTPVTKKRFTSLLDAGYSCILIPGGVQEAFLIEHGSEIAFLKSRRGFVRIAMEKGKPLVPVFCFGQSNVYKWWKPGGKLVLNFARAVKFSPVYFWGIFGSPIPFKHPMHVVVGRPIELEKTPEPTPEEVAKIHSQFVEALQDLFERHKARAGYPNLELRIV***

**>GmDGAT2A Glyma.09G195400.1**

**MQRTAAATDEPRRRSGDAEGEKVFKGSEVFGDTSPNYLKTILALALWLGTIHFNVALVLFAIFFLSLHKALLLFGLLFVLMVIPVDEKSKFGRKLSRYICKHVCAYFPITLHVEDMKAFHPNRAYVFGYEPHSVLPIGVVALADNTGFMPLPKIKVLASSAIFYTPFLRHIWTWLGLTPVTRKRFTSLLDAGYSCILIPGGVQEAFLMEHGSEIAYLKARRGFVRIAMEKGKPLVPVFCFGQSNVYKWWKPGGKLILNFARAVKFSPIYFWGIFGSPIPFKHPMHVVVGRPIELEKNHEPTPEEVARIHSQFVEALQDLFERHKARAGYPNLELRIV***

**>GmDGAT2E Glyma.11G088800.1**

**QWRCGCIHFNIALVLFAVFFLPLSKSILVFGFLFVFMVLPINQKSRFGRNLSRFICKHACNYFPITLHVEDMKAFDPNRAYVFGYEPHSVLPIGIVALAEHTGFMPLPKVKVFYTPFLRHTWTWLGLTPATKKIFISLLASGHSCILIPGGVQEAFLMQRGTEIAFLKARRGFVRITMVKGRPLVPVFCFGQSNVYKWWKPGGKFFLKFARAIPGAAVKSPLPFRHPMHVVVGRPIELDKKPEPTTEEVAKIHSQFVEALQDLFERLSKP***

**>GmDGAT2D Glyma.01G156000.1**

**MAAEPVSDGGAAAEKLISGREEFGDSSNLFSAILAMVLWLGAIHFNIALILLAVFFLPLSKSLLVFGFLFGFMVLPINEKSRFGRRLSRFICKHACNYFPITLHVEDMKAFDPNRAYVFGYEPHSVLPIGIVALADHTGFMPLPKVKVLASSTVFYTPFLRHLWTWLGLTPATKKNFISLLASGHSCILIPGGVQEAFHMQHGTEIAFLKARRGFVRVAMVKGKPLVPVFCFGQSNVYKWWKPGGKLFLKFARAIKFTPICFWGIFGSPLPFRHPMHVVVGRPIEVDKNREPTTEEVAKIHGLFVEALQDLFERHKARAGYPNLELRIV***

**>GmDGAT2C Glyma.16G115800.1**

**MGKVFNGVEEFSESRNVFKTVPALVLYLGAIHFNLALILWATVFLPLSKGLLVFGLLLVFVLIPVDENSIFGHKLSKYICKHICSYFPITLHVEEAKAFRPDQAYVFGYEPHSVFPIGIVALGDSTGFMPLAKTKFLASSAVRVFYIPFLRHIWTWLGFTPVTKQNFISSLEAGYSCILVPGGVRETFFMEPGCEIAFLKQRRGFVRIALQMGLPLVPVFCFGQTKAYKWWKPPGRLMQNLARFLKIIPLFFWGIYGSPIPFKNPLYIVVGRPIELEKNPEPTMEQVAKVHSQFVEALQDLFDRHKAHAGYTNLELKIF***

**>GmDGAT1B Glyma.17G053300.1**

**MAISDEPESVATALNHSSLRRRPSATSTAGLFNSPETTTDSSGDDLAKDSGSDDSINSDDAAVNSQQQNEKQDTDFSVLKFAYRPSVPAHRKVKESPLSSDTIFRQSHAGLFNLCIVVLVAVNSRLIIENLMKYGWLIKSGFWFSSKSLRDWPLFMCCLSLVVFPFAAFIVEKLAQRKCIPEPVVVVLHIIITSTSLFYPVLVILRCDSAFVSGVTLMLFSCVVWLKLVSYAHTNYDMRALTKLVEKGEALLDTLNMDYPYNVSFKSLAYFLVAPTLCYQPSYPRTPYIRKGWLFRQLVKLIIFTGVMGFIIEQYINPIVQNSQHPLKGNLLYATERVLKLSVPNLYVWLCMFYCFFHLWLNILAELLRFGDREFYKDWWNAKTVEDYWRMWNMPVHKWMIRHLYFPCLRHGLPKAAALLIAFLVSALFHELCIAVPCHIFKLWAFGGIMFQVPLVLITNYLQNKFRNSMVGNMIFWFIFSILGQPMCVLLYYHDLMNRKGKLD***

**>GmDGAT1A Glyma.13G106100.1**

**MAISDEPETVATALNHSSLRRRPTAAGLFNSPETTTDSSGDDLAKDSGSDDSISSDAANSQPQQKQDTDFSVLKFAYRPSVPAHRKVKESPLSSDTIFRQLQSHAGLFNLCIVVLVAVNSRLIIENLMKYGWLIKSGFWFSSKSLRDWPLFMCCLSLVVFPFAAFIVEKLAQQKCIPEPVVVVLHIIITSASLFYPVLVILRCDSAFLSGVTLMLFACVVWLKLVSYAHTNYDMRALTKSVEKGEALPDTLNMDYPYNVSFKSLAYFLVAPTLCYQPSYPRTPYIRKGWLFRQLVKLIIFTGVMGFIIEQYINPIVQNSQHPLKGNLLYAIERVLKLSVPNLYVWLCMFYCFFHLWLNILAELLRFGDREFYQDWWNAKTVEDYWRMWNMPVHKWMIRHLYFPCLRHGIPKAVALLIAFLVSALFHELCIAVPCHIFKLWAFGGIMFQVPLVFITNYLQNKFRNSMVGNMIFWFIFSILGQPMCVLLYYHDLMNRKGKLD***

**>GmDGAT1C Glyma.09G065300.1**

**MAISDVPAAAGTTATTTSDSDLRQPSLRRRSSAGVLFDAARDSGSDNSLTGKITDDDNIKDHKPNNHAASDDNVGAAANDAGQEHRQPVADFKYAYRPSVPAHRRIKESPLSSDNIFRQSHAGLFNLCIVVLVAVNSRLIIENLMKYGWLIKYGFWFSSKSLRDWPLFMCCLSLAIFPLAAFVVERLAQQKCISEPVVVLLHLIISTVELCYPVLVILRCDSAFVSGVTLMLLTCIVWLKLVSYAHTNYDMRALTVSNEKGETLPNTLIMEYPYTVTFRSLAYFMVAPTLCYQTSYPRTPSVRKGWVFRQLVKLIIFTGVMGFIIEQYMNPIVQNSTHPLKGNLLYAIERILKLSVPNVYVWLCMFYCFFHLWLNILAELVRFGDREFYKDWWNAKTVEEYWRMWNMPVHKWMVRHIYFPCLRRGIPKGAASLIAFLVSAVFHELCIAVPCHMFKLWAFIGIMFQVPLVLITNYLQNKYRNSMVGNMIFWFIFCILGQPMSVLLYYHDLMNRKGEVD***

**>GmDGAT3A Glyma.13G118300.1**

**MEISGSVLRQLSYVSGYGTPTRSRGVASRVGLRMGTGSGFCDEGHLQYYQDTKKILTPKKLKLLKGFSKLGLASDPEKLAMFHDLQQNLTSDAGEVLLRELEAARAKEKEMKKKRKQEIKAKLKASKMNCESSSSSSSESSDSDGDCDQVVDMNCFRAGAGVVVPAPVEESPLPKTPIVEDTNAKAHRDAMALCSKNDISVSSVRDCIKSESAVVTAAPQKRIEVCMGTKCKRSGAAALMQEFERVVGVEGGAVVSCKCMGKCKTAPNVKVQNSVDHSLARGLDDSVNIPANPLCIGVGLGDVDAIVARFLGESHTDIGMIGAATAT***

**>GmDGAT3B Glyma.17G041600.1**

**MEISGTVLRQVSYVSGYGTHTRSRGLAPRFGVRMGMGSGFCDEGHLRYYQDTKKVLTPKKKLKLLKGFSKLGLASDPEKLAMFYDLQQNLTSDAGDVLLRELEAARAKEKEVKKKRKQEKKAKLKAAKMNCESSSSSSSESSDSDCGCDQVVDMNTFRAGVGVGVGVGVGVVAPAPVEESPLPKTAPIVEDANAHCVAMELCSKNDIYVSSASNGFKNESAVVTSAPQKRIEVCMGNKCKRSGAAALMQEFEKVVGVEGVAVVACKCMGKCKTAPNVKVQNSVDHNSLAQGLDDSVKIPANPLCIGVGLEDVDAIVARYFWESHTDIGMAGAGAATAT***

**>AtDGAT1 AT2G19450.1**

**MAILDSAGVTTVTENGGGEFVDLDRLRRRKSRSDSSNGLLLSGSDNNSPSDDVGAPADVRDRIDSVVNDDAQGTANLAGDNNGGGDNNGGGRGGGEGRGNADATFTYRPSVPAHRRARESPLSSDAIFKQSHAGLFNLCVVVLIAVNSRLIIENLMKYGWLIRTDFWFSSRSLRDWPLFMCCISLSIFPLAAFTVEKLVLQKYISEPVVIFLHIIITMTEVLYPVYVTLRCDSAFLSGVTLMLLTCIVWLKLVSYAHTSYDIRSLANAADKANPEVSYYVSLKSLAYFMVAPTLCYQPSYPRSACIRKGWVARQFAKLVIFTGFMGFIIEQYINPIVRNSKHPLKGDLLYAIERVLKLSVPNLYVWLCMFYCFFHLWLNILAELLCFGDREFYKDWWNAKSVGDYWRMWNMPVHKWMVRHIYFPCLRSKIPKTLAIIIAFLVSAVFHELCIAVPCRLFKLWAFLGIMFQVPLVFITNYLQERFGSTVGNMIFWFIFCIFGQPMCVLLYYHDLMNRKGSMS***

**>AtDGAT2 AT3G51520.1**

**MGGSREFRAEEHSNQFHSIIAMAIWLGAIHFNVALVLCSLIFLPPSLSLMVLGLLSLFIFIPIDHRSKYGRKLARYICKHACNYFPVSLYVEDYEAFQPNRAYVFGYEPHSVLPIGVVALCDLTGFMPIPNIKVLASSAIFYTPFLRHIWTWLGLTAASRKNFTSLLDSGYSCVLVPGGVQETFHMQHDAENVFLSRRRGFVRIAMEQGSPLVPVFCFGQARVYKWWKPDCDLYLKLSRAIRFTPICFWGVFGSPLPCRQPMHVVVGKPIEVTKTLKPTDEEIAKFHGQYVEALRDLFERHKSRVGYDLELKIL***

**>AtDGAT3 AT1G48300.1**

**MEKEKKALKKKAKVLKSLSKNLDMFSSIGFGLDPEAGLVGEIQTKTISEATEILVKQLEQLKAEEKILKKQRKEEKAKAKAMKKMTEMDSESSSSSESSDSDCDKGKVVDMSSLRNKAKPVLEPLQPEATVATLPRIQEDAISCKNTSEALQIALQTSTIFPSMANPGQTLKTVEAVSVVGLPLNRVEVCMGGKCKRSGGALLLDEFQRAMTGFEGSAVACKCMGKCRDGPNVRVVKETDAVMTDSVRTPSKTLCVGVGLQDVETIVTSFFDEECSREGLGSVSY***

**PDAT protein sequence**

**>GmPDAT1A Glyma.17G051300.1**

**MSFLRRRKAPANPSPNEEKEKDKKKVIKEKKIIKGENIIIKRWSCVDSCCWFVGLICSIWWFLLFLYNAMPASFPQYVTEAITGPLPDPPGVKLRKEGLTVKHPVVFVPGIVTGGLELWEGRQCADGLFRKRLWGGTFGELYKRPLCWVEHMSLDNETGLDHPGIRVRPVSGLVAADYFAPGYFVWAVLIANLARIGYEEKNMYMAAYDWRISFQNTEVRDQTLSRMKSNIELMVATNGGNKVVVIPHSMGVLYFLHFMKWVEAPAPMGGGGGSDWCAKHIKAVMNIGGPFLGVPKSVAGLFSIEARDIAVARTFAPGFWDKDVFGLKTLQHLMRMTRTWDSTMSMIPKGGDTIWGGLDWSADVSYNCSVKKNKNNDTYGAFQNGKENLGFMKNINYGRLISFGKDIADLHSSKLERLDFRGALKGRNLANTSNCDVWTEYHDMGVDGIKAVTDYKAYTADSILDLLHFVAPKMMKRGDAHFSYGIADNLDDQKYKHYKYWSNPLETRLPNAPDMEIYSMYGVGIPTERAYVYKLTPQSECHIPFQIDTSADGGNEYTCLRDGVYSSDGDETVPVLSAGFMCAKGWRGRTRFNPSGIQTFIREYDHAPPANLLEGRGTQSGAHVDILGNFALLEDIIRVAAGASGKDLGGDRVHSDIFKWSEKINLKL***

**>GmPDAT1B Glyma.13G108100.1**

**MSFIRRRKAPANPVRNDEEKEKDKKKVIKAKIIEGEKITIKRWSCVDSCCWFVGLICSIWWFLLFLYNAMPASFPQYVAEAITGPLPDPPGVKLRKEGLTVKHPVVFVPGIVTGGLELWEGRQCADGLFRKRLWGGTFGELYKRPLCWVEHMSLDNETGLDRPGIRVRPVSGLVAADYFAPGYFVWAVLIANLARIGYEEKNMYMAAYDWRISFQNTEVRDRTLSRMKSNIELMVATNGGNKVVVIPHSMGVLYFLHFMKWVEAPAPMGGGGGSDWCAKHIKAVMNIGGPFLGVPKSVAGLFSIEARDIAVARTFAPGFLDKDVFGLQTLQHLMRMTRTWDSTMSMIPKGGDTIWGGLDWSADVSYNCSAKKHKNNDTYSAFQNGKENLGFMKNINYGRLISFGKDIAELHSSKLERLDFRGALKGRNLANTSNCDVWTEYHDMGVEGIKAVTDYKAYTADSILDLLHFVAPKMMKRGDAHFSYGIAGNLDDQKYKHYKYWSNPLETRLPNAPDMEIYSMYGVGIPTERAYVYKLTPQSECHIPFQIDTSADGGNEYTCLRDGVYSSDGDETVPVLSAGFMCAKGWRGKTRFNPSGIRTFIREYDHAPPANLLEGRGTQSGAHVDILGNFALLEDIIRVAAGASGEDLGGDRVHSDIFKWSEKINLKL***

**>GmPDAT1C Glyma.07G036400.1**

**MSLLRRRKGSEPEKGPSPSSEPKVLSEDETEDDKNNKKNKKKRDEVGEKKKNKWSCFDSCCWWVGCICTLWWFLLFLYQMMPSSIPQYVTEAFTGPMPDPPGLKLKKEGLKVKHPVVFVPGIVTGGLELWEGHLCAEGLFRKRLWGGTFGEVYKRPSCWVDHMSLDNETGLDPPGIRVRPVSGLVAADYFAAGYFVWAVLIANLARIGYEEKTMYMAAYDWRIAFQNTEVRDQTLSRIKSNIELMVATNGGNKAVIIPHSMGVLYFLHFMKWVEAPAPTGGGGGPDWCSTYIKAVVNIGGPFLGVPKAIAGLFSAEARDIAVARTIAPGFLDNDLFRIQTLQHVMKMTRTWDSTMSMIPRGGDTIWGGLDWSPEEGYHPSQRKHSNNNTQLKDHETNQTNFVNYGRMISFGRDVAEAHSPEIQMTDFRGAIKGRSIANTTCRDVWTEYHEMGFEGVRAVAEHKVYTAGSVVDLLQFVAPKMMARGSAHFSYGIADNLDDPKYNHYKYWSNPLETKLPNAPDMEIFSMYGVGLPTERSYIYKLTPFAECYIPFEIDTTQDGGSDEDSCLQGGVYTVDGDETVPVLSSGFMCAKGWRGKTRFNPSGIRTYVREYDHSPPANLLEGRGTQSGAHVDIMGNFALIEDVIRVAAGAKGEDLGGDKVYSDIFKWSEKIKLPL***

**>GmPDAT1D Glyma.16G005800.1**

**MSLLRRRKGSEPGKGPSHSSEPKVLSEEETEDDKNKKNKKKKNNKWSCFDSCCWGVGCICTLWWFLLFLYQMMPSSIPQYVTEAFTGPMPDPPGLKLKKEGLKVKHPVVFVPGIVTGGLELWEGHQCAEGLFRKRLWGGTFGEVYKRPSCWVDHMSLDNETGLDPPGIRVRPVSGLVAADYFAAGYFVWAVLIANLARIGYEEKTMYMAAYDWRIAFQNTEVRDQTLSRIKSNIELMVATNGGNKAVIIPHSMGVLYFLHFMKWVEAPAPMGGGGGPDWCSKYIKAVVNIGGPFLGVPKAIAGLFSAEARDIAVARTIAPGFLDNDLFRIQTLQHVMKMTRTWDSTMSMIPRGGDTIWGGLDWSPEEGYHPSQRKHSSDYTQLTDQETNQTNVVNYGRMISFGRDVAEAHSSKIEMADFRGAIKGRSVANTTCRDVWTEYHEMGFEGVRAVAEHKVYTAGSIVELLQFVAPKMMARGSAHFSYEIADNLDDPKYNHYKYWSNPLETKLPNAPDMEIFSMYGVGLPTERSYIYKLTPFAECYIPFEIDTTQDGGSDEDSCLQGGVYTVDGDETVPVLSSGFMCAKGWRGKTRFNPSGMRTYVREYDHSPPANLLEGRGTQSGAHVDIMGNFALIEDVIRVAAGAKGEDLGGDKVYSDIFKWSEKIKLPL***

**>GmPDAT2A Glyma.12G084000.1**

**MVSISKLKKFYLVEPLKSSSLGFHSFEETKKCESIVINEVEGKRQKKHRKKQCKDWRCIDYCFWMIGYMCTTWWLLSLLYGCLPAMLLGFEAPVSPGVRLSREGVTALHPVVLVPGIVTGGLELWEGRSCAEGLFRKRLWGDSFAQILKRPLCWLEHLSLHDETGLDPPGIRVRAVPGLVAADNFASGYLLWADLIENLARIGYEGKNLFMAAYDWRLSFQNTEIRDQALSRLKSHIELMFVTNGYKKVVVVPQSMGAIYFLHFLKWVETPPPMGGGSGPGWCDKYIKAIMNVSPAFLGDPRAVSNIFSTESSVGFVRTVASGILNFDYVGRQTLEHAMRVCRTWDSIISLMPKGGETIWGGLDWCLEDWNNYDQQEISWGSNSATFNLSYEAVWIDCDEMSRESIQKISKKRAYTARTVFDILNFVAPKMMKRAEAHFSHGIAENLEDPKYAHYRYWSNPLETKLPDAPDMEIYCLYGVGIPTERSHVHKFYPSEKDKSIPFQIDSSADGEDGSWLHNGVYFVDGDESVPIVSSGFMCAKGWHGRTRFNPSGTATYTIEYQLKQPGGLIDRRGLENGASSNIMGNAALIEDVLLVAAGATGVDIGGDRIFSDIMRMSERINLRL***

**>GmPDAT2B Glyma.11G190400.1**

**MVSIWRLKKFCLVEPVIVNPLSLGFQSFEETKKDESIVTNEVEGNKREKKHEWRCIDYCFWMIGYMCTTWWLLSLVYGCLPATLFGFEAPESPGVRLSREGVTALHPVVLVPGIVIGGLELWEGRSCAEGLFRKRLWVIVLFKSSKGIRVRAVPGLVAADNFASGYLLWADLIENLARIGYEGRNLFMSAYDWRLSFQNTEIRDQALSRLKSHIELMFVTNGYKKVVVVPQSMGAIYFLHFLKWVETPPPMGGGGGGPGWCDKYIKAIMNISPAFLGDPRAVSNIFSTEGSVTFVRALASGILNFDYLGRQTLERVMRVCRTWDSIISLMPKGGETIWGGLDWCLEQWNTYDQEISWGSNSATFNLSCEAVWTDYDEMSRESIQKVAKKRDYTASTVFDLLNFVAPKMMKRGEAHFSHGIAKNLDDPKYAHHKYWSNPLETKLPDAPDMEIYCLYGIGILTERSHIHKFSTSEKDKSIPFQIDSSVDREEEGSWLQNGVYYVDGDESVPIVSSGFIEYQLKQPSRFFDRTSLESGASSNIMGNAALIEDVLLVAAGATGVDIRGDRTFSDIMRMSDRIILRL***

**>AtPDAT1 AT5G13640.1**

**MPLIHRKKPTEKPSTPPSEEVVHDEDSQKKPHESSKSHHKKSNGGGKWSCIDSCCWFIGCVCVTWWFLLFLYNAMPASFPQYVTERITGPLPDPPGVKLKKEGLKAKHPVVFIPGIVTGGLELWEGKQCADGLFRKRLWGGTFGEVYKRPLCWVEHMSLDNETGLDPAGIRVRAVSGLVAADYFAPGYFVWAVLIANLAHIGYEEKNMYMAAYDWRLSFQNTEVRDQTLSRMKSNIELMVSTNGGKKAVIVPHSMGVLYFLHFMKWVEAPAPLGGGGGPDWCAKYIKAVMNIGGPFLGVPKAVAGLFSAEAKDVAVARAIAPGFLDTDIFRLQTLQHVMRMTRTWDSTMSMLPKGGDTIWGGLDWSPEKGHTCCGKKQKNNETCGEAGENGVSKKSPVNYGRMISFGKEVAEAAPSEINNIDFRGAVKGQSIPNHTCRDVWTEYHDMGIAGIKAIAEYKVYTAGEAIDLLHYVAPKMMARGAAHFSYGIADDLDDTKYQDPKYWSNPLETKLPNAPEMEIYSLYGVGIPTERAYVYKLNQSPDSCIPFQIFTSAHEEDEDSCLKAGVYNVDGDETVPVLSAGYMCAKAWRGKTRFNPSGIKTYIREYNHSPPANLLEGRGTQSGAHVDIMGNFALIEDIMRVAAGGNGSDIGHDQVHSGIFEWSERIDLKL***

**>AtPDAT2 AT3G44830.1**

**MSPLLRFRKLSSFSEDTINPKPKQSATVEKPKRRRSGRCSCVDSCCWLIGYLCTAWWLLLFLYHSVPVPAMLQAPESPGTRLSRDGVKAFHPVILVPGIVTGGLELWEGRPCAEGLFRKRLWGASFSEILRRPLCWLEHLSLDSETGLDPSGIRVRAVPGLVAADYFAPCYFAWAVLIENLAKIGYEGKNLHMASYDWRLSFHNTEVRDQSLSRLKSKIELMYATNGFKKVVVVPHSMGAIYFLHFLKWVETPLPDGGGGGGPGWCAKHIKSVVNIGPAFLGVPKAVSNLLSAEGKDIAYARSLAPGLLDSELLKLQTLEHLMRMSHSWDSIVSLLPKGGEAIWGDLDSHAEEGLNCIYSKRKSSQLSLSNLHKQNYSLKPVSRVKEPAKYGRIVSFGKRASELPSSQLSTLNVKELSRVDGNSNDSTSCGEFWSEYNEMSRESIVKVAENTAYTATTVLDLLRFIAPKMMRRAEAHFSHGIADDLDDPKYGHYKYWSNPLETKLPEAPEMEMYCLYGVGIPTERSYIYKLATSSGKCKSSIPFRIDGSLDGDDVCLKGGTRFADGDESVPVISAGFMCAKGWRGKTRFNPSGMDTFLREYKHKPPGSLLESRGTESGAHVDIMGNVGLIEDVLRIAAGASGQEIGGDRIYSDVMRMSERISIKL***

**LPCAT protein sequence**

**>GmLPCAT1 Glyma.17G131500.1**

**MSMDSMATSIGVSVPVLRFLLCFAATVPLSFLCRFLPRGLPKHLYSSVVGVALSYLSFGFSSNLHFLVPMFLGYASMLLYRPRCGILTFFLGFGYLIGCHVYYMSGDAWKEGGIDATGALMVLTLKVISCAVNYNDGLLKEEGLREAQKKYRLIKLPSLIEYIGYCLCCGSHFAGPVYEMKDYLDWTEGKGIWGTEAKGPSPSPYGATLRALLQAGFCMAMYLNLVPHFPLSKFTDPTYHEWCFWKKLSYQYMSGFTARWKYYFIWSISEASIIISGLGFSGWTDSSPPKPRWDRAKNVDIIGVEFAKSAVTIPAVWNIQVSTWLRHYVYERLIQTGKKPGFFQLLATQTVSAVWHGLYPGYIIFFVQSALMIAGSRVIYRWQQAVPPTMSLVKNVLVFTNFAYTLLVLNYSCVGFMVLSLHETLASYGSVYYIGTIVPVVMILLGKVIKPGKPARSKARKEQ***

**>GmLPCAT2 Glyma.05G049500.1**

**MAMDSMAASIGVSVPVLRFLLCFAATVPLSFLCRLLPRGLPKHLYSAAVGVALSYLSFGVSSNLHFLVPMFLGYASMLLFRPRCGILTFFLGFGYLIGCHVYYMSGDAWKEGGIDATGALMVLTLKVISCAVNYNDGLLKEEGLREAQKKYRLIKLPSLIEYIGYCLCCGSHFAGPVYEMKDYLDWTEGKGIWSTEAKGPLPSPYGATLRALLQAGFCMAMYLNLVPHFPLSKFTDPTYHEWCFWKKLSYQYMSGFTARWKYYFIWSISEASIIISGLGFSGWTDSSPPKPCWDRAKNVDIIGVEFAKSAVTIPAVWNIQVSTWLRHYVYERLIQTGKKPGFIQLLATQTVSAVWHGLYPGYIIFFVQSALMIAGSRVIYRWQQAVPPTMSLVKNVLVFTNFAYTLLVLNYSCVGFMVLSLHETLASYGSVYYIGTIIPVVLILLAKVIKPGKPARSKARKEQ***

**>AtLPCAT1 AT1G12640.1**

**MDMSSMAGSIGVSVAVLRFLLCFVATIPVSFACRIVPSRLGKHLYAAASGAFLSYLSFGFSSNLHFLVPMTIGYASMAIYRPKCGIITFFLGFAYLIGCHVFYMSGDAWKEGGIDSTGALMVLTLKVISCSMNYNDGMLKEEGLREAQKKNRLIQMPSLIEYFGYCLCCGSHFAGPVYEMKDYLEWTEGKGIWDTTEKRKKPSPYGATIRAILQAAICMALYLYLVPQYPLTRFTEPVYQEWGFLRKFSYQYMAGFTARWKYYFIWSISEASIIISGLGFSGWTDDASPKPKWDRAKNVDILGVELAKSAVQIPLVWNIQVSTWLRHYVYERLVQNGKKAGFFQLLATQTVSAVWHGLYPGYMMFFVQSALMIAGSRVIYRWQQAISPKMAMLRNIMVFINFLYTVLVLNYSAVGFMVLSLHETLTAYGSVYYIGTIIPVGLILLSYVVPAKPSRPKPRKEE***

**>AtLPCAT2 AT1G63050.1**

**MELLDMNSMAASIGVSVAVLRFLLCFVATIPISFLWRFIPSRLGKHIYSAASGAFLSYLSFGFSSNLHFLVPMTIGYASMAIYRPLSGFITFFLGFAYLIGCHVFYMSGDAWKEGGIDSTGALMVLTLKVISCSINYNDGMLKEEGLREAQKKNRLIQMPSLIEYFGYCLCCGSHFAGPVFEMKDYLEWTEEKGIWAVSEKGKRPSPYGAMIRAVFQAAICMALYLYLVPQFPLTRFTEPVYQEWGFLKRFGYQYMAGFTARWKYYFIWSISEASIIISGLGFSGWTDETQTKAKWDRAKNVDILGVELAKSAVQIPLFWNIQVSTWLRHYVYERIVKPGKKAGFFQLLATQTVSAVWHGLYPGYIIFFVQSALMIDGSKAIYRWQQAIPPKMAMLRNVLVLINFLYTVVVLNYSSVGFMVLSLHETLVAFKSVYYIGTVIPIAVLLLSYLVPVKPVRPKTRKEE***

**>LPCAT_YEAST**

**MYNPVDAVLTKIITNYGIDSFTLRYAICLLGSFPLNAILKRIPEKRIGLKCCFIISMSMFYLFGVLNLVSGFRTLFISTMFTYLISRFYRSKFMPHLNFMFVMGHLAINHIHAQFLNEQTQTTVDITSSQMVLAMKLTSFAWSYYDGSCTSESDFKDLTEHQKSRAVRGHPPLLKFLAYAFFYSTLLTGPSFDYADFDSWLNCEMFRDLPESKKPMRRHHPGERRQIPKNGKLALWKVVQGLAWMILSTLGMKHFPVKYVLDKDGFPTRSFIFRIHYLFLLGFIHRFKYYAAWTISEGSCILCGLGYNGYDSKTQKIRWDRVRNIDIWTVETAQNTREMLEAWNMNTNKWLKYSVYLRVTKKGKKPGFRSTLFTFLTSAFWHGTRPGYYLTFATGALYQTCGKIYRRNFRPIFLREDGVTPLPSKKIYDLVGIYAIKLAFGYMVQPFIILDLKPSLMVWGSVYFYVHIIVAFSFFLFRGPYAKQVTEFFKSKQPKEIFIRKQKKLEKDISASSPNLGGILKAKIEHEKGKTAEEEEMNLGIPPIELEKWDNAKEDWEDFCKDYKEWRNKNGLEIEEENLSKAFERFKQEFSNAASGSGERVRKMSFSGYSPKPISKKEE**

**>LPCAT_SCHPO**

**MLPYIDIPFQYVAAILGASSDELKLITSFLLSYPLAGVLKRIPDSKPALKNLFIIGVSAFYLVGLFDLWGGVWTLAHLYKDLLCHGLGLSSVWDTYLSTSYLANFEMNLECLLNFAGYVLFFPSLFAGPAFDYVDYESWVETTMFEVPAGTNPAKLPPTRKKRKIPRSGTPATWKAVAGIAWILAFLKLSGMYNPELVVGDQYMTRLKYYGVWALTEGSCILSGLGYKGIDPTTGKVSWDRLQNVSPWGVESAQNTRAYLGGWNINTNNWLRNYMYLRVTPRGKKPGFRASMATFTTSAFWHGFYPGYYLTFVLASFVQTVAKNCRRYFRPFFLDPKTTQPKPTKIYYDVASWLVTQTAFCFVTAPFVLLSLPASFLVWARVYFYGAVGTALATAFFSSPAKGLLIQKLKARVPAPDLKRVNSNDSIQHHEPVLGLPGEPQKDLEELVGEVRAEVELRKRKGSLGKGAIGGVGKKG**

**PAH protein sequence**

**>GmPAHα Glyma.19G175600.1**

**MNVVGKVGSLITQGVYSVATPFHPFGGAVDVIVVQQQDGTFRCTPWYVRFGKFQGVLKGAEKIVRINVNGIEANFHMYLDNSGEAYFVKEVDDDCGDKGIKSNGSPEVADNSEFRREHGGVEIHEKDNSYLSMNDTPGYRLGHSISDSGVPYLSGESRSSVLSQLQREESDVDRIFYEFPDDQSSFEGSLDVSEYESSRYENLDIENLVDSQGSQPEVVLVSVDGHVLTAPISKSEQNEDNVQLKTPQFHLGPGEETDFYEGNGEFISAENAWAADYVSQVDASTADVPANSYDTKVGDDSSGLLLEAQRKEVTICHTEEALVIKNHEDHHLQTDSEEVVSCMKRQSVFKSCLELNEFTHHQAGNADSQDVVSSLEVQNSAEESNANCSITDENEQENIKQSRNIDELSPVSGPTSLDERSSLELEVELQEVDKYAPVEVDTGSGSHSGTKDIIECNDEHVGKSVSNDLVDDSQQTSALEDSCKKSELTEPQTATSNEEDQSHSALRFEASLCGHELKAGMGLVAAAEVFEAHRISAEEFRSSALSIIKNENLVLKFRERYLLWEKAAPLVLGMTVFGLDLPVEPKDTIPVGQDDAVKAKNDAPGPASSGRRWRLWPMPFRRVKTIDHTDSVSSEEVFVDSESDWQTSVVEPSPTSARHESPRKQFVRTNVPSNEMIASLNLKDGQNLVTFSFSSRVLGTQQVDAHIYLWKWNARIVISDVDGTITKSDVLGQFMPLVGKDWSQSGVARLFSAIKENGYQLLFLSARAIVQAYLTRNFLLNLKQDGKTLPNGPVVISPDGLFPSLYREVIRRAPHEFKIACLEDIRRLFPSDYNPFYAGFGNRDTDELSYRKIGIPKGKIFIINPKGEVAISHRIDAKSYTSLHTLVNDMFPPTSLVEQEDFNSWNYWRMPFSDVD***

**>GmPAHβ1 Glyma.13G134500.1**

**MNVVGKVGSLITQGVYSVATPFHPFGGAVDVIVVQQQDGTFRSTPWYVRFGKFQGVLKGAEKFVRINVNGVEANFHMYLDNSGEAYFLKEVDDDKGVDSIEAVQDSIDKKNGYLINVHRLDHSISDSGVLRLKDESDSLVVSQLQRAESDIDRRFYEFPDDRSSLEDSVELSEYESNSYESLEGDNFGESQGSHPEMVLVSVDGHILTAPISESEQAEENVQLKTPQFHLGPGEETDLCEGNGEFSTGENAWAAGYINQLGAQTTNVQPRLCDTNGDDNTSKLLLKLCQGEEAHICEAQDTLEIKNQDHIKTDSKGAASGIKRENVFKSYLELQDFGQQAGNADLQDIGSSLEIQNSAEESNASCPVVDENEQESIAISKNGDELSPHSGSTSSNGHRSLKSELEIQEVEKNASGKIETASGSHSVTTDTEQNDEHVDKSVSNDELDESQQTPALKDVRATSEVVEPQTETSNKGDQSHLGLGFEMSLCGHELKVGMGSIAAAEVFEAHRISVVDFTSSAPSIIKNQNLVIKFKERYMTWEKAAPLVLGMAVYGLDLPVESKDTIPVEQDHALKSRDDDLGSSSSGRRWRLWPIPFRKVKTFEHTNSNSSNEEVFLDSESGSLIEPTPTSSTQGSSHKQFLRTNVPTTEQIASLNLKEGQNLVTFSFSTRVLGTQQVDAHIYLWKWNARIVISDVDGTITKSDVLGQFMPLVGKDWTQSGVARLFCAIKENGYQLLFLSARAIVQAYLTRNFLLNLKQDGKTLPNGPVVISPDGLFPSLYREVIRRAPHEFKIACLEDIKRLFPSDYNPFYAGFGNRDTDELSYRKIGVPKGKIFIINPKGEVAISHRIGAKSYTSLHTLVNDMFPPTSLVEQEDYNSWNYWKTPLPDID***

**>GmPAHβ2 Glyma.10G046400.1**

**MNVVGKVGSLITQGVYSVATPFHPFGGAVDVIVVQQQDGTFRSTPWYVRFGKFQGVLKGAEKFVRINVNGVEANFHMYLDNSGEAYFLKEVDDDKVVDSIEAVQDSIDKKNGYLSNVHRLDHSISDSGVLQLKDESDSLVLPQLQRAESDVDRRFYEFPDDRSSLEDSVELSEYESNSYESLEGDNFGESQGSHPEMVLVSVDGHILTAPISESEQTEENVQLKTPQFHLGPGEETDLCEGNGEFSTGESAWAADYINQLGAQTTNVQSRRCDTNGDDNTSKLLLEVCQGEEAHICLAQDTVEIKNQEDHMKTDSEEAASGIKRENVFKSCLELQDFGQQAGNADLQNIGSSLKIQNSVEESNASHPAVDENEQESIAISKNGDELSPPSGSASSNGHRSPKSELETQEVEKNASGEVETASGSHSVTTYSEQNDEHVDKTVTNDELDDNQQTPALKDVRATSEVVEPQTETSNKGDQSHLGLGFEISLCGHELKVGMGSVAAAEAFEAHRISVVDFTSSAPSIIKNQNLVIKFKERYMTWEKAAPLVLGMAVYSLDLPVESKDTIPVEQDHALKSRDDDLGSSSSGRRWRLWPIPFRKVKTFEHTNSNSSNEEVFLDSESGSLIEPTPASSTQGSPHKQFLRTNVPTTKQIASLNLKEGQNLVTFSFSTRVLGTQQVDAHIYLWKWNARIVISDVDGTITKSDVLGQFMPLVGKDWTQSGVARLFCAIKENGYQLLFLSARAIVQAYLTRNFLLNLKQDGKTLPNGPVVISPDGLFPSLYREVIRRAPHEFKIACLEDIKRLFPSDYNPFYAGFGNRDTDELSYRKIGIPKGKIFIINPKGEVAISQRIGAKSYTSLHTLVNDMFPPTSLVEQEDYNSWNYWKTPLPDID***

**>AtPAH1 AT3G09560.2**

**MSLVGRVGSLISQGVYSVATPFHPFGGAIDVIVVQQQDGSFRSTPWYVRFGKFQGVLKGAEKFVRISVNGTEADFHMYLDNSGEAYFIREVDPAANDTNNLISGSENNNGNQNNGVTYRLEHSLSDSGTGELREGFDPLSRLERTESDCNRRFYDFQDDPPSPTSEYGSARFDNLNVESYGDSQGSDSEVVLVSIDGHILTAPVSVAEQEAENLRLNTPQFHLAPGDGTEFCEGNTEFASSETPWDTEYIDKVEESSDTANIASDKVDAINDERNDLDSHSRDNAEKDSHDAERDLLGSCLEQSELTKTSENVKSEEPGPTFEDRNLKEGEFPLRTIMENDRSEDEVTIESIDTLVDSFESSTTQITIEEVKTTEGSRISVDSNADSECKDEQTSAETAILFNNQESSISVDSNADSECKDEQPRISAETAILINNQEGGIIESEDQDSERVSIDSTREEVDKDNEDRKTVVSVGVTSSVDEGEPDTDQRYELSLCKDELRQGMGLSAAAEVFDAHMISKEEYINSATSILESENLVVRIRETYMPWTKAARIVLGKAVFDLDLDIQPDDVISVEENESPKPKDDETTITPSSSGTRWRLWPIPFRRVKTVEHTGSNSSSEEDLFVDSEPGLQNSPETQSTTESRHESPRRQLVRTNVPTNEQIASLNLKDGQNMITFSFSTRVLGTQQVDAHIYRWRWDTKIVISDVDGTITKSDVLGQFMPFIGKDWTQSGVAKLFSAIKENGYQLLFLSARAIVQAYLTRNFLNNLKQDGKALPTGPVVISPDGLFPALYREVIRRAPHEFKIACLEDIRKLFPTDYNPFYAGFGNRDTDELSYRKLGIPKGKIFIINPKGEVATGHRIDVKKSYTSLHTLVNDMFPPTSLVEQEDYNPWNFWKLPIEEVE***

**>AtPAH2 AT5G42870.1**

**MNAVGRIGSYIYRGVGTVSGPFHPFGGAIDIIVVEQPDGTFKSSPWYVRFGKFQGVLKNGRNLIRIDVNGVDSGFNMYLAHTGQAYFLREVEDVVGESESGEVYTLSSGDEAETTSRDDVVDKVKIPLKSRSCNYDSPSPRTGNGKIVGKPGILGYVFGGRSVRESQDCGVERAEIAADLLEVKWSTNIDTRKRGKGMSSESLDGKDYGESTSTSGKSCVEGSSEMLVDSDSILETPLVASPTLRFLDEKEQDFRESTNVEDYCEEDGSSGVVVENGLCEASSMVFSVTSEGSGNVEIFVEPRTEALAEDAVSGSDLDSKQELLRAPESVEIATLGSADQADMGSIGTSQEGSSTGSPVQDENKITIKDMHISAGDFEKSQSASGESILQPEIEEEQFSFSDLDECKPGGNSSVGSSSSDTVKVDGKESYDETKTSPEKGVENTMALSEPINIERKKDIFTDEMERLVGSLPIMRLQNNDDMDASPSQPLSQSFDPCFNTSKLDLREDESSSGGLDAESVAESSPKLKAFKHVIANPEVVELSLCKHLLSEGMGAEAASQAFNSEKLDMEKFASLGPSILENDKLVVKIGGCYFPWDAAAPIILGVVSFGTAQVFEPKGMIAVDRNEKPGDVLAQGSGSWKLWPFSLRRSTKEAEASPSGDTAEPEEKQEKSSPRPMKKTVRALTPTSEQLASLDLKDGMNSVTFTFSTNIVGTQQVDARIYLWKWNSRIVVSDVDGTITRSDVLGQFMPLVGIDWSQTGVTHLFSAVKENGYQLIFLSARAISQASVTRQFLVNLKQDGKALPDGPVVISPDGLFPSLFREVIRRAPHEFKIACLEEIRGLFPPEHNPFYAGFGNRDTDEISYLKVGIPRGKIFIINPKGEVAVNRRIDTRSYTNLHTLVNRMFPATSSSEPEDFNTWNFWKLPPPSLM***

**CCT protein sequence**

**>GmCCT1 Glyma.09G051200.1**

**MEEQEECQVGETKETPTPPVRVYADGIYDLFHFGHARSLEQAKKLFPNTYLLVGCCNDEITHKYKGKTVMTEKERYESLRHCRWVDEVIPDAPWVITQEFLDKHQIDYVAHDSLPYADASGAGKDVYEYVKSVGKFKETKRTDGISTSDIIMRIIKDYNQYVMRNLDRGYTRKELGVSYVKEKRLRMNMGLKKLQERVKKQQEEVGKKIQTVGKIAGMHPNEWVENADRLVAGFLEMFEEGCHKMGTAIRDRIQERLRAQQLKSLLYDEWDDDNEFYDDDEYYTD***

**>GmCCT2 Glyma.15G157500.1**

**MEEQECQVVENKETPTPPVRVYADGIYDLFHFGHARSLEQAKKLFPNTYLLVGCCNDEITHKYKGKTVMTEKERYESLRHCRWVDEVIPDAPWVISREFLDKHQIDYVAHDSLPYADASGAGKDVYEYVKSVGKFKETKRTDGISTSDIIMRIIKDYNQYVMRNLDRGYTRKELGVSYVKEKRLRMNMGLKKLQERVKKQQEKVGKKIQTVGKIAGMHPNEWVENADRLVAGFLEMFEEGCHKMGTAIRDRIQEQLKAQQLKSLLYDEWDDDEFYDDDDEYYTD***

**>AtCCT1 AT2G32260.1**

**MSNVIGDRTEDGLSTAAAASGSTAVQSSPPTDRPVRVYADGIYDLFHFGHARSLEQAKLAFPNNTYLLVGCCNDETTHKYKGRTVMTAEERYESLRHCKWVDEVIPDAPWVVNQEFLDKHQIDYVAHDSLPYADSSGAGKDVYEFVKKVGRFKETQRTEGISTSDIIMRIVKDYNQYVMRNLDRGYSREDLGVSFVKEKRLRVNMRLKKLQERVKEQQERVGEKIQTVKMLRNEWVENADRWVAGFLEIFEEGCHKMGTAIVDSIQERLMRQKSAERLENGQDDDTDDQFYEEYFDHDMGSDDDEDEKFYDEEEVKEEETEKTVMTDAKDNK***

**>AtCCT2 AT4G15130.1**

**MSVNGENKVSGGDSSSSDRPVRVYADGIFDLFHFGHARAIEQAKKSFPNTYLLVGCCNDEITNKFKGKTVMTESERYESLRHCKWVDEVIPDAPWVLTTEFLDKHKIDYVAHDALPYADTSGAGNDVYEFVKSIGKFKETKRTEGISTSDIIMRIVKDYNQYVLRNLDRGYSREELGVSFEEKRLRVNMRLKKLQEKVKEQQEKIQTVAKTAGMHHDEWLENADRWVAGFLEMFEEGCHKMGTAIRDGIQQRLMRQESEENRRLLQNGLTISKDNDDEQMSDDNEFAEEDCVNVSNKGIETVKK**

**>PCY1_YEAST CCT**

**MANPTTGKSSIRAKLSNSSLSNLFKKNKNKRQREETEEQDNEDKDESKNQDENKDTQLTPRKRRRLTKEFEEKEARYTNELPKELRKYRPKGFRFNLPPTDRPIRIYADGVFDLFHLGHMKQLEQCKKAFPNVTLIVGVPSDKITHKLKGLTVLTDKQRCETLTHCRWVDEVVPNAPWCVTPEFLLEHKIDYVAHDDIPYVSADSDDIYKPIKEMGKFLTTQRTNGVSTSDIITKIIRDYDKYLMRNFARGATRQELNVSWLKKNELEFKKHINEFRSYFKKNQTNLNNASRDLYFEVREILLKKTLGKKLYSKLIGNELKKQNQRQRKQNFLDDPFTRKLIREASPATEFANEFTGENSTAKSPDDNGNLFSQEDDEDTNSNNTNTNSDSDSNTNSTPPSEDDDDNDRLTLENLTQKKKQSAN**

**>PCY1_PLAFK CCY**

**MDSSNYFHDCKTMLSEHNESIESSNNDINGKQKEHIKKGNSENQDVDPDTNPDAVPDDDDDDDDNSNDESEYESSQMDSEKNKGSIKNSKNVVIYADGVYDMLHLGHMKQLEQAKKLFENTTLIVGVTSDNETKLFKGQVVQTLEERTETLKHIRWVDEIISPCPWVVTPEFLEKYKIDYVAHDDIPYANNQKKKKKKKSKGKSFSFDEENEDIYAWLKRAGKFKATQRTEGVSTTDLIVRILKNYEDYIERSLQRGIHPNELNIGVTKAQSIKMKKNLIRWGEKVTDELTKVTLTDKPLGTDFDQGVENLQVKFKELFKIWKNASNKLITDFTRKLEATSYLTSIQNIIDYEIENDDYASSNFDDETSS**

**PDCT protein sequence**

**>GmPDCT1 Glyma.07G029800.1**

**MNGGAEASVNHRRRHQAASANGVKIANGAMAKPSSTLCYDASFMKWTVADAVHVATHHWMPCLFALGLLFFMAVEYTLLMVPPSSPPFDLGFIATRSLHALLESSPNLNTLFAGLNTVFVGMQTSYILWTWLIEGRPRATISALFMFTCRGILGYSTQLPLPQGFLGSGVDFPVGNVSFFLFFSGHVAGSVIASLDMRRMQRWELAWTFDVLNVLQAVRLLGTRGHYTIDLAVGVGAGILFDSLAGKYEDSKRNAALSTTHRAQFDCVNNVDIAKKINK***

**>GmPDCT2 Glyma.08G213100.1**

**MNGGAEASLNHRRKHQTAPADGAKGVKVANGAMGKPSSSKHSCGASFMKWTVADAVHVVTHHWMPCLFALGLLFFMAVEYTLLMVPPSSPPFDLGFIATRSLHALLESSPNLNTLFAGLNTVFVGMQTSYILWTWLIEGRPRATISALFMFTCRGILGYSTQLPLPQGFLGSGVDFPVGNVSFFLFFSGHVAGSVIASLDMRRMQRWELAWTFDVLNVLQAVRLLGTRGHYTIDLAVGVGAGILFDSLAGKYEDSKRNGALKHNLIA***

**>AtPDCT AT3G15820.1**

**MSAAAAETDVSLRRRSNSLNGNHTNGVAIDGTLDNNNRRVGDTNTHMDISAKKTDNGYANGVGGGGWRSKASFTTWTARDIVYVVRYHWIPCMFAAGLLFFMGVEYTLQMIPARSEPFDLGFVVTRSLNRVLASSPDLNTVLAALNTVFVGMQTTYIVWTWLVEGRARATIAALFMFTCRGILGYSTQLPLPQDFLGSGVDFPVGNVSFFLFFSGHVAGSMIASLDMRRMQRLRLAMVFDILNVLQSIRLLGTRGHYTIDLAVGVGAGILFDSLAGKYEEMMSKRHLGTGFSLISKDSLVN***

**>ScPDCT**

**MGFFIPQSSLGNLKLYKYQSDDRSFLSNHVLRPFWRKFATIFPLWMAPNLVTLLGFCFIIFNVLTTLYYDPYFDQESPRWTYFSYAIGLFLYQTFDACDGMHARRTGQQGPLGELFDHCIDSINTTLSMIPVCSMTGMGYTYMTIFSQFAILCSFYLSTWEEYHTHKLYLAEFCGPVEGIIVLCISFIAVGIYGPQTIWHTKVAQFSWQDFVFDVETVHLMYAFCTGALIFNIVTAHTNVVRYYESQSTKSATPSKTAENISKAVNGLLPFFAYFSSIFTLVLIQPSFISLALILSIGFSVAFVVGRMIIAHLTMQPFPMVNFPFLIPTIQLVLYAFMVYVLDYQKGSIVSALVWMGLGLTLAIHGMFINDIIYDITTFLDIYALSIKHPKEI**

**DAGT protein sequence**

**>GmDAGT1 Glyma.07G000300.1**

**MGERGDEAVTPAGRLFLQAEMKQVIHCVIGLKNPIDAELVKSQVRNSTMLQHPRFTSLMVRGEGGVEHWRPTEIDIDRHVLIIEEAVGEREEEDESAINKYLAELSIDSDGLSMEKPLWEIHLLKAHKCVIFRIHHALGDGISLMSMLLASCRKLNNPNALPTIAASASTSASTSASKTNLINFRNLLATLWFCFIFALEFILRCLWIRDPKSALTGGAGVELWPRKIATATFSLEDMKTVKTAANATINDVLFAVISSGISRYLDFRAPNGLRDGVQLTGLAMVNLRKHPGLQELSNMMRSNSGARWGNKFGMILLPIYYHRTNTSDPLEYLKRAKAMIDRKKRSLEASFSYKIGDFVMSTLGPKFASLLNYRILCHTSFTISNVVGPQEEIMIGGNPITFLRANNSALPHALILNMVSYAGRADMQVQVAKDIIPDPEFLAKCFEDALLEMKEQVTAKI***

**>GmDAGT2 Glyma.09G196400.1**

**MASREREGEPLSPTGKLFHEPSLNCYVIAIMGCKTSINPQVIREGLCQTLLKHPRFTSKLVKKGRKTKWIPTKVDLDNHIIVPEIDSNLEYPDRFVEDYVSHFTKTPLDQSKPLWELHLLNIKTSDAEAVSVFRIHHSIGDGASLISLLLAATRKTSDPNALPTVPIPKKDTSHQRSSSPFRWLFVIWWALLLIWHTFVDMLLFTFTIFFIKDTPTPLKAGALGVELHNKRIVHRTVSMDDIKLVKNEMKTTINDVLLGVTQAALTRYLNRAYADVGANSNGVKQRSSVLKKIRLRASILVNIRPVGGIQELADMMAEKSKVKWGNCMGYIILPFSIVLYKDPLEYVRHAKATIDRKKHSLEAICSYACAKLVLNLLGVKVAAAITRRVLFNTTVAFSNVPGPVEEISFYGHPVAYIAPSVYGHPLALTIHFQSYANNMTISLAVDPLVISDPYLLCDDLEQSLKLIRDAIQKKHTVDAV***

**>AtDAGT AT5G37300.1**

**MKAEKVMEREIETTPIEPLSPMSHMLSSPNFFIVITFGFKTRCNRSAFVDGINNTLINAPRFSSKMEINYKKKGEPVWIPVKLRVDDHIIVPDLEYSNIQNPDQFVEDYTSNIANIPMDMSKPLWEFHLLNMKTSKAESLAIVKIHHSIGDGMSLMSLLLACSRKISDPDALVSNTTATKKPADSMAWWLFVGFWFMIRVTFTTIVEFSKLMLTVCFLEDTKNPLMGNPSDGFQSWKVVHRIISFEDVKLIKDTMNMKVNDVLLGMTQAGLSRYLSSKYDGSTAEKKKILEKLRVRGAVAINLRPATKIEDLADMMAKGSKCRWGNFIGTVIFPLWVKSEKDPLEYIRRAKATMDRKKISLEAFFFYGIIKFTLKFFGGKAVEAFGKRIFGHTSLAFSNVKGPDEEISFFHHPISYIAGSALVGAQALNIHFISYVDKIVINLAVDTTTIQDPNRLCDDMVEALEIIKSATQGEIFHKTEV***

**>BnDAGT**

**MAREKQAEITIMEPLSPVSRLFVSPGFYGVIVFTLGFKTRCNSSAIVEGIKNTWIKLPRFSSKVVMDDKKNGEAVWVPVNVRVEDHVFVPDIDHSDITNPDQFIEDYTSNIANTLMDMSRPLWEFHVLNIKTSNAESLGIGKFHHSLGDGMSLMSLLYASSRKISDPNALPTTATTRKQVGSNDNWWLVARFLLMIRVIFTTFIELFKSLLTLCFMRDTKTPLMGKPGDRNGPRKIIHRIVSFDDVKFVKNTMKMKVNDVLLGITQAGLSRYLRKKYGDDPVTEKKKSLEETRLRGTIAVNLRPETKIKDLADMMTKGSKCRWGNFIGVVIFPLWIRSEDDPLEYVRRAKATMDKKKISMEALVLYGFIKFTMKIFGVKAVEAITKRVFSHTTLTFSNVLGPNEDISFFEHPMCYVGASALIGPQALIIHYVSYADKIIINLAVDTTVIPDPHVLCDNLVESLEIIKLSLLEKGLHKMEV**

**>MtDAGT**

**MACSSEGGGEPLSPAARLFHSPSFNCYVIAIIGCKTSINPQVIRDGLCQTILKHPRFTSKLVKKGRKTRWTETTIDLDNHIIVPQIDSKIDFPDRFVEDYISNFTKTPLDISKPLWELHLLNIKTSNAESIGIFRIHHSLGDGTSLISLLIAATRKTSDPNALPTVPTTRKRDDSNVHNCSIIVSFWLSILWGLRLIWNTIVDVLLLVLTILFFKDTHTPLKGAHGVELNTKRFVYLMVSMDDIKLVKAEMKTTINDVLLGLTQAGLARYLNREYGVKNANDGAAMSKSGIPKNIRLRASILVNIRASPGIQDLADMMAEKGKARWGNKMGYIIFPFNIALQEDPLEYVRQAKATIDRKKQSLEAICSYACAKLVLNLFGVKIAGVITRRVLFHTTMAFSNVAGPVEEISFYGHPVAFIAPSVYGHPHALTIHFQSYANQMTISMAVDPTIIPDPYLLCDDFEESLKLICDNVVKKRHIAEII**

**>ScDAGT**

**MRPLHPIDFIFLSLEKRQQPMHVGGLFLFQIPDNAPDTFIQDLVNDIRISKSIPVPPFNNKLNGLFWDEDEEFDLDHHFRHIALPHPGRIRELLIYISQEHSTLLDRAKPLWTCNIIEGIEGNRFAMYFKIHHAMVDGVAGMRLIEKSLSHDVTEKSIVPPWCVEGKRAKRLREPKTGKIKKIMSGIKSQLQATPTVIQELSQTVFKDIGRNPDHVSSFQAPCSILNQRVSSSRRFAAQSFDLDRFRNIAKSLNVTINDVVLAVCSGALRAYLMSHNSLPSKPLIAMVPASIRNDDSDVSNRITMILANLATHKDDPLQRLEIIRRSVQNSKQRFKRMTSDQILNYSAVVYGPAGLNIISGMMPKRQAFNLVISNVPGPREPLYWNGAKLDALYPASIVLDGQALNITMTSYLDKLEVGLIACRNALPRMQNLLTHLEEEIQLFEGVIAKQEDIKTAN**

**DAG-CPT (also called AAPT) protein sequence**

**>GmAAPT1 Glyma.12G081900.1**

**MGYIGTHGVAALHRYKYSGVDHSYVAKYVLQPFWSRFVNFFPLWMPPNMITLMGFMFLLLSALLGYIYSPQLDTAPPRWVHFAHGLLLFLYQTFDAVDGKQARRTNSSSPLGELFDHGCDALACTFEALAFGSTAMCGRTTFWWWLISAITFYGATWEHYFTNTLILPVINGPTEGLMIIYICHFFTAIVGAEWWVQQFGKSLPFLNWLPYLGGIPTFKAILCLMIAFGVTPTVTCNVSNVYKVVKGKNGSMPLALAMLYPFVVLVGGVLVWDYLSPSDIMGKYPHLVVIGTGLTFGYLVGRMILAHLCDEPKGLKTGMCMSLMFLPLAIANVLASRLNDGVPLVDERLVLLGYCAFSVTLYLHFATSVIHEITNALGIYCFRITRKEA***

**>GmAAPT2 Glyma.02G128300.1**

**MGYIGAHGVAALHRYKYSGVDHSYVAKYVLQPFWSRFVNFFPLWMPPNMITLMGFMFLLVSALLGYIYSPQLDTPPPRWVHFAHGLLLFLYQTFDAVDGKQARRTNSSSPLGELFDHGCDALACTFEALAFGSTAMCGRNTFWWWLISAITFYGATWEHYFTNTLILPVINGPTEGLMIIYICHFFTAIVGAEWWVQQFGKSLPFLNWLPYLAGIPTFKAILCLMIAFGVTPTVTCNVSNVYKVVKAKNGSMPLALAMLYPFVVLVGGVLVWDYLSPLDIMGRYPHLVVIGTGLTFGYLVGRMILAHLCDEPKGLKTGMCMSLMFLPLAIANVLASRLNDGVPLVDERLVLLGYCAFSVTLYLHFATSVIHEITNALGIYCFRITRKEA***

**>AtAAPT1 AT1G13560.1**

**MGYIGAHGVAALHRYKYSGVDHSYLAKYVLQPFWTRFVKVFPLWMPPNMITLMGFMFLVTSSLLGYIYSPQLDSPPPRWVHFAHGLLLFLYQTFDAVDGKQARRTNSSSPLGELFDHGCDALACAFEAMAFGSTAMCGRDTFWFWVISAIPFYGATWEHYFTNTLILPVINGPTEGLALIFVSHFFTAIVGAEWWAQQLGQSIPLFSWVPFVNEIQTSRAVLYMMIAFAVIPTVAFNVTNVYKVVRSRNGSMVLALAMLYPFVVLLGGVLIWDYLSPINLIATYPHLVVLGTGLAFGFLVGRMILAHLCDEPKGLKTNMCMSLLYLPFALANALTARLNAGVPLVDELWVLLGYCIFTVSLYLHFATSVIHEITEALGIYCFRITRKEA***

**>AtAAPT2 AT3G25585.2**

**MGYIGAHGVAALKKHKYSGVDHSYLAKYVLQPFWNRFVKIFPLWMPPNMITLMGFMFLLTSALLGYIYSPKLDSPPPRWVHFAHGLLLFLYQTFDAVDGKQARRTNSSSPLGELFDHGCDALGCALETMAYGSTAMCGRDTFWFWVISAVPFFGATWEHYFTNTLTLPVVNGPTEGLALIYCGHFFTAIVGAEWWAQPFGKSIPLFSWVPFLNEMQMSRIILFSMIFFAVIPTLAINTSNVYKVVHSRNGSMLLALAMLYPLVTLIAGVLIWDYLSPIDLIRNYPHLVVLGTGLAFGFLVGRMILAHLCDEPKGLKTNMCMSLLYLPFALANALTARLNDGVPLVDEFWVLLGYCIFTLSLYAHFATSVIHEITTALGIYCFRITRKEA***

**>ScAAPT**

**MGFFIPQSSLGNLKLYKYQSDDRSFLSNHVLRPFWRKFATIFPLWMAPNLVTLLGFCFIIFNVLTTLYYDPYFDQESPRWTYFSYAIGLFLYQTFDACDGMHARRTGQQGPLGELFDHCIDSINTTLSMIPVCSMTGMGYTYMTIFSQFAILCSFYLSTWEEYHTHKLYLAEFCGPVEGIIVLCISFIAVGIYGPQTIWHTKVAQFTWQDFVFDVETVHLMYAFCTGALIFNIVTAHTNVVRYYESQSTKSATPSKTAENISKAVNGLLPFFAYFSSIFTLVLIQPSFISLALILSIGFSVAFVVGRMIIAHLTMQPFPMVNFPFLIPTIQLVLYAFMVYVLDYQKESIVSALVWMGLGLTLAIHGMFINDIIYDITTFLDIYALSIKHPKEI**

**>ZmAATP**

**MGGYIGQHGIAALHKYKYSGVDNSLVAKYILQPFWSRCVNLFPLWMPPNMITLTGFVFLMTSALIGYIYSPHLDTAPPRWVHLAHGLLLFLYQTFDAVDGKQARRTNSSSPLGELFDHGCDALACAFESLAFGSTAMCGATTFWFWVISAIPFYLATWEHFFTNTLILPVINGPTEGLMMIYISHFLTFFLGGEWWAQSFRKSIPFMNWVPLVPEIPFYGVVLFLMIIFGVIPTIGANISNVSKVVKARKGSMMLALAMLFPFVVLLAGVLSWSFLSPEDIMGNYPHLLVVGTGFAFGFLVGRMILAHLCDEPKGLKTGMCMSLLYLPLAVANALTAKLNGGIPLFDEWLVLVGFCVYTVSLYVHFSTTVIHEITNALGIYCFRITRKEA**

**>RcAAPT**

**MVTRNEVKERMGYIGTHGVAALHRYKYSGVDHSYVAKYVLQPFWTRFVNFFPLWMPPNMITLTGFMFLLTSALLGYIYSPHLDTAPPRWVHFAHGLLLFLYQTFDAVDGKQARRTNSSSPLGELFDHGCDALACAFESMAFGSTAMCGRDTFWFWVISAVPFYGATWEHFFTNTLILPAVNGPTEGLMLIYVAHFFTALVGAEWWPQHFGKSFPFLNWVPFISEIQTYKAVLYLMIAFAVMPTIAFNVSNVYKVVQARKGSMLLALAMLYPFVVLMGGVLVWDYLSASNLMESYPHLVILGTGLAFGFLVGRMILAHLCDEPKGLKTNMCMSLLYLPFAIANALTARLNDGLDGNFAITSFLCRVPLVDEFWWDSIYTLQHLSFMKSRQPWALIASEGRFEVVLSYLDVIRTQIKNNKERSLKNAVIDLASMSGGMKDIDD**

**PLA protein sequence**

**>AtsPLA2-α AT2G06925.1**

**MAAPIILFSFLLFFSVSVSALNVGVQLIHPSISLTKECSRKCESEFCSVPPFLRYGKYCGLLYSGCPGERPCDGLDSCCMKHDACVQSKNNDYLSQECSQKFINCMNNFSQKKQPTFKGNKCDADEVIDVISIVMEAALIAGKVLKKP***

**>AtsPLA2-β AT2G19690.2**

**MMFRTSLMRFAAAFFAIVFVVLVGVARSEECTRTCIAQNCDTLSIRYGKYCGIGHSGCPGEEPCDDLDACCKIHDHCVELNGMTNISCHKKFQRCVNRLSKAIKQSKNKKVGFSTKCPYSVVIPTVNQGMDIGILFSQLAYHRKFVTD***

**>AtsPLA2-γ1 AT4G29460.1**

**MITGLALSRVAFGLTAFLLLAVVSSQEKCSNTCIAQNCNSLGIRYGKYCGIGYFGCPGEPPCDDLDACCMTHDNCVDLKGMTYVNCHKQFKRCVNKLSKSIKHSNGEKIGFSTQCPYSIVIPTVFNGMDYGIFFSGIGNIFNPPVLGSVPVVEVDLARSKVDTKDGLGTKLGLQTKEGSKVSASLNI***

**>AtiPLA2-β1 AT2G44810.1**

**MEYQGLQNWDGLLDPLDDNLRREILRYGQFVESAYQAFDFDPSSPTYGTCRFPRSTLLERSGLPNSGYRLTKNLRATSGINLPRWIEKAPSWMATQSSWIGYVAVCQDKEEISRLGRRDVVISFRGTATCLEWLENLRATLTHLPNGPTGANLNGSNSGPMVESGFLSLYTSGVHSLRDMVREEIARLLQSYGDEPLSVTITGHSLGAAIATLAAYDIKTTFKRAPMVTVISFGGPRVGNRCFRKLLEKQGTKVLRIVNSDDVITKVPGVVLENREQDNVKMTASIMPSWIQRRVEETPWVYAEIGKELRLSSRDSPHLSSINVATCHELKTYLHLVDGFVSSTCPFRETARRVLHR***

**>AtcPLA2 AT4G19860.1**

**MSLLLEEIIRSVEALLKLRNRNQEPYVDPNLNPVLLVPGIAGSILNAVDHENGNEERVWVRIFGADHEFRTKMWSRFDPSTGKTISLDPKTSIVVPQDRAGLHAIDVLDPDMIVGRESVYYFHEMIVEMIGWGFEEGKTLFGFGYDFRQSNRLQETLDQFAKKLETVYKASGEKKINVISHSMGGLLVKCFMGLHSDIFEKYVQNWIAIAAPFRGAPGYITSTLLNGMSFVNGWEQNFFVSKWSMHQLLIECPSIYELMCCPYFKWELPPVLELWREKESNDGVGTSYVVLESYCSLESLEVFTKSLSNNTADYCGESIDLPFNWKIMEWAHKTKQVLASAKLPPKVKFYNIYGTNLETPHSVCYGNEKMPVKDLTNLRYFQPTYICVDGDGTVPMESAMADGLEAVARVGVPGEHRGILNDHRVFRMLKKWLNVGEPDPFYNPVNDYVILPTTYEFEKFHENGLEVASVKESWDIISDDNNIGTTGSTVNSISVSQPGDDQNPQAEARATLTVQPQSDGRQHVELNAVSVSVDA***

**>GmsPLA2-XIB-1 Glyma.01G002400.1**

**MVPTPQLKYVLLLFYCTFAFNLLSTPACALNIGAETTGVAVSVSKECSRQCESSFCSVPPLLRYGKYCGLLYSGCPGERPCDGLDACCMKHDQCVSAKNNDYLSQECSQTFINCMNNFKNSRAPTFKGNTCDADDVIEVIHVVMEAALLAGRVLHKP***

**>GmsPLA2-XIB-2 Glyma.07G129900.1**

**MVPAPSFKYVLFFCCTFAFNLLSTPVRALNIGAETTGVAVSVGKECSRQCESSFCSVPPLLRYGKYCGLLYSGCPGERPCDGLDACCMKHDQCVSAKNNDYLSQECSQTFINCMNNFKNSKAPTFKGNTCDVDDVIEVIHVVMEAALLAGRVLHKP***

**>GmsPLA2-XIB-3 Glyma.08G028800.1**

**MVPSQLSKYGLLFISCTFFLINFLTIPISSLNIGVETTGITVSVSKECSRTCESSFCSVPPLLRYGKYCGLLYSGCPGEKPCDGLDACCMYHDKCVQAKNNDYLSQECSQTFINCMQKFKNSRAPTFKGNACQVDDVIEVINVVMEAALLAGRVLHKP***

**>GmsPLA2-XIA-1 Glyma.01G004000.1**

**MSRAAASFGILLCLFLAAAAVVNCSDQANCSTTCIAEQCDTVGIKYGKYCGVGYWGCAGEKPCDDLDACCMAHDDCVDKFGMTHVKCHKKLKNCLTRELKSGKVGFSKECPYSRAAPTMIRGMDLAILLSQLGDSVPH***

**>GmsPLA2-XIA-2 Glyma.07G127900.1**

**MSRAAASFGILLCLLLLVAAVNCSDQGNCSTTCIVEQCDTIGIKYGKYCGVGYWGCAGEKPCDDLDACCMAHDNCVDKFGMTHVKCHKRLKNCLTRELKSGKVGFSKECPYSRAAPTMIRGMDLAILLSQLGDFSVPH***

**>GmsPLA2-XIC-1 Glyma.07G232000.1**

**MDFLGKIPWFNAQVNTDLATNSIPIETFTEQPKQELGNDPKLPFLSLFPWGNRAGEKFQRPSTINKELKRQARCGNGVGKDGEATPSRFRPYVCQVPWHTGVRAFLSQLFPRYGHYCGPNWSSGKDGGSLVWDRRPIDWLDFCCYCHDIGYDTHDQAKLLKADLAFLECLEKQHGSTKGDPHVAHLYKTMCVNGLRNFLIPYRRNIVNLQQFGQPMIQFGWLSNLRWGGWNFQKTHRLSSLGGSTVS***

**>GmsPLA2-XIC-2 Glyma.20G041000.1**

**MFGCFLLFLLAGYKKIWFCGSYLCTLVTLVFSTMDLGFLGKIPWFNAQLNTDSGSNSVPIDTFTEQPKQELGNDSKLPFLSLFPWVNRAGDKFQRPSTINKELKRQARRRNGVGKDGEVNPLRFRPYVCKVPWHTGVRAFLSQLFPRYGHYCGPNWSSGKDGGSLVWDRRPIDWLDFCCYCHDIGYDTHDQAKLLKADLAFLECLEKHHGSTKGDPHVAPIYKTMCLNGLRNFLIPYRRNIVNLQQFGQPMIQFGWLSNLRWGSWNFQKTHRLSSVGGSTVS***

**>GmiPLA2-XIA-1 Glyma.18G251500.1**

**MRTLHNYTPTLTRPQCTTLVQSTKPLQKMEKMMNMPQLRSSSLPPLSKKVGKRWKEYQGMNNWDGLLDPLDENLRAEILRYGHFVEAAYKSFEFDPSSPNYATCKFPKNTLFEKSGLHNTGYKVTKHLRATSGIKLPSWVDKAPSWVAAQSSYVGYVAVCNDKEEIKRLGRRDIVVAYRGTTTCLEWLENLRATLTHVSVPSITTETTTEPCSMEENGAMVESGFLSLYTSTVSNNKSFMSLQDMVRKEIGRIRKTYQGENLSLTITGHSLGAALATLTAYDIKNSFLQPPPLVTVISFGGPRVGNRSFRRRLEEQGTKVLRIVNSDDVITKVPGFVFDDVDKTEDVAACNGGVQVAKFQRWIRKRAEEVQWLLYSEVGKELRLCSRDSPYLRGVNIATSHDLNTYLHLVDGFVSSTCPFRATAKRFLQH***

**>GmiPLA2-XIA-2 Glyma.09G243100.1**

**MRTLHYYTSTLIRPQCTTLVQTTKPVQKMEKMMNMPQLQSSSLSSSPPLSKKVGKRWKEYQGMNNWDGLLDPLDENLRAEILRYGHFVEAAYKSFEFDPSSPNYATCKFQKNTLFEQCGLRNTGYKVTKHLRATSGIKLPSWVATQSSYVGYVAVCNDKEEIKRLGRRDIVVAFRGTATCLEWLENLRATLTHVSVPSVATGITAEPCSMDGNGAMVESGFLSLYTSAGSSKQSFTSLQDMVRKEIGRILKTYEGENLSLTITGHSLGAALATLTAYDIKNSFIRQPPVTVISFGGPRVGNRSFRRQLEETGIKLLRIVNSDDVITKVPGFVFDDVDKTDDDVACNGGAHVVQRWIRKRAEEVQWLLYSEVGKELRLCSRDSPYLRGVNIATCHDLNTYLHLVDGFVSSTCPFRATAKRFLQH***

**>GmiPLA2-XIB-1 Glyma.18G251600.1**

**MKFTIKTPHPLPSPQSNTLLKPRCIITTTQTSKPTNNKNLHFSNDTRQAALHLEKMINLEEHNNYSNYFPSMQSSKLGKRWKEYHGMSNWDGLLDPLDDNLRAEILRYGHFVETVYKSFEFDPSSPNYANSRFPRKDLLERCGLHNTGYKVTKYLRATSGIQLPSWVDKAPTWVAKQTSYVGYVAVCHDKEEIKRLGRRDVVVAYRGTTTCLEWLENFRASLTNLPIPCNTKRAFEKNGVMDRSGAMVESGFLSLYTSSLPRKTFRSLQEMVRREISRILETYRGEQLSLTVTGHSLGAALATLTAYDVKTAFPGLPVTVISFGGPRVGDPRFRRMLERQGTKVLRIVNSDDVITKVPGFVFDDGLASDGGVHVPGFPRWIQKRVEEAQLVYAEVGRELRLCSKDSPYLGNTNVATCHELNTYLHLVDGFVSSTCPFRASAKRFLQR***

**>GmiPLA2-XIB-2 Glyma.09G243000.1**

**MKLTIKTPHPLPSPQSNTLLKPRCITTTTQTSKPTSNKNLHMSNDTRQAALRLEKMINLEEEHNTNNYFPSMQSSKLGKRWKEYHGMSNWEGLLDPLDDNLRAEILRYGHFVETAYKSFEFDPSSPNFANSRFPKKALLERCGLPKTRYKVTKYLRATSGIQLPSWVDKVPRWVAKQTSYVGYVAVCHDKEEIKRLGRRDVVVAYRGTTTCLEWLENFRASLTNLPIPCSSKRAFEKNGVMDGSGAMVESGFLSLYTSSLPAKVSLQEMVRREISRILDTYRGEQLSLTVTGHSLGAALATLTAYDVKTAFPELPVTVISFGGPRVGDRRFRRQLERQGTKVLRIVNSDDVITKLPGFVFDDDVASAGGVHVAGFPSWIQKRVEEAQLVYAEVGKELRLCSRDSPYLGNTNVATCHELNTYLHLVDGFVSSTCPFRASAKRFLQR***

**>GmcPLA2-XIB Glyma.18G244500.1**

**MRFCPCFGSEEAKGVADRDPVLLVSGMGGSIVNSKPKKFGFTTRVWVRLLLADVEFRNKIWSLYNPQTGYTETLDKKSEIVVPDDDHGLYAIDILDPSWFTKCIHLTEVYHFHDMIDMLVGCGYNKGTTLFGYGYDFRQSNRIGKVMEGLKSKLETAHKASGGRKVNLISHSMGGIMISCFMSLYRDVFTKYVNKWICLACPFQGAPGCINDSLLTGLEFVDGFQSYFFVKRWTMHQLLVECPSIYEMLANPYYEWKKQPEILVWRKHTKDGDNNINLESYGPTQSISLFEEALRDNEVNYKGKTISLPFNFDILDWAVETRQLIANAKLPDGVCFYNIYGTSLDTPFDVCYGSENSPIEDLSEICHTMPLYSYVDGDGTVPSESAKGDGLEATERVGVAASHRGILRDETVFQHIQKWLGVEPMVGKHSKTSKVADAQPMVL***

**>GmcPLA2-XIA-2 Glyma.20G020800.1**

**MAILLGEILQSLELWLKLIKNPQPQPYVNPNLDPVLLVPGVGGSMLHAVDESEGSRERVWVRFLNAEYTLKTKLWSRYDPSTGKTESMDPNSRIMVPEDRHGLHAIDILDPDLMLGSDSVYYFHDMIVEMRKWGFEEGKTLFGFGYDFRQSNRLQETMDRLAAKLESIYNAAGGKKINIITHSMGGLLVKCFMCLQSDIFEKYVKNWVAICAPFQGAPGTINSTFLNGMSFVEGWEQNFYISKWSMHQLLIECPSIYELMGCPNSHWKHIPALELWRERHDSDGKSHIVLESYPPCDSIKVLEQALVNNIVNYNGEDLPLPFNFEILKWANKTWEILSSAKLPSQVKFYNIYGTSLETPHSVCFGSGDKPVTDLQQLRYFQAKYVCVDGDGTVPIESAKADGLNAEARVGVPGEHQRILREPHVFRLLKHWLKAGEPDPFYNPVNDYVILPTAFEMERHKEKGVEVASLKEEWEIISKVQDDQSSTADKVCSISVSQEGANQSYSEAHATVIVHPDSEGKQHVQLNALAVSVDAS***

**>GmcPLA2-XIA-1 Glyma.07G221800.1**

**MAILLGEILQSLELWLKLIKNPQPQPYVNPNLDPVLLVPGVGGSMLHAVDETDGSHERVWVRFLNAEYTLKTKLWSRYDPSTGKTESMDPNSTIIVPEDRHGLHAIDILDPDLMFGSDSVYYFHDMIVEMRKWGFEEGKTLFGFGYDFRQSNRLKETMDRLAAKLESIYNAAGGKKINIITHSMGGLLVKCFMCLQSDIFEKYVKNWVAICAPFQGAPGTIYSTFLNGMSFVEGWEQNFYISKWSMHQLLIECPSIYELMGCPNSHWQHIPVLELWRERRDSDGKSHIVLESYPPCDSIEVLKQALLNNTVNYNGVDLPLPFNLEILKWANKTWEILSSAKLPSQVKFYNIYGTSLDTPHSVCFGSGDKPVTDLQQLCYFQAKYVCVDGDGTVPIESAKADGLNAEARVGVPGEHQRILREPHVFRLLKHWLKAGEPDPFYNPVNDYVILPTAFEMERHKEKGVEVASLKEEWEIISKVQDDQSCTADKVCSISVSQEGANQSYSEAHATVIVHPDNEGKQHVQLNALAVSVDAS***

**>GmPLA1-Ibeta9 Glyma.11G036900.1**

**MMQISSTVPAHKLHKFQAIRCPSFSFRCQQASSSSLKQPSIFQTKPFISTESTRLHLANLDKLLETQKPVVPPTQIQHQPIINDPKEKKGRSFLEGLDLGRLWPEMKATDEMSPRHLKRLQRLLSMTGEYSPRNILGGRWREYHGSNDWKGMLDPLDENLRREVVRYGEFVQAAYQAFHSDPAMSTEEPPHPQHVALPDRSYRMTKSLYATSSIGLPKWVDEVAPDLGWMTQRSSWVGYVAVCEDRREIARMGRRDIIISLRGTSTCMEWAENLRAHMVEMGDEEGKAKVECGFMSLYKTKGAQVASLAESVVEEVRRLIDLYRGEELSISVIGHSLGATLALLVADEISTCCPKVPPVAVFSFGGPRVGNKAFGDRLTAKNVKVLRIVNSQDVITRVPGIFVSEELEQKIRNVGGGVLEENTPLAYSHVGTELRVQTKMSPYLKPDADMACCHDLEAYLHLVDGFLASNCPFRSNAKRSLARLMQDQSANVKKLYTSKAKSLTVNLSRQGSMSMSSCLSSPS***

**>GmPLA1-Ibeta7 Glyma.17G145900.1**

**MMQISSTIPAPNLHMFQTRRTSFRCRASPLNPTTSSSPQSIKSVSDSTRLHLSNLDNLLQKQSPTTQPKQQEELTLAATIQNNKTTTTEKKGKNVLEGLNLARLWPDMKATEEMSPRHLNRLQRLLSKTDEYSPRNTLGSLWREYHGSHDWKGMLDPLDENLRREVVRYGEFVQAAYHSFHSNPAMSAEEPPLPRHMVLPDRSYRITKSLYATSSIGLPKWVDDVAPDLGWMSQRSSWVGYVAVCDDRREIVRLGRRDIVISLRGTATCLEWVENMRAQLINIDSSSSSRGKPKVECGFLSLYKTRGSHVPSLKESVIEEVKRLMKLYQGETLSITITGHSLGAALALLVADDVSMCSTDVPPVAVFSFGGPRVGNRAFGDKLAAQNVKVLRIVNSQDVITKVPGMLVSEEVEKKLRNSKLGAGVLDIFDEYSHTGTELRVDTKMSPFLKPDADMACCHDLEAYLHLVDGFLASNCPFRANAKRSLARLMQDQGANVKKLYTSKAKALSLNLQRQASFSISGCLPSPS***

**>AtiPLA2-1 AT2G44180.1**

**MAIGNPEVATMGKENTEAESSNGNESQLSSDLTKSLDLAEVKEDEKDNNQEEEDGLKAEASTKKKKKKSKSKKKKSSLQQTDPPSIPVLELFPSGDFPQGEIQQYNDDNLWRTTSEEKREMERLQKPIYNSLRQAAEVHRQVRKYMRSILKPGMLMIDLCETLENTVRKLISENGLQAGIAFPTGCSLNNVAAHWTPNSGDKTVLQYDDVMKLDFGTHIDGHIVDSAFTVAFNPMFDPLLAASRDATYTGIKEAGVDVRLCDVGAAVQEVMESYEVEINGKVYQVKSIRNLNGHSIGRYQIHAEKSVPNVRGGEQTKMEEGELYAIETFGSTGKGYVREDLECSHYMKNYDVGHVPLRLPRAKQLLATINKNFSTLAFCRRYLDRLGETKYLMALKNLCDSGIIEPCPPVCDVKGSYISQFEHTILLRPTCKEIISKGDDY***

**>AtcPLA2 AT4G19860.1**

**MSLLLEEIIRSVEALLKLRNRNQEPYVDPNLNPVLLVPGIAGSILNAVDHENGNEERVWVRIFGADHEFRTKMWSRFDPSTGKTISLDPKTSIVVPQDRAGLHAIDVLDPDMIVGRESVYYFHEMIVEMIGWGFEEGKTLFGFGYDFRQSNRLQETLDQFAKKLETVYKASGEKKINVISHSMGGLLVKCFMGLHSDIFEKYVQNWIAIAAPFRGAPGYITSTLLNGMSFVNGWEQNFFVSKWSMHQLLIECPSIYELMCCPYFKWELPPVLELWREKESNDGVGTSYVVLESYCSLESLEVFTKSLSNNTADYCGESIDLPFNWKIMEWAHKTKQVLASAKLPPKVKFYNIYGTNLETPHSVCYGNEKMPVKDLTNLRYFQPTYICVDGDGTVPMESAMADGLEAVARVGVPGEHRGILNDHRVFRMLKKWLNVGEPDPFYNPVNDYVILPTTYEFEKFHENGLEVASVKESWDIISDDNNIGTTGSTVNSISVSQPGDDQNPQAEARATLTVQPQSDGRQHVELNAVSVSVDA***

**>AtsPLA2-AT2G06925.1**

**MAAPIILFSFLLFFSVSVSALNVGVQLIHPSISLTKECSRKCESEFCSVPPFLRYGKYCGLLYSGCPGERPCDGLDSCCMKHDACVQSKNNDYLSQECSQKFINCMNNFSQKKQPTFKGNKCDADEVIDVISIVMEAALIAGKVLKKP***

**>AtsPLA2-AT2G19690.2**

**MMFRTSLMRFAAAFFAIVFVVLVGVARSEECTRTCIAQNCDTLSIRYGKYCGIGHSGCPGEEPCDDLDACCKIHDHCVELNGMTNISCHKKFQRCVNRLSKAIKQSKNKKVGFSTKCPYSVVIPTVNQGMDIGILFSQLAYHRKFVTD***

**>AtsPLA2-1 AT4G29460.1**

**MITGLALSRVAFGLTAFLLLAVVSSQEKCSNTCIAQNCNSLGIRYGKYCGIGYFGCPGEPPCDDLDACCMTHDNCVDLKGMTYVNCHKQFKRCVNKLSKSIKHSNGEKIGFSTQCPYSIVIPTVFNGMDYGIFFSGIGNIFNPPVLGSVPVVEVDLARSKVDTKDGLGTKLGLQTKEGSKVSASLNI***

**CEK protein sequence**

**>GmCEK1 Glyma.02G078500.1**

**MAIKAIELLKGSGNHEEIFEVLAAVAASLGDVIDDVNTLQVTPLKGAMTNEVFEVNWPTKSDGHQRRVLVRLYGEGVEVFFNRVDEIQTFECMSKHGQGPRLLGRFTTGRVEEFIHAKTLSAADLRDPEISALIASKMREFHNLHMPGAKKAQLWQRMRKWLSHAKSLCSPKETKNFGLDNLDAEINMLVELLSQGNQQIGFCHNDLQYGNIMMDEDTRAITLIDYEYASYNPIGYDLANHFCEMVANYHSDEPHVLDYSKYPGLEERQRFVYNYLSSEGKKPSNSEVDQLVNLAEKYTLANHLFWGLWGLISSHVNTIDFDYKEYARQRFQQYWLKKPTLLDSPSIVSQDGIANGVNH***

**>GmCEK2α Glyma.13G189900.1**

**MGATEEALQNLVNVNVVDAENPKNIKVSGTEDSVVNDKELGAKETPIKDKKDSIPGEVKEMLKSLASEWENVVDINALQVIPLKGAMTNEVFQIKWQTTAGESSRKVLLRTYGEGTGIFFDRDVETLSASDLRDPSISALIAAKLKEFHDLDMPGPKTVNLWDRLRNWLSEAKRLCSPEEAEAFHLDTMDKEISALENFLSDTHQRIGFCHNDLQYGNIMFDEESSSVTIIDYEYANYNPVAYDIANHFNEMAANYHTDTPHVLDFTKYPDLEERRRFAHAYLSSSGEQPSDTEEHVNKIDFDYKEYAKQRLQEYWSRKTCLLGSHEYSSHDNATKVNGEQTLTSTTNRKPAKRNSVSNKLKKIFGLGFFRSKH***

**>GmCEK3α1 Glyma.20G170300.1**

**MAIKAIELLKGCGSQEEIMEVLAAVASDLGDVIDDVNTLQVIPLNGAMTNEVFQINWPTKNGGEIRKVLVRLYGEGVEVFFDREEEIRNFDCISKHGQGPRLLGRFTSGRVEEFIHARTLSAADLRDPEVSALIASKMREFHNLHMPGAKKVQIWHRMRKWLGQAKSLCSPKDEKNFGLDNLDEEINILEKKLSEGYQEIGFCHNDLQYGNIMMDEETRLITIIDYEYASYNPIAYDLANHFCEMVADYHSDTPHVLDYTKYPGLEERQRFIRNYLSSEGNKPSNAKVNQLAKAAEKYTLANHLFWGLWGLISSYVNKIDFDYKEYARQRFQQYWIRKPTLLDSPSIVSLDETVNGLMPSFT***

**>GmCEK3α2 Glyma.10G221200.1**

**MAIKTMELLKGCGSQEEIMEVLSAVASDLGDVIDDVNTLQVIPLNGALTNEVFQINWPTKNDGEVRKVLIRLYGEGVEVFFDREEEIRTFECISKHGQGPRLLGRFTSGRVEEFIHARTLSAADLRDPEVSALIASKMREFHNLHMPGAKKAQIWHRVRKWLGQAKSLCSPKDAKKFGLDNLDEEINILEKKLSEGYQEIVFCHNDLQYGNIMMDEETRLITIIDYEYAGYNPIAYDLANHFCEMVADYHSDTPHVLDYKKYPGLEERQRFIRNYLSSEGNKPSNAKVNQLVKAAEKYTLANHLFWGLWGLISSYVNKIDFDYKEYGRQRFQQYWIRKPTLLDSPSIVSLDETVNGLLSSFT***

**>GmCEK4α1 Glyma.04G148300.1**

**MGAEVKIWNPVEVAEQARHDYASQIHSSHLTIDPSLELPLMAPLVLKLCKDMFKAWSNLDDSRFVVEKISGGITNLLLKVSVKQENCIEETITVRLYGPNTEYIIDRQRELQATKYITAAGFGAKWLGIFGNGMVQSFINAQTLSPSDMREPKLASKIAKQLQRFHHVEIPGSKEPQLWNDVWKFFEKASVLEFDDSKMQKTYETISFKEVHDEIVELKGLCDLLKSPVIFAHNDLLSGNIMINYEEDKLYFIDYEYASYNYRGYDIGNHFAEYAGFECDYDLYPNMNEQYHFLRHYLKPERPQEVSEKDLETLYVEANTFSLASHVFWALWGLIQAKMSPIEFDYLGYFFLRYHEYKRQKEKYFLLARSYLSGCKNE***

**>GmCEK4α2 Glyma.06G216800.1**

**MGAEVKIWNPVEVAEQARHDYASQIHPSHLTIDPSLELPQMTPLVLKLCKDMFKAWSNLDDSCFVVEKISGGITNLLLKVSVKQENCIEETITVRLYGPNTEYIIDRQRELQATKYITAAGFGAKWLGIFGNGMVQSFINAHTLSPSDMREPKLAAKIAKQLQRFHHVEIPGSKEPQLWNDVWKFFEKASVLEFDDSKMQKTYETISFKEVHDEIVELKGLCDLLKSPVIFAHNDLLSGNIMMNCEEDKLYFIDYEYASYNYRGYDIGDHFAEYAGFECDYDLYPNMNEQYHFLRHYLKPERPQEVSEKDLETLYVEANTFSLASHIFWALWGLIQAKMSAIEFDYLGYFFLRYHEYKRHKEKYFLLAQSYLSGCKNEMSR***

**>GmCEK4β1 Glyma.06G309300.1**

**MGAAEDLVNSTPGAAESKANHTPGPSENPIKDNTSTSENPVKDKALGAEIPEAGGAENPGSDQKNVEQNPASEQVSIAENMVNDQAGVTDNTAKEQVAGSAEDPTKNQAAGGAEDPPKDQVAGGAENPLNDLIDLAQNPDSEQAGSEENLLNNQAGGVENSAKNHAAAGAGDPPNDQAADGSENSAKNISARSAGDPPHDQTADGIENSLSNQSEATEKLVNDEQGPAESFVEDEVHVIETPEKCQKYGVETPISSKAERLPEEAKEILKSLASKWEDVFDANALQVIPLKGAMTNEVFQIKWPTTTGELSRKVLVRMYGEGVDVFFNRDNEIQTFEFMSKNGQGPRLLGRFMNGRVEEFIHARTLSASDLRDPSISALIATKMKEFHDLDMPGEKKVHLWDRLRNWFSEAKRLSSPKEFEAFYLDTIDKEISILEKELSGPHQRIGFCHNDLQYGNIMLDEETNSVTIIDYEYASYNPVAFDIANHFCEMAANYHTEEPHILDYNKYPDFEERQRFVQAYLSTSGEQLSNSEVEQLLQEIEKYTLANHLFWGVWGIISAQVNTIDFDYKEYAKQRFQEYWARKPYLLINSEAPSPYNVPEGTGELASALPTKSKNSGIFRKMKRVLGLGLFKSKS***

**>GmCEK4β2 Glyma.04G132000.1**

**MGATEDLVNSTQGAAECQENRTTGPSENPIKDNTSSTSENLVQYKALDAENPVSDQQGGAEIPETGGAENPASDQKNVEQIPASEQVGIAENMVNDQAGGAEDPTKDRTAGGAENPLNDLIFLAQNSESEQAGSVENLLSDQAGVAEDHTKNRAAGSAENPSNDLIDLAQNPESEQAGSEENLLNYQAGGVENSAKNHAAAGAGDPPNDQAADGAENSAKNLSARSAGDPPHDQAADAIENSLNNQTEATEKLVKDEQGTAESFVEDVADAVEMPENSQRNGVETPVSNKADRLPEEAKEILKSLASKWEDVLDANALQVIPLKGAMTNEVFQIKWPTMTGELSRKVLVRMYGEGVDVFFDRDNEIHTFEFMSKNGQGPRLLGRFTNGRVEEFIHARTLSASDLRDPSISALIAAKMKEFHDLDMPGEKKVHLWDRLRNWLSEAKRLSSPKEVEAFYLDTIDKEISILEKELSGSHQQIKFCHNDLQYGNIMLDEETNSVTIIDYEYASYNPVAFDIANHFCEMAANYHTEEPHILDYNKYPDFEERQRFVQAYLSTSGEQPSDSEVEQLLQEIEKYTLANHLFWGVWGIISAQVNTIDFDYKEYAKQRFQEYWARKPYLLINSEAPSPYNVPEGTGELASAVPTKSKNSGIFRKMKRVLGLGLFRSKS***

**>AtCEK1 AT1G71697.1**

**MAIKTKTSLIPSCSSPEDLKRVLQTLGSSWGDVVEDLERLEVVPLKGAMTNEVYQINWPTLNGEDVHRKVLVRIYGDGVDLFFNRGDEIKTFECMSHHGYGPKLLGRFSDGRLEEFIHARTLSADDLRVAETSDFIAAKLREFHKLDMPGPKNVLLWERLRTWLKEAKNLASPIEMDKYRLEGLENEIN**

**LLEERLTRDDQEIGFCHNDLQYGNVMIDEVTNAITIIDYEYSSFNPIAYDIANHFCEMAANYHSDTPHVLDYTLYPGEGERRRFISTYLGSTGNATSDKEVERLLKDAESYTLANHIFWGLWGIISGHVNKIEFDYMEYARQRFEQYWLRKPLLLEG***

**>AtCEK2 AT1G74320.1**

**MTMGGTEKNVENKQYRLPREVKEALQAIASEWEDVIDSKALQVIPLKGAMTNEVFQIKWPTREKGPSRKVLVRIYGEGVEIFFDREDEIRTFEFMSKHGHGPLLLGRFGNGRIEEFLHARTLSACDLRDPEISGRIATRMKEFHGLEMPGAKKALLWDRLRNWLTACKRLASPEEAKSFRLDVMEMEINMLEKSLFDNDENIGFCHNDLQYGNIMMDEETKAITIIDYEYSCYNPVAYDIANHFCEMAADYHTETPHIMDYSKYPGVEERQRFLKTYMSYSDEKPSDTMVKKLLEDVEKYTLASHLIWGLWGIISEHVNEIDFDYMEYARQRFEQYWLTKPRLLAASEHK***

**>AtCEK3 AT4G09760.2**

**MAVGIFGLIPSSSPDELRKILQALSTKWGDVVEDFESLEVKPMKGAMTNEVFMVSWPRKETNLRCRKLLVRVYGEGVELFFNRDDEIRTFEYVARHGHGPTLLGRFAGGRVEEFIHARTLSATDLRDPNISALVASKLRRFHSIHIPGDRIMLIWDRMRTWVGQAKNLCSNEHSTEFGLDDIEDEINLLEQEVNNEQEIGFCHNDLQYGNIMIDEETNAITIIDYEYASYNPIAYDIANHFCEMAADYHSNTPHILDYTLYPGEEERRRFICNYLTSSGEEAREEDIEQLLDDIEKYTLASHLFWGLWGIISGYVNKIEFDYIEYSRQRFKQYWLRKPKLLSFFPS***

**>AtCEK4 AT2G26830.1**

**MGAAKNIWALANAEDAANDAEQIPYSSFVVDTSLPLPLMIPRIIELCKDLFKNWGELDDSLFSVERVSGGITNLLLKVSVKEDTNKEVSVTVRLYGPNTEYVINREREILAIKYLSAAGFGAKLLGGFGNGMVQSFINARTLEPSDMREPKIAAQIARELGKFHKVDIPGSKEPQLWVDILKFYEKASTLTFEEPDKQKLFETISFEELHKEIIELREFTGLLNAPVVFAHNDLLSGNFMLNDEEEKLYLIDFEYGSYNYRGFDIGNHFNEYAGYDCDYSLYPSKEEQYHFIKHYLQPDKPDEVSIAEVESVFVETDAYKLASHLYWAIWAIIQARMSPIEFEYLGYFFLRYNEYKKQKPLTFSLVTSHLSASL***
